# Exploring a career pathway for home support workers in Ireland: a systematic scoping review of the international evidence

**DOI:** 10.3389/frhs.2024.1360920

**Published:** 2024-03-13

**Authors:** Elizabeth Morrow, Carmel Kelly, Clodagh Killeen, Edward Naessens, Mary Lynch

**Affiliations:** ^1^ Faculty of Nursing and Midwifery, Royal College of Surgeons Ireland, Dublin, Ireland; ^2^ Leading Healthcare Providers Skillnet, Skillnet Ireland, Dublin, Ireland

**Keywords:** home support workers, home care, workforce planning, career pathway, ageing population, person-centred care, ageing in place, digital health

## Abstract

**Background:**

In prosperous nations like Ireland, home support workers (HSWs) play an increasingly vital role in providing person-centred care to ageing populations. However, challenges such as workforce shortages, role ambiguity, low pay, and limited career advancement, hinder workforce development and career building.

**Method:**

A scoping review using key terms for “HSWs” and “career pathways” was conducted following the Joanna Briggs Institute Methodology, examining electronic databases (Web of Science, PubMed, MEDLINE, EMBASE, CINAHL, PscyINFO, Social Care Online, Social Sciences Citation Index). Inclusion criteria were applied, and a thematic analysis followed and inductive-deductive approach.

**Results:**

The review encompassed 261 relevant articles from different countries. Four key themes were identified: (1) Data-driven decision-making on the future workforce, (2) Attracting and developing a competent and motivated home support workforce, (3) Enhancing working lives and retention at every stage of career pathways, and (4) Crafting career pathways to improve quality and impact.

**Discussion:**

Leadership, collaboration, and data-driven decision-making across policy, research and practice are pivotal for expanding and enhancing home support. Emphasising a shift towards preventative self-management models, supported by digitally skilled and regulated HSWs, could enhance independence and quality of care. Clear career structures, professional development, and inclusive organisational environments are essential to attract, retain, and empower a competent and motivated workforce, fostering quality and impact.

**Conclusion:**

This scoping review provides foundational evidence to establish career pathways for HSWs, identifying key areas for development such as data collection, care model transformation, career progression structures, and systems for safety and quality improvement.

## Highlights

• This scoping review provides a comprehensive view of the evidence on career pathways for home support workers (HSWs) offering insights into key issues and areas for workforce development and career building.
• The evidence highlights the critical need to prioritise leadership, collaboration, and data-driven decision-making to shape the future of the home support sector in line with the needs of ageing populations.
• Clearer career structures could help to attract and retain HSWs, offering opportunities for core competency and advanced training, leadership development, and progression to more skilled work aligned to specific client group needs, and higher grades of renumeration.
• Strategies to cultivate successful careers and a thriving workforce should focus on safeguarding the rights, personal safety, and wellbeing of HSWs and clients as well as involving HSWs more in teams, organisations, and policy decisions.
• Fostering quality and impact in home support work requires developing systems for quality improvement and measures of service evaluation that recognise relational aspects of supportive care.

## 1 Introduction

Populations globally are ageing rapidly, with at least 35 countries expected to become “super-aged” by 2030. This classification refers to a situation where more than 1 in 6 citizens is over 65 years-of-age, such as the populations of Japan, South Korea, Germany, and Italy. Population ageing can be seen as one of the greatest successes of public health. However, a key challenge is not only the extension of life expectancy but to ensure those extra years are healthy and disability free for as long as possible: the notion of ageing well and independently at home. All countries face major challenges to ensure that their health and social systems are ready to make the most of this demographic shift.

At present prosperous countries are experiencing escalating demand for high-quality home-based support for elders, contributing to a mounting elder care crisis [Global Coalition on (1, 2)]. The United Nations Decade of Healthy Ageing 2021–2030 (3) signifies a concerted effort to address the needs of older people, their families, and communities worldwide with sustainable solutions.

Demand for home support services is driven by multiple socio-demographic factors, including rising numbers of older people with late-life dependency and complex health and care needs. Evidence suggests that person-centred integrated care closer to home can improve health outcomes and maintain independence for older individuals who want to age in place (4–6). Developments in digital health and remote healthcare provision also offer new opportunities for innovation in care and provision of compassion-based support in home settings (7, 8).

In Ireland a rapidly ageing population is projected to increase from 629,800 in 2020 to between 1.53 and 1.6 million by 2051 (9). This situation poses significant challenges to present healthcare and home-based care infrastructures. Despite a significant funding increase to Services for Older People, reaching €665 million in 2021 from €288 million in 2014, demand has surged. Over 20 million hours of home support were delivered to 55,043 service users in 2021 (10), with projections indicating a 50% rise in demand by 2030 (11).

In Ireland and other countries workforce supply challenges, are associated with the stigma of elder care and domestic work, low pay, and poor working conditions (5). A further demographic challenge looms, with many HSWs themselves ageing (42% of HSWs in Ireland were over 60-years-old in 2020). In many countries, rural and low-supply areas, such as districts with high rates of older people, face even worse shortages. Reliance on overseas recruitment and the prevalence of undeclared workers in some countries, has been a focus for political debate regarding the ethical treatment of migrant workers and the regulation of home support work. These pressing challenges underscore the need for comprehensive and transformative evidence-based solutions.

### 1.1 Rationale

This scoping review aims to investigate international evidence on home support workers (HSW) and career pathways and frameworks. The findings will inform strategies for workforce and career development in Ireland, enhancing the sector's capacity and capabilities. Recognising this is vital for both immediate and long-term population health, as well as the social and economic wellbeing of the nation.

### 1.2 Objectives

The aim of this scoping review was to provide a comprehensive depth and a balanced perspective of the emerging topic of career pathways for HSWs to inform future policy, research, and practice.

Objectives were:

1) To use a structured scoping review methodology to identify the most relevant evidence internationally on HSWs and career pathways and frameworks.
2) To synthesise available evidence to identify key themes, issues, and gaps in the evidence.
3) To generate actionable recommendations for policy, research and practice on a career pathway for HSWs.

### 1.3 Approach

The scoping review followed an approach originally proposed by Arksey and O'Malley (12), further enhanced by the work of Levac et al. (13) and consolidated in Joanna Briggs Institute guidelines (14, 15). Presentation of the scoping review conforms with PRISMA-ScR (Preferred Reporting Items for Systematic reviews and Meta-Analyses extension for Scoping Reviews) (16). Scoping reviews are useful for examining emerging or diverse sources of evidence on a topic to map evidence, concepts, theories, sources or knowledge gaps (17). Unlike a systematic review, scoping reviews do not focus on synthesizing results of studies based on a formal process of methodological appraisal to judge the quality of the evidence (15).

The approach follows five stages: Formulating research questions, Identifying relevant studies, Selecting, charting, collating, Summarizing, Reporting results (12, 18).

### 1.4 Definitions and scope

The scope of the review was refined by defining the two core concepts of the review based on existing terms and classifications used in the literature (18). A “home support worker” (HSW) was defined as an individual employed to provide support to a person (client or service user) in their private residence. Further detail of the various different terms for HSW is provided separately (Supplementary Material: Home support worker role terminology). A “career pathway” was defined as comprehensive development framework incorporating policy enhancements, best practices, and structural elements to develop the workforce and build careers. The approach emphasised purpose and principles of a career pathway (19, 20). Recognising the importance of economic evaluation in healthcare (21) a separate element of the scoping review (to be published separately) was to seek evidence on economic perspectives using key terms for economic methods and concepts (22, 23).

Table 1 shows the key search terms used in the literature searches.

**Table 1:** Key search terms.

Concept	Key search terms
(1a) Home support worker	Care worker Care manager Community care worker Domestic worker Domestic care staff Front-line care worker Home care Home care agency Homecare agency Home-care agency Home care services Homecare services Home-care services Home care support worker Homecare support worker Home-care support worker Home carer Home health aide Home help Home nursing Home support services In-home services In-home care Paid carer Personal assistant Professional carer Professional home health agency staff Senior care worker Support worker Unlicensed assistive personnel
(1b) Excluded terms	Community support worker Dental devices home care Foster home care Family support worker Healthcare assistant Home care services hospital-based Medical trainee Medical residency Out-of-home care Residency training Social worker Sexual orientation Veterinary
(2a) Career framework/pathway	Advanced training Appraisal Career Career development Career framework Career pathway Career progression Clinical supervision Coaching Continuing professional development Course Education Induction In-service training Experiential learning Levels of experience Mentorship Online learning On-the-job training Orientation Peer-learning Personal skills Practical training Practice learning Professional development Professional portfolio Qualification Shadowing Skills development Specialize Specialism Supervision Training Workplace education Workplace learning
(3) Economic perspectives	Conjoint analysis Contingent behaviour Contingent valuation Cost analysis Cost benefit Cost effective* Cost effective* analysis Cost of illness Cost outcome Cost utilit* Cost-effectiv* Cost-utilit* DALY DCE Discrete choice experiment Economic analys* Economic evaluation* Economic reviewEconomics Health impact assessment Health related quality of life Impact analys* Markov Mental Opportunity cost QALY QoL Quality adjusted life year Return on investment Revealed preference Social cost benefit Social prescribing Social return on investment SROI Stated preference Trade-off* Travel cost model

* Denotes a boolean wildcard function for truncation representing different word endings.

## 2 Materials and methods

Presentation of the methods follows PRISMA-ScR guidelines. A review protocol was not published for this scoping review. Ethical approval was not required.

### 2.1 Eligibility criteria (inclusions/exclusions)

**Figure 1:**
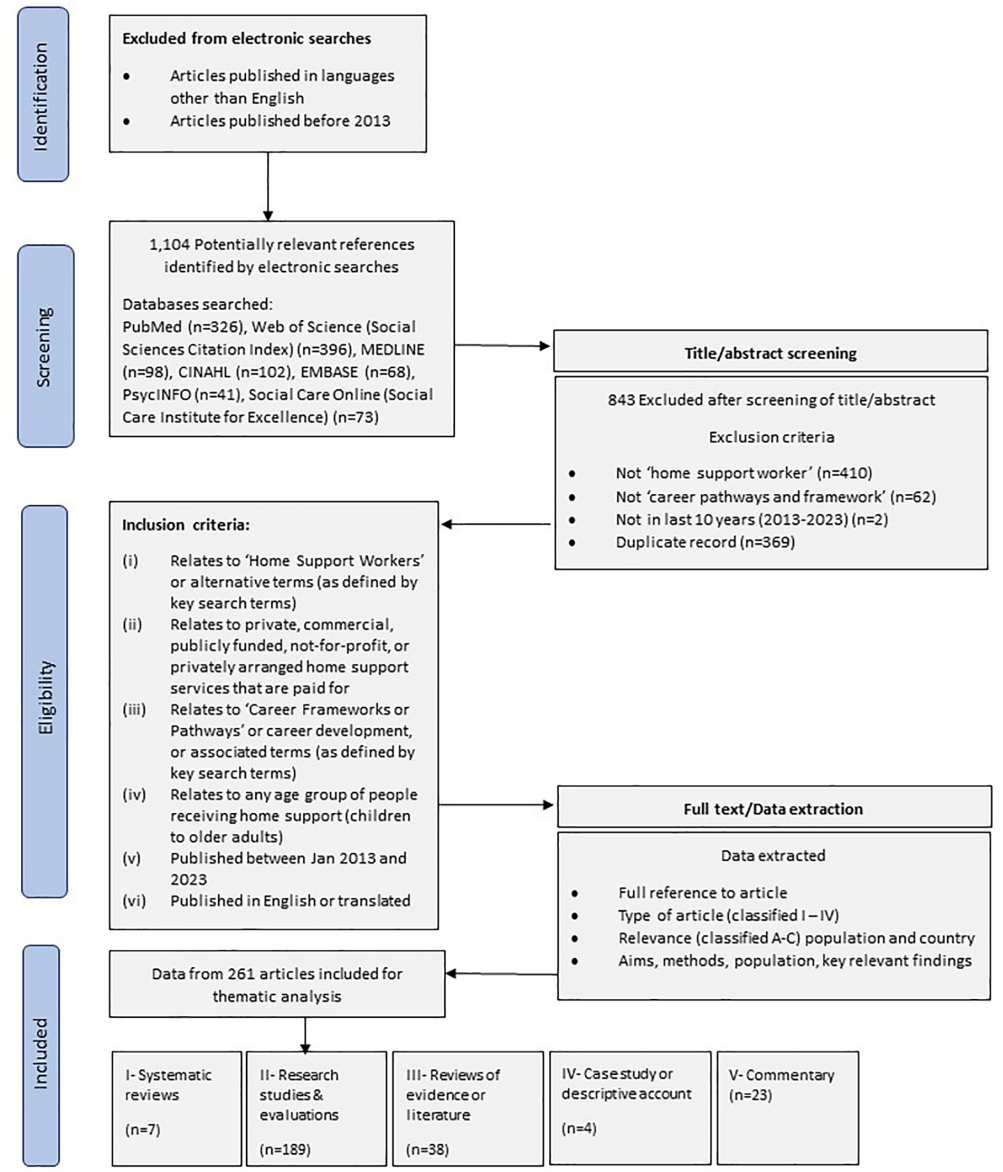
Inclusion flow diagram illustrating the search and screening process.

The review is inclusive of all literature published in English language and articles written in other languages when translations were available. To enhance relevance of the included articles, we limited years considered to publication in the last 10 years (2013–2023). Inclusion and exclusion criteria, shown in Figure 1, were developed from the literature.

### 2.2 Search process

**Figure 2:**
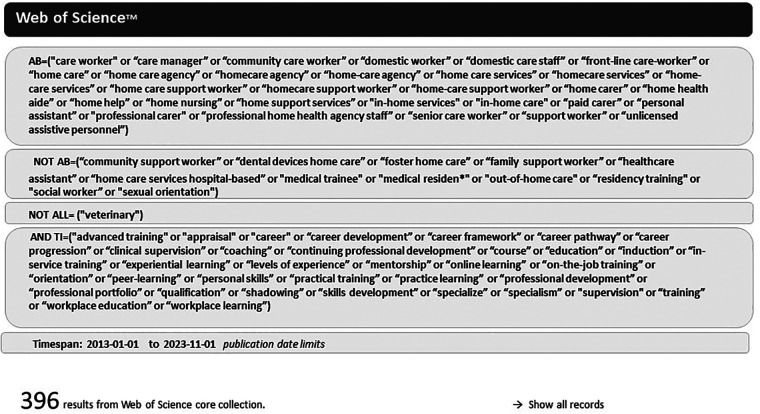
Search strategy (example of Web of science search).

Preliminary searches were undertaken using Google search in August 2023 to develop the review question (15). Searches of electronic databases included literature published in the last 10 years (Jan 2013–Nov 2023). Sources were Web of Science (covering over 12,000 high impact journals and the Social Science Citation Index), PubMed (biomedical literature from MEDLINE, life science journals, and books), EMBASE (medical research), CINAHL (nursing and allied health literature), PscycINFO (behavioural and social sciences), Social Care Online. Source were selected according to the review topic (24). In line with best practices the searches were performed by an experienced researcher (EM) and validated independently (EN, ML) (18). Figure 2 presents an example search strategy for Web of Science (Figure 2).

### 2.3 Screening

Figure 1 depicts the overall search process. A total of 1,104 articles were identified for screening based on title and abstract, using inclusion/exclusion criteria. Reasons for exclusion of articles were documented for rigor (16).

### 2.4 Data charting

Data from included articles were extracted into customised tables using Microsoft Word. Tables were piloted with 10 articles, with minor adjustments made. Data included (1) Full article reference, (2) Article type (categories below), (3) Article relevance (categories below), and (4) Summary of aims, methods, and key findings. Data charting included noting population group and context when relevant and training programmes (marked *TP in Supplementary Material: Bibliography).

### 2.5 Analysis

Descriptive analysis of the included articles used date of publication, country of publication, and basic classifications of quality and relevance. Articles were coded following the GRADE system (25) and an assessment of their relevance was based on study population and country (detail in Supplementary Material: Bibliography).

The thematic analysis applied an inductive-deductive approach to explore the research question and the data (17). The analysis followed the review process: familiarization to understand the focus of articles, identification of preliminary themes during data extraction, charting and development of a thematic code framework (Supplementary Material: Thematic code framework). Theme headings were derived from the data. Themes were checked and validated by team members resulting in four key themes, described below.

## 3 Results

The searches identified 1,104 potentially relevant articles. All were assessed for eligibility based on titles or abstracts. A total of 261 relevant articles were included (Supplementary Material: Bibliography). Included articles were 23.6% of those screened, indicating an accurate search with a high specificity (26). Table 2 provides a description of the included literature (Table 2).

**Table 2:** Description of the included literature.

Year of publication	Numbers of articles published on the topic increased steadily between 2013 (*n* = 14) and 2022 (*n* = 38), with 31 articles as of November 2023
Article types	I- Systematic review (2.6%, *n* = 7), II- Research or evaluation using recognised method (Quantitative, Qualitative or Mixed method) (72.4%, *n* = 189) III- Review of evidence or literature or policy, review submitted to a commission, review to inform a white paper (14.5%, *n* = 38) IV- Case study or descriptive account (1.5%, *n* = 4). V- Commentary: comment, editorial, discussion paper, roundtable discussion (8.8%, *n* = 23)
Relevance Grades (population/country)	A- Relates to home support workers in Ireland (or worldwide) (*n* = 14) B- Relates to a similar professional group or another country (*n* = 201) C- Relates to a similar professional group and another country (*n* = 46)
Article country	USA = 81 (31%), UK = 53 (20.3%) (England = 41, Wales = 3, Scotland = 2), Worldwide or international focus (*n* = 22), Canada = 21, Australia = 15, China = 10, Sweden = 10, Finland = 8, Taiwan = 7, Norway = 6. Three articles each: Japan, Singapore, Germany. Two articles each: Holland, Italy, Iran, Israel, Korea. One article each: France, Ireland, Belgium, Thailand, South Africa, Switzerland, Brazil, Turkey, Sub-Saharan Africa, Tunisia, Spain
Systematic reviews	Topics (*n* = 7): home carer training, supervision, and interventions on older people’s health and wellbeing (27); training and education needs of homecare workers for dementia and cancer support (28); communication skills training in dementia care (29); experiences of older LGBTQI + individuals receiving home care services (30); domestic helpers as frontline workers in China's home-based elder care (31); home care quality indicators using the Resident Assessment Instrument-Home Care (RAI-HC) (32); health of home care workers (33)
Research articles	Qualitative research (*n* = 73) (interviews, qualitative survey, focus groups, consensus building approaches, ethnography). Quantitative research (survey designs and experimental methods) (*n* = 47). Randomized controlled trial (RCT) methods (*n* = 5). Mixed method studies (*n* = 35). Programme evaluations (*n* = 26). Document analysis (*n* = 5). Model development (*n* = 3)

Table 3 summarizes key themes and subthemes and shows the number of articles in each theme (Table 3).

**Table 3:** Overview of themes in the literature.

Key themes	Subthemes (*n* = 29)	Articles (*n* = 261)
Data-driven decision-making on the future workforce	Subthemes (*n* = 8): Leadership and collaboration between policy, research and practice Upskilling and delegation (new enhanced roles) Models of home care provision Employee voice, engagement and involvement Workforce shortages, shortfalls and untapped strengths Digital technology and digital skills in home care Sector economic perspectives Public health, disaster preparedness, crisis management	(*n* = 95) 29 16 14 13 8 6 5 4
Attracting and developing a competent and motivated home support workforce	Subthemes (*n* = 8): Advanced or client group specific training (advanced/specialist HSWs) Core competencies training for home support work (early career HSWs) In-service learning opportunities (continuous professional development) Career development and progression systems Cost-benefits of employment and training Preparation, entry, early experiences (career starters) Leadership, governance, and management training (people management) Perceived career success (career achievers/leavers)	(*n* = 83) 35 12 12 9 7 6 1 1
Enhancing working lives and retention at every stage of career pathways	Subthemes (*n* = 7): Personal safety and wellbeing Job satisfaction and retention Safety in the home setting Care planning, care plans and coordination of care Inclusion and voice in teams, organisations, and policymaking Role clarity, preventing role drift and having clear boundaries Organisational economic perspectives	(*n* = 53) 15 13 8 5 5 4 3
Crafting career pathways to improve quality and impact	Subthemes (*n* = 6): Structural organisation of care and jobs (“organising for quality”) Careers of migrant workers Service quality indicators and measures Families and caregivers Service economic perspectives Interface with other services or providers	(*n* = 30) 13 6 4 4 2 1

### 3.1 Theme 1: data-driven decision-making on the future workforce

Leadership and collaboration between policy, research and practice: This prominent theme of the literature unfolds across various countries and time periods. In the US, the Health Workforce Research Center was established to address the long-term care needs of an aging population (34), as well as concerns about safety risks and health hazards (35–37). Leadership and collaboration was needed to establish preventive interventions, inform policy, education, and practice, and set a new course for research (38, 39). These calls to action are underpinned by well-documented challenges internationally associated with low pay and recognition (40, 41), stigma (42–44), work stress (45), and lack of quality standards (46–49). Leadership and multi-agency collaboration efforts focus on workforce planning and sector growth to comprehensively assesses issues in training, recruitment, and retention (50–54).

Upskilling and delegation (new enhanced roles): This theme conveys the need to safely equip HSWs with more advanced skills to transform the model of care, such as responding to the needs of older cancer patients (55, 56). Research has also demonstrated the positive impact of delegation of low risk medication support (57, 58) and home care following stroke (59). Upskilling is essential for improving quality and job satisfaction in the sector (60), provided that the fundamentals of care such as nutrition support and oral care are still met (61–64). There is good reason to believe that allowing time for HSWs to use their support skills, as well as to develop more advanced skills, could improve safety, quality of life for clients and generate healthcare system efficiencies (1, 65). Competencies for more advanced roles and more diverse skillsets need to be clearly defined (66–68), for example, in supporting exercise and reablement (69, 70).

Models of home care and support: Home support needs to expand and develop to encourage supported self-management and build on professional approaches that promote positive health outcomes and innovative interventions that reduce dependency (1, 71, 72). In particular dementia support is recognised as a crucial area for developing the model of home-based person-centred support in partnership with unpaid caregivers (73–78). The Global Coalition on Aging (79) explores relationship-based home care (an outcomes based or person-centred model) advocating for building a caregiving workforce with appropriate skills to deliver different levels of support (80, 81). Changes in the model of care towards client-centred care and reablement have been shown to be feasible (82–84), but the success of changes relates to organisational factors, such as determinants of staff health, service provision, and quality of care measures (69).

Employee voice, engagement and involvement: The literature consistently suggests that employees should be more involved in developing best practices, training, education, and home support workforce development initiatives (85–89). Research advocates a tailored approach, where employee involvement is routinely included yet undertaken flexibly to suit local needs and requirements (90, 91). Employee engagement in decisions about training and continuous professional development (CPD) needs to be encouraged to improve access and relevance (92–96).

Workforce shortages, shortfalls and untapped strengths: The challenge of managing a diverse workforce incudes older women balancing work and care responsibilities (97), and apprenticeship programmes (98). Research has looked into the best practice of employers with less than 10% staff turnover (99) and approaches that combine health and social care approaches to workforce development (100). Workforce issues in rural communities and retention challenges in these areas is an international concern (101–103). Modelling career success is advocated as a way of enhancing recruitment and retention (104).

Digital technology and digital skills in home care: Research has examined training and use of technologies in home support for heart failure patients (105) however the use of assistive technology, robotics, and technologies for optimal workflows is underexplored (106–108). HSWs digital skills are recognised as being crucial to effective use of digital health technologies (109). China's quality supervision indicators for Internet + home care based on the SERVQUAL model, offers a theoretical basis for quality supervision and continuous improvement in Internet + home care (110).

Sector economic perspectives: Research internationally highlights sector challenges of financial sustainability, pay equity, market challenges, and the cost-effectiveness of training programmes (111–113). Economic perspectives include de-investment decisions (114) and the economic benefits of health promoting interventions for prevention of functional loss and promoting quality of life (115).

Public health, disaster preparedness, crisis management: Research conveys the heightened risks and challenges faced by HSWs during the COVID-19 pandemic, and the urgent need for future supportive interventions and policies that address public health issues and involve the home support workforce in preparing for future events (116–118). COVID-19 related issues include economic unevenness across sectors and regions, altered landscapes of healthcare towards more remote and virtual services, increased demand for home-based solutions, and urgency in workforce improvements (119, 120). Solutions offered include sector reform in terms of workforce planning, improved terms and conditions, fair and sustainable funding, and strategies to prevent the perpetuation of existing health and healthcare inequalities.

### 3.2 Theme 2: attracting and developing a competent and motivated home support workforce

Advanced or client group specific training (advanced/specialist HSWs): Provision of advanced training is a strong theme of the international literature, as well as the development of core competencies in the home support workforce. Within these areas dementia is a key area for advance skills development (27–29, 76, 77, 121–136). In Taiwan trials of virtual reality-based training for dementia have had promising results (137).

Other areas of advanced training being explored are heart failure support (105, 116, 138–144), palliative care (145, 146), end-of-life support (147), mental health (148), hypertension care (149), long-term care (150), and diabetes support (151).

Core competencies training for home support work (early career HSWs): Looking across specific studies and training programmes provides an indication of the necessary core skills training for home support work that involves direct client contact. Topics are:

- Recognising medical emergencies and essential health and support needs (152–155)
- Person centred home support, empathy, and diversity awareness (LGBTQI+, neurodiversity) (30, 156, 157)
- Personal care training, ADL, IADL, infection control, oral health care (158–161)
- Personal assistance (instrumental activities, supervised activities) (160)
- Mental health awareness (depression, loneliness, social anxiety) (162)
- Safety in the home environment (clients and HSWs) including falls prevention (163)
- Communication with clients, cross-cultural communication, cultural competency in the home (164)
- Communication with health care professionals, other services and providers (165)
- Disability/different ability training (hearing loss, visual impairment, learning disability) (166, 167).

In-service learning opportunities (continuous professional development): Various initiatives are contextualised by a wider argument for the need to increase organisational support, supervisor support and peer assistance in the home support sector (168, 169). These arguments are supported by evidence that positive learning outcomes, such as knowledge gained and skills learned, are associated with greater career motivation (170–172) and consolidation of standardised training and competency-based requirements across organisations and sectors (173–176), as well as a climate for innovation (177). Research has shown mixed results for the effectiveness of decision aid training (178) and interprofessional education (179).

Career development and progression systems: Evidence shows multiple barriers to career development and progression in home support work (180, 181). Some researchers argue for equivalency programmes, which take into consideration experience and skills rather than only accepting specific qualifications, to facilitate workforce growth (182). There is some evidence to show the positive impact of appraisals on quality of care provided by support workers, but it is less clear whether appraisals or mentorship support career development or progression (183, 184). There is evidence internationally to show that having opportunities for training, CPD and a supportive work environment, supports employees in their career goals and commitment to stay in home care work (133, 171, 172, 185–187). What is clear is that some provider organisations take a more active approach to career progression than others, emphasising the importance of crafted career pathways for staff advancement and retention of employees and their skills within the organisation (188).

Cost-benefits of employment and training: Research has focused on the cost and benefits of specific courses and training programmes, with some attention to impact on workforce quality and retention issues (180, 181, 189). There is limited evidence of upward career mobility within healthcare occupations in the USA, and other countries, reinforcing the importance of clarifying career pathways for individuals in entry-level positions (190, 191). One training programme evaluation was able to demonstrate a significant reduction in costly adverse events and the positive impact of quality training on patient outcomes (192). Other studies highlight the impact of a workforce training intervention on value-based payment measures (193) and real-life constraints in training programme development (194).

Preparation, entry, early experiences (career starters): A range of factors are known to influence recruitment and early retention to home-based care roles including clarity about specific job roles and fair financial compensation (195, 196), as well as preparatory training, legal protection, and working conditions (31, 197–199).

Leadership, governance, and management training (people management): the need for better governance information and training is a worldwide issue (200). Research shows significant associations between the education level of long-term care administrators and quality indicators, suggesting that promoting further educational attainment of administrators in home support could improve the quality of services provided (201).

Perceived career success (career achievers/leavers): Low status of home care work hinders HSWs feeling valued in their work (202). One USA study showed that HSWs leaving their jobs were more educated, had higher income, and were more likely to be White ethnicity, suggesting low status and pay contribute to high turnover (203).

### 3.3 Theme 3: enhancing working lives and retention at every stage of career pathways

Personal safety and wellbeing: The physical and emotional nature of the work, lack of safety and support systems, as well as individual client and worker characteristics, contribute to work-related burden in home support work impacting job satisfaction and retention (204–211). Client behaviours in the home, such as occupational tobacco smoke exposure, can create significant health challenges for HSWs, requiring standard terms and agreements to be put in place with clients and families to safeguard employees (212). Workplace health promotion programmes for employees can improve health outcomes and reduce stress (213, 214). Personal social networks can also help HSWs to cope with demanding job roles and emotionally demanding elements of the work such as client bereavement (215–218).

Job satisfaction and retention: Factors underpinning staff motivation and retention include having clear employment terms and conditions (196, 219), as well as enhanced control and support in the role (220, 221), and a positive work culture and modelling successful careers (222–225). The meaning of the work, and recognising the joyful moments of the work, supported HSW's resilience during the COVID-19 pandemic (226). Other research from Germany, UK, Finland and Taiwan, underscores the significance of understanding the factors involved in job satisfaction for retaining employees by developing more tailored and effective job satisfaction strategies (227–230), particularly fair workload and intensity (231).

Safety in the home setting: Safeguarding personal and client safety is a crucial issue that influences job satisfaction and retention of workers in home environments (232). A fundamental client safety concern is the importance of clear organisational protocols and support for HSWs who may be responsible for clients who have acute symptoms so that HSW know when and how to seek emergency assistance for clients (233). Lack of information and education can cause other types of client safety risks, such as the use of physical restraints by family caregivers (234) and dementia-related mitigation interventions (235) or the reduction of falls risk (163, 236). Working in homes and traveling between clients, presents multiple occupational safety risks that can include violence, pests, and driving accidents (237–241).

Care planning, care plans and coordination of care and support: The provision of staff resources on culturally competent care in the home environment has been shown to improve coordination of care (242). Evidence suggests that systems for care planning and associated care planning tools need to recognise the importance of person-centred relational care and the provision of support that facilitates client self-management capabilities, alongside planning and monitoring the delivery of specific tasks such as personal care (243). Assessing, documenting and understanding the needs of specific client groups, such as people with heart failure, is a key issue for care quality, underscoring the need for disease-specific training, care planning, and improved communication of care plans (140, 244, 245).

Inclusion and voice in teams, organisations, and policymaking: Studies from Sweden, Ireland, Belgium, and the USA highlight various inclusion challenges including gender and lack of voice in policy debates (246–248). The literature notes that ambitions to transform healthcare towards more integrated care closer to home, requires HSW to be involved in decision-making, have access to more inclusive training, and to collaborate in initiatives and innovations alongside healthcare teams and other stakeholders (249, 250).

Role clarity, preventing role drift and having clear boundaries: Having clear roles and responsibilities is essential for setting boundaries and protecting HSWs from exploitation and harm (251, 252), including avoiding “market” and “gift” economies of care where HSWs routinely work over their paid hours (253, 254).

Organisational economic perspectives: Economic research on care managers in elderly care shows they are aware of efficiency demands but perceive their role as being to support staff to find ways to use resources better, rather than to enforce efficiency policy (160). Other research has looked into the reasons behind overstretched services, indicating a need to balance development aims with service delivery and to allocate funding for training not only funding for new posts (166, 167).

### 3.4 Theme 4: crafting career pathways to improve quality and impact

Structural organisation of care and jobs (“organising for quality”): Arguments in the literature stress the critical need to address time constraints, low pay, and inadequate training in the home care sector generally across different countries (255–258). More specifically, studies in the USA and Norway reveal discrepancies in assessing working conditions, scheduling and hours, pointing to the need for standardisation of assessment tools, and improved client data and organisational data collection, and communication regarding the organisation of care and the impact on occupational health outcomes (259–263). Planning tools and work optimisation models can help to assess care plan complexity, revealing congruencies and discrepancies in employee skills and capacity (264–266) and redesign work so that it supports staff recovery (267). Other research emphasises reorganisation of care and new roles for societal needs (268, 269) as well as building in time to deliver interpersonal sensitivity (270).

Careers of migrant workers: Migrant workers play an indispensable role in providing home support work, especially in high-income countries (271, 272). Female migrant domestic workers globally lack opportunities to develop their career and there is a need for tailored employer responses to support fairness and equality (273–276).

Service quality indicators and measures: Safety markers are one indicator of quality in home care and tools such as the International Classification for Patient Safety can be useful (277). In China, a unique set of 77 quality indicators tailored to the Chinese home care context were developed using a modified Delphi technique (278). Despite the widespread implementation of home care quality indicators derived from the Resident Assessment Instrument-Home Care (RAI-HC) research reveals insufficient testing of item validity and reliability in specific countries, underscoring the need for further investigations to enhance the reliability of quality measurement tools (32). However there is recognised need for more work on quality indicators and measures and their implementation (279).

Families and caregivers: Several studies highlight the importance of maintaining healthy family functioning by providing reliable home support (280, 281). Ethnic background can affect experiences of attaining paid help, highlighting the need for enhanced support and targeted interventions for ethnically diverse communities (282). The development of family-centered quality indicators for long-term care, could help to improve the experiences family members and caregivers have of home support services (283).

Service economic perspectives: Key economic issues at a service level relate to the cost benefit of training interventions for workforce retention (284) and to the benefits of service payment models that support quality of care by paying for relational care (285).

Interface with other services or providers: Research from Turkey highlights the complex challenges in medical, nursing, and social welfare aspects of support in the home environment and the necessity to coordinate care between providers (286).

## 4 Discussion

The findings emphasise the pressing need to transform home support services to address the needs of ageing populations. They identify many areas for improvement, such as data collection, care model development, training and qualifications, career structures, opportunities for in-service learning and CPD. Overall there is strong evidence to support the development of career pathways to improve recruitment, retention and job satisfaction, expanding workforce capacity and building successful careers.

### 4.1 Leadership, collaboration and data-driven decision-making are critical factors in the expansion and enhancement of home support

The international literature demonstrates the critical role of policy, research, and workforce planning processes in shaping the future of home support and transforming the model of home care in line with societal needs. Thus based on these results, policymakers are urged to set clear goals for home support to encourage leadership, collaboration and data collection across the sector (e.g., a minimum data set), prioritise the sector as part of the solution to overburdened healthcare systems, and address sector workforce development priorities. A well-skilled and well-regulated workforce will be invaluable to advancing home-based digital health, harnessing the advantages of AI technologies and implementing Human-AI Intelligent Caring (7, 8).

Part of developing a sustainable model of healthcare is to clarify expectations around home support. The aim should be to counter avoidable paternalism and dependency by promoting a model of preventative supported self-management as far as possible. Policy frameworks that help to bridge research gaps, enable different stakeholders to adopt systems thinking approaches, and find collaborative solutions, are crucial for sector growth. In this literature, dynamic workforce planning linked to outcomes is consistently recommended for positive sector-wide impact. Policymakers should be aware that international perspectives from diverse settings stress the need for collaborative and HSW-inclusive approaches to address supply and demand challenges. In particular, occupational health risks and challenges faced by HSWs all over the world highlight the urgency of implementing a protective human rights-based framework education, and standards to protect employee safety and rights as well as the rights of clients, unpaid carers and families.

Ongoing struggles internationally to combat stigma and role ambiguity associated with aged care work generally, underline the need to encourage professionalism and professional development interventions to challenge societal perceptions of home support. The health professions need to be made more aware of the marginalization of the home support workforce and encourage inclusion of HSW representatives in conversations about integrated care systems. With these strategies and investments, home support stands to be a dynamic sector that has a central role in integrated person-centred care.

### 4.2 Clearer career structures could help to attract and develop a competent and motivated home support workforce

**Figure 3:**
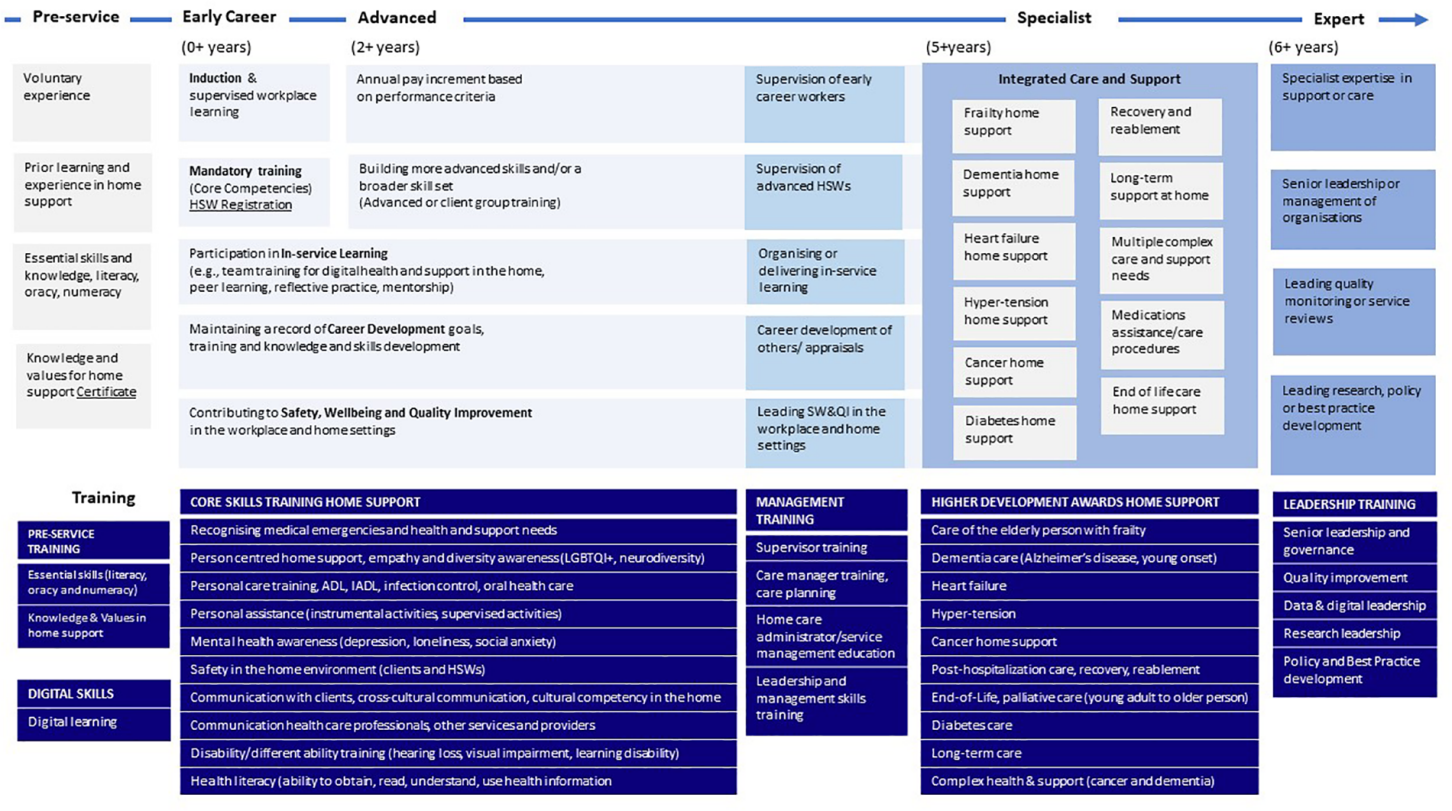
A tentative career structure for home support workers developed from the literature.

Drawing the evidence together visually, Figure 3 sets out a tentative career structure for HSWs that involves provision of a range of training (preparatory, digital skills, core competency, advanced training, leadership, governance and management), linked to career opportunities, and supported by CPD and in-service learning.

Progression towards advanced roles and specialised training, particularly in dementia, is a robust subtheme internationally, and marks a clear direction for workforce development (one area for advance roles illustrated by Figure 3). Positive impacts on the workforce are observed with career focused interventions like goal setting, carer training, and supervision frameworks, supported by studies from Taiwan, Sweden, and the USA. More advanced heart failure and end-of-life training, along with broader core competencies training for early-career HSWs, appear to be crucial components of expanding and enhancing the capabilities of a home support workforce nationally.

Home support providers should be made aware that successful careers are not only achieved through training and qualifications, but they are also underpinned by in-service learning opportunities and positive inclusive organisational environments. Building robust research into the cost-benefits of employment and training is critical for enabling stakeholders to understand the benefits of sector growth, drawing on global examples from Finland, Canada, the USA, China, and the UK. Entry experiences for career starters need exploration with stakeholders and applied research, to ensure role clarity, fair compensation, and preparatory training become standard practice within employing organisations and nations.

Education and training providers should be aware that gaps exist in leadership training at all levels, as well as governance, and management training in the home support sector. It is unclear whether this is due to lack of tailored courses, barriers to access, or low expectations and engagement with skills development and this requires further investigation at a national level. The impact of educational attainment on service quality remains unclear and requires future research. In many countries low status of home support work and poor pay hinder perceived career success, impacting recruitment and turnover. The status quo could be challenged by future research to establish clear pay grades and promote understanding and recognition for markers of progression and career success within the sector.

### 4.3 Strategies to cultivate successful careers and a thriving home support workforce

The results demonstrate the value of reframing HSW jobs as a career that has meaning, purpose, and structures for progression. Clearer career structures are likely to attract and retain a competent workforce. However, evidence on employee voice, engagement and involvement provides further important considerations to inform future research into successful careers and a thriving workforce.

The literature on improving working lives and retention of HSWs provides key insights to inform effective career pathways. International challenges in personal safety and wellbeing illustrate the importance of addressing grief, hazards, violence, and stress for a resilient workforce. Globally, job satisfaction and retention are influenced by factors such as control, support, and working conditions and these factors play into perceived career success. Thus, development of national policies for training, supervision, and risk mitigation will be vital for safety at home, impacting both job satisfaction and retention. Organisational economic perspectives stress the interplay between balancing efficiency demands, investing in career development, and funding availability for jobs, necessitating strategic approaches for HSW's workforce planning and progression. These insights contribute to a nuanced understanding for a relevant and impactful career pathway for HSWs. Nurturing a professional narrative could be a very powerful way of addressing many of the issues faced by HSWs. For example, in medicine and nursing e-portfolios provide a mechanism for spotting what is and is not working, as well as guiding professional development.

### 4.4 Fostering quality and impact in home support work

The evidence underlines the necessity of an effective career pathway to address structural challenges, like time constraints and low pay, and the negative effects on quality. Home support provider organisations and nations, could benefit from future research and development work that draws on a broad range of existing expertise in healthcare quality improvement, CPD in the health professions, career development theory, Lean Thinking techniques (increasing value and reducing waste), and organisational change methodologies. Essential elements of organising for quality include HSW tailored training and support to deliver increasingly complex and skilled work.

Economic perspectives in this topic area, stress recognising relational care in payment models. This is a direct challenge to the apparent efficiencies of time-task commissioning. They also emphasise the indicative cost benefits of investing in staff training and CPD, which may have benefits for healthcare savings. Ultimately, improved efficiencies and coordination at a health system level requires collaborative initiatives that bring HSWs together with healthcare providers and family caregivers for innovation and improvement.

Table 4 draws these discussion points together into a Recommendations Framework, which highlights the coordinated development of home support around four key areas, across policy, research and practice (Table 4).

**Table 4:** Recommendations framework.

Key areas	Policy	Research	Practice
Leadership, collaboration and data-driven decision-making	• Encourage data-driven decision-making to set goals for the sector in the short-term and longterm with an emphasis on supported self-management • Facilitate home support sector engagement in emergency health communication and crisis mitigation planning • Incentivise standardised data collection across the sector (minimum data set for service providers and a national register for HSWs) • Encourage digital skills and data awareness training across the sector • Establish centralised low cost or no-cost electronic certification and registration systems to facilitate CPD • Incentivise workforce expansion and enhancement to address priority areas of need (e.g., dementia, frailty, heart failure, end of life support)	• Use research as a collaborative space for interdisciplinary, interprofessional and inter-sector solutions to home support challenges • Develop workforce models and projections (incorporating large-scale workforce surveys) to inform decisions about workforce expansion and enhancement in priority areas (e.g., dementia, frailty, heart failure, end of life support) • Develop specifications for minimum data sets to inform flexible workforce planning in line with changing population needs • Establish test sites for new initiatives and approaches in areas such as upskilling and delegation • Develop systems to identify and reduce inequalities in provision and access to home support • Investigate sector economic perspectives, costs, benefits, and funding models for sustainable provision	• IEmphasise to staff and clients the importance of collecting reliable data and developing digital skills to improve service quality • Implement standardised digital data collection systems to capture data on client group need, staffing, and the organisation • Use data about client group needs to explore and adopt innovative home support models • Use data about staffing to improve the organisation of care, incorporating staff skills and preferences for work • Use data about the organisation to improve service quality, efficiency, and employee retention • Seek to work more closely with allied health professional services, including public health nurses, occupational therapists, day centre care, and convalescent care, to plan and manage person-centred care and support
Career structures that attract and develop a competent and motivated workforce	• Address workforce supply shortages by investing in national and local recruitment strategies that emphasise professionalism and career pathways • Develop policies for workforce development and careers to include roles and responsibilities for career development and progression • Develop national web-based and digital career development and progression systems for HSWs • Encourage agreement on role definitions, qualifications and career progression structures linked to training and pay • Encourage professionalism at all levels by facilitating access to core training, advanced training, leadership development and management training • Encourage best practice development across the sector, including guidance on the organisation of care, client and HSW safety, and career development	• Develop a structured career pathway for HSW progression, including core competencies, training, advanced or client group-specific training, and management, leadership and governance training • Develop best practice guidance on recruitment and retention drawing on the experiences of high performing providers • Investigate the effectiveness of different pay and financial incentive models to increase recruitment and retention • Capture the experiences of senior and established staff (role models) on career success and use these insights to enhance recruitment and retention • Explore the costs and benefits of training programmes on career progression and staff retention • Examine factors influencing drop-out rates and motivations to stay for early career HSWs	• Encourage pre-service preparation and essential skills development • Provide supervised learning for new recruits until core competencies are achieved • Develop programmes for all staff for In-service learning for continuous professional development, Career development, and Safety, wellbeing and quality improvement • Develop a strategy for supporting advanced training and specialist roles for more experienced staff • Establishing mentorship programmes and encourage peer support networks including support for migrant workers • Take up leadership and management training to improve organisation of care and a professional supportive organisational culture
Strategies to cultivate successful careers and a thriving workforce	• Develop sector policies and national guidance to safeguard the rights, personal safety and wellbeing of home support workers and clients • Encourage interventions to enhance job satisfaction and positive working relationships within organisations, as well as collaboration between organisations and sectors • Encourage strategies that promote career success and professionalism	• Investigate the impact of organisational safety cultures on retention and job satisfaction • Explore the effectiveness of support mechanisms in preventing burnout and intention to leave and promoting wellbeing • Produces evidence to support safe and effective role development, clarify role boundaries and prevent job drift	• Implement organisational support systems for supervised working at early career levels • Establish mechanisms to enhance job satisfaction • Develop trusted spaces for debriefing and sharing work experiences • Focus on creating quality work environments supporting autonomy and anti-discriminatory practices
Fostering quality and impact in home support work	• Develop policies and funding mechanisms based on assessing and addressing complexity of client care needs, responding to different levels of need, and changes in need over time • Encourage the sector to develop approaches to supported self-management alongside provision of essential support tasks • Promote staffing models that involve HSWs in finding solutions to enhance safety and job satisfaction • Integrate home support services into broader health systems, encouraging collaboration for quality and efficiency • Use policy to encourage spread of effective interventions that target quality and impact	• Investigate the impact of staffing policies and ways of working (e.g., supervised learning and delegated care) on safety, job satisfaction, employee involvement and overall care quality • Explore effectiveness of different home care and support provision models in meeting client needs, in particular the safe and ethical use of digital technologies for supported self-management at home • Research cost benefits of investing in staff training and expanding skill sets and the range of support provided by the sector • Produce evidence to support implementation, upscaling or roll-out of effective interventions to improve quality and impact	• Encourage prospective employees to gain essential skills in literacy, oracy, numeracy, and digital skills and to build relevant experience for a career in home support • Develop staffing policies with involvement from employees, focusing on staff and client safety, job satisfaction and retention • Emphasise sufficient time for client interactions and interpersonal sensitivity (relational care) in the organisation and measurement of services • Encourage collaboration and coordination between home support workers (peer support), with other service providers, and with other professions and sectors • Share best practice with others and seek to adopt and adapt effective interventions to foster quality and impact

## 5 Conclusion

This scoping review highlights the importance of establishing a clear career pathway for home support workers in Ireland and other countries with ageing populations. The review identifies key areas for future focus, such as data collection, digital integration, care model transformation, career structures, training, and leadership development. It stresses the importance of comprehensive best practices, including safety measures, a range of training, and collaborative initiatives, for a high quality and sustainable home support workforce.

The recommendations framework developed for policy, research, and practice provides a set of clear guidelines for developing a home support workforce. Investing in data and decision-making is crucial for shaping the future of home support and transforming the model of care in line with societal needs. Attracting and developing a competent and motivated home support workforce is essential for expanding capacity, enhancing skills, improving job satisfaction, and achieving the goal of providing effective home-based support. The results stress the importance of comprehensive best practices, including safety measures, a range of training, and collaborative initiatives, for a high-quality and sustainable home support workforce.

However, there are limitations to this scoping review, such as generalisability, publication bias, scope, implementation challenges, and long-term impact. Therefore, ongoing monitoring and evaluation are necessary for sustainability and effectiveness of specific interventions. Furthermore the recommendations framework developed for policy, research, and practice must be validated as a Green Paper (287), with a range of local stakeholders across Ireland, before being developed into a White Paper (due April 2024) that reflects and responds to the national context and needs of the population. These recommendations can be discussed by stakeholders internationally and inform career pathways in other countries and contexts.

## Data Availability

The original contributions presented in the study are included in the article/Supplementary Material, further inquiries can be directed to the corresponding author.
