# Supplementary material for: Exploring a career pathway for home support workers in Ireland: a systematic scoping review of the international evidence
Source: Front Health Serv. 2024 Mar 13;4:1360920. doi: 10.3389/frhs.2024.1360920 (PMC10967662; doi:10.3389/frhs.2024.1360920)
Supplement: Supplementary file 1 [file Datasheet1.zip › Data Sheet 1_v1/Bibliography of included literature.DOCX]

**Supplementary Material- Bibliography of included literature**

**Evidence Grades:**

I- Systematic Review

II- Research or evaluation using recognised method (Quantitative, Qualitative or Mixed method)

III- Review of literature or policy, review of evidence, submitted to a commission or to develop a white paper

IV- Case study, descriptive account

V- Commentary: comment, editorial, discussion paper, roundtable discussion

**Relevance Grades (Country):**

1. Relates to home support workers in Ireland (or worldwide)
2. Relates to a similar professional group or another country
3. Relates to a similar professional group and another country

| **Full reference of article** | **Evidence Grade** | **Relevance (Country)** | **Theme**  **Subtheme** | **Summary of Key Findings or Insights** |
| --- | --- | --- | --- | --- |
| Cooper, C., Cenko, B., Dow, B., & Rapaport, P. (2017). A systematic review evaluating the impact of paid home carer training, supervision, and other interventions on the health and well-being of older home care clients. *International psychogeriatrics*, *29*(4), 595–604. https://doi.org/10.1017/S1041610216002386 | I- Systematic review | A- Home care older people, dementia  (worldwide) | Theme 1- Advanced or Client Group Specific Training (care of the elderly) | - This systematic review analyses ten papers described eight interventions. The six quantitative evaluations used diverse outcomes that precluded meta-analysis. In the only quantitative study (a cluster Randomized Controlled Trial), rated higher quality, setting meaningful goals, carer training, and supervision improved client health-related quality of life. The interventions that improved client outcomes comprised training with additional implementation, such as regular supervision and promoted care focused on clients' needs and goals. - Qualitative synthesis of four studies shows intervention elements carers valued were ‘greater flexibility to work to a needs-based rather than a task-based model’, ‘learning more about clients’, and ‘improved communication with management and other workers’. - There is a dearth of evidence regarding effective strategies to improve how home care is delivered to older clients, particularly those with dementia. More research in this sector including feasibility testing of the first home care intervention trials to include health and life quality outcomes for clients with more severe dementia is needed. |
| Newbould L, Samsi K, Wilberforce M. (2022) Developing effective workforce training to support the long-term care of older adults: A review of reviews. Health Soc Care Community. 2022 Nov;30(6):2202-2217. doi: 10.1111/hsc.13897. | III- Review of literature | A- Learning intervention to support care of older adults (worldwide) | Theme 1- Advanced or Client Group Specific Training (care of the elderly) | - This review of reviews aimed to identify and synthesise evidence to support the design of learning interventions for non-registered practitioners supporting older people in long-term care (people's own homes, hospices or residential/nursing care). - The objectives were to inform the analysis part of the Analysis, Design, Development Implementation and Evaluation framework by finding evidence on the following five components of learning: content, format (teaching strategies and resources/media), structure, contextual factors (barriers and enablers) and measures used when monitoring the effectives of learning. Databases searched included Pro-quest (ASSIA), Scopus, Ovid (PsycINFO, Medline, Embase and Social Policy and Practice), SCIE Online and Cochrane Reviews and reference searching, with the last search being conducted in April 2021. Fifteen papers were identified as eligible for inclusion. - Most of the interventions aimed to improve dementia care (n = 10), with others exploring LGBT+ competency (n = 2), or other forms of professional development (n = 3). Common features of effective learning included a multifaceted approach, with in-practice learning being blended with additional implementation strategies (e.g. supervision) and didactic learning/worksheets. - An important contextual factor was working within an organisational culture which supported shared learning and reflection. This may also help encourage engagement with training, where staff are unwilling to attend if it may compromise care delivery. Concludes that future research should focus on the characteristics of trainers and the structure of learning, with more research being needed in in mental and physical morbidities outside the remit of dementia to improve the overall quality of the social care workforce. |
| Cunningham N, Cowie J, Watchman K, Methven K. (2020) Understanding the training and education needs of homecare workers supporting people with dementia and cancer: A systematic review of reviews. *Dementia*. 2020;19(8):2780-2803. doi:10.1177/1471301219859781 | I- Systematic review | B- Training for dementia and cancer care (worldwide) | Theme 1- Advanced or Client Group Specific Training (complex health needs, cancer and dementia) | - Many people with dementia, supported by family carers, prefer to live at home and may rely on homecare support services. People with dementia are also often living with multimorbidities, including cancer. The main risk factor for both cancer and dementia is age and the number of people living with dementia and cancer likely to rise. Upskilling the social care workforce to facilitate more complex care is central to national workforce strategies and challenges. Training and education development must also respond to the key requirements of a homecare workforce experiencing financial, recruitment and retention difficulties. - This systematic review of reviews provides an overview of dementia and cancer training and education accessible to the homecare workforce. Findings reveal there is a diverse range of training and education available, with mixed evidence of effectiveness. Key barriers and facilitators to effective training and education are identified in order to inform future training, education and learning development for the homecare workforce supporting people with dementia and cancer. |
| Sung, H. C., Su, H. F., Wang, H. M., Koo, M., & Lo, R. Y. (2021). Psychometric properties of the dementia knowledge assessment scale-traditional Chinese among home care workers in Taiwan. *BMC psychiatry*, *21*(1), 515. https://doi.org/10.1186/s12888-021-03530-6 | II- Research (Quant) | B- Assessment of dementia knowledge in home care workers (Taiwan) | Theme 1- Advanced or Client Group Specific Training (dementia training) | - The Dementia Knowledge Assessment Scale (DKAS) is a reliable and valid measurement of dementia knowledge for diverse allied health professionals, but its traditional Chinese version has not been formally validated yet. The purpose of this study was to translate the DKAS from English to traditional Chinese and evaluate its psychometric properties among home care workers in Taiwan. - The DKAS scale was translated into traditional Chinese through a forward translation and back translation process following the cross-cultural translation guideline. A total of 285 home care workers in eastern Taiwan were recruited using a convenience sample. A total of 252 participants completed the questionnaires, giving a response rate of 88.4%. We tested the construct validity by confirmatory factor analysis (CFA) and evaluated the reliability by internal consistency. - The results of the CFA supported the 25-item, four-factor model for the DKAS-TC. The DKAS-TC achieved a good overall Cronbach's alpha of .93 and McDonald's omega of 0.94 with acceptable subscales McDonald's omega ranged from .77 to .82. - The DKAS-TC has adequate construct validity and reliability and can serve as an assessment tool to evaluate the knowledge level of home care workers in a dementia training program in Taiwan. The dementia knowledge level among home care workers in Taiwan was inadequate. There is a need for developing suitable dementia care training tailored to their learning needs and educational levels, and to improve their quality of care for those with dementia. |
| Eggenberger, E., Heimerl, K., & Bennett, M. I. (2013). Communication skills training in dementia care: a systematic review of effectiveness, training content, and didactic methods in different care settings. *International psychogeriatrics*, *25*(3), 345–358. https://doi.org/10.1017/S1041610212001664 | I- Systematic review | B- Communication training carers (USA) | Theme 1- Advanced or Client Group Specific Training (dementia) | - Caring for and caring about people with dementia require specific communication skills. Healthcare professionals and family caregivers usually receive little training to enable them to meet the communicative needs of people with dementia. - This review identifies existent interventions to enhance communication in dementia care in various care settings. Searches of MEDLINE, AMED, EMBASE, PsychINFO, CINAHL, The Cochrane Library, Gerolit, and Web of Science for scientific articles reporting interventions in both English and German. An intervention was defined as communication skills training by means of face-to-face interaction with the aim of improving basic communicative skills. Both professional and family caregivers were included. The effectiveness of such training was analyzed. Different types of training were defined. Didactic methods, training content, and additional organizational features were qualitatively examined. - The review included 12 trials, involving a total of 831 persons with dementia, 519 professional caregivers, and 162 family caregivers. Most studies were carried out in the USA, the UK, and Germany. Eight studies took place in nursing homes; four studies were located in a home-care setting. No studies could be found in an acute-care setting. - The authors provide a list of basic communicative principles for good communication in dementia care. Didactic methods included lectures, hands-on training, group discussions, and role-play. - This review shows that communication skills training in dementia care significantly improves the quality of life and wellbeing of people with dementia and increases positive interactions in various care settings. Communication skills training shows significant impact on professional and family caregivers' communication skills, competencies, and knowledge. Additional organizational features improve the sustainability of communication interventions. |
| Zabihi, S., Duffy, L., Kelleher, D., Lord, K., Dar, A., Koutsoubelis, F., Banks, S., Rapaport, P., Mason, C., Vickerstaff, V., Barber, J. A., Manthorpe, J., Walters, K., Lang, I., Rockwood, K., Duggan, S., Kales, H., & Cooper, C. (2022). Feasibility and acceptability of NIDUS-Professional, a training and support intervention for homecare workers caring for clients living with dementia: a cluster-randomised feasibility trial protocol. BMJ open, 12(12), e066166. https://doi.org/10.1136/bmjopen-2022-066166 | II- Programme evaluation | B- Training programme for dementia care (England) | Theme 1- Advanced or Client Group Specific Training (dementia) | - Most people living with dementia want to remain living in their own homes and are supported to do so by family carers and homecare workers. There are concerns that homecare is often unable to meet the needs of this client group, with limited evidence regarding effective interventions to improve it for people living with dementia. - This article explains work to develop a training and support programme for homecare workers (NIDUS-Professional) [*TP] to be delivered alongside support sessions for people living with dementia and their family carers (NIDUS-Family). The study was to assess (1) its acceptability among homecare workers and employing agencies, and (2) the feasibility of homecare workers, people living with dementia and their family carers completing the outcomes of intervention in a future randomised controlled trial. - This is a cluster-randomised (2:1) single-blind, multisite feasibility trial. The aim is to recruit 60-90 homecare workers, 30-60 clients living with dementia and their family carers through 6-9 English homecare agencies. In the intervention arm, homecare staff will be offered six group sessions on video call over three months, followed by monthly group sessions over the subsequent three-month period. Outcome measures will be collected at baseline and at six months. |
| Su, H. F., Koo, M., Lee, W. L., Sung, H. C., Lee, R. P., & Liu, W. I. (2021). A dementia care training using mobile e-learning with mentoring support for home care workers: a controlled study. *BMC geriatrics*, *21*(1), 126. https://doi.org/10.1186/s12877-021-02075-3 | II- Programme evaluation | B- Home care support worker dementia training (Taiwan) | Theme 1- Advanced or Client Group Specific Training (dementia) | - Caring for older adults with dementia at home can be challenging for home care workers. There is a need to develop suitable training for home care workers to improve the quality of dementia care. This study evaluated a 12-week dementia care training including mobile e-learning, social networking, and mentoring support group meetings on the dementia care knowledge, attitude, and competence of home care workers. - This controlled study involved 140 home care workers from two home care agencies, which were selected from 12 home care agencies in eastern Taiwan. The two home care agencies were randomly allocated to either the intervention group or the control group. The intervention group received mobile e-learning, mentor-led online social support networking, and monthly face-to-face mentoring support group meetings. Participants in the control group received 8-h conventional lectures. The primary outcomes were knowledge, attitude, and competence in dementia care. Questionnaires consisting of the Dementia Knowledge Assessment Scale, Approaches to Dementia Questionnaire, and Sense of Competence in Dementia Care Staff scale were administered to the participants at three time points (baseline, end of the 12-week intervention, and 12 weeks after the end of the intervention). - Generalized estimating equation analyses showed that the intervention significantly improved the knowledge, attitude, and competence of home care workers on dementia care. The effects remained significant even 12 weeks after the end of the intervention. Mobile e-learning and an online environment provides a platform that is self-directed, flexible, accessible, and cost-effective for training home care workers. - These findings provide a call to action for nurse educators and policy makers to re-design existing dementia care training for home care workers to meet the critical home care needs of a growing dementia population. |
| Fallahpour, M., Borell, L., Sandberg, L., & Boström, A. M. (2020). Dementia Care Education Targeting Job Strain and Organizational Climate Among Dementia Care Specialists in Swedish Home Care Services. *Journal of multidisciplinary healthcare*, *13*, 85–97. https://doi.org/10.2147/JMDH.S214378 | II- Programme evaluation | B- Dementia care training and impact on job strain (Sweden) | Theme 1- Advanced or Client Group Specific Training (dementia) | - An increasing number of older people live at home with various limitations, such as dementia, requiring well-educated and trained home care staff to meet their complex care needs. Dementia care specialists working in home care service have reported high levels of job strain in comparison with home care staff in general. - This pilot study aimed to evaluate the effects of a dementia care education model targeting self-reported job strain and organizational climate, among dementia care specialists in home care service. A quasi-experimental, one-group pretest- posttest design was applied, with 12 months' follow-up. Participants were dementia care specialists who worked in home care service (n=34 baseline; n=30 follow-up). The data were collected using the Strain in Dementia Care Scale and Creative Climate Questionnaires, administered pre- and post-intervention. The intervention applied an educational model based on previous research in dementia care and a person-centered approach. - The educational model was implemented in the context of home care services. Of 34 participants at baseline, only 21 responded to the questionnaires, due to a drop off of 13 participants and recruitment of 9 new participants (follow-up=30). A significantly reduced perceived job strain among the participants was detected, indicating perceiving less difficulty in "Balancing competing needs" when caring for the older persons. No significant difference was found in the self-reported creative organizational climate between the two occasions. - The educational model could be integrated into dementia home care and it seemed to reduce job strain among dementia care specialists. Future studies are needed to evaluate the effects of the intervention using a stronger study design and a larger sample. |
| Gaugler JE, Hobday JV, Robbins JC, Barclay MP. (2016) Direct Care Worker Training to Respond to the Behavior of Individuals With Dementia: The CARES® Dementia-Related Behavior™ Online Program. Gerontol Geriatr Med. 2016 Jan-Dec;2:2333721415626888. doi: 10.1177/2333721415626888. PMID: 26894209; PMCID: PMC4755277. | II- Programme evaluation | B- Direct care worker dementia training (USA) | Theme 1- Advanced or Client Group Specific Training (dementia) | - Only a handful of online training programs are available for direct care workers (DCWs) to acquire the strategic skills needed to improve dementia care in instances of challenging or inappropriate behavior. - Utilizing pre- and post-test data from a convenience sample of 40 DCWs, the present study sought to determine (a) whether DCWs’ knowledge of responding to dementia-related behavior increased following participation in the CARES® Dementia-Related Behavior™ Online Training Program (or CARES® Behavior) and (b) if CARES® Behavior was acceptable and useful. - The average number of correct scores on a dementia care knowledge measure was significantly higher among DCWs after viewing the online modules when compared with pre-test scores (*p* < .01). Descriptive empirical and open-ended data also suggested that the interactive, “real-world” content of CARES® Behavior was feasibly delivered online, acceptable, and may influence how DCWs deliver clinical care to individuals with dementia-related behavior. |
| Guerrero, L. R., Shim, A., Gans, D., Schickedanz, H. B., & Tan, Z. S. (2019). Training for In-Home Supportive Services Caregivers in an Underserved Area. *Journal of health care for the poor and underserved*, *30*(2), 739–748. https://doi.org/10.1353/hpu.2019.0053 | II- Programme evaluation | B- Dementia training for home care workers (USA) | Theme 1- Advanced or Client Group Specific Training (dementia) | - Despite the complexity of care associated with older adults who have been diagnosed with Alzheimer's disease and related dementias, many in-home paid caregivers receive little to no training in competent care for these older adults. The In-Home Supportive Services program in California provides care for elderly, blind, and disabled people with the goal of ensuring the care recipients may remain safely in their own homes. Yet, these caregivers are not required to receive training in any specific disease or condition prior to providing caregiving services. - A Geriatrics Workforce Enhancement Program and the Riverside County Department of Social Services jointly developed and implemented three different interactive, community-based, knowledge and skills-based training courses for IHSS caregivers [*TP]. - Analysis of pre/post course evaluations across all three courses demonstrated statistically significant gains in knowledge and self-rated skills among the participants. |
| Smith, R., Ooms, A., & Greenwood, N. (2017). Supporting people with young onset dementia and their families: An evaluation of a training course for care workers. *Nurse education in practice*, *27*, 7–12. https://doi.org/10.1016/j.nepr.2017.08.007 | II- Programme evaluation | B- Training for young onset dementia (England) | Theme 1- Advanced or Client Group Specific Training (dementia) | - This article reports the findings of an evaluation of a training course for care workers who care for people with dementia in the community. Twenty-four care workers participated in the training which took place in London and Surrey, United Kingdom. - The training had a significant positive impact on participants' confidence in understanding the experiences and social care needs of people with young onset dementia (YOD) and their families. Participants also perceived that the training would help them improve their working practice by furthering their understanding of practical approaches to supporting and caring for people with dementia in general. Additionally, participants reported many ways in which they perceived being able to specifically support and empower people with YOD. - It was concluded that the short training course improved knowledge and confidence for care workers on dementia care, and specifically in understanding how to support people with YOD and their families. Dementia specific training should be considered by service managers as a way of potentially increasing care worker job satisfaction. |
| Yang, Y. Y., Yang, Y. P., Chen, K. M., Wang, C. J., Chang, S. H., & Wang, J. J. (2023). A Feasibility Evaluation of the Need-Centered Watch-Assess-Need Intervention-Think Education and Training Program for Behavioral and Psychological Symptoms of Dementia. *The journal of nursing research : JNR*, *31*(2), e266. https://doi.org/10.1097/jnr.0000000000000548 | II- Programme evaluation | B- Care home training for dementia (Taiwan) | Theme 1- Advanced or Client Group Specific Training (dementia) | - Despite the high prevalence of dementia among residents living in long-term care facilities in Taiwan, most care providers in these facilities have not received adequate training to deal with the behavioral and psychological symptoms of dementia (BPSD). An original care and management model for BPSD has been developed, and model-based recommendations for an education and training program have been made. However, empirical testing has not yet been conducted to determine the efficacy of this program. - This study was designed to evaluate the feasibility of using the Watch-Assess-Need intervention-Think (WANT) education and training program for BPSD in long-term care settings. A mixed-method design was used. Twenty care providers and 20 corresponding care receivers (residents with dementia) from a nursing home in southern Taiwan were enrolled. Data were collected using a variety of measurement tools, including the Cohen-Mansfield Agitation Inventory, Cornell Scale for Depression in Dementia, Attitude towards Dementia Care Scale, and Dementia Behavior Disturbance Self-efficacy Scale. Qualitative data, including care-provider perspectives on the efficacy of the WANT education and training program, were also collected. Repeated measures were conducted on the results of quantitative data analysis, whereas content analysis was performed on the results of qualitative data analysis. - Findings indicate that the program relieves agitated behavior, alleviates depression in those with dementia, and enhances care-provider attitudes toward dementia care. However, no significant improvement was found in self-efficacy among the care providers. In terms of qualitative outcomes, care providers indicated they perceived improved self-efficacy in managing BPSD, improved ability to view problems from a more need-centered perspective, improved attitudes toward dementia and patients' BPSD, and decreased care burden and stress. - The study found the WANT education and training program to be feasible in clinical practice. Because of this program's simple and easy-to-remember characteristics, it is recommended that it be vigorously promoted to care providers in both long-term institutional and home care settings to help them effectively address the BPSD. |
| Yeh, J., Pond, B., Beld, M., Garcia, A., Mauricio, J., Mata-Pacheco, J., Eldridge, C., & Ross, L. (2023). Enhancing Dementia Knowledge and Self-Efficacy of In-Home Supportive Services Caregivers Through Online Training. Journal of applied gerontology : the official journal of the Southern Gerontological Society, 42(4), 617–626. https://doi.org/10.1177/07334648221144023 | II- Programme evaluation | B- In-home supportive service training for dementia (USA) | Theme 1- Advanced or Client Group Specific Training (dementia) | - California's In-Home Supportive Services (IHSS) program provides vital home care to low-income consumers, some of whom live with Alzheimer's disease or related dementias (ADRD). Yet, most IHSS caregivers receive little or no training in dementia care. - This article presents preliminary descriptive results of the IHSS + ADRD Training Project, a 10-week, competency-based, dementia training program [*TP], delivered online, for IHSS caregivers, in Alameda County. - Increase in dementia knowledge and self-efficacy at training completion supports the importance of continuing and expanding this work. |
| Goh, A.M.Y., Polacsek, M., Malta, S. *et al.* (2022) What constitutes ‘good’ home care for people with dementia? An investigation of the views of home care service recipients and providers. *BMC Geriatr* **22**, 42. https://doi-org.proxy.library.rcsi.ie/10.1186/s12877-021-02727-4 | II- Research (Qual) | B- Dementia care training in home care (Australia) | Theme 1- Advanced or Client Group Specific Training (dementia) | - This study explored what people receiving and providing care consider to be ‘good’ in-home care for people living with dementia. The study involved 36 in-depth interviews and two focus groups with key stakeholders in Australia in the first quarter of 2018. Participants included those receiving care (4 people living with dementia, 15 family carers) or providing care (9 case managers, 5 service managers, 10 home care workers). Qualitative thematic analysis was guided by Braun and Clarke’s six-step approach. - Consensus was reached across all groups on five themes considered as important for good in-home dementia care: 1) Home care workers’ understanding of dementia and its impact; 2) Home care workers’ demonstrating person-centred care and empathy in their care relationship with their client; 3) Good relationships and communication between care worker, person with dementia and family carers; 4) Home care workers’ knowing positive practical strategies for changed behaviours; 5) Effective workplace policies and workforce culture. The results contributed to the co-design of a dementia specific training program [*TP] for home care workers. - The authors conclude that it is crucial to consider the views and opinions of each stakeholder group involved in providing/receiving dementia care from home care workers, to inform workforce training, education program design and service design. Results can be used to inform and empower home care providers, policy, and related decision makers to guide the delivery of improved home care services. |
| Leverton, M., Burton, A., Beresford-Dent, J., Rapaport, P., Manthorpe, J., Mansour, H., Guerra Ceballos, S., Downs, M., Samus, Q., Dow, B., Lord, K., & Cooper, C. (2021). 'You can't just put somebody in a situation with no armour'. An ethnographic exploration of the training and support needs of homecare workers caring for people living with dementia. *Dementia (London, England)*, *20*(8), 2982–3005. https://doi.org/10.1177/14713012211023676 | II- Research (Qual) | B- Home care dementia training and support (England) | Theme 1- Advanced or Client Group Specific Training (dementia) | - Homecare workers carry out complex work with people living with dementia, while under-supported, undervalued and undertrained. This ethnographic study explores the skills, training and support needs of homecare workers supporting people living with dementia. - The study involved 82 interviews with people living with dementia (n = 11), family caregivers (n = 22), homecare staff (n = 30) and health and social care professionals (n = 19) and conducted 100-hours of participant observations with homecare workers (n = 16). Interview and observational findings were triangulated and analysed data thematically. - Four themes: 1) 'Navigating the homecare identity and role': describing challenges of moving between different role identities and managing associated expectations, 2) 'Developing and utilising relational and emotional skills': boundaries between caring and getting emotionally involved felt blurred and difficult to manage, 3) 'Managing clients who resist care': homecare workers experienced clients' reactions as challenging and felt "thrown to the wolves" without sufficient training, and 4) 'Drawing on agency and team support': homecare work could be isolating, with no shared workplace, busy schedules and limited opportunity for peer support. - It is important that training and support for homecare workers addresses the relational, emotional and rights-based aspects of the role. Where a flexible, responsive, person-centred service is required, corresponding training and support is needed, alongside organisational practices, taking account of the broader context of the homecare sector. |
| Morgan, D., Kosteniuk, J., O’Connell, M., Dal Bello-Haas, V., Stewart, N. &  Karunanayake, C. (2016) Dementia-related work activities of home care nurses and aides: Frequency, perceived competence, and continuing education priorities, Educational Gerontology, 42:2, 120-135, DOI: 10.1080/03601277.2015.1083390 | II- Research (Quant) | B- Dementia training in home care (Canada) | Theme 1- Advanced or Client Group Specific Training (dementia) | - An understanding of the specific dementia learning needs of home care staff is needed to plan relevant continuing education (CE) programs and supports. The study's objective was to examine frequency and perceived competence in performing 20 dementia-related work activities and identify CE priorities among home care staff. - A cross-sectional survey of all home care staff in a primarily rural health region was used to gather data. Of 111 eligible staff, 82 participated (41 nursing aides, 41 nurses/case managers). To explore the relationship between activity frequency (F) and competence (C), the proportion of nurses and aides in four quadrants for each activity was examined: (1) low F-low C, (2) low F-high C, (3) high F-low C, and (4) high F-high C. Nurses/case managers were significantly more likely than aides to regularly perform 11 activities and to report high competence in 9 activities; aides were more likely to assist with two activities (personal care and daily living activities). Thus, nurses/case managers performed a broader range of activities and reported higher competence overall. - The top CE topic for both groups was recognizing differences between dementia subtypes, but rankings for most activities varied by group. Aides' CE priorities indicated a desire to develop competence in low frequency-low competence activities, suggesting an expanded role in supporting dementia patients and their families. Nurses' CE priority topics were in the high F-high C quadrant, indicating a need to further develop competence in these activities. Findings have implications for planning CE programming for home care providers. |
| Kosteniuk, J., Morgan, D., O’Connell, M., Dal Bello-Haas, V. & Stewart N. (2016) Focus on dementia care: Continuing education preferences, challenges, and catalysts among rural home care providers, Educational Gerontology, 42:9, 608-620, DOI: 10.1080/03601277.2016.1205404 | II- Research (Quant) | B- Dementia training in home care (Canada) | Theme 1- Advanced or Client Group Specific Training (dementia) | - Home care staff who provide housekeeping and personal care to individuals with dementia generally have lower levels of dementia care training compared with other health care providers. The study's purposes were to determine whether the professional role of home care staff in a predominantly rural region was associated with preferences for delivery formats of dementia-specific continuing education (CE) programs, and challenges and catalysts to attending CE on any topic. - In 2014, 82 of 111 eligible home care staff in one Saskatchewan health region completed a cross-sectional postal survey (73.9% response rate). The survey included 41 nurses/case managers (client care coordinators, assessors, and team managers) and 41 continuing care aides (home health aides). - Nurses/case managers and aides were equally likely to report moderate to high interest in locally delivered CE and low interest in Internet-based and computer-based CE. Compared with nurses/case managers, aides were more likely to report challenges to CE attendance due to CE not being a requirement of their position or relevant to their work. Low staffing levels were the top challenge regardless of professional role. Nurses/case managers and aides were equally likely to identify locally offered programs and paid time off as the top two catalysts of CE attendance. - The authors argue that given the growing number of individuals with dementia receiving home care services, the current study suggests that continuing education should be offered locally and included in rural staff’s paid time in order to encourage attendance. |
| Sung, H. C., Su, H. F., Lee, W. L., Yamakawa, M., & Wang, H. M. (2022). Effects of a dementia virtual reality-based training with peer support for home care workers: A cluster randomized controlled trial. *International journal of geriatric psychiatry*, *37*(9), 10.1002/gps.5799. https://doi.org/10.1002/gps.5799 | II- Research (Quant) | B- VR dementia training (Taiwan) | Theme 1- Advanced or Client Group Specific Training (dementia) | - Home care workers who are the first-line care workers for community-dwelling dementia patients often have limited dementia knowledge, skills, and empathy towards those with dementia. Research is sparse on dementia care training using virtual reality (VR) technology and support network for home care workers. - This cluster randomized controlled trial evaluated the effects of a dementia VR-based training with peer support on dementia knowledge, attitude, competence, and empathy of home care workers. Each home care worker team was used as the unit for randomization. Sixteen teams were randomly assigned to either VR group or non-VR control group There was a total of 124 participants completed the study, the VR group (n = 61) received a dementia VR-based training consisted of 3-month dementia care e-book modules, dementia VR-based activity and 1-h monthly face-to-face peer support group meetings. The non-VR control group (n = 63) only receive the 3-month dementia care e-book modules and 1-h monthly regular staff meetings with no VR activity. Outcome measures were assessed at three time points: baseline, the end of the 3-month intervention, and 1-month post intervention. - Generalized estimating equations results indicate that the improvement in dementia knowledge, attitudes, competence, and empathy over time is significant in the VR group compared to the non-VR control group. The effects remained significant 1 month after the end of the 3-month intervention. - The authors conclude that innovative and accessible dementia training using VR technology with peer support is a promising training approach to improve dementia knowledge, attitudes, competence, and empathy of home care workers. |
| Stargatt, J., Bhar, S., Petrovich, T., Bhowmik, J., Sykes, D., & Burns, K. (2021). The Effects of Virtual Reality-Based Education on Empathy and Understanding of the Physical Environment for Dementia Care Workers in Australia: A Controlled Study. *Journal of Alzheimer's disease : JAD*, *84*(3), 1247–1257. https://doi.org/10.3233/JAD-210723 | II- Research (Quant) | B- Dementia training for dementia care workers (Australia) | Theme 1- Advanced or Client Group Specific Training (dementia) | - There is support for the effectiveness of virtual reality (VR) technology in dementia education. However, it is not yet known if VR is a useful tool for improving empathy and understanding of dementia care environments among dementia care workers. - This study compared learning outcomes of VR versus non-VR (control) workshops for dementia care workers of different ages and English-speaking backgrounds. - Dementia care workers enrolled in workshops on dementia care principles. Once participants were enrolled, workshops were assigned at random to deliver non-VR or VR-based education. Participants (N = 114, 91.8%female, mean age = 46.4; SD = 13.2; n = 60VR condition, 54control condition) completed self-report measures of empathy towards people living with dementia, understanding of dementia care environments, dementia knowledge, and attitudes towards dementia at pre- and post-workshop. - Significant pre-post main effects were observed for empathy, understanding of dementia care environments, and attitudes. Interaction effects were not found; improvements in outcomes were similar between conditions. However, interaction effects were observed for subgroups. Empathy improved significantly more in the VR condition for older participants. Understanding of dementia care environments improved more in the VR condition for younger and non-English-speaking background participants. - Using VR may not augment teaching outcomes for all learners. VR may differentially assist leaners of different ages and English-speaking backgrounds. More research is needed to understand for which variables and for whom VR is a useful teaching tool. |
| Kane, L., Leighton, C., Limbrick, H., Kilinc, S., Ling, J., & Eberhardt, J. (2023). You clapped, you cheered, but did anybody hear? A mixed-methods systematic review of dementia homecare workers’ training and psychosocial needs. Home Health Care Services Quarterly, 42(4), 282-310. | III- Review of literature | A- Home care education and support (worldwide) | Theme 1- Advanced or Client Group Specific Training (dementia) | - The homecare sector's high turnover rate is linked to poor working conditions and a lack of person-centered practice. Limited research exists on the training and psychosocial needs of homecare workers caring for people living with dementia (PLWD). This systematic review explored these needs and identified 285 studies, of which seven studies met the inclusion criteria. - A narrative synthesis identified four themes: "training and education challenges and facilitators;" "social isolation and the importance of peer support;" "emotional attachments and distress experienced by homecare workers;" and "working with families and its emotional impact on homecare workers." This review highlights the unmet educational and psychosocial needs of homecare workers and the negative impacts these unmet needs have. - To improve person-centered practice in homecare, workers require dementia-specific training, and concurrent emotional and peer support, alongside support managing relationships with clients' families. Future research is required to implement an intervention to meet these needs. |
| Goh, A. M. Y., Gaffy, E., Hallam, B., & Dow, B. (2018). An update on dementia training programmes in home and community care. *Current opinion in psychiatry*, *31*(5), 417–423. https://doi.org/10.1097/YCO.0000000000000438 | III- Review of the literature | A- HSW dementia training (Australia) | Theme 1- Advanced or Client Group Specific Training (dementia) | - Argues that many people living with dementia (PLWD) wish to continue living at home. The quality of home care services directly influences their ability to stay at home, their quality of life, and can promote independence for PLWD, and reduce burden for the family carer. For high-quality, effective, person-centered community-based dementia care, a knowledgeable and empathetic workforce is crucial. - This article presents a review and summary of the literature investigating dementia training programmes for community home care professionals and care workers, referred to collectively as home care workers (HCWs). - The review found a significant lack of evidence-based, codesigned specialist dementia training programmes for HCWs that address the needs of all stakeholders in home care, and which formally measure outcomes. To enable PLWD to maintain living at home in the community, dementia friendly and knowledgeable HCWs are needed. This review highlights the need for more research into the design and evaluation of evidence-based dementia specialist training programmes for community care. |
| Yeh, J., Pond, B., Beld, M., Garcia, A., Mauricio, J., Mata-Pacheco, J., Eldridge, C., & Ross, L. (2023). Enhancing Dementia Knowledge and Self-Efficacy of In-Home Supportive Services Caregivers Through Online Training. *Journal of applied gerontology : the official journal of the Southern Gerontological Society*, *42*(4), 617–626. https://doi.org/10.1177/07334648221144023 | IV- Case study | B- Online dementia training (USA) | Theme 1- Advanced or Client Group Specific Training (dementia) | - California's In-Home Supportive Services (IHSS) program provides vital home care to low-income consumers, some of whom live with Alzheimer's disease or related dementias (ADRD). Yet, most IHSS caregivers receive little or no training in dementia care. - This article presents preliminary descriptive results of the IHSS + ADRD Training Project, a 10-week, competency-based, dementia training program [*TP], delivered online, for IHSS caregivers, in Alameda County. - Increase in dementia knowledge and self-efficacy at training completion supports the importance of continuing and expanding this work. |
| Goh, A. M., Doyle, C., Gaffy, E., Batchelor, F., Polacsek, M., Savvas, S., Malta, S., Ames, D., Winbolt, M., Panayiotou, A., Loi, S. M., Cooper, C., Livingston, G., Low, L. F., Fairhall, A., Burton, J., & Dow, B. (2022). Co-designing a dementia-specific education and training program for home care workers: The 'Promoting Independence Through Quality Dementia Care at Home' project. *Dementia (London, England)*, *21*(3), 899–917. https://doi.org/10.1177/14713012211065377 | IV- Case study of co-design of training programme | A- Older people, dementia (England) | Theme 1- Advanced or Client Group Specific Training (dementia) | - Describes the co-design framework used in the co-design of a dementia specialist training program for home care workers. The Promoting Independence Through quality dementia Care at Home program [*TP] is a successful example of co-design methodology used across multiple project stages and with various stakeholder groups, including people living with dementia, family carers, home care workers, managers and researchers. Co-design methods were tailored to each stage, purpose, and stakeholder group, and to facilitate the involvement of people living with dementia. - Findings provide unique insights into optimising input from co-design partners, including people living with dementia; the methodology, conditions and requirements for participants to co-design and implement ideas; and perspectives on the enablers and challenges of using co-design in this population. |
| Gregory S. (2018). Diabetes care in care home and residential settings. *British journal of community nursing*, *23*(10), 510–513. https://doi.org/10.12968/bjcn.2018.23.10.510 | IV- Case study | B- Diabetes training for carers (England) | Theme 1- Advanced or Client Group Specific Training (diabetes) | - An increasing number of people require insulin to manage their diabetes, many of them in supported environments such as residential care homes. Community nursing teams are likely to have a growing caseload of care home residents who require diabetes care, including insulin injections, and many unregistered practitioners are being asked to take on this role. If community nurse leads, matrons, frailty teams and pharmacy teams work together to provide training to staff in care homes (particularly unregistered practitioners), diabetes care can be improved. - Training suitable for groups and for people with different levels of understanding and supported by written resources. Courses are interactive to engage and motivate all learners, and methods of teaching include group work, visual teaching aids and games. Self-assessment around competencies, using a competency framework, not only demonstrates the development of staff but also highlights key areas of diabetes care. |
| Baik, D., Leung, P. B., Sterling, M. R., Russell, D., Jordan, L., Silva, A. F., & Masterson Creber, R. M. (2021). Eliciting the educational needs and priorities of home care workers on end-of-life care for patients with heart failure using nominal group technique. Palliative medicine, 35(5), 977–982. https://doi.org/10.1177/0269216321999963 | II- Research (Qual) | B- Home care worker training needs end of life care (USA) | Theme 1- Advanced or Client Group Specific Training (end of life care) | - Home care workers, as paid caregivers, assist with many aspects of home-based heart failure care. However, most home care workers do not receive systematic training on end-of-life care for heart failure patients. The aim of this study was to elicit the educational needs and priorities of home care workers caring for community- dwelling adults with heart failure at the end-of-life. - Nominal group technique involving a semi-quantitative structured group process and point rating system was used to designate the importance of priorities elicited from home care workers. Individual responses to the question, "If you have ever cared for a heart failure patient who was dying (or receiving end-of-life care on hospice), what are some of the challenges you faced?", were aggregated into categories using directed content analysis methods. - Forty-one home care workers were recruited from a non-profit training and education organization in New York City. - Individual responses to the question were aggregated into five categories: (1) how to cope and grieve; (2) assisting patients with behavior changes, (3) supporting patients to improve their quality of life, (4) assisting patients with physical symptom management, and (5) symptom recognition and assessment. - The findings confirm the need for the formal development and evaluation of an educational program for home care workers to improve the care of heart failure patients at the end-of-life. There is also a need for research on integrating home care workers into the interprofessional healthcare team to support optimal health outcomes for patients with heart failure. |
| Booi, L., Sixsmith, J., Chaudhury, H., O’Connor, D., Surr, C., Young, M., & Sixsmith, A. (2023). "I didn't know it was going to be like this.": unprepared for end-of-Life care, the experiences of care aides care in long-term care. *BMC palliative care*, *22*(1), 132. https://doi.org/10.1186/s12904-023-01244-y | II- Research (Qual) | B- Care aide training for EOL care in long-term care (Canada) | Theme 1- Advanced or Client Group Specific Training (end-of-life) | - Care aides provide up to 70-90% of the direct care for residents in long-term care (LTC) and thus hold great potential in improving residents' quality of life and end-of-life (EoL) care experiences. Although the scope and necessity of the care aide role is predicted to increase in the future, there is a lack of understanding around their perceptions and experiences of delivering EoL care in LTC settings. The aim of this study was to gain an understanding of the perspectives, experiences, and working conditions of care aides delivering end-of-life care in LTC in a rural setting, within a high-income country. - Data were collected over ten months of fieldwork at one long-term care home in western Canada; semi-structured interviews (70 h) with 31 care aides; and observation (170 h). Data were analysed using Reflexive Thematic Analysis. - Two themes were identified: (i) the emotional toll that delivering this care takes on the care aids and (ii) the need for healing and support among this workforce. Findings show that the vast majority of care aides reported feeling unprepared for the delivery of the complex care work required for good EoL care. Findings indicate that there are no adequate resources available for care aides' to support the mental and emotional aspects of their role in the delivery of EoL care in LTC. Participants shared unique stories of their own self-care traditions to support their grief- processing, and emotional healing. - The authors conclude that to facilitate the health and well-being of this essential workforce internationally, care aides need to have appropriate training and preparation for the complex care work required for good EoL care. It is essential that mechanisms in LTC become mandatory to support care aides' mental health and emotional well-being in this role. Implications for practice highlight the need for greater care and attention played on the part of the educational settings during their selection and acceptance process to train care aides to ensure they have previous experience and societal awareness of what care in LTC settings entails, especially regarding EoL experiences. |
| Tsui, E. K., Wang, W. Q., Franzosa, E., Gonzalez, T., Reckrey, J. M., Sterling, M. R., & Baron, S. (2020). Training to Reduce Home Care Aides' Work Stress Associated with Patient Death: A Scoping Review. *Journal of palliative medicine*, *23*(9), 1243–1249. https://doi.org/10.1089/jpm.2019.0441 | III- Review of literature | A- Home care workers End of Life training (USA) | Theme 1- Advanced or Client Group Specific Training (end-of-life) | - Explains that home care workers (HCWs)-including home health aides, personal care aides, and other direct care workers-provide functional and other essential support that allows older, disabled, and seriously ill people to live at home. As a growing number of patients are aging and dying at home, HCWs are increasingly providing care at the end of life (EOL). Although prior qualitative studies have shown that patient death is an impactful and challenging experience for HCWs, the majority of HCWs receive almost no training on EOL issues. - The goal of this scoping review was to identify intervention studies describing training of HCWs in EOL issues to map types of training and to assess the degree to which existing efforts address HCW health and well-being. - Of the 393 articles screened, 26 underwent full-text review and 6 met inclusion criteria. Only one article discussed training designed for and implemented with HCWs exclusively. Other training simultaneously targeted multiple kinds of workers. - Supporting HCWs in reducing their stress and improving their coping skills was substantially addressed in only one article, although HCWs' emotional needs were addressed less centrally in several others. The findings suggest that there is a paucity of EOL training interventions tailored specifically to the experiences and positioning of HCWs. The authors recommend that future intervention studies address the multiple facets of HCWs' stress related to patient death to improve EOL care in the home. |
| Sterling, M. R., Cho, J., Leung, P. B., Silva, A. F., Ringel, J., Wiggins, F., Herring, N., Powell, A., Toro, O., Lee, A., Loughman, J., Obodai, M., Poon, A., Goyal, P., Kern, L. M., & Safford, M. M. (2022). Development and Piloting of a Community-Partnered Heart Failure Training Course for Home Health Care Workers. *Circulation. Cardiovascular quality and outcomes*, *15*(11), e009150. https://doi.org/10.1161/CIRCOUTCOMES.122.009150 | II- Programme evaluation | C- Home care worker heart failure training  (USA) | Theme 1- Advanced or Client Group Specific Training (heart failure) | - Argues that despite their unique contributions to heart failure (HF) care, home healthcare workers (HHWs) have unmet educational needs and many lack HF caregiving self-efficacy. - A community-partnered approach was used to develop and pilot a HF training course for HHWs with the Training and Employment Fund, a benefit fund of the largest healthcare union in the United States. This led to the development of a 2-hour virtual HF training course that met HHWs’ job-specific needs. English and Spanish-speaking HHWs interested in HF training, with access to Zoom, were eligible to participate in the study. A mixed methods design with pre/post surveys and semi-structured interviews was used to evaluate the course: (a) feasibility, (b) acceptability, and (c) effectiveness (change in knowledge [Dutch Heart Failure Knowledge Scale range 0−15] and caregiving self-efficacy [HF Caregiver Self-efficacy Scale range 0−100]). - Of the 210 HHWs approached, 100 were eligible and agreed, and 70 enrolled. Of them, 53 (employed by 15 different home care agencies) participated. - Post-training data showed significant improvements in HF knowledge and HF caregiving self-efficacy. The greatest gains occurred among those with the lowest pre-training scores. Participants found the course engaging, technically feasible, and highly relevant to their scope of care. Scalability to the workforce at large could be achieved with a train-the-trainer model. |
| Leung, P. B., Silva, A. F., Cho, J., Kaur, H., Lee, A., Escamilla, Y., Wiggins, F., Safford, M. M., Kern, L. M., Shalev, A., & Sterling, M. R. (2022). Eliciting the educational priorities of home care workers caring for adults with heart failure. *Gerontology & geriatrics education*, *43*(2), 239–249. https://doi.org/10.1080/02701960.2020.1793760 | II- Research (Qual) | B- Training needs for heart failure (USA) | Theme 1- Advanced or Client Group Specific Training (heart failure) | - Although home care workers (HCWs) are increasingly caring for adults with heart failure (HF), many feel unprepared and lack HF training. To serve as the foundation for a future educational intervention, this study aimed to elicit HCWs' HF educational needs. - Working with the largest healthcare union in the US to recruit 41 HCWs employed by 17 home care agencies. Using the nominal group technique, the study asked HCWs to respond to three questions: When caring for an HF patient: (1) What information do you want? (2) What symptoms worry you? (3) What situations do you struggle with? Participants ranked their responses by priority. Data were consolidated by question. - For question 1, participants ranked HF signs and symptoms most highly, followed by HF treatment and medications. For question 2, chest pain was most worrisome, followed by neurologic changes and shortness of breath. For question 3, participants struggled with encouraging patients to follow a heart-specific diet. - HCWs expressed a need to learn more about signs and symptoms of HF and ways to assist patients with HF self-care. These findings can inform the development of an HF training program for HCWs that specifically addresses their expressed needs. |
| Sterling, M. R., Cho, J., Ringel, J. B., & Avgar, A. C. (2020). Heart Failure Training and Job Satisfaction: A Survey of Home Care Workers Caring for Adults with Heart Failure in New York City. *Ethnicity & disease*, *30*(4), 575–582. https://doi.org/10.18865/ed.30.4.575 | II- Research (Quant) | B- Home care workers heart failure training (USA) | Theme 1- Advanced or Client Group Specific Training (heart failure) | - Home care workers (HCWs), who include home health aides and personal care attendants, frequently care for adults with heart failure (HF). Despite substantial involvement in HF care, prior qualitative studies have found that HCWs lack training and confidence, which creates challenges for this workforce and potentially for patient care. - This study quantified the prevalence of HF training among HCWs and determined its association with job satisfaction. A cross-sectional survey of agency employed HCWs caring for HF patients across New York, NY from 2018-2019. HF training was assessed with, "Have you received prior HF training?" Job satisfaction was assessed with, "How satisfied are you with your job?" The association between HF training and job satisfaction was determined with robust Poisson regression. - 323 HCWs from 23 agencies participated; their median age was 50 years, 94% were women, 44% were non-Hispanic Black, 23% were Hispanic, 78% completed ≥ high school education, and 72% were foreign-born. They had been caregiving for a median of 8.5 years and 73% had cared for 1-5 HF patients. Two-thirds received none/a little HF training and 82% felt satisfied with their job. In a fully adjusted model, HCWs with some/a lot of HF training had 14% higher job satisfaction than those with none/a little HF training. - The majority of HCWs have not received HF training. HF training was associated with higher job satisfaction, suggesting that HF training programs may improve HCWs' experience caring for this patient population. |
| Sterling, M. R., Barbaranelli, C., Riegel, B., Stawnychy, M., Ringel, J. B., Cho, J., & Vellone, E. (2022). The Influence of Preparedness, Mutuality, and Self-efficacy on Home Care Workers' Contribution to Self-care in Heart Failure: A Structural Equation Modeling Analysis. *The Journal of cardiovascular nursing*, *37*(2), 146–157. https://doi.org/10.1097/JCN.0000000000000768 | II- Research (Quant) | B- Home care workers supporting self-care for heart failure (USA) | Theme 1- Advanced or Client Group Specific Training (heart failure) | - Home care workers (HCWs) are increasingly caring for patients with heart failure (HF). Previous studies have shown that they contribute to HF patients' care, but how their preparedness and their relationship with patients (mutuality) influence caregiving is unknown, as well as the role of HCWs' self-efficacy. - Guided by the Situation-Specific Theory of Caregiver Contribution to HF Self-Care, this study investigated the influence of HCWs' preparedness and mutuality on HCWs' contribution to HF self-care and the mediating effect of HCWs' self-efficacy in the process. A cross-sectional survey of HCWs who cared for patients with HF. The survey included the Caregiver Preparedness Scale, Mutuality Scale, Caregiver Contribution to Self-Care of HF Index, and Caregiver Self-Efficacy in Contributing to Self-Care Scale. Structural equation modeling and a mediation analysis. - A total of 317 HCWs employed by 22 unique home care agencies across New York, NY, completed the survey. They had a median age of 50 years, 94% were women, and 44% were non-Hispanic Black. - Results demonstrated that mutuality had a direct influence on HCW contribution to self-care and preparedness influenced their contribution to self-care, but only through the mediation of self-efficacy. Home care workers' preparedness, mutuality, and self-efficacy have important roles in influencing their contribution to HF self-care. As a workforce increasingly involved in the care of patients with HF, knowing the mechanisms underpinning HCWs' contribution to self-care may illuminate future interventions aimed at improving their contributions and HF patient outcomes. |
| Padula, M. S., D'Ambrosio, G. G., Tocci, M., D'Amico, R., Banchelli, F., Angeli, L., Scarpa, M., Capelli, O., Cricelli, C., & Boriani, G. (2019). Home care for heart failure: can caregiver education prevent hospital admissions? A randomized trial in primary care. Journal of cardiovascular medicine (Hagerstown, Md.), 20(1), 30–38. https://doi.org/10.2459/JCM.0000000000000722 | II- Research (Quant) | C- Caregiver education for heart failure management (Italy) | Theme 1- Advanced or Client Group Specific Training (heart failure) | - To assess the feasibility and effectiveness of a low-complexity, low-cost model of caregiver education in primary care, targeted to reduce hospitalizations of heart failure patients. - A cluster-randomized, controlled, open trial was proposed to general practitioners, who were invited to identify patients with heart failure, exclusively managed at home and continuously attended by a caregiver. Participating general practitioners were then randomized to: usual treatment; caregiver education (educational session for recognizing early symptoms/signs of heart failure, with recording in a diary of a series of patient parameters, including body weight, blood pressure, heart rate). The patients were observed at baseline and during a 12-month follow-up. - Three hundred and thirteen patients were enrolled (163 in the intervention, 150 in the usual care group), 63% women, mean age 85.3 ± 7.7 years. At the end of the 12-month follow-up, a trend towards a lower incidence of hospitalizations was observed in the intervention group (hazard ratio 0.73; 95% CI 0.53-1.01 P = 0.061). Subgroup analysis showed that for patients with persistent/permanent atrial fibrillation, age less than 90 years or Barthel score equal to or greater than 50 a significant lower hospital admission rate occurred in the intervention group (hazard ratio 0.63; 95% CI 0.39-0.99, hazard ratio 0.66; 95% CI 0.45-0.97; and hazard ratio 0.61; 95% CI 0.41-0.89; respectively). - Caregivers training for early recognition of symptoms/signs of worsening heart failure may be effective in reducing hospitalizations, although the benefit was evident only in specific patient subgroups (with persistent/permanent atrial fibrillation, age <90 years or Barthel score ≥ 50), with only a positive trend in the whole cohort. |
| Stawnychy, M. A., Ringel, J. B., Barbara Riegel, & Sterling, M. R. (2023). Better Preparation and Training Determine Home Care Workers' Self-Efficacy in Contributing to Heart Failure Self-Care. *Journal of applied gerontology : the official journal of the Southern Gerontological Society*, *42*(4), 651–659. https://doi.org/10.1177/07334648221113322 | II- Research (Quant) | B- HCW training for heart failure (USA) | Theme 1- Advanced or Client Group Specific Training (heart failure) | - Home care agencies and policy-makers can target caregiving preparation and HF training to improve HCWs' confidence in caring for adult Heart Failure patients. In this secondary analysis of survey data home, care workers with adequate self-efficacy had at least some prior HF training (55% vs. 17%) and greater job satisfaction (90% vs. 77%). - Significant determinants for adequate self-efficacy were employment length, preparation for caregiving, and HF training. |
| Alvarez, C., Ibe, C., Dietz, K., Carrero, N. D., Avornu, G., Turkson-Ocran, R. A., Bhattarai, J., Crews, D., Lipman, P. D., Cooper, L. A., & RICH LIFE Project Investigators (2022). Development and Implementation of a Combined Nurse Care Manager and Community Health Worker Training Curriculum to Address Hypertension Disparities. *The Journal of ambulatory care management*, *45*(3), 230–241. https://doi.org/10.1097/JAC.0000000000000422 | II- Programme evaluation | B- Hyper-tension control, teamwork (USA) | Theme 1- Advanced or Client Group Specific Training (hyper-tension) | - The use of nurse care managers (CMs) and community health workers (CHWs) has demonstrated effectiveness in supporting improved blood pressure management among racially, ethnically, and socioeconomically minoritized populations. - A partnership with a community advisory board (CAB) to develop a CM and CHW training curriculum [*TP] and team-based collaborative care intervention to address uncontrolled hypertension. The objective of this study was to train CMs and CHWs to implement patient-centered techniques and address social determinants of health related to hypertension control. In partnership with a CAB, the team developed and implemented a training curriculum for the CM/CHW collaborative care team. - The training improved CM and CHW confidence in their ability to address medical and nonmedical issues that contribute to uncontrolled hypertension in their patients; however, preexisting norms and beliefs among CMs and CHWs created challenges with teamwork. The training curriculum was feasible and well-received. Additionally, the CMs' and CHWs' reactions provided insights to improve future collaborative care training and teamwork. |
| Russell, D., Fong, M. C., Gao, O., Lowenstein, D., Haas, M., Wiggins, F., Brickner, C., & Franzosa, E. (2022). Formative Evaluation of a Workforce Investment Organization to Provide Scaled Training for Home Health Aides Serving Managed Long-Term Care Plan Clients in New York State. *Journal of applied gerontology : the official journal of the Southern Gerontological Society*, *41*(7), 1710–1721. https://doi.org/10.1177/07334648221084182 | II- Programme evaluation | B- HSW providing long-term care (USA) | Theme 1- Advanced or Client Group Specific Training (long-term care) | - As part of its Medicaid program restructuring, New York State funded 11 Workforce Investment Organizations (WIO) to support training initiatives for the long-term care workforce. Focusing on one WIO, this formative evaluation examined quality improvement training programs delivered to 11,163 Home Health Aides employed by home care agencies serving clients of Managed Long-Term Care plans. - Results are presented from a thematic analysis of qualitative interviews with organizational and program stakeholders examining contextual factors influencing program objectives, implementation, barriers and facilitators, and perceived outcomes. The findings suggest that WIO training programs were implemented during a period of shifting organizational strategies alongside value-based payment reforms and challenges to aide recruitment and retention. - Stakeholders appraised WIO training programs positively and valued program flexibility and facilitation of communication and collaboration between agencies and plans. However, delivery and implementation challenges existed, and industry-wide structural fragmentation led stakeholders to question the WIO's larger impact. |
| Stonehouse, D. (2013). Appraisal And Its Benefits For The Support Worker. British Journal Of Healthcare Assistants. 07. 246-249. 10.12968/bjha.2013.7.5.246. | V- Commentary | B- Appraisals for home care workers | Theme 1- Career Development and Progression Systems (appraisals) | - Within this article, the author examines what an appraisal is and how, if done correctly, it supports and develops quality care. Appraisals receive a negative press with both employees and managers. The reasons for this are explored before moving on to identify the purpose and benefits that following an appraisal process brings. The many different forms that appraisals can take are highlighted. What factors make up an appraisal are discussed, from reviewing past performance to the setting of realistic and yet challenging goals. The importance of preparation for the support worker prior to the appraisal process is highlighted as a key factor to success. |
| Luz, C., & Hanson, K. (2015). Training the personal and home care aide workforce: challenges and solutions. Home Health Care Management & Practice, 27(3), 150-153. | II-Research (Quant) | C- Training programme for personal home care aides (USA) | Theme 1- Career Development and Progression Systems (barriers and challenges to career progression) | - Personal care aides (PCAs) are critical to meeting the need for low-cost, high-quality care for frail older adults at home. Developing this workforce entails not only increasing its size but also ensuring that PCAs possess the skills necessary to deliver competent, safe, and respectful care. Yet, no federal PCA competencies or training requirements exist, and state requirements vary widely. - In 2010, a 77-hour PCA model training program [*TP] was developed as part of a national demonstration. However, a key finding of this study was that many enrollees faced serious socio-economic challenges that prevented them from graduating. This report details findings from a survey sent to all non-completers to ascertain reasons for attrition and improve program success. It offers recommendations for future program planners. |
| Donovan, P. & Haydon, A. (2020) From support worker to district nurse team manager: a narrative. British Journal of Healthcare Assistants. https://doi.org/10.12968/bjha.2020.14.3.110 | V- Commentary | C- Career journey from support worker to district nurse team manager (UK) | Theme 1- Career Development and Progression Systems (career planning tools and career journey stories and inspiration) | - As the general population ages, so does the nursing workforce. A recent Queen's Nursing Institute (QNI) report (2019) stated that 1 in 10 qualified district nurse positions have been lost in the past 10 years—without a trained workforce to replace those who retire. Workforce planning is central to ensure safe and effective care in the community setting. - This article aims to provide a narrative account of one student who started her career at the University of Bolton as a trainee assistant practitioner and over time achieved her present position as a district nurse team manager. The account includes her journey from support worker to assistant practitioner, studying at the University of Bolton as a trainee assistant practitioner on a ‘work, earn and learn’ programme. The account details the support she received from mentors in the trust, alongside her onward journey to registered nurse and specialist nurse practitioner qualifications. |
| Akaragian, S., Crooks, H., & Pieters, H. C. (2013). Perspectives of unlicensed assistive personnel on career development. *Journal of continuing education in nursing*, *44*(9), 415–423. https://doi.org/10.3928/00220124-20130716-48 | II- Research (Qual) | B- HSW career development preferences and licensure (USA) | Theme 1- Career Development and Progression Systems (licensure process) | - An equivalency program, Method 3 (qualification based on equivalent education and/or experience) is a viable but underused option for unlicensed assistive personnel (UAP) who pursue licensure. This study describes the perceptions of UAP on opportunities for career development. Eighteen UAP participated in three focus groups. - Thematic analysis identified three major themes: core driving forces, processes of career development, and anticipated and desirable outcomes. - Method 3 provides a realistic approach to help UAP persevere with career development. Collaboration with management and peers, encouragement, and effective communication contributed to the success of participants, despite obstacles and challenges. Camaraderie and flexible scheduling were critical elements in participants' pursuit of first licensure. Taking small steps was described as an effective approach for UAP to persevere with career development. Support for informal career development is essential. - Argues that nursing leaders should consider an equivalency approach to accommodate individual preferences and learning needs for career development. |
| Liao, L., Xiao, L. D., Chen, H., Wu, X. Y., Zhao, Y., Hu, M., Hu, H., Li, H., Yang, X., & Feng, H. (2020). Nursing home staff experiences of implementing mentorship programmes: A systematic review and qualitative meta-synthesis. *Journal of nursing management*, *28*(2), 188–198. https://doi.org/10.1111/jonm.12876 | III- Review of the literature | C- Implementation of mentorship for nursing home staff (China) | Theme 1- Career Development and Progression Systems (mentorship programmes) | - The aim of this study was to determine nursing home staff experiences in mentorship programmes, and staff perceptions of the enablers and barriers to implement mentorship programmes. Mentorship programmes are perceived as playing an important role in improving the quality of care in nursing homes. However, little is known about research evidence across the global about staff's experiences in the programmes. - A search for studies published from the earliest available date to April 2019 was undertaken. Two reviewers performed data extraction and an appraisal of eight studies using tools from the Joanna Briggs Institute. A pragmatic meta-aggregative approach was applied to synthesise the findings. The qualitative research that was included was analysed to identify 63 findings that were organised into 12 categories and combined into three syntheses. - The implementation of effective mentorship programmes is influenced by three factors: mentor capability, opportunity in the mentorship programmes, and motivation in the mentorship programmes. There are a number of studies of nursing home staff experiences of mentorship programmes. However, systematic reviews that synthesise findings in this field are lacking. It is crucial to tailor the programme design to suit each unique nursing home care setting. Barriers and enablers have been identified, and no barriers are insurmountable. - The study findings will inform nurse managers of an ideal environment for the implementation of a successful mentorship programme. Nursing homes need to establish and sustain mentorship programmes to help improve workforce capacity in delivering high-quality care for residents. |
| Kaldy. J. (2023) Building the Way to Advancement with Career Pathways. Provider Magazine https://www.providermagazine.com/Issues/2023/Fall/Pages/Building-the-Way-to-Advancement-with-Career-Pathways.aspx | V- Commentary | B- Developing career pathways in health care roles (USA) | Theme 1- Career Development and Progression Systems (organisational approach to career progression and development strategies) | - Providers are crafting and supporting career pathways as a way to show staff that advancement and retention are important parts of their growth. Qualified medication aides, apprenticeship programs, and mentorship can be parts of those pathways. - To become a Certified Medication Aide (CMA) a person has to work as a CNA for a year, demonstrate clinical competencies, and complete 160 hours of training. The role not only comes with new responsibilities, but the medication aide also gets a salary bump. |
| Blau, G., Chapman, S.A., & Neri, M.T. (2016). Testing the Relationship Between Personal/Home Care Aide Trainees’ Career Goals and Their Commitment to Home Care. Home Health Care Management & Practice, 28, 150 - 154. | II-Research (Quant) | C- Training programme for personal home care aides (USA) | Theme 1- Career Development and Progression Systems (personal career aspirations, goals, preferences, objectives, home care intent) | - Using a pre-training and post-training survey research design, 683 participants completed a personal/home care aide (P/HCA) training program [*TP]. After training, participants chose a career goal and were placed in one of the following groups: (1) only goal to be a P/HCA, (2) become a certified nurse aide/assistant or home health aide, (3) become a licensed vocational nurse, or (4) go to college to earn a degree in a health-related area. - Factor analysis of home care commitment items indicated two measures: home care intent and stepping stone. Group 2 trainees had a significantly higher home care intent than trainees in Groups 1 and 4. However, Groups 2, 3, and 4 were each more likely to view P/HCA training as a “stepping stone to higher level jobs” than Group 1. |
| Burger, S., et al. (2018) Picker Institute Europe. Exploring education and training in relation to older people's health and social care. pp. 118. Oxford, UK. https://dunhillmedical.org.uk/wp-content/uploads/2021/08/18-08P1.pdf | II- Research (Mixed method) | B- Education and training of older care workforce (England) | Theme 1- Career Development and Progression Systems (supportive work environment) | - This research, commissioned by Dunhill Medical Trust, evaluates the education and training for the workforce providing care for older people in the UK and looks at the views of health and social care staff on the training they have received. - The research involved a knowledge audit involving desk research and telephone interviews with eight stakeholders; qualitative telephone depth interviews with 41 health and social care staff; and a systematic review which analysed the results of 36 studies. - The findings cover: the impact of education and training on care; what training should include; barriers to learning; initiatives and best practice models that currently exist; and barriers to provide quality care to older people. - The results found a need for tailored training, more continuing professional development, and for more recognition and incentives for those choosing a career in health and social care for older people. - Other findings included that existing research is scarce and its results are inconclusive; and the amount and quality of education and training appears to be varied and inconsistent across geographies and professions. - The research also found that whilst education and training are important, it found that continuing professional development, access to knowledge and information and a positive and supportive culture were more so. |
| Fitzpatrick, J. M., Bianchi, L. A., Hayes, N., Da Silva, T., & Harris, R. (2023). Professional development and career planning for nurses working in care homes for older people: A scoping review. International Journal of Older People Nursing, 18, e12519. | III- Review of literature | C- Career planning and development of nurses in care homes (UK) | Theme 1- Career Development and Progression Systems (supportive working environment) | - A skilled, knowledgeable, and compassionate nurse workforce is pivotal to caring well for older people living in care homes. This requires the provision of continuing professional development and career planning for nurses, which are key also for nurse recruitment and retention. Continuing professional development and career planning strategies and interventions should be evidence-driven. - The objective of this review was to identify the extent, range and nature of contemporary evidence regarding professional development and career planning for nurses caring for older people living in care homes. The methodological framework used was the Joanna Briggs Institute guidance for scoping reviews. The PRISMA extension for scoping reviews was used as the reporting framework. Four databases were searched from January 2010 to July 2021. Results were screened independently by two reviewers using eligibility criteria. Full texts and the reference lists of eligible articles were reviewed. Data were extracted for key elements from the 25 articles included. - Of the 25 articles, the majority were authored in the United States (n = 10) and UK (n = 8) with the remaining from Australia (n = 3), Canada (n = 3) and the Netherlands (n = 1). Four articles reported on professional development programmes. Three literature reviews addressed challenges for nurse participation in professional development, experiences of care home nurses as clinical leaders and managers, and leadership. Two expert commentaries reported on the challenges related to professional development and career planning for care home nurses. - Sixteen empirical studies investigated a range of topics including: competencies, roles, intention to stay and leave, continuing professional development, and leadership. Key emergent factors that support professional development and career planning were as follows: access to structured learning opportunities addressing knowledge and skills specific to nursing frail older adults, a supportive working environment including adequate staffing, study time and flexible working, and integration of leadership development. - This scoping review has highlighted factors that support and challenge professional development and career planning for nurses working in the care home sector. There remain important gaps in the opportunities for professional development and career planning for care home nurses that warrant attention. |
| Meyer-Kühling, I., Wendelstein, B., Pantel, J., Specht-Leible, N., Zenthöfer, A., & Schröder, J. (2015). A contribution to improve communication between professional caregivers and physicians]. *Pflege*, *28*(5), 277–285. https://doi.org/10.1024/1012-5302/a000447 | II- Programme evaluation | B- Communication training for nursing home paid carers (Germany) | Theme 1- Core Competencies Training for Home Care Work (communication with clinicians/healthcare professionals) | - Failures of communication between professional caregivers and physicians affect the quality of supply of nursing home residents. As part of a model project this study aimed to develop training for caregivers to improve communication and promote cooperation with physicians. - For the needs assessment as a basis to develop the training 56 professional caregivers and 40 physicians engaged in nursing home care answered questionnaires regarding their cooperation. Based on these results a module for communication between professional caregivers and physicians was developed and adapted the TANDEM [*TP] communication training for caregivers by Haberstroh and Pantel (2011). 25 professional caregivers in leading positions have been trained as multipliers in order to provide their colleagues the communication training with the additional element (TANDEMplus). TANDEMplus was evaluated in forms of reflection rounds and feedback questionnaires. - 254 professional caregivers, housekeeping staff and daytime companions participated in a complete TANDEMplus training by the multipliers until July 2014. The implementation of their developed communication strategies into practice was experienced positively by the participants. The module “communication with physicians” is relevant for professional caregivers to raise awareness of their own competence and facilitate a structured information exchange. The training of multipliers was executed in order to ensure transfer effects and sustainability. |
| Filmer, T., & Herbig, B. (2020). A training intervention for home care nurses in cross-cultural communication: An evaluation study of changes in attitudes, knowledge and behaviour. *Journal of advanced nursing*, *76*(1), 147–162. https://doi.org/10.1111/jan.14133 | II- Research (Mixed method) | C- Cross-cultural communication training for home care nurses (China) | Theme 1- Core Competencies Training for Home Care Work (communication with clients, cross-cultural communication) | - To assess whether a training intervention in cross-cultural communication can positively impact attitudes, knowledge and behaviour and to investigate possible dependencies between these components. The study was a controlled longitudinal multimethod evaluation. - Training based on theoretical considerations and informed by semi-standardized interviews with home care nurses was developed and evaluated. Participants rated their cross-cultural attitudes, knowledge and behaviour and answered case vignettes assessing their knowledge before and after this training. Shift observations assessed behavioral aspects at t1 and t2. Data were collected between June 2016-March 2017 and between April 2017-November 2017. Analyses of variance and multiple linear regression models were employed. - The training showed promising tendencies with cross-cultural attitudes, knowledge and behaviour with diverging results for initially quite high self-reports showing positive but mostly not significant developments and objective assessments mostly showing significant positive changes. There were significant associations between self-reported cross-cultural behaviour at t1 and objective cross-cultural knowledge at t2, whereas self-rated and objectively assessed knowledge showed no significant associations. Shift observations showed significant positive developments in participants' communication behaviour. - The study shows the importance of using different methods and targeting different outcomes areas to rate impacts of (cross-cultural) training interventions. Future studies should consider challenging conditions in home care nursing affecting the success of interventions and investigate mechanisms of skill acquisition in nursing. This is one of very few studies using multi-method approach to evaluate a cross-cultural competency intervention and simultaneously assess cross-cultural attitudes, knowledge and behaviour including possible dependencies between these aspects. |
| Palesy, D., & Jakimowicz, S. (2019). Health literacy training for Australian home care workers: Enablers and barriers. *Home health care services quarterly*, *38*(2), 80–95. https://doi.org/10.1080/01621424.2019.1604458 | II- Research (Qual) | B- Home support worker health literacy training (Australia) | Theme 1- Core Competencies Training for Home Care Work (health literacy) | - The rapidly expanding Australian home care workforce represents an untapped resource for improving health literacy (HL) and health outcomes of their clients. - Nine home care workers (HCWs) were interviewed for this study to gain data around their experiences of providing HL support to their clients, key HL needs and priorities, and training that would best these needs. - Findings indicate that HCWs are providing HL support and identify a number of enablers and barriers to providing this support. Core inclusions for a health literacy training checklist are suggested. |
| Palesy, D., & Jakimowicz, S. (2020). Health literacy support for Australian home-based care recipients: A role for homecare workers? Home health care services quarterly, 39(1), 17–32. https://doi.org/10.1080/01621424.2019.1691698 | II- Research (Qual) | B- Home care worker health literacy (Australia) | Theme 1- Core Competencies Training for Home Care Work (health literacy training) | - Clear links have been established between low health literacy (HL) levels and poor health outcomes. One means of improvement may be found in the rapidly growing paid home care workforce, whose direct and frequent contact with aged/disabled care recipients positions them to provide HL support. - This study examines Australian homecare worker (HCW) experiences in HL when providing assistance to their care recipients. A self-reported cross-sectional survey collected data from 75 HCWs. They reported concerns about their clients' HL yet were cautious about providing support in this area. HL levels of the HCWs themselves were unconvincing, and the majority requested targeted education and training. Further research is needed into HL levels of both HCWs and care recipients, client demographics, the types of HL support being requested of HCWs, a more detailed scoping of the HCW role, and the curriculum and pedagogies which may comprise a HL education and training program for HCWs. |
| Smith, R., & Wright, T. (2021). Older lesbian, gay, bisexual, transgender, queer and intersex peoples’ experiences and perceptions of receiving home care services in the community: A systematic review. *International Journal of Nursing Studies*, *118*, 103907. | I- Systematic review | B- LGBTQI+ awareness training (worldwide) | Theme 1- Core Competencies Training for Home Care Work (LGBTQI+ awareness) | - Numbers of older lesbian, gay, bisexual, transgender, queer and intersex (LGBTQI+) people are increasing worldwide in line with the ageing populations of many countries. Most LGBTQI+ people want to remain in their own homes as they age, making it important to understand their experiences and perceptions of receiving home care. - This systematic review aimed to examine older (over 60 years) LGBTQI+ people's perceptions and experiences of using formal home care services in the community. The following six electronic databases were searched from the date of their first records until the first week of March 2020: MEDLINE; PsycINFO; Social Policy and Practice; CINAHL; SSCI; and ASSIA. Hand searches of the reference lists of the included studies and relevant reviews were also conducted. Only peer reviewed research published in English was included. There were no restrictions on study design. Findings were analysed using narrative synthesis. - Seven studies involving 169 participants were included in the synthesis. All were qualitative. Most participants were either lesbian women or gay men, with no studies investigating home care for transgender, queer, intersex or other sexual minorities. Fear of accessing home care services due to the perceived threat of homophobia and past negative experiences of discrimination were common. Some concealed any LGBTQI+ materials in their homes to try and hide their sexuality from home care workers. Despite fear of discrimination, lesbian women and gay men reported wanting and expecting the same level of care, dignity and respect as their heterosexual counterparts. Mandatory LGBTQI+ sensitivity training for home care workers was identified for reducing homophobia and increasing the inclusivity of service providers. - Older lesbian women and gay men fear or experience discrimination from home care workers, with some choosing to hide their sexuality causing stress and anxiety. Sensitivity training in the needs of older LGBTQI+ people should be considered by home care service providers as a way of reducing homophobic attitudes which may exist among some home care workers. Due to the paucity of studies and their focus on older lesbian women and gay men, more research is needed to explore the experiences of other sexual minorities receiving home care services who are represented by the LGBTQI+ umbrella term. |
| Daley, A. and MacDonnell, J.A. (2015), ‘That would have been beneficial’: LGBTQ education for home-care service providers. Health Soc Care Community, 23: 282-291. https://doi-org.proxy.library.rcsi.ie/10.1111/hsc.12141 | II- Research (Qual) | B- Diversity training for home healthcare providers (Canada) | Theme 1- Core Competencies Training for Home Care Work (LGBTQI+ awareness) | - This paper reports qualitative findings from a pilot study that explored the lesbian, gay, bisexual, transgender and queer (LGBTQ) education needs of home-care service providers working in one large, urban Canadian city. The pilot study builds upon research that has documented barriers to health services for diversely situated LGBTQ people, which function to limit access to good-quality healthcare. LGBTQ activists, organisations and allies have underscored the need for health provider education related to the unique health and service experiences of sexual and gender minority communities. However, the home-care sector is generally overlooked in this important body of research literature. - This study used purposeful convenience sampling to conduct four focus groups and two individual interviews with a total of 15 professionally diverse home-care service providers. Data collection was carried out from January 2011 to July 2012 and data were analysed using grounded theory methods. - The identification of the overarching theme, ‘provider education’ and it had two sub-themes: (i) experiences of LGBTQ education; and (ii) recommendations for LGBTQ education. The study findings raise important questions about limited and uneven access to adequate LGBTQ education for home-care service providers, suggest important policy implications for the education and health sectors, and point to the need for anti-oppression principles in the development of education initiatives. |
| Hoge, M. A., McFaul, M., Cauble, L. L., Craft, K. L., Paris, M., Jr., & Calcote, R. M. (2016). Building the skills of direct care workers: The Alaskan core competencies initiative. Journal of Rural Mental Health, 40(1), 31–39. https://doi.org/10.1037/rmh0000045 | II- Programme evaluation | C- Home care worker mental health awareness training (USA) | Theme 1- Core Competencies Training for Home Care Work (mental health awareness) | - A large proportion of the health and social service workforce is comprised of direct care workers who have no formal preservice education and receive a limited amount of on-the-job training. These workers are essential in all geographic areas and are especially critical in rural and frontier regions where access to advanced health care professionals is limited. - Driven by stakeholder demand, the State of Alaska launched the multiyear Alaskan Core Competencies initiative to strengthen the training of its direct care workforce. - This article details the development of a set of cross-sector core competencies relevant to workers in the fields of mental health, addictions, developmental and physical disabilities, and the long-term care of older adults. Also described are the related assessment tools, curriculum, and train-the-trainer learning communities, which were developed to enable the dissemination of the competencies. The authors conclude by discussing the growing interest nationally in competencies for this workforce, the challenges of adapting one set of competencies for varied jobs in diverse health and social service sectors, and the financial barriers to widespread adoption of competency-based worker training. (PsycINFO Database Record (c) 2016 APA, all rights reserved) |
| Hsu, W. C., Hsieh, Y. P., & Lan, S. J. (2021). Home care aides' attitudes to training on oral health care. *PloS one*, *16*(4), e0249021. https://doi.org/10.1371/journal.pone.0249021 | II- Research (Quant) | B- Home care aides training in oral health care (Taiwan) | Theme 1- Core Competencies Training for Home Care Work (oral health care) | - This study investigated home care aides' (HCAs) oral health care experience, knowledge, and their intention to receive professional training, to explain and predict factors of their intention to receive such training. This cross-sectional study collected data through a structured questionnaire. HCAs affiliated with home care agencies in Taichung, Taiwan were recruited through purposive sampling. - A total of 487 questionnaires were distributed from September to December 2015 with 280 valid responses collected (57.4%). This study predicted the factors of HCAs' intention to receive oral health care training through a decision tree analysis. The decision tree model classified the respondents with an accuracy of 77.5%. The optimal predictor variable was oral health care knowledge. Among the low-scoring respondents on oral health care knowledge, 76.4% were classified in the "uninterested" group, whereas 84.8% of the high scorers were classified in the "interested" group. The second-best predictor variable was whether oral health care is part of the job responsibility. Among those who answered Yes, 92.9% were in the interested group, as were 76.5% of those who answered No. - It is recommended to add "disease and oral care-related content" and "safety protection, assessment, and usage of oral care tools during practical oral care process" to the oral healthcare training course content for HCAs to improve HCAs' oral healthcare knowledge and oral care skills. These research findings are valuable and may be considered in the future development of the in-service educational training of oral healthcare for HCAs. |
| Edman, K., & Wårdh, I. (2022). Oral health care beliefs among care personnel working with older people - follow-up of oral care education provided by dental hygienists. *International journal of dental hygiene*, *20*(2), 241–248. https://doi.org/10.1111/idh.12588 | II- Research (Quant) | B- Oral care in home care (Sweden) | Theme 1- Core Competencies Training for Home Care Work (oral health care) | - The proportion of older people in the population is increasing rapidly. Along with this comes an increase in the number of people requiring assistance in daily living, including oral care. Swedish law stipulates that care personnel who work with older people should be offered oral health education every year. The aim of this study was to investigate oral health care beliefs among such personnel. - A questionnaire study was conducted among 2167 personnel providing care to older people at special accommodation sites and in home care. Data were collected using the Nursing Dental Coping Beliefs Scale. Descriptive statistics were calculated, and logistic regression analysis was performed. - Personnel working in home care had lower odds of having an internal locus of control than those working in special accommodation, and personnel with less than 10 years of working experience had lower odds than their more experienced counterparts. Men had higher odds of having an external locus of control than women. It seems important to ensure that home care personnel and less experienced personnel attend oral care educational sessions, and to encourage male staff to focus on oral care work. |
| Kelly, C. M., Morgan, J. C., & Jason, K. J. (2013). Home care workers: interstate differences in training requirements and their implications for quality. *Journal of applied gerontology : the official journal of the Southern Gerontological Society*, *32*(7), 804–832. https://doi.org/10.1177/0733464812437371 | III- Review of state policies | B- HSWs differences in state law and impact on quality (USA) | Theme 1- Core Competencies Training for Home Care Work (personal care, personal assistance) | - Home care workers are the fastest growing segment of the US direct care workforce who provide nonmedical services that are not reimbursed by Medicare; consequently, requirements for training and supervision are left to the states. The purpose of this study was to compare state training requirements and to identify core competencies for home care workers. Content analysis of relevant state laws determined that 29 states require a license for home care providers. Of these 29 states, 26 require orientation and 15 require in-service training for home care workers; the duration and content of these programs vary widely across the states. Fifteen states require on-site supervision of home care workers. - Argues that in addition to current state training requirements (e.g., activities of daily living (ADLs) and instrumental activities of daily living (IADL) assistance; infection control), other core competencies (e.g., basic medication information; behavioural management) should also be mandatory. More frequent on-site supervision is also necessary to improve home care quality. |
| Kajander-Unkuri, S., Kämäräinen, P., Hartikainen, T., & Turjamaa, R. (2022). Effectiveness of a combined web-based and simulation-based continuing education on home-care professionals' competence to evaluate older people's acute care needs in Finland. *Health & social care in the community*, *30*(5), e1765–e1774. https://doi.org/10.1111/hsc.13605 | II- Research (Quant) | B- Home care training in acute health conditions (Finland) | Theme 1- Core Competencies Training for Home Care Work (recognising medical emergencies and acute care needs) | - Home-care professionals need competencies to ensure that they evaluate their older peoples' health conditions, especially in acute care situations. - This study aimed to investigate the effectiveness of combined web-based and simulation-based continuing education on home-care professionals' competence regarding evaluating older people's needs for acute care. A quasi-experimental pre-test-post-test study was conducted with home-care professionals who were working in older people's care in Finland. Home-care professionals (N = 254) had participated in combined web-based and simulation-based continuing education in 2017-2019. Data were collected using a questionnaire developed for this study before (n = 171) and after (n = 83) the education. The data were analysed statistically. - The mean competence score was 3.22 ± 0.51 before the education and 3.92 ± 0.57 after the education. Improvements were detected in overall competence and in all eight subscales. Improvements were highest in health assessment and consultation and the lowest in type 2 diabetes, but this was already the highest level of self-assessed competence in the pre-test assessment. The combined web-based and simulation-based continuing education was effective in increasing the home-care professionals' competence in evaluating older people's needs for acute care. Evaluating older people's needs for acute care should be embedded in curricula and continuing education programmes. |
| Walsh, W., Meyer, C., & Cyarto, E. V. (2023). Home care worker-supported exercise program to address falls: a feasibility study. *Australian journal of primary health*, 10.1071/PY22248. Advance online publication. https://doi.org/10.1071/PY22248 | II- Programme evaluation | C- Falls prevention (Canada) | Theme 1- Core Competencies Training for Home Care Work (Safety in the home, falls prevention) | - Falls are a major concern for community-dwelling older adults. The Otago Exercise Program (OEP) is an evidence-based home program that reduces the risk of falls. Exercise participation and program adherence can be challenging. Home care workers (HCWs) are well positioned to provide support for older adults. - This feasibility study included: HCW training; HCW in-home support of a physiotherapist tailored OEP; online physiotherapy consultations; older participant questionnaires and functional outcome measures; and HCW and older participant interviews. - Twelve older adults, eight HCWs and one physiotherapist participated. A small falls risk reduction, and improvement in falls efficacy, quality of life and functional improvement were noted. Thematic analysis showed formal and informal support was valued by older adults and HCWs. A role-ordered matrix synthesis highlighted variable ongoing independent program participation. - By Your Side, a physiotherapist-led and home care worker-supported modified OEP provides a feasible and acceptable option for falls prevention in home care services. Collaborative teamwork, and both formal and informal support, are key aspects to optimising engagement and benefits. |
| Luz, C. C., Hanson, K. V., Hao, Y., & Spurgeon, E. (2018). Improving Patient Experiences and Outcomes Through Personal Care Aide Training. *Journal of patient experience*, *5*(1), 56–62. https://doi.org/10.1177/2374373517724349 | II- Programme evaluation | B- Effects of personal care aide training (USA) | Theme 1- Cost-Benefits of Employment and Training (cost-benefits of training) | - The rapidly aging US population is resulting in major challenges including delivering quality care at lower costs in the face of a critical health-care workforce shortage. The movement toward home care has dramatically increased the need for qualified, paid personal care aides (PCAs). Adequate PCA training that focuses on skills for person-centered, at home support is an imperative. - This study provides evidence that clients of PCAs who have completed a comprehensive, evidence based PCA training program, titled Building Training…Building Quality (BTBQ) [*TP], report higher satisfaction and better health outcomes, compared to clients of PCAs with lesser or other training. - A mixed-methods, quasi-experimental design was used to compare self-reported survey responses from clients of BTBQ-trained PCAs (treatment group) with responses from clients of non-BTBQ-trained PCAs (control group). Clients of BTBQ-trained PCAs had significantly fewer falls and emergency department visits compared to clients whose PCAs had no BTBQ training (P < .05). Conclusion: BTBQ-like PCA training reduces costly adverse events. |
| Ayalon, L., & Shinan-Altman, S. (2021). Tension between reality and visions: Lessons from an evaluation of a training program of paid elder care workers. *Health & social care in the community*, *29*(6), 1915–1924. https://doi.org/10.1111/hsc.13305 | II- Programme evaluation | B- Training programme for elder care workers (Israel) | Theme 1- Cost-Benefits of Employment and Training (cost-benefits of training) | - The present study is based on a 3-year evaluation of an Israeli training program for local paid elder care workers, called, 'community care'. Interviews were conducted with all stakeholders involved in the program, including program developers, facilitators, funders, trainees, dropouts, graduates, employers and older care recipients. - Qualitative thematic analysis was used. Analysis was supplemented by quantitative data concerning the program's inputs, outputs, and outcomes. - The program had multiple strengths, including a substantial funding stream and a highly skilled and committed team. Yet, out of 130 participants, 94 completed the program and 31 worked as care workers afterwards. - Three main challenges to the efficacy of the training program were identified. A first challenge stems from the gap between the program's vision and real-life requirements and constraints. The second challenge concerns a disagreement between stakeholders concerning the definition of the new community care profession as an opportunity to empower trainees and encourage personal growth versus the community care worker as being no different from the traditional direct paid carer. A third challenge concerns the program's lack of integration between personal/physical care on the one hand and emotional and psychological care, on the other hand. The findings stress the importance of adequately conducting a needs assessment prior to embarking on a new social program and the tension between an ideal prototype and real-life constraints. The findings also stress the necessity of top-down processes, supported by the government to the development of a new profession of community elder care. |
| Luz C, Hanson K. (2015) Filling the Care Gap: Personal Home Care Worker Training Improves Job Skills, Status, and Satisfaction. Home Health Care Management & Practice. 2015;27(4):230-237. doi:10.1177/1084822315584316 | II- Programme evaluation | B- Training programme (USA) | Theme 1- Cost-Benefits of Employment and Training (cost-benefits of training) | - With an aging population and provider shortages, personal care aides (PCAs) hold potential for providing low-cost, high-quality in-home supports and services. They comprise an unprecedented workforce in terms of size and rapid growth. However, this workforce is also characterized by costly high-turnover rates that threaten quality of care and outcomes. It is imperative that measures be taken to improve PCA skills and stabilize their employment. - In 2010, a PCA training program was developed titled “Building Training . . . Building Quality” as part of a national demonstration. - Key findings were that learners’ skills, employability, and job satisfaction significantly improved, and “intent to stay” was associated with increased confidence in ability to do the job. |
| Kemeny, M. E., & Mabry, J. B. (2017). Making meaningful improvements to direct care worker training through informed policy: Understanding how care setting structure and culture matter. *Gerontology & geriatrics education*, *38*(3), 295–312. https://doi.org/10.1080/02701960.2015.1103652 | II- Research (Mixed method) | B- Learning and working environments in long term care (USA) | Theme 1- Cost-Benefits of Employment and Training (cost-benefits of training) | - A well-intentioned policy governing the training of direct care workers (DCWs) who serve older persons, in practice, may become merely a compliance issue for organizations rather than a meaningful way to improve quality of care. - This study investigates the relationships between best practices in DCW training and the structure and culture of long-term support service (LTSS) organizations. Using a mixed-methods approach to analyzing data from 328 licensed LTSS organizations in Pennsylvania. - The findings suggest that public policy should address methods of training, not just content, and consider organizational variations in size, training evaluation practices, DCW integration, and DCW input into care planning. Effective training also incorporates support for organizations and supervisors as key aspects of DCWs' learning and working environment. |
| Fong, M. C., Russell, D., Brickner, C., Gao, O., Vito, S., & McDonald, M. (2022). Medicaid long-term care workforce training intervention and value-based payment metrics. *Health services research*, *57*(2), 340–350. https://doi.org/10.1111/1475-6773.13930 | II- Research (Quant) | B- Workforce training in long-term care (USA) | Theme 1- Cost-Benefits of Employment and Training (cost-benefits of training) | - This study aimed to examine the impact of a scaled implementation of workforce training intervention on value-based payment measures in a large home-based Medicaid managed long-term care plan population in New York. - Study data was managed long-term care clients' health assessments from the Uniform Assessment System of New York merged with paid claims, home health aide operational visit data, and workforce training rosters between 2018 and early-2020. A quasi-experimental design was used. Exposure and control groups were constructed using the proportion of service hours delivered by trained aides between clients' baseline and follow-up/outcome assessments. Multivariate logistic generalized linear and additive models were estimated to examine associations between exposure to trained aides and value-based payment measures. The analytic sample consisted of 19,212 pairs of assessments from 13,320 long-term care clients continuously enrolled in the plan between baseline and follow-up/outcome assessments. Matched assessment pairs were 6-10 months apart. - Over 27% of the study population (n = 3656 clients) received services from one or more of 8683 trained aides. Statistically significant associations were observed for four of seven value-based payment measures; however, the presence and magnitudes of positive training effects differed by client service needs. With covariate adjustment, workforce training had the largest estimated positive impacts on rates of flu vaccination among average-need clients (1.60%,), not experiencing uncontrolled pain among above-average-need clients (0.69%), stable/improved pain intensity among heavy-need clients (1.25%,), and stable/improved shortness of breath among light-need clients (0.88%, SE = 0.003). - Although the study shows mixed associations between scaled workforce training implementation and value-based payment metrics, it shows that workforce training could benefit high-need long-term care recipients. Health indicators more sensitive to the daily support provided by direct care workers should be integrated into value-based health care models. |
| Bradley, P. (2015) How to do the Care Certificate Standards in 10 hours, for £36. British Journal of Healthcare Assistants, 9, 11, Health Matters. https://doi.org/10.12968/bjha.2015.9.11.530 | V- Commentary | C- Assessment of Care Certificate training (England) | Theme 1- Cost-benefits of Employment and Training (cost-Benefits of training or certification) | - Describes the process of training and attaining a Care Certificate entirely online. Argues that it is not acceptable to be assessed online and not in the work environment. The commercialisation of certification processes should not risk quality. |
| Snyder, C. R., Dahal, A., & Frogner, B. K. (2018). Occupational mobility among individuals in entry-level healthcare jobs in the USA. *Journal of advanced nursing*, *74*(7), 1628–1638. https://doi.org/10.1111/jan.13577 | II- Research (Quant) | C- Job movement from entry level healthcare occupations to personal care/services occupations (USA) | Theme 1- Cost-Benefits of Employment and Training (occupational mobility) | - The aim of this study was to explore career transitions among individuals in select entry-level healthcare occupations. Entry-level healthcare occupations are among the fastest growing occupations in the USA. Public perception is that the healthcare industry provides an opportunity for upward career mobility given the low education requirements to enter many healthcare occupations. The assumption that entry-level healthcare occupations, such as nursing assistant, lead to higher-skilled occupations, such as Registered Nurse, is under-explored. - The researchers analysed data from the Panel Study of Income Dynamics, which is a nationally representative and publicly available longitudinal survey of US households. Using longitudinal survey data, they examined the job transitions and associated characteristics among individuals in five entry-level occupations at the aide/assistant level over 10 years timeline (2003-2013) to determine whether they stayed in health care and/or moved up in occupational level over time. - This study found limited evidence of career progression in health care in that only a few of the individuals in entry-level healthcare occupations moved into occupations such as nursing that required higher education. While many individuals remained in their occupations throughout the study period, the results show that 28% of the sample moved out of these entry-level occupations and into another occupation. The most common "other" occupation categories were "office/administrative" and "personal care/services occupations." Whether these moves helped individuals advance their careers remains unclear. - The authors argue that employers and educational institutions should consider efforts to help clarify pathways to advance the careers of individuals in entry-level healthcare occupations. |
| Pitkälä, K., Finne-Soveri, H., Immonen, S., et al. (2018) Interdisciplinary team education promotes innovations in the home care of older people, Educational Gerontology, 44:4, 276-287, DOI: 10.1080/03601277.2018.1465283 | II- Programme evaluation | B- Gerontological team learning (Finland) | Theme 1- In-service Learning Opportunities (interprofessional education intervention) | - This article describes a new type of team training that involves undergraduate students of medicine, students from the Aalto University (industrial engineering and management, architecture, information networks, collaborative, and industrial design and bioinformation technology) and specialized home care nurses. During the course, the students learned interdisciplinary teamwork and created innovations in the care of older people. The 18 participants formed six micro teams (three persons in each team: one specialized nurse, one medical student and one from Aalto University). The course consisted of two seminars and 3 full days of home visits to older people’s homes. - Participants were encouraged to make one innovation in each home visit that would improve the older person’s well-being or streamline the processes of home care. During the course, the participants promptly formed tight teams. They valued the know-how of the other team members and learned openly from each other. They also created several practical innovations in home care which they presented to executives of older people’s care in a final seminar. The course received very good feedback from the students. - This course is an encouraging example of how gerontological interdisciplinary team training may be successfully applied. The article describes both the learning outcomes and the innovations the students produced during their home visits. It also discusses the learning theories behind effective interdisciplinary team learning. |
| Bookey-Bassett S. (2023). Feasibility testing of an interprofessional education intervention to support collaborative practice in home care for older stroke survivors with multiple chronic conditions. Journal of Interprofessional Care, 1–12. Advance online publication. https://doi.org/10.1080/13561820.2023.2262511 | II- Research (Mixed method) | B- Feasibility of interprofessional education for stroke home care (Canada) | Theme 1- In-service Learning Opportunities (interprofessional education intervention) | - This mixed-methods study examined the feasibility of implementing a new six-month, theory-based, interprofessional education intervention, and explored its effects and impact on collaborative practice among home care providers caring for older adult stroke survivors (≥65) with multiple chronic conditions. The evaluation utilized a qualitative descriptive and one group repeated measures design which included participant questionnaires, focus groups and field notes. Participants included 37 home care providers (registered nurses, physiotherapists, occupational therapists, personal support workers, care coordinators, and their supervisors) in Ontario, Canada. The intervention was feasible and acceptable to home care providers. - Perceived benefits included improved communication and collaboration within teams, enhanced role understanding, increased learning with and from each other, and increased appreciation of all team members' expertise. - From 3 to 6 months post initial IPE training, there was a statistically significant improvement in three domains of collaborative practice as measured by the Collaborative Practice Assessment Tool (communication/information exchange; community linkage and coordination of care; decision-making and conflict management) and one domain of collaborative practice, as measured by the 19-item Team Climate Inventory (task orientation). Implications for implementing interprofessional education in home care practice settings are described. Further testing in other populations and settings is warranted. |
| Kim, H., and Park, C. and Lee, N. (2020) Effects of Health Perception, Generativity and Wisdom on Job Competency of Korean Care Workers. Medico Legal Update, 20(2), 718–723. https://doi.org/10.37506/mlu.v20i2.1199 | II- Research (Quant) | Nurse-led interventions to support job competencies in care workers (China) | Theme 1- In-service Learning Opportunities (nurse-led interventions to enhance care worker competencies) | - The purpose of this study was to produce fundamental materials for nursing intervention development for care workers’ job competency enhancement. Material information was developed by analyzing impact of care workers’ health perception, generativity, and wisdom on job competency. - Data was collected by means of self-report questionnaire with ethical considerations from 116 care workers at two nursing homes and one care worker center in D Borough in D City, two nursing homes in J Borough and one nursing home in S Borough. The collected data was then analyzed by frequency scale, mean, standard deviation, t-test, ANOVA, Pearson correlational coefficients and stepwise multiple regression. - The care workers’ health perception scored 3.86 points out of 5 points, generativity scored 2.75 points out of 4 points and wisdom scored 3.49 points out of 5 points and job competency scored 3.79 points out of 5 points. The job competency of care workers was a moderate positive correlation with health perception (r=.49), generativity (r=.34), wisdom (r=.47). The health perception of care workers had a significant influence on job competency. Health perception, wisdom and work experience of care workers explained 39.6% of job competency. - Care workers should be provided with environmental and institutional strategies for constant service provision without career disruption. Job competency was affected by care workers’ health, wisdom and work experience, therefore, regular check-up and spur for maintenance of good health are required, in addition to constant refresher and capability enhancement education for extended wisdom on caring and life in daily lives. - A capability management program should be developed which takes these factors into consideration. Job competency increased in proportion to work years in elder caring, therefore, strategies for institutional management of manpower is needed at a policy-level. |
| Kelleher, D., Lord, K., Duffy, L., Rapaport, P., Barber, J., Manthorpe, J., Leverton, M., Dow, B., Budgett, J., Banks, S., Duggan, S., & Cooper, C. (2022). Time to reflect is a rare and valued opportunity; a pilot of the NIDUS-professional dementia training intervention for homecare workers during the Covid-19 pandemic. *Health & social care in the community*, *30*(5), e2928–e2939. https://doi.org/10.1111/hsc.13737 | II- Research (Qual) | B- Reflective practice in dementia training (England) | Theme 1- In-service Learning Opportunities (opportunities and time for peer learning using group reflective practices) | - Argues that most people living with dementia want to continue living in their own home for as long as possible and many rely on support from homecare services to do so. There are concerns that homecare often fails to meet the needs of clients with dementia, but there is limited evidence regarding effective interventions to improve its delivery for this client group. - This study aimed to assess whether a co-designed, 6-session dementia training intervention for homecare workers (NIDUS-professional) [*TP] was acceptable and feasible. Facilitated training sessions were delivered over 3 months, followed by 3, monthly implementation meetings to embed changes in practice. Two trained and supervised facilitators without clinical qualifications delivered the intervention via group video-calls during Oct 2020-March 2021 to a group of seven homecare workers from one agency in England. Participants provided qualitative feedback 3- and 6-months post intervention. - Qualitative interview data and facilitator notes were integrated in a thematic analysis. Adherence to the intervention and fidelity of delivery were high, indicating that it was acceptable and feasible to deliver in practice. Thirty of a possible 42 (71.4%) group sessions were attended. - Thematic analysis found one over-arching theme: 'Having time and space to reflect is a rare opportunity'. Within this four subthemes were (Having time to reflect is a rare opportunity; Reflecting with peers enhances learning; Reflection and perspective taking can improve care; Recognising skills and building confidence). The findings highlight how participants valued the intervention to discuss their work and learn new skills. - Attendance was lower for the implementation sessions, perhaps reflecting participants' lack of clarity about their purpose. The findings help to consider how to maintain positive impacts of the manualised sessions, so that these are translated into tangible, scalable benefits for people living with dementia and the homecare workforce. A randomised feasibility trial is underway. |
| Josset, J. M., Lavoyer, F., & Pâme, P. (2018). [Humanizing care through self-observation and the sharing of professional practices. Feedback from a training session in home caregiving services]. *Recherche en soins infirmiers*, (135), 55–59. https://doi.org/10.3917/rsi.135.0055 | II – Research (Qual) | B- Training session for home care (France) | Theme 1- In-service Learning Opportunities (opportunities to follow up on training and embedding training in practice) | - Since the French National Authority for Health first promoted the “ethics of care-giving” at the end of 2012, the humanization of home care remains a challenging topic. In order to handle the difficulties of managing a team of caregivers faced with strenuous work, practice exchange groups are often set up. However, there tends to be very little feedback on the implementation process. - By studying the accounts and comments of caregivers in the town of Bouguenais throughout a training session undertaken in the year 2017-2018, this study enriches this theoretical proposal with the pitfalls, stages, and discoveries of a concrete experiment. - The article highlights salient points such as the importance of self-observation, relationship to time, and the role of judgment in interactions with peers and patients. |
| Palesy D. (2016). Home Health Aide Training: An Appeal for Organizational Support. *Home healthcare now*, *34*(7), 381–387. https://doi.org/10.1097/NHH.0000000000000418 | II- Research (Qual) | B- HSW (Australia) | Theme 1- In-service Learning Opportunities (opportunities to follow up on training and embedding training in practice) | - How home healthcare aides (HHAs) adapt their classroom training to their workplaces is central to their own safety and that of their care recipients. A qualitative approach was adopted for this inquiry, where new workers were interviewed in-depth following their classroom training. - Findings suggest a perceived lack of supervisor support for classroom training and lack of follow-up in the workplace. Moreover, the need for more peer support was contended, and more comprehensive written materials in clients' homes may also assist workers' learning and enacting safe manual handling techniques in the workplace. The article concludes with recommendations for supporting HHAs' learning and includes suggestions for future research. |
| Tafvelin, S., Stenling, A., Lundmark R., & Westerberg, K. (2019) Aligning job redesign with leadership training to improve supervisor support: a quasi-experimental study of the integration of HR practices, European Journal of Work and Organizational Psychology, 28:1, 74-84, DOI: 10.1080/1359432X.2018.154188 | II- Research (Mixed method) | B- Job redesign and leadership training for home care supervisors (Sweden) | Theme 1- In-service Learning Opportunities (supervisor support and climate for innovation) | - This quasi-experimental study examines the alignment of a job redesign initiative with leadership training aimed at increasing supervisors’ opportunities for providing support to employees. In addition, the study examined intervention-mediated effects on climate for innovation through increases in perceived supervisor support. To test the hypothesized process, the study used employee ratings (N = 524) of perceived supervisor support and climate for innovation collected at three time points over 2 years in the home help services in seven Swedish municipalities. - Results of latent growth curve analyses showed that employees in the intervention group had a stronger and positive slope of perceived supervisor support relative to the comparison group. Further, the growth trajectories of perceived supervisor support were positively associated with climate for innovation at the 24-month follow-up. The study contributes to the human resource management literature by showing that alignment of employment practices such as training with work practices such as job redesign may be a promising strategy for achieving positive outcomes at multiple levels in organizations. |
| Stone, R.I. and Bryant, N.S. (2019) The Future of the Home Care Workforce: Training and Supporting Aides as Members of Home-Based Care Teams. J Am Geriatr Soc, 67: S444-S448. https://doi-org.proxy.library.rcsi.ie/10.1111/jgs.15846 | V- Commentary | B- Home Support Worker roles, training and inclusion in care teams (USA) | Theme 1- In-service Learning Opportunities (team-based initiatives and learning) | - Argues that home health, home care, and personal care aides provide most of the paid hands-on care delivered to seriously ill, functionally impaired individuals in their homes, assisted living, and other noninstitutional settings. This workforce delivers personal care, assistance with activities of daily living, and emotional support to their patients. They are often the eyes and ears of the health system, observing subtle changes in condition that can provide important information for clinical decision making and therapeutic intervention. Despite this fact, the growing number of team-based home care initiatives have failed to incorporate this workforce into their programs. - Suggests that barriers to inclusion of aides into teams include a basic lack of value and understanding on the part of clinical team members and society in general of the complex tasks that these caregivers perform, inadequate investments in training and education of this workforce to develop their knowledge and competencies, and variation in state delegation laws that limit the scope of practice and consequently the ability of aides to work effectively in teams and to advance in their careers. - Provides examples of USA team-based programs and educational interventions that include home care aides. Argues that building on the few programs that have successfully included aides as key members of home care teams, federal and state policymakers, educators, and health systems and providers should standardize competency-based training requirements, expand nurse delegation consistently across states, and support evaluation, dissemination, and replication of successful programs. |
| Olson, R., Hess, J. A., Parker, K. N., Thompson, S. V., Rameshbabu, A., Luther Rhoten, K., & Marino, M. (2018). From Research-to-Practice: An Adaptation and Dissemination of the COMPASS Program for Home Care Workers. *International journal of environmental research and public health*, *15*(12), 2777. https://doi.org/10.3390/ijerph15122777 | II- Programme evaluation | B- Home support worker training (Canada) | Theme 1- In-service Learning Opportunities (training that enables communities of practice) | - The COMmunity of Practice And Safety Support (COMPASS) program [*TP] was developed to prevent injuries and advance the health and well-being of home care workers. The program integrates elements of peer-led social support groups with scripted team-based programs to help workers learn together, solve problems, set goals, make changes, and enrich their supportive professional network. - After a successful pilot study and randomized controlled trial, COMPASS was adapted for the Oregon Home Care Commission's training system for statewide dissemination. The adapted program included fewer total meetings (7 versus 13), an accelerated meeting schedule (every two weeks versus monthly), and a range of other adjustments. The revised approach was piloted with five groups of workers (total n=42) and evaluated with pre- and post-program outcome measures. After further adjustments and planning, the statewide rollout was implemented. - In the adaptation pilot several psychosocial, safety, and health outcomes changed by a similar magnitude relative to the prior randomized controlled trial. - Preliminary training evaluation data (n=265) show high mean ratings indicating that workers like the program, find the content useful, and intend to make changes after meetings. Facilitating factors and lessons learned from the project may inform future similar efforts to translate research into practice. |
| Adisso, É. L., Taljaard, M., Stacey, D., Brière, N., Zomahoun, H. T. V., Durand, P. J., Rivest, L. P., & Légaré, F. (2022). Shared Decision-Making Training for Home Care Teams to Engage Frail Older Adults and Caregivers in Housing Decisions: Stepped-Wedge Cluster Randomized Trial. *JMIR aging*, *5*(3), e39386. https://doi.org/10.2196/39386 | II- Research (Quant) | B- Home care teams of fail older adults shared decision-making (Canada) | Theme 1- In-service Learning Opportunities (use of decision aides) | - Explores issue of frail older adults and caregivers who need support from their home care teams in making difficult housing decisions, such as whether to remain at home, with or without assistance, or move into residential care. - The study evaluated the effectiveness of adding web-based training and workshops for care teams in interprofessional shared decision-making to passive dissemination of a decision guide on the proportion of frail older adults or caregivers of cognitively impaired frail older adults reporting active roles in housing decision-making. - A cluster randomized trial with home care teams in 9 health centers in Quebec, Canada. Participants were frail older adults or caregivers of cognitively impaired frail older adults facing housing decisions and receiving care from the home care team at one of the participating health centers. The intervention consisted of a 1.5-hour web-based tutorial for the home care teams plus a 3.5-hour interactive workshop in interprofessional shared decision-making using a decision guide that was designed to support frail older adults and caregivers in making housing decisions. The control was passive dissemination of the decision guide. The primary outcome was an active role in decision-making among frail older adults and caregivers, measured using the Control Preferences Scale. Secondary outcomes included decisional conflict and perceptions of how much care teams involved frail older adults and caregivers in decision-making. The researchers performed an intention-to-treat analysis. A total of 311 frail older adults were included in the analysis. - Although the intervention slightly reduced decisional conflict for caregivers, shared decision-making training did not equip home care teams significantly better than provision of a decision aid for involving frail older adults and their caregivers in decision-making. |
| Blau, G., Chapman, S.A. and Neri, M. (2016), Testing the impact of career motivation on knowledge gained versus skills learned for a sample of personal/home care aide trainees, *Career Development International*, Vol. 21 No. 2, pp. 144-159. https://doi-org.proxy.library.rcsi.ie/10.1108/CDI-11-2015-0149 | II- Programme evaluation | B- Home care aides (USA) | Theme 1- In-service Learning Opportunities (Using appropriate learning approaches to gain skills and knowledge) | - Aims to distinguish knowledge gained versus skills learned as two learning-related training criteria; and to then test the impact of two career motivation variables, home care intent and stepping stone, for explaining these training criteria beyond controlled-for variables. - The research used a sample of 720 personal/home care aides (P/HCAs) who filled out pre-training and post-training surveys. Training consisted of 25 modules, lasting approximately 100 hours on various P/HCA knowledge bases, with training sessions generally five to six hours/day, four to five days/week over a three to four week period. Factor analyses, correlation, and hierarchical regression analyses were used to test the hypotheses. - Results were that these two learning outcomes, knowledge gained vs skills learned, could be differentiated and reliably measured. Subsequent hierarchical regression analyses showed additional discriminant validity for these two learning outcomes. For the two measured career motivation variables, home care intent and stepping stone, home care intent was positively related to both learning outcomes but stepping stone only had a significant positive impact on skills learned. Training delivery was significantly related only to knowledge gained, while instructor rating was significantly related only to skills learned. |
| Boscart, V., McCleary, L., Stolee, P. et al. (2020) Enhancing nursing home care for seniors: impact of a living classroom on nursing assistant’s education, Educational Gerontology, 46:8, 461-472, DOI: 10.1080/03601277.2020.1774842 | II- Research (Mixed method) | B- Living classrooms for nursing assistants (Canada) | Theme 1- In-service Learning Opportunities (workplace learning led by community colleges, living classroom | - Nursing Assistants (NAs) are the largest workforce in nursing homes, but often lack adequate preparation for their role. The Living Classroom (LC) is an integrated learning approach, whereby a NA program is delivered in a nursing home (NH) in collaboration with a community college. - This paper describes the implementation and evaluation of the LC. Mixed methods were used to gather data from 48 NA students, 5 faculty, and 42 NH staff over 30 weeks. Students, faculty, and nursing home staff described the LC as a positive learning experience. - Students’ gerontological knowledge increased over time (p = .0012). Students reported very positive relationships with program mentors and NH residents. The LC provides a unique approach to prepare NAs to work in nursing homes. This model could expand to other educational programs with a gerontology focus. |
| Castle, N. G., Furnier, J., Ferguson-Rome, J. C., Olson, D., & Johs-Artisensi, J. (2015). Quality of care and long-term care administrators' education: does it make a difference? *Health care management review*, *40*(1), 35–45. https://doi.org/10.1097/HMR.0000000000000007 | II- Research (Quant) | B- Home care administrator education and service quality (USA) | Theme 1- Leadership, Governance and Management Training (home care administrator/service management education) | - Long-term care administrators (administrators) can influence the care nursing home residents receive. However, little research has examined what factors of administrators are associated with how they influence care. - In this research, the association between administrators' education and quality of nursing home care is examined. In addition, the association between state educational and training requirements and quality of nursing home care is examined. Information collected from 3,941 administrators was matched with secondary data, including Nursing Home Compare; the Online Survey, Certification and Reporting data; and the Area Resource File. The quality indicators restraint use, catheter use, inadequate pain management, low-risk residents with pressure ulcers, and high-risk residents with pressure ulcers were examined. - Positive, statistically significant associations were found between the education level of administrators and all five quality indicators. Likewise, positive statistically significant associations were found between state educational requirements and state training requirements and the five quality indicators. - If these associations hold true, then promoting further educational attainment of individual administrators may influence quality of care. The state educational requirements and training requirements for administrators' licensure may represent an additional means of influencing the quality of care in nursing homes. |
| Banijamali , S., Jacoby, D. & Hagopian, A. (2014) Characteristics of Home Care Workers Who Leave Their Jobs: A Cross-Sectional Study of Job Satisfaction and Turnover in Washington State, Home Health Care Services Quarterly, 33:3, 137-158, doi: 10.1080/01621424.2014.929068 | II- Research (Quant) | C- Factors associated with leaving home care (USA) | Theme 1- Perceived Career Success (reasons to leave) | - Attracting and retaining a stable and motivated home care workforce has become a top policy priority. - The researchers surveyed 402 former home care workers in Washington State. The study compared these “leavers” to current home care workers recently surveyed. Those who left the profession were more highly educated, had higher household income, and were more likely to be White. Those newly employed have better benefits, wages, hours, and career mobility than in their home care jobs. The low status and poor pay of home care workers may result in the inability of the profession to retain those who face better prospects. |
| Skills for Care (2020) Being a personal assistant  pp. 28. Leeds, UK.  https://www.skillsforcare.org.uk/Recruitment-support/Support-individual-employers-PAs/Personal-Assistant/Hear-from-personal-assistants.aspx | V- Commentary | B- Personal assistant role in adult social care (England) | Theme 1- Preparation, entry, early experiences (clarity about role, role descriptions) | - Comprehensive information and guidance for personal assistants and those who are considering this career. A personal assistant works directly with one or more individuals, to support them with various aspects of their daily life so that they can live it in a way they choose. They are usually employed directly by a person who needs care and support, and who manages and pays for this through a personal budget or with their own money. This person is their employer (and are often referred to as an ‘individual employer’). They can also be employed by a family member or representative, when the person they are supporting doesn’t have the physical or mental capacity to be the employer. - This resource addresses key aspects relating to this profession, looking at: what a personal assistant is; what makes being a personal assistant a rewarding job; what personal assistants do; who can be a personal assistant; the recruitment process; starting work; working together; getting support; sorting out problems; learning and development; frequently asked questions; and how the personal assistant role fits into the world of social care and health. |
| Martyn, J., Wilkinson, A., Zanella, S. (2022) Identifying the continuing education needs of personal care workers in two residential aged care facilities by an appreciative inquiry study, Collegian,29, 6:887-893. https://doi.org/10.1016/j.colegn.2022.09.009. | II- Research (Qual) | B- Training needs of personal care workers (Australia) | Theme 1- Preparation, entry, early experiences (preparatory/induction programmes, new starter support systems) | - Personal care workers make up most of the workforce in residential aged care facilities, but they are under-served by continuing education providers. - This study aimed to explore the Continuing Education needs of personal care workers from the perspectives of care staff at two residential aged care facilities in regional Queensland, Australia. This appreciative inquiry study used a visioning activity to inspire thoughts of the ideal PCW during the interviews and focus groups about the continuing education needs of personal care workers. - Gaps exist in the preservice preparation and continuing education of personal care workers, despite a reliance on the personal care worker role to be the 'eyes and ears' of other health professionals. The personal care workers identified their aspirational education needs for ‘best’ practice. - Personal care workers must be adequately prepared through evidence-based continuing education to respond to residents' emerging needs. However, there was an educational mismatch for them because the PCW curriculum does not adequately prepare the participants for their role. Identifying personal care workers continuing education needs will enhance practice and improve the quality of care. Improving personal care worker education will address several public concerns about the quality of care in residential aged care facilities. - The personal care worker preservice education curriculum is inadequate. Improved personal care worker knowledge and skills are possible when the participants inform the curriculum. Furthermore, if aspirational curricula are designed by potential participants, then continuing education can build ideal practices. |
| Shinan-Altman, S., Riabzev, A., & Ayalon, L. (2019). Mixed motivations to provide formal care to older adults: lessons from a training program. *International psychogeriatrics*, *31*(3), 341–349. https://doi.org/10.1017/S1041610218000868 | II- Research (Qual) | B- Recruitment of young workers to home care (Israel) | Theme 1- Preparation, entry, early experiences (promoting and valuing home care as purposeful, meaningful, rewarding work) | - With the aging of the population, the demand for long-term services is increasing accordingly and the recruitment of paid caregivers to older adults has become a critical issue. Hence, there is a great need to train people in the care of older adults. - This study examined motivations to participate in a new program, which aims to train young people (ages 19-25) to become paid caregivers for older adults. The study is based on focus groups with program participants (N = 33) and on phone interviews with former participants (N = 8) and face-to-face interviews with staff members (N = 14). Transcripts were analyzed using qualitative content analysis. - Three major themes emerged: (1) "Motivations to participate in the program," referred to participants' motivations to work with older adults. (2) "Reduced motivations due to ambiguity of roles," referred to the unclear definition of the care worker's role, which created a decrease in participants' motivations to provide care. (3) "Inadequate financial compensation," concerned participants' reduced motivation to participate in the program as a result of financial promises that were not realized. - Findings suggest that it is important to be cautious about the motivations for participation in the program because by emphasizing the difficult situation of older adults, one stresses the low status assigned to older adults, rather than positive attributes. It is also suggested that care workers should be financially rewarded, so that despite the difficulties that may arise during work, they will feel valued and will want to remain in the profession. |
| Wang, J. & Wu, B. (2017) Domestic helpers as frontline workers in China’s home-based elder care: A systematic review, Journal of Women & Aging, 29:4, 294-305, doi: 10.1080/08952841.2016.1187536 | I- Systematic review | B- Domestic workers providing care for older adults (China) | Theme 1- Preparation, entry, early experiences (specifics of the job role, responsibilities, and rights) | - A systematic review of the existing empirical studies focusing on Chinese domestic helpers in mainland China and foreign domestic helpers in Hong Kong who provide care for community-dwelling older adults. There are very limited studies specifically focusing on this population. - The findings synthesized domestic helpers’ characteristics, acknowledged their contributions to elder care in China, and showed multiple challenges facing them, such as issues related to their physical health and emotional well-being, lack of legal rights protection, difficulties of adapting life in the host city, lack of training, and risk of abuse and sexual harassment. - The review findings support the need for developing training and educational programs about legal rights protection and cultural competency for domestic helpers and the need to promote domestic helpers’ access to health care and social welfare and opportunities for career advancement, and provide respectful working conditions. |
| Atkinson, C., Crozier, S. and Lewis, L. (2016) Factors that affect the recruitment and retention of domiciliary care workers and the extent to which these factors impact upon the quality of domiciliary care: interim findings summary Welsh Government Social Research pp10. Cardiff, Wales.https://www.gov.wales/sites/default/files/statistics-and-research/2019-07/160118-factors-affect-recruitment-retention-domiciliary-care-workers-interim-en.pdf | II- Research (Mixed method) | B- Domiciliary care staff recruitment and quality of care (Wales) | Theme 1- Preparation, entry, early experiences (values for home care, humanistic care abilities) | - Reports on interim research findings which explore the factors that influence the recruitment and retention of domiciliary care workers and the extent to which these factors impact upon the quality of domiciliary care. The research consisted of a literature review and qualitative data collected from seven local authority commissioners, 32 registered domiciliary care managers and 41 domiciliary care workers using telephone interviews and focus groups. Factors identified as having a negative impact on the recruitment and retention of domiciliary care workers included pay, working hours, working environment, lack of training and development opportunities, and a lack of career structure. - Participants felt factors having a positive influence on recruitment and retention were largely related to individual motivations about care work, including satisfaction gained from caring for clients, work flexibility and a lack of alternative options of employment. The extent to which these factors impact on care quality are discussed in the following areas: reliability, continuity, flexibility, staff attitudes, and skills and knowledge. The research highlights the need for a well-trained, well-paid and secure workforce with appropriate working patterns in order to encourage the recruitment and retention care workers and to deliver high quality care. The report provides recommendations to the Welsh Government on how they could improve the quality of domiciliary care through policy that has a positive influence on individuals to become and remain working as domiciliary care workers. Findings will also inform a public consultation on policy interventions to improve the quality of domiciliary care. (Edited publisher abstract) |
| Zhang, T., Zhang, L., Wen, X., & Li, H. (2023). Level of humanistic care ability and its influencing factors among nursing aides in long-term care facilities. Geriatric nursing (New York, N.Y.), 52, 56–62. https://doi.org/10.1016/j.gerinurse.2023.05.007 | II- Research (Quant) | C- Nursing aides’ humanistic care ability (China) | Theme 1- Preparation, entry, early experiences (values for home care, humanistic care abilities) | - The objective of this study was to determine the level and influencing factors of the humanistic care ability (HCA) in nursing aides, thus providing a baseline for its improvement. This study investigated 302 nursing aides in six long-term care facilities (LTCFs) in Suzhou between December 2021 and June 2022 by convenience sampling. A descriptive questionnaire and the Caring Ability Inventory were applied in this study. - The HCA was at a low level, and its influencing factors were education level, marital status, personality, reason for employment, and the degree of perceived care from colleagues (p<0.05). - Concludes that nursing aides' HCA needs to be strengthened urgently. Nursing aides with poor education, widowed, single, and an introversion type of personality should receive more attention. Additionally, creating a warm atmosphere among colleagues and cultivating the nursing aides' motivation for eldercare will help improve their HCA. |
| Brown, P., Leverton, M., Burton, A., Harrison-Dening, K., Beresford-Dent, J., & Cooper, C. (2022). How does the delivery of paid home care compare to the care plan for clients living with dementia?. *Health & social care in the community*, *30*(5), e3158–e3170. https://doi.org/10.1111/hsc.13761 | II- Research (Document analysis) | B- Care plans (Finland) | Theme 2- Care Planning and Care Plans (home care plans) | - Many people living with dementia choose to remain in their own homes, supported by home-care workers, who provide care that is specified in care plans. - This study explored how care plans of clients living with dementia, compared with ethnographic observations of home care they received. In a secondary, reflexive thematic analysis, the researchers reviewed care plans for 17 clients living with dementia and transcripts from 100 h of observations with 16 home-care workers delivering care to them. - The overarching theme was: Care plans as a starting point but incomplete repository. Clients' care plans provided useful background information but did not reflect a wealth of knowledge home-care workers built through practice. Two sub-themes described: (a) Person-centred care planning: whether and how the care plan supported tailoring of care to clients' needs and (b) Filling in the gaps: home-care workers often worked beyond the scope of vague, incomplete or out-of-date care plans. The analysis found considerable inconsistencies between care plans and the care that was delivered. Care plans that were comprehensive about care needs, and rich in person-specific information aided the delivery of person-centred care. Lack of documentation was sometimes associated with observed failures in person-centred care, as helpful information and strategies were not shared. Including information in care plans about how, as well as what care tasks, should be completed, and frequently discussing and updating care plans can create more person-centred plans that reflect changing needs. Electronic care planning systems may support this. |
| Turjamaa, R., Hartikainen, S., Kangasniemi, M., & Pietilä, A. M. (2015). Is it time for a comprehensive approach in older home care clients' care planning in Finland? Scandinavian journal of caring sciences, 29(2), 317–324. https://doi.org/10.1111/scs.12165 | II- Research (Document analysis) | B- Care plans nursing (Finland) | Theme 2- Care Planning and Care Plans (patient care plans/nursing care plans) | - Home-care services require access to high quality information. Apart from the provision of right-time organised planning of care and to document information about clients' needs, in home care, the care planning is intended to facilitate continuity and individual nursing through nursing documentation of the assessment of the client. - The aim was to describe the contents of older (+75 years) home-care clients' electronic care and service plans and to evaluate how the clients' resources have been taken into account. - The data were collected from the care and service plans (n = 437) of home-care services during July 2010. The data were analysed by quantitative methods and by thematic content analysis. - Based on the analysis, medication was the most reported component in all plans (92.7%); other commonly reported components were self-care (85.4%) and coping (78.0%). Components within respiratory, follow-up treatment, life cycle and health behaviour were forgotten. Most of the care and service plans were designed from the home-care professionals' point of view but the plans lacked the perspective of older clients. - To be able to promote older home clients' ability to live at home, home-care planning needs to be individually designed and must take into account clients' needs and their perspectives regarding meaningful activities and social relationships. In addition, there is a need to develop a more comprehensive care planning system, based on the clients' individual needs and standards of care planning. |
| Sterling, M. R., Silva, A. F., Leung, P. B. K., Shaw, A. L., Tsui, E. K., Jones, C. D., Robbins, L., Escamilla, Y., Lee, A., Wiggins, F., Sadler, F., Shapiro, M. F., Charlson, M. E., Kern, L. M., & Safford, M. M. (2018). "It's Like They Forget That the Word 'Health' Is in 'Home Health Aide'": Understanding the Perspectives of Home Care Workers Who Care for Adults With Heart Failure. Journal of the American Heart Association, 7(23), e010134. https://doi.org/10.1161/JAHA.118.010134 | II- Research (Qual) | B- Home care workers for heart failure (USA) | Theme 2- Care Planning, Care Plans and Coordination of Care (communication and coordination of care) | - Home care workers (HCWs) increasingly provide long-term and posthospitalization care for community-dwelling adults with heart failure (HF). They observe, assist, and advise these patients, yet few studies have examined their role in HF. - As the foundation for future interventions, this study sought to understand the perspectives of HCWs caring for adults with HF. - The study involved 8 focus groups in partnership with the Home Care Industry Education Fund, a benefit fund of the 1199 Service Employees International Union United Healthcare Workers East, the largest healthcare union in the United States. English- and Spanish-speaking HCW s with HF clients were eligible to participate. Data were analyzed thematically. Forty-six HCW s employed by 21 unique home care agencies participated. - General and HF-specific themes emerged. Generally, HCWs (1) feel overworked and undervalued; (2) find communication and care to be fragmented; (3) are dedicated to clients and families but are caught in the middle; and, despite this, (4) love their job. With respect to HF, HCW s (1) find it frightening and unpredictable; (2) are involved in HF self-care without any HF training; and (3) find the care plan problematic. - Although frequently involved in HF self-care, most HCW s have not received HF training. In addition, many felt poorly supported by other healthcare providers and the care plan, especially when their clients' symptoms worsened. Interventions that provide HF -specific training and aim to improve communication between members of the home health care team may enhance HCW s' ability to care for adults with HF and potentially lead to better patient outcomes. |
| Hines D. (2014). Cultural competence: assessment and education resources for home care and hospice clinicians. Home healthcare nurse, 32(5 Suppl), S4–S11. https://doi.org/10.1097/NHH.0000000000000080 | III- Review of resources | C- Culturally competent care the home environment (USA) | Theme 2- Care Planning, Care Plans and Coordination of Care (culturally competent organisational policies and practices) | - Home healthcare and hospice clinicians face many challenges in the complex healthcare system caring for patients and their families in the home environment. One of those challenges is providing culturally competent care for an increasingly diverse population. - This article highlights free, easily accessible, online resources to assist clinicians and organizations to assess organizational and individual cultural competence and provide many resources for cultural competency education programs. |
| Puustinen, J., Kangasniemi, M., Pasanen, M., & Turjamaa, R. (2023). Recognising older people's individual resources and home-care-specific tasks in home care in Finland: A document analysis of care and service plans. *Scandinavian journal of caring sciences*, *37*(2), 507–523. https://doi.org/10.1111/scs.13135 | II- Research (Document analysis) | B- Care plans (Finland) | Theme 2- Care Planning, Care Plans and Coordination of Care (home care plans) | - Comprehensive care and service planning in home care is tailored to older people's individual needs and resources in order to support them living at home. However, little is known about how these individual resources and home-care-specific tasks are recognised in older people's care and service plans. - This article aims to describe the content of care and service plans in older people's home care with special attention to their individual resources and home-care-specific tasks. This was a document-based cross-sectional study with mixed-methods analysis, carried out in Eastern Finland during Spring 2018. A document analysis using the deductive Finnish Care Classification (FinCC), and an inductively developed framework of older people's care and service plans (n = 71). The data were analysed with descriptive statistical methods. - Altogether, 1718 notes were relevant to the FinCC main categories: 707 (41%) focused on older people's needs and 1011 (59%) on nursing interventions. The researchers identified 1104 notes based on the 26 inductively developed main categories: the majority (n = 628, 57%) focused on individual resources and the remainder (n = 476, 43%) on home-care-specific tasks. Increasing age resulted in fewer notes on safety and sensory functions. There were fewer notes on resources related to sleeping and wakefulness after longer care and service periods. An increased number of home visits resulted in more documentation on tasks related to pharmaceutical issues, including repeat prescriptions. - Individual resources for older people were documented, to some extent, in their care and service plans. It is necessary to review these alongside home-care-specific tasks that support older people's independence and safety at home. Individual resources need to be recognised in order to enable home-care professionals to provide tailored, high-quality home care services. Home-care-specific tasks should be supported by documentation with updated, sensitive home care classifications. |
| Sjöberg Forssberg, K., Parding, K. and Vänje, A. (2021), "Conditions for workplace learning: a gender divide?", Journal of Workplace Learning, Vol. 33 No. 4, pp. 302-314. https://doi-org.proxy.library.rcsi.ie/10.1108/JWL-08-2020-0134 | II- Research (Qual) | B- Gender inequalities in workplace learning, home care (Sweden) | Theme 2- Inclusion and Voice in Teams, Organisations and Policymaking (marginalisation of the workforce, lack of involvement in levels of decision making) | - This paper examines and discusses conditions for workplace learning in gender-segregated workplaces in the public sector, how social constructions of gender contribute to (or constrain) the workplace learning conditions within two workplace contexts. - The research was carried out through an interactive approach with data from 12 semi-structured interviews with workers and first-line managers from technical maintenance and home care in a Swedish municipality, validated at an analysis seminar with 27 participations, from both workplace contexts the Swedish Work Environment Authority and us researchers. - The results indicate that gender affects conditions for workplace learning and contributes to an enabling learning environment in the male-dominated workplace context and to a constraining learning environment in the female-dominated workplace context. The identified differences are created in both organisational structures and the organisations cultures. - When analysing conditions for workplace learning from a gender perspective, the approach of comparative, cross-case analyses is useful. An interactive approach with women and men describing and analysing their work experiences together with researchers is a fruitful way of making gender visible. The theoretical approach in this study illuminates how social constructions of gender operate and affect conditions for workplace learning and contributes to a deeper understanding of underlying causes to unequal conditions in different workplace contexts. - The findings imply a gender divide which, from the theoretical strands, can be seen as an expression of asymmetrical power relations and where these gendered learning conditions probably also affect the quality of the services. The findings contribute to existing gender theoretical literature by demonstrating that gender is essential to take into consideration when understanding working conditions in different workplace contexts. This study contributes to workplace learning literature by exploring the different ways in which social constructions of gender contribute to enabling and constraining learning environments. |
| Lolich, L., & Timonen, V. (2020). Fortunate and fearful: emotions evoked by home-care policies for older people in Ireland. *Emotions and Society*, *2*(1), 61-78. | II- Research (Qual) | A- Home care policy (Ireland) | Theme 2- Inclusion and Voice in Teams, Organisations and Policymaking (lack of policy influence or control over policy impact) | - This article examines the emotions of fear and feeling fortunate experienced by key actors in home-care services in Ireland. The authors take a relational approach to emotions; that is to say, an understanding that emotions are produced in social interactions and play an essential part in how people engage with, and respond to, long-term care policies. - The study involved focus groups and in-depth interviews with 104 participants. - The findings show that the most vulnerable participants - service users and care workers on precarious contracts - feel fortunate or fearful about outcomes that had, or would have, a direct impact on them: respectively, having a good carer and obtaining job satisfaction, or losing a home-care package and not having enough work. Professionals were more likely to speak about luck and fear, not in relation to what could happen to them directly but in relation to the fate of service users and care workers. - The unregulated home-care services in Ireland have influenced actors to construe their own and others' participation in the system as increasingly individualised, where desired outcomes depend on one's good luck or strong personal relationships. For the system to work properly trust needs to be present not only at the micro level of individual relationships but also at a system level. This could lead to a decline in emotions that centre on feeling fortunate and fearful, and an increase in expressions of trust and a sense of control by both care providers and care recipients. |
| Bensliman, R., Casini, A., & Mahieu, C. (2022). "Squeezed like a lemon": A participatory approach on the effects of innovation on the well-being of homecare workers in Belgium. *Health & social care in the community*, *30*(4), e1013–e1024. https://doi.org/10.1111/hsc.13506 | II- Research (Qual) | B- Working conditions in home care (Belgium) | Theme 2- Inclusion and Voice in Teams, Organisations and Policymaking (preventing unintended negative impact of innovations in home care) | - Innovative programs that emerge in response to the rapidly changing care needs of older adults provide an opportunity to study the transformations in working and employment conditions within the homecare sector. This study aimed to understand how innovations introduced in the homecare sector have affected the well-being of homecare workers providing non-medical domestic support to older adults who wish to age in place. - The study is based on a participatory approach involving homecare workers exposed to two innovations in Wallonia (Belgium) that relate to flexible working hours, worker training, and technological equipment. It involved a literature review, six semi-structured individual interviews with managers, and eight workshops based on the 'Group Analysis Method' involving 9 to 12 homecare workers. - The results revealed that the innovations deteriorated working conditions, intensified occupational psychosocial risk factors, and impacted work-life balance. This gave rise to tensions that ultimately had a negative impact on the well-being of workers and on the quality of their care relationship with older adults/caregivers, while also weakening the viability of the services. The workers proposed some avenues to improve and regulate these tensions. |
| Faul, A. C., Schapmire, T. J., D'Ambrosio, J., Feaster, D., Oak, C. S., & Farley, A. (2010). Promoting sustainability in frontline home care aides: Understanding factors affecting job retention in the home care workforce. Home Health Care Management & Practice, 22(6), 408-416. | II- Research (Quant) | C- Home care aide retention in agencies (USA) | Theme 2- Inclusion and Voice in Teams, Organisations and Policymaking (vulnerability to exploitation) | - With the growth of the older adult population increasing at an exponential rate, caring for this population has become increasingly difficult. As many choose to age in place (i.e., in the home environment), both the public and private sectors are being forced to respond. The emergence of home health care and the employment of home care aides is one of the ways that the industry has evolved to meet this crisis. However, retention of home care aides has proven problematic. - This study explores factors affecting home care aide retention in agencies that employ home care aides. A sample of 116 home care aides employed by three agencies was surveyed to identify retention issues relative to the home care aide. A hypothetical model of home care aid job retention was tested with a hierarchical regression analysis, where 11 variables were entered in five steps, using a time-sequenced order. - The results indicated that the hypothetical model was able to explain 41% of the variance in months employed as home care aides, with age, education, wages earned, and intrinsic satisfaction as the most significant contributors to the model. Being able to predict retention of home care aides allows employing agencies to realign themselves to retain workers and improve quality of care. - The authors argue that it is important to realise that the same traits that predict retention among home care aides may also leave these workers vulnerable to exploitation. |
| Snyder R. E. (2019). Commentary on the Future of the Home Care Workforce: Training and Supporting Aides as Members of Home-Based Care Teams. Journal of the American Geriatrics Society, 67(S2), S449–S450. https://doi.org/10.1111/jgs.15868 | V- Commentary | C- Workforce planning (USA) | Theme 2- Inclusion and Voice in Teams, Organisations and Policymaking (inclusion in teams and their decision making) | - Training for and integration of home care aides into home care teams are important underpinnings of age-friendly health systems that allow older adults to age in place, where they overwhelmingly want to be. Home care aides can play a critical role in keeping what matters to the older adult at the centre of all care decisions. - State initiatives, such as Hawaii’s Kapuna Caregivers Program, illustrate how important it will be to implement the recommendations by Stone and Bryant to bolster the training and inclusion of the home care workforce to improve care. They suggest standardising competency training to ensure that aides are appropriately trained and incorporated into team care; increasing investments in geriatric and team training; expanding career path opportunities including nurse delegation; and evaluation of existing programs to integrate aides into team-based care. They assert that to succeed requires the full recognition by clinicians, health system leaders, policymakers, and the public that inclusion of home care aides is essential. |
| Gleason, H. P. (2020). The Effect of Job Demands, Control, and Support on Job Satisfaction and Intent to Leave among Massachusetts Home Health and Supportive Home Care Aides (Doctoral dissertation, University of Massachusetts Boston). | II- Research (Mixed method) | C- Home health aide workforce (USA) | Theme 2- Job Satisfaction and Retention (factors associated with job satisfaction) | - The Home Health Aide (HHA) workforce is one of the fastest growing occupations in the country. Despite being a high demand field, the workforce is plagued by instability due to low recruitment and poor retention, not surprising outcomes for positions that are physically and emotionally demanding, generally low paid, with inconsistent hours. A better understanding of the factors that influence the HHAs’ job satisfaction and intent to leave the job is critical to building a strong workforce capable of providing high quality care. - This study examined the effect of job demands on satisfaction and intent to leave for HHAs as compared to Supportive Home Care Aides (SHCAs), an advanced HHA position in Massachusetts, to determine if aspects of the SHCA position, enhanced control and support, positively influence their work outcomes. Employing a mixed methods research design, the quantitative portion used generalized estimating equations (GEEs) to determine the direct and moderation effect of job demands, control, and support on worker outcomes and the qualitative portion used focus groups with HHAs to further investigate the impact of job demands, control, and support on the individual worker’s experiences on the job. - The quantitative results support a positive direct and moderation effect for control and support on worker outcomes, though the effect varied considerably by type of worker and by outcome. The qualitative results support the quantitative findings, highlighting the important role of control and support on HHAs’ overall experience on the job. The focus groups also brought attention to the many challenges of the job, including the responsibilities HHAs take on that often go beyond their job description. - This research has implications for home care policy and practice. Identifying job factors that positively influence a HHA’s job satisfaction or decision to remain on the job can greatly benefit home care agencies that are seeking to retain their workforce and attract new workers to the field. Implementing mechanisms, such as adoption of advanced HHA positions, to enhance the control HHAs have over their job and the amount of support they receive should be a priority for home care industry leaders. |
| Atkinson, C., Crozier, S., Lewis, L. (2016) Factors that affect the recruitment and retention of domiciliary care workers and the extent to which these factors impact upon the quality of domiciliary care. Welsh Government Social Research. Pp. 171. Cardiff, Wales. https://www.gov.wales/sites/default/files/statistics-and-research/2019-07/160317-factors-affect-recruitment-retention-domiciliary-care-workers-final-summary-en.pdf | II- Research (Mixed method) | B- Recruitment and retention of domiciliary care workers (Wales) | Theme 2- Job Satisfaction and Retention (factors associated with job satisfaction) | - Final report of research to examine the factors that affect the recruitment and retention of domiciliary care workers and the extent to which these factors impact upon the quality of domiciliary care. It focuses specifically on the influence of employment terms, conditions and career structures for domiciliary care workers. - The project consisted of a systematic literature review and data analysis. For the data analysis focus groups and one-to-one interviews were conducted with seven domiciliary care commissioners, 32 registered managers of domiciliary care providers and 41 domiciliary care workers. Workshops were also attended by an additional 17 commissioners and 16 registered managers. - Issues identified in relation to terms and conditions included: lack of time and funding to access training; low rates of pay which were often constrained by funding levels, especially in the independent sector; the use of zero hours contracts in the independent sector; and long working hours. Managers felt that working conditions and career structures acted as both a barrier to the recruitment and retention of staff. - The report discusses the implications of employment terms and conditions on six aspects of care quality: skills and knowledge, flexibility, continuity, reliability, communications and staff attitudes. Suggestions for future research and policy options to improve the quality of domiciliary care by positively influencing the recruitment and retention of domiciliary care workers are included. |
| Gleason, H. P., Miller, E. A., & Boerner, K. (2023). Focusing on the Positive: Home Health Aides’ Desire for Autonomy and Control. *Journal of Applied Gerontology*, *42*(4), 728-736. https://doi.org/10.1177/07334648221145177 | II- Research (Mixed method) | C- Home health aide workforce (USA) | Theme 2- Job Satisfaction and Retention (factors associated with job satisfaction) | - Home Health Aides (HHAs) are one of the fastest growing workforces in the country, yet the industry struggles to recruit and retain workers. This study explored HHAs’ experiences with the level of control, autonomy, and decision-making authority in their work. Six focus groups with 37 HHAs were conducted in Massachusetts. - Findings showed that HHAs viewed control as a positive job characteristic, which attracted them to and led them to remain in the position. Positive benefits included having control over client selection, location, hours, and the ability to determine their day-to-day tasks and schedules. - The study results highlight the value that HHAs place on autonomy and control and the potential benefit that these job qualities have for greater recruitment and retention of these workers. Amplifying control by bolstering training and expanding scope of practice may entice new individuals to pursue a HHA career and help maintain those currently in the position. |
| Ruotsalainen, S., Jantunen, S. & Sinervo, T. (2020) Which factors are related to Finnish home care workers’ job satisfaction, stress, psychological distress and perceived quality of care? - a mixed method study. *BMC Health Serv Res* 20, 896. https://doi-org.proxy.library.rcsi.ie/10.1186/s12913-020-05733-1 | II- Research (Qual) | C- Working conditions and practices of home care teams (Finland) | Theme 2- Job Satisfaction and Retention (factors associated with job satisfaction) | - The desire to increase the role of home care in Finland has created problems in home care work. Working conditions have deteriorated, the quality of care experienced is low, and staff members suffer from time pressure and stress, amongst other things. - The aim of this article is to explore the challenges, stressors, teamwork and management factors that are associated with home care staff members’ well-being, job satisfaction and experienced care quality, and further, how staff members experience their work. A survey was sent to home care workers in two case organizations that participated in the study. In addition, semi-structured theme interviews with home care workers were conducted. The data from the survey was analysed using analysis of covariance, and interview data was analysed using the Grounded Theory-based method from Gioia et al. - Respondents of the survey and the interview participants were mainly female practical nurses. The results from the survey showed, for example, that time pressure was associated with higher stress and psychological distress, and interruptions were associated with lower job satisfaction and higher stress. In addition, variables related to teamwork, such as participative safety, were shown to explain the variation in quality of care. The analysis of the interview data further brought up dissatisfaction with management practices, which seems to have led to a decrease in job satisfaction. Exhaustion and strain were present among staff members, which originated from an insufficient number of carers. - Current working conditions and work practices in Finnish home care are stressful. The results from this study indicate that having more autonomy at work was associated with job satisfaction, according to both analyses. Team climate and idea implementation were related to quality of care. Therefore, increasing self-organizing team practices might be a possible development method for improving working conditions and staff members’ well-being. Implementing self-organizing team practices could possibly also attract employees to work in home care and prevent turnover. |
| McCreary D. D. J. (2020). Home Health Nursing Job Satisfaction and Retention: Meeting the Growing Need for Home Health Nurses. *The Nursing clinics of North America*, *55*(1), 121–132. https://doi.org/10.1016/j.cnur.2019.11.002 | II- Research (Quant) | B- Job satisfaction and retention in home nursing (USA) | Theme 2- Job Satisfaction and Retention (factors associated with job satisfaction) | - To meet the significant increase in the demand for home health care, retention of home health nurses is essential. Job satisfaction is the major determinant of retention. - Assessment of satisfaction indicators is a useful method to inform a home health agency plan to improve job satisfaction of home health nurses. Satisfaction was assessed using a standardized instrument, the Home Healthcare Nurse Job Satisfaction scale. The outcomes of a quality improvement process informed the development of a retention plan strategy to help leaders retain this important nursing work force in home health. |
| Carr, S., (2014) Pay, conditions and care quality in residential, nursing and domiciliary services. Joseph Rowntree Foundation pp. 8. York, UK. https://myhomelife.org.uk/wp-content/uploads/2015/02/JRF-report-on-care-pay-conditions-summary.pdf | II- Research (Mixed Method) | B- Social care staff motivation and retention (England) | Theme 2- Job Satisfaction and Retention (factors that promote good work and retention) | - This paper summarises the findings of a Joseph Rowntree Foundation study that looked at staff pay, status and quality of care in social care. It supplements this with messages from two Joseph Rowntree Foundation reports on rewarding work for low-paid employees and career progression for workers in a number of sectors, including the care sectors. Specifically this summary explores what motivates and supports care workers to do a good job and deliver quality care. - The main points are summarised under three headings: motivation and values; pay and progression; and conditions to do the job well. The summary highlights the essential part working conditions and organisational culture play in ensuring staff feel valued and that recruitment and retention of staff is maximised. - Care workers do demanding work for low pay. Evidence on the direct causal relationship between increased pay and improved care quality is as yet inconclusive. However, research shows the importance of making staff feel valued; chances for progression; managerial support and human resource management that is proportional. - The low social status accorded to care work needs to be addressed as it influences how the whole sector is perceived and reward understood. - In order to provide the appropriate conditions, rewards and support to staff, organisations need to understand the personal motivation of care workers. Many are loyal to the sector because they are motivated by a primary commitment to service users. - Working conditions and organisational culture are essential parts of the overall approach to ensuring low-paid staff feel valued and satisfied, recruitment and retention of talented staff is maximised, and the continuity of care associated with quality is maintained. - Employee retention is important in a sector where staff continuity is needed to ensure relationship-building between care worker and service user that is of vital importance to care quality, particularly for older people. |
| Hsu, W., & Shih, F. P. (2023). Key Factors for Enhancing Home Care Workers' Intention to Stay by Multiple-Criteria Decision Analysis. *Healthcare (Basel, Switzerland)*, *11*(5), 750. https://doi.org/10.3390/healthcare11050750 | II- Research (Mixed method) | B- Factors that promote retention of home care workers (Taiwan) | Theme 2- Job Satisfaction and Retention (factors that promote good work and retention) | - The ageing population is increasing rapidly in Taiwan, where the ageing rate exceeds even that of Japan, the United States and France. The increase in the disabled population and the impact of the COVID-19 pandemic have resulted in an increase in the demand for long-term professional care, and the shortage of home care workers is one of the most important issues in the development of such care. - This study explores the key factors that promote the retention of home care workers through multiple-criteria decision making (MCDM) to help managers of long-term care institutions retain home care talent. A hybrid model of multiple-criteria decision analysis (MCDA) combining Decision-Making Trial and Evaluation Laboratory (DEMATEL) and the analytic network process (ANP) was employed for relative analysis. Through literature discussion and interviews with experts, all factors that promote the retention and desire of home care workers were collected, and a hierarchical MCDM structure was constructed. Then, the hybrid MCDM model of DEMATEL and the ANP was used to analyze the questionnaire data of seven experts to evaluate the factor weights. - According to the study results, the key direct factors are improving job satisfaction, supervisor leadership ability and respect, while salary and benefits are the indirect factor. This study uses the MCDA research method and establishes a framework by analyzing the facets and criteria of different factors to promote the retention of home care workers. - The results will enable institutions to formulate relevant approaches to the key factors that promote the retention of domestic service personnel and to strengthen the intention of Taiwan's home care workers to stay in the long-term care industry. |
| Karmacharya, I., Janssen, L. M., & Brekke, B. (2023). “Let Them Know That They're Appreciated”: The Importance of Work Culture on Direct Care Worker Retention. Journal of Gerontological Nursing, 49(8), 7-13. | II- Research (Qual) | B- Factors that support quality and retention of direct care workforce (USA) | Theme 2- Job Satisfaction and Retention (factors that promote good work and retention) | - Retaining direct care workers (DCWs) in all areas of long-term services and supports (LTSS) remains an unending challenge. The current study purposively selected 12 high performing LTSS communities (e.g., nursing homes, assisted living, home care agencies) in Ohio with high family satisfaction and staff retention. Qualitative interviews were conducted in 2022 with 21 personnel in leadership positions and 16 DCWs (e.g., certified nursing assistants, personal care aides). Interviews were audio recorded, transcribed, and a team of two coders conducted the thematic analysis. - Three themes of work culture emerged as important for retention: (1) Family-Like Organizational Approach, (2) Supportive Working Conditions, and (3) Worker Empowerment. - DCWs value empathic relationships with their clients and supervisors. Favorable working conditions consisted of choice, participative leadership, and effective communication. DCWs experienced empowerment through appreciation, financial incentives, and career advancement opportunities. Findings suggest practical changes for person-centered care practitioners that are feasible across any organizational work culture setting. |
| Tangchitnusorn, K., & Prachuabmoh, V. (2023). Motivation and Quality of Work Life of In-Home Paid Caregivers of Older Adults. Journal of applied gerontology : the official journal of the Southern Gerontological Society, 42(4), 737–746. https://doi.org/10.1177/07334648221145167 | II- Research (Qual) | C- The experiences of in-home paid caregivers (Thailand) | Theme 2- Job Satisfaction and Retention (factors that promote good work and retention, intrinsic, extrinsic motivation) | - Hiring in-home paid caregivers (IPCs) to tend to older adults at home has been one of the popular long-term care strategies in several urban aging societies. Relevant studies in the Global South that provide empirical findings have been lacking. - This study contributes to the literature on home care by conducting a qualitative analysis of work motivation and the quality of work life of female IPCs in Thailand. - The study found that work motivation involved the desire for personal economic improvement and the search for an emotional anchor. Analysis of quality of work life pointed to three major themes: great contribution but low recognition and support; interdependence as an ultimate career goal; and effective training as a key to opportunity and success. - The authors propose a theoretical extension to the existing care theories and suggested necessary policy interventions to sustain the supply of IPCs. |
| Boström, A. M., Lundgren, D., Kabir, Z. N., & Kåreholt, I. (2022). Factors in the psychosocial work environment of staff are associated with satisfaction with care among older persons receiving home care services. Health & social care in the community, 30(6), e6080–e6090. https://doi.org/10.1111/hsc.14045 | II- Research (Quant) | B- Home care psychosocial working conditions (Sweden) | Theme 2- Job Satisfaction and Retention (psychosocial work factors) | - Older persons in Sweden are increasingly encouraged to continue living at home and, if necessary, be supported by home care services (HCS). Studies have examined whether the work environment of staff has an impact on the experiences and well-being of older persons in residential care facilities, but few have examined such associations in HCS. - This study examined associations between home care staff's perceptions of their psychosocial work environment and satisfaction with care among older people receiving HCS. The setting was 16 HCS work units. Two surveys were conducted, one on psychosocial working conditions of staff, one on satisfaction of older persons receiving HCS. For each work unit, data on individual satisfaction were matched to average values concerning psychosocial work conditions. Outcomes analysed with linear regressions were overall satisfaction and indices regarding assessment of performance of services, contact with staff and sense of security. The index for treatment by staff was analysed with ordered logistic regressions. Cluster correlated-standard error clustering on work units was used. - Results showed that good working conditions were important for satisfaction with care, specifically overall satisfaction, treatment by staff and sense of security. The most important psychosocial work factors were work group climate, sense of mastery, job control, overall job strain, frustrated empathy, balancing competing needs, balancing emotional involvement and lack of recognition. Receiving more HCS hours was associated with stronger relationships between working conditions and satisfaction with care, especially with overall satisfaction and treatment by staff as outcomes. - The authors argue that managers and policymakers for home care need to acknowledge that the working conditions of home care staff are crucial for the satisfaction of older persons receiving HCS, particularly those receiving many HCS hours. Psychosocial work factors together with job strain factors are areas to focus on in order to improve working conditions for staff and outcomes for older persons. |
| Assander, S., Bergström, A., Olt, H., Guidetti, S., & Boström, A. M. (2022). Individual and organisational factors in the psychosocial work environment are associated with home care staffs' job strain: a Swedish cross-sectional study. *BMC health services research*, *22*(1), 1418. https://doi.org/10.1186/s12913-022-08699-4 | II- Research (Quant) | B- Job strain home care (Sweden) | Theme 2- Job Satisfaction and Retention (psychosocial work factors) | - Home care staff (HCS) provide an essential service to enable older adults to age in place. However, unreasonable demands in the work environment to deliver a safe, effective service with high quality has a negative impact on the individual employee’s well-being and the care provided to the older adults. The psychosocial work environment is associated with employees´ well-being, although, knowledge regarding which individual and organisational factors that contribute to job strain for HCS is limited. These factors need to be identified to develop targeted interventions and create sustainable work situations for HCS. - This study aimed to explore how HCS´s perceived job strain is associated with, and to what extent can be explained by, individual and organisational factors of the psychosocial work environment and psychosomatic health. An explorative cross-sectional questionnaire survey design was used in a large Swedish county. Five home care agencies with a total of 481 HCS were asked to respond to a questionnaire regarding their perceived level of job strain (Strain in Dementia Care Scale), psychosocial work environment (QPSNordic34+), and psychosomatic health (Satisfaction with Work Questionnaire). Multiple linear regression (MLR) analyses were conducted to explore the association between job strain and individual and organisational factors. - In total, 226 (46%) HCS responded to the questionnaire. Both individual and organisational factors were significant predictors of job strain and explained a variance ranging between 39 to 51%. The organisational factor, job demand and the individual factor feeling worried and restless was most frequently represented in these MRL models. A higher job strain was also associated with adverse outcomes regarding leadership, organisational culture and climate, and control at work. - This study indicates that there is an intertwined complexity of individual and organisational factors that are associated with the HCS´s perception of job strain. Implementation of new multidimensional work strategies, such as a reablement approach, could support the development of efficient strategies for HCS and reduce the level of job strain. Policy changes for the provision of home care are also needed to support the development of a sustainable and healthy psychosocial work environment. |
| Gebhard, D., Neumann, J., Wimmer, M., & Mess, F. (2022). The Second Side of the Coin-Resilience, Meaningfulness and Joyful Moments in Home Health Care Workers during the COVID-19 Pandemic. International journal of environmental research and public health, 19(7), 3836. https://doi.org/10.3390/ijerph19073836 | II- Research (Quant) | B- Home care work resilience and joyful moments (Germany) | Theme 2- Job satisfaction and Retention (Resilience and joyful moments of work) | - Nursing literature predominantly focuses on job demands but is scarce for resources related to nurses' work. In the face of the COVID-19 pandemic, resources that can buffer the health-impairing effects of increased demands gain importance. - The aim of this study was to explore resilience, meaning of work and joyful moments in home health care workers in South Germany during the pandemic. Resilience and meaning of work were measured quantitatively; moments of joy were investigated qualitatively by audio diaries and analysed with qualitative content analysis. In all, 115 home health care workers (mean age = 47.83 ± 11.72; 81.75% female) filled in the questionnaires and 237 diary entries were made by 23 persons (mean age = 46.70 ± 10.40; 91.30% female). - The mean scores of resilience (5.52 ± 1.04; 1-7) and meaning of work (4.10 ± 0.92; 1-5) showed high levels, with significantly higher values in females. - Home care workers experienced joyful moments 334 times in 60 different types in the categories of social relationships, work content, work organization, work environment and self-care. A deeper understanding of resilience, meaning of work and joyful moments provides a basis for the development of worksite health promotion programs that address both demands and resources in home health care workers. |
| Sayin, F. K., Denton, M., Brookman, C., Davies, S., Chowhan, J., & Zeytinoglu, I. U. (2021). The role of work intensification in intention to stay: A study of personal support workers in home and community care in Ontario, Canada. Economic and Industrial Democracy, 42(4), 917-936. https://doi.org/10.1177/0143831X18818325 | II- Research (Quant) | A- PSW work intensity (Canada) | Theme 2- Job Satisfaction and Retention (work intensity and intention to stay) | - This study examines the role of work intensification in personal support workers’ (PSWs) intention to stay, while exploring the mediating relationships of stress and extrinsic and intrinsic job satisfaction. A 2015 survey of 938 PSWs is analysed using structural equation modelling. - It is found that work intensification is negatively associated with intention to stay, and this relationship is mediated through stress and intrinsic job satisfaction. Extrinsic job satisfaction does not play a mediating role. Findings explain the mechanism between work intensification and intention to stay, and demonstrate work intensification as a challenge in retaining PSWs in home and community care work. |
| Julin, A. (2020). “We don’t have a choice–we need to change!” A case study on experienced efficiency and the manager role in the home care. Thesis. Lund University. Dept Political Science. https://lup.lub.lu.se/luur/download?func=downloadFile&recordOId=9009734&fileOId=9016272 | II- Research (Qual) | Care manager efficiency role and behaviours (Sweden) | Theme 2- Organisational Economic Perspectives (efficiency of organisations and care manager’s role and behaviours) | - Efficiency requirements are today found in all Swedish municipalities and regions. With the care manager in lead, the home care organisations are today expected to find efficient solutions to organisational problems. The case of the care manager is studied through a thematic analysis of interview data and recruitment announcements. - From 11 interviews with care managers and 23 care managers recruitment profiles, certain similarities and differences are found. By applying theories such as transformational leader and manager role as well as leadership behaviours on the collected material, this thesis aims at broadening knowledge on the relations between the efficiency and the manager’s role, including their tasks and the organisation of home care. - This thesis shows that the care managers are well aware of the efficiency requirements in the elderly care, however, in contrast to earlier research, they do not perceive the political decisions as demanding as their fellow managers did. Furthermore, the role of the care manager is a complex position which requires different behaviours. Consequently, the managers see a new form of home care appearing, where the main tasks of the managers are to guide the employees towards a smarter use of the resources. This shows a shift in thinking and which will have to follow in the rest of the society, too. |
| Kessler, I., Steils, N., Esser, A., & Grant, D. (2021). Understanding career development and progression from a healthcare support worker perspective. Part 1/2. British Journal of Healthcare Assistants, 15(11), 526-531. | V- Commentary | B- Career progression and development in healthcare support worker roles (England) | Theme 2- Organisational Economic Perspectives (organisational capacity to implement and fulfil training agendas) | - With access to and progression within the HCSW role seen as a means of opening-up the NHS workforce to diverse socio-economic groups often facing deep-seated labour market challenges (HEE, 2014b). More prosaically, career pathways for HCSWs into pre-registration nurse training, possibly via the nursing associate role, have been seen as a grow-your-own way of helping to address shortages in registered nurses. Alongside this is the pre-eminent objective of HCSW career development: supporting HCSWs to grow within the role itself, so enabling them to deliver high-quality care. - Such a crowded HCSW learning and development agenda generates a host of questions. These questions centre on the organisational balance to be struck in pursuing the myriad HCSW development aims outlined above, not least within the context of a training activity which extends beyond support workers to the wider NHS workforce. - Indeed, in this broader context, issues of organisational capacity to deliver on the HCSW agenda arise, alongside concerns about how to align the pursuit of different aims with the appropriate training and development programmes. For example, alongside HCSW apprenticeships designed to support progression through different job roles, other programme options are available, such as the higher development award, that is suited to HCSWs in role, who wish to undertake continuing professional development while staying in their existing role. |
| Kessler, I., Steils, N., Esser, A., & Grant, D. (2022). Understanding career development and progression from a healthcare support worker perspective. Part 2. British Journal of Healthcare Assistants, 16(1), 6-10. | V- Commentary | B- Career progression and development in healthcare support worker roles (England) | Theme 2- Organisational Economic Perspectives (organisational capacity to implement and fulfil training agendas) | - The article presents perspective and discussion on the importance of functional skills to career progression. Topics include absence of organisational funding for HCSW training contrasted with the funding of HCSWs; and feeling the COVID-19 situation greatly affecting the training opportunities because of staff shortages. |
| Tsui EK, Franzosa E, Cribbs KA, Baron S. (2019) Home Care Workers’ Experiences of Client Death and Disenfranchised Grief. *Qualitative Health Research*. 29(3):382-392. doi:10.1177/1049732318800461 | II- Research (Qual) | C- Home care worker grief (USA) | Theme 2- Personal Safety and Wellbeing (coping with client death and bereavement) | - While many types of health care workers experience patient death, home care workers do so under vastly different social and economic circumstances. When a client dies, home care workers often lose both a close relationship and a job. Though research suggests that health care workers’ grief may frequently be disenfranchised, there is no in-depth study of the mechanisms that disenfranchise home care workers’ grief specifically. - To address this gap, this study used focus groups and peer interviews between home care workers in New York City. - The authors describe four interrelated grief strategies they employ to navigate social and employer-based “grieving rules.” The findings suggest that home care workers’ grief is disenfranchised via employer and societal underestimations of their relationships with clients and their losses when clients die, particularly job loss. Building on our findings, the authors suggest alterations to agency practices and home care systems to improve support for workers. |
| Bien, E. A., Davis, K. G., Small, T. F., Reutman, S., & Gillespie, G. L. (2021). Design and development of the home healthcare worker observation tool. *Journal of nursing education and practice*, *11*(9), 29–38. https://doi.org/10.5430/jnep.v11n9p29 | II- Research (Mixed method) | B- Development of a tool to assess home care occupational risks (USA) | Theme 2- Personal Safety and Wellbeing (occupational hazards) | - The rapidly growing number of home healthcare workers (HHCW) are exposed to unique occupational hazards within each patient home. This article describes the development of an observation tool to document occupational hazards HHCWs encounter. - Tool development followed three steps: determining content domain, content validity, and inter-rater agreement. - Expert feedback guided the revision of content domain to 636 items. Scale level content validity index (S-CVI) was 0.90. Inter-rater agreement tests resulted in percent agreement and accuracy mean of 89.5% and frequency variables resulted in standard deviations from 0 to 8.62. - The observation tool encompasses the diverse range of occupational hazards HHCWs encounter; inter-rater percent agreement and overall accuracy scores were acceptable. Future pilot testing of this tool among broader raters and populations is recommended to characterize its usability, internal consistency, and reliability to assess HHCW occupational hazards. |
| Bien, E., Davis, K., & Gillespie, G. (2020). Home Healthcare Workers' Occupational Exposures. Home healthcare now, 38(5), 247–253. https://doi.org/10.1097/NHH.0000000000000891 | III- Review of literature | B- Review of occupational hazards in home care (USA) | Theme 2- Personal Safety and Wellbeing (occupational hazards) | - Home healthcare workers (HHCWs) belong to one of the fastest growing industries and have an unpredictable work environment, potentiating their risk of exposures to occupational hazards. More patients seeking care for chronic health conditions, and improvements in technology and medical advancements are allowing more complex patient care to be provided at home. - A comprehensive integrative review was completed, identifying nine articles that provide an overview of the occupational hazards HHCWs face. Analysis of the articles indicates occupational hazards are similar across studies. Occupational exposures reported by HHCWs align within all the studies and include exposures to blood, saliva, dangerous conditions walking to and within the home, second-hand smoke, aggressive pets, violence, and ergonomic concerns. These studies have been methodologically limited to self-reports, including surveys, interviews, and focus groups but include quantitative and qualitative data. - Future research can further describe and identify specific occupational exposures and health hazards, subsequently leading to modifications to protect the health and safety of HHCWs, personal care workers, and the informal caregivers who provide care in the home. |
| Yeh, I. L., Samsi, K., Vandrevala, T., & Manthorpe, J. (2019). Constituents of effective support for homecare workers providing care to people with dementia at end of life. *International journal of geriatric psychiatry*, *34*(2), 352–359. https://doi.org/10.1002/gps.5027 | II- Research (Qual) | B- Support for home care workers (England) | Theme 2- Personal Safety and Wellbeing (organisational systems for monitoring personal safety and wellbeing) | - The aim of this study was to enhance understanding about homecare workers providing care to people with dementia at the end of life by exploring homecare workers' perceptions of challenges and the support they needed and sometimes received. - Qualitative semi-structured interviews were conducted with 29 homecare workers and 13 homecare managers in England. Framework analysis was used to analyse the data. - Four overarching challenges were identified: working with clients with dementia, including clients' sometimes unpredictable responses, communication difficulties, and mood changes; caring for the dying; conflict with family members; and working alone, which often left homecare workers at risk of exhaustion, fatigue, and a sense of isolation. When their work entailed high levels of emotion, such as a client's death or getting embroiled in a client's family conflict, they felt emotionally drained, under-prepared, and overwhelmed. Supportive elements include receiving encouragement and learning from experienced peers and their feelings being acknowledged by managers at their employing homecare agency. Some workers were offered time off or encouraged to attend the client's funeral as a means of supporting the process of bereavement. - Peer and manager support are essential and effective in coping with work pressures. There is a need to develop models of effective support to alleviate staff's practical, emotional, and interpersonal pressures. However, due to the isolating nature of homecare work, managers may not recognise early signs of their staff finding stress unmanageable and miss the opportunity to mitigate these negative effects. |
| Beavis J, McKenzie S, Davis L, Ellison N. (2022) Implementation and evaluation of clinical supervision for support workers in a paediatric palliative care setting. *Clinical Child Psychology and Psychiatry*. 27(2):369-384. doi:10.1177/13591045211055565 | II- Research (Qual) | B- Supervision of support workers in paediatric palliative care (UK) | Theme 2- Personal Safety and Wellbeing (organised emotional support, clinical supervision) | - Support workers represent a large proportion of the NHS workforce and yet their supervisory needs are often overlooked. - This study focused specifically on a cohort of support workers in a community paediatric palliative care setting. Peer supervision was implemented for this group, initially face to face and then virtually. The experiences of clinical supervision for this group were investigated through responses to an online survey (n = 25) and two focus groups (n = 7). Survey data were analysed concurrently with a thematic analysis. - The following themes and sub-themes were developed from transcribed focus groups: (1) Barriers to engagement (2) Being Listened to (3) What Worked Well: Logistics. Overall, delivery of supervision was effective to a mixed degree – though support workers appreciated a space to be listened to, their distrust of colleagues and other barriers impeded the capacity of supervision to achieve more than support and catharsis for this group. The authors argue that future projects should focus on introducing more preliminary interventions to promote reflection and peer support for these groups as well as continue to consider the supervisory needs of support workers. |
| Beavis J, Davis L, McKenzie S. (2021) Clinical Supervision for Support Workers in Paediatric Palliative Care: A Literature Review. *Clinical Child Psychology and Psychiatry*. 26(1):191-206. doi:10.1177/1359104520961431 | III- Review of literature | B- Supervision for home support workers in paediatric palliative care (UK) | Theme 2- Personal Safety and Wellbeing (organised emotional support, clinical supervision) | - Providing home care to children with complex physical health needs is an emotionally challenging role. Extant literature and documents such as the Cavendish Review (2013) have reported that a large proportion of care for this population is carried out by non-registered staff (support workers). Provision of clinical supervision for nurses working in palliative care is increasing, however, supervision needs of support workers are commonly neglected. - This paper sought to synthesise what is known about clinical supervision practices for support workers in paediatric palliative care (PPC). A literature review was conducted in accordance with integrative review guidelines. 315 papers were identified initially, 15 studies were included in this review. - Four commonalities were identified: importance of team cohesion, varying degrees of formality, self-awareness and practicalities. Support workers received varying forms of supervision and some facilitators faced organisational difficulties involving staff in supervision. Support workers who received staff support generally appreciated it in recognition that their work is complex and emotionally difficult. - This paper highlights that further research should investigate the efficacy of clinical supervision as a method of reducing stress and burnout for support workers. Any implementation of supervision should involve a considered approach to training and supervision to ensure fidelity. |
| Tang, B., Mamubieke, M., Jilili, M., Liu, L., & Yang, B. (2022). Amelioration and deterioration: Social network typologies and mental health among female domestic workers in China. *Frontiers in public health*, *10*, 899322. https://doi.org/10.3389/fpubh.2022.899322 | II- Research (Quant) | B- Domestic worker’s supportive social networks  (China) | Theme 2- Personal Safety and Wellbeing (personal supportive social networks, family and friends) | - Previous quantitative studies on the effects of social network types on mental health have obtained inconsistent or conflicting results, due to problems such as sample selection bias or crude measurement of variables. - This study used statistical methodology to examine the effect of various forms of social network on the mental health of a sample of 987 Chinese female domestic workers. The study measured social network types in terms of both network attributes (friend networks and family networks) and interaction channels (face-to-face, telephone, and WeChat/QQ channels, where the latter are two popular online messaging platforms in China) and used the coarsened exact matching method to obtain a balanced sample. - The results showed that social network typologies had positive and negative effects on the mental health of this sample of domestic workers, as evidenced by (1) In terms of network attributes, family networks were associated with improved mental health and friend networks were associated with worsened mental health; (2) In terms of interaction channels, the significant amelioration in mental health from family networks came from face-to-face interactions, the significant deterioration in mental health from friends networks came from telephone interactions, and in terms of other interaction channels, family networks and friends networks had no significant effect on mental health. |
| Solis, C., Mintz, K. T., Wasserman, D., Fenton, K., & Danis, M. (2023). Home Care in America: The Urgent Challenge of Putting Ethical Care into Practice. *The Hastings Center report*, *53*(3), 25–34. https://doi.org/10.1002/hast.1487 | III- Review of ethical theory | B- Home care (USA) | Theme 2- Personal Safety and Wellbeing (quality work environment) | - Home care is one of the fastest-growing industries in the United States, providing valuable opportunities for millions of older adults and people with disabilities to live at home rather than in institutional settings. Home care workers assist clients with essential activities of daily living, but their wages and working conditions generally fail to reflect the importance of their work. - Drawing on the work of Eva Feder Kittay and other care ethicists, the authors argue that good care involves attending to the needs of another out of a concern for their well-being. Such care should be standard in the home care system. Yet, because of the pervasive racial, gender, and economic inequalities that the home care industry perpetuates, home care workers and their clients cannot reasonably be expected to care about each other. The authors endorse reforms aimed at enabling home care workers and their clients to form and maintain professional relationships that cultivate care. |
| Huang, S. F., & Liao, J. Y. (2023). Home Care Aides' Perspectives of Occupational Tobacco Smoke Exposure: A Q Methodology Study. *Nicotine & tobacco research : official journal of the Society for Research on Nicotine and Tobacco*, *25*(10), 1641–1647. https://doi.org/10.1093/ntr/ntad087 | II- Research (Quant) | B- occupational tobacco smoke exposure (Taiwan) | Theme 2- Personal Safety and Wellbeing (risks to health) | - The increased growth of older adults has generated demand for home care aides (HCAs). Occupational tobacco smoke exposure (OTSE) may risk their health which should be paid attention to. - This study explored the HCAs' perspectives of OTSE to inform health promotion programs catering to individual needs. A two-stage Q methodology was employed for data collection and analysis. Thirty-nine Q statements were extracted in the first stage and then 51 HCAs with OTSE were recruited to complete Q sorting in the second stage. PQ Method software was used for data analysis. Principal component analysis was performed to determine the most appropriate number of factors. - The five factors identified from HCAs' perspective of OTSE explained 51% of the variance. The HCAs agreed that OTSE could increase cancer risk. The HCAs with factor I did not care about OTSE, tending to complete their work. The HCAs with factor II agreed with the health hazards of OTSE but did not know how to help their clients stop smoking. The HCAs with factor III cared about OTSE but were afraid of disrupting the client-provider relationship. The HCAs with factor IV regarded OTSE as a priority for occupational interventions whereas the HCAs with factor V did not think OTSE was an issue and believed that they could balance work and OTSE health hazards. - The study findings will inform the design of home care pre-service and on-the-job training courses. Long-term care policies should be developed to promote smoke-free workplaces. There are five types of HCAs' perspectives on OTSE. The tailor interventions can be developed to help them avoid the OTSE (eg, opening windows for ventilation or using air purification equipment) and have an OTSE-free space. |
| Womack, K. N., Alvord, T. W., Trullinger-Dwyer, C. F., Rice, S. P. M., & Olson, R. (2020). Challenging Aggressive Behaviors Experienced by Personal Support Workers in Comparison to Home Care Workers: Relationships between Caregiver Experiences and Psychological Health. International journal of environmental research and public health, 17(15), 5486. https://doi.org/10.3390/ijerph17155486 | II- Research (Qual) | C- Managing aggression personal support workers (Canada) | Theme 2- Personal Safety and Wellbeing (Risks to personal safety) | - Personal support workers (PSW) are caregivers for children and adults with intellectual and developmental disabilities (IDDs) or adults experiencing mental illness or other behavioral health conditions. The work can be very meaningful, but many PSWs must prepare for, monitor, and manage challenging behaviors, including aggression. - This study was designed to estimate the prevalence of aggression experienced by PSWs in Oregon and compare it to a previous sample of Oregon home care workers (HCWs). This comparison included an analysis of relationships between exposures to aggression and psychological health factors. PSWs in Oregon (N = 240) were surveyed electronically at a single time point. - PSWs generally reported higher rates of exposure to aggression compared to HCWs. Experiences with aggression were positively associated with fatigue and weakly associated with depression, but not stress. PSWs' self-reported lost work time injury rate was elevated compared to the US average, but it was comparable to previous self-reported injury rates from HCWs. Physical demands of work were the most prevalent reported primary safety concern, followed by challenging behaviors (including aggression). Developing tailored training to help PSWs understand, plan for, minimize, and manage challenging behaviors is a social priority. |
| Byon, H. D., Lee, M., Choi, M., Sagherian, K., Crandall, M., & Lipscomb, J. (2020). Prevalence of type II workplace violence among home healthcare workers: A meta-analysis. American journal of industrial medicine, 63(5), 442–455. https://doi.org/10.1002/ajim.23095 | II- Research (Quant) | C- Workplace violence towards home healthcare workers (Korea) | Theme 2- Personal Safety and Wellbeing (risks to personal safety) | - Home healthcare workers (HHWs) provide medical and nonmedical services to home-bound patients. They are at great risk of experiencing violence perpetrated by patients (type II violence). Establishing the reliable prevalence of such violence and identifying vulnerable subgroups are essential in enhancing HHWs' safety. We, therefore, conducted meta-analyses to synthesize the evidence for prevalence and identify vulnerable subgroups. - Five electronic databases were searched for journal articles published between 1 January 2005 and 20 March 2019. A total of 21 studies were identified for this study. Meta-analyses of prevalence were conducted to obtain pooled estimates. Meta-regression was performed to compare the prevalence between professionals and paraprofessionals. - Prevalence estimates for HHWs were 0.223 for 12 months and 0.302 for over the career for combined violence types, 0.102 and 0.171, respectively, for physical violence, and 0.364 and 0.418, respectively, for nonphysical violence. The prevalence of nonphysical violence was higher than that of physical violence for professionals in 12 months (0.515 vs 0.135) and over the career (0.498 vs 0.224) and for paraprofessionals in 12 months (0.248 vs 0.086) and over the career (0.349 vs 0.113). Professionals reported significantly higher nonphysical violence for 12-month prevalence than paraprofessionals did (0.515 vs 0.248, P = .015). - A considerable percentage of HHWs experience type II violence with higher prevalence among professionals. Further studies need to explore factors that can explain the differences in the prevalence between professionals and paraprofessionals. - The findings provide support for the need for greater recognition of the violence hazard in the home healthcare workplace. |
| Phoo, N. N. N., & Reid, A. (2022). Determinants of violence towards care workers working in the home setting: A systematic review. American journal of industrial medicine, 65(6), 447–467. https://doi.org/10.1002/ajim.23351 | III- Review of literature | B- Violence towards HSWs (USA) | Theme 2- Personal Safety and Wellbeing (risks to personal safety) | - Home care is a rapidly growing industry. Violence towards home care workers is common, while also likely underreported. This violence adversely affects the physical and mental health of both workers and care recipients. - This review of the literature aimed to identify and appraise recent evidence on the determinants of violence towards care workers working in the home setting. Six electronic databases: the Cumulative Index to Nursing and Allied Health Literature (CINAHL), EMBASE, Informit, Medline, PsycINFO, and Web of Science, were systematically searched. A systematic review was conducted in accordance with the Joanna Briggs Institute manual for evidence synthesis. - A total of 18 papers met the inclusion criteria. All were cross-sectional surveys. Most studies were from the United States. The most investigated associations were those between the medical history of clients, workers' apprehension of violence, worker-client relationship, or care plans, and any form of violence or verbal abuse. - Violence was common in clients with cognitive disorders, substance abuse disorder, and limited mobility; toward workers who feared that violence might happen; toward those who had very close or very distant worker-client relationships; and when care plans were not inclusive of clients' needs. The current review highlights a gap in evidence on determinants of violence towards care workers working in the home setting as well as suggesting potential areas where research could reduce such violence. |
| Muramatsu, N., Sokas, R. K., Lukyanova, V. V., & Zanoni, J. (2019). Perceived Stress and Health among Home Care Aides: Caring for Older Clients in a Medicaid-Funded Home Care Program. Journal of health care for the poor and underserved, 30(2), 721–738. https://doi.org/10.1353/hpu.2019.0052 | II- Research (Qual) | B- Home Care Aide’s health and wellbeing (USA) | Theme 2- Personal Safety and Wellbeing (stress, burnout, emotional labour) | - Home care aides (HCAs) provide housekeeping and personal care services to help older clients remain in the community. However, little is known about the health of HCAs, who themselves constitute an underserved population. - The goal of this study was to investigate how HCAs' work and life contexts manifest themselves in HCAs' health as perceived by HCAs. Six focus groups were conducted with HCAs (N=45). - Analysis revealed how HCAs' work-and life-related stress accumulated over time and affected HCAs' health and interaction with their older clients. Home care aides were interested in personal health promotion and client well-being. Home care aides may constitute an underused resource for the care of older adults with disabilities. Information about intricately intertwined work and life contexts should inform policymakers and home care providers in their efforts to improve the quality of publicly funded home care services. |
| Gebhard, D., & Wimmer, M. (2023). The Hidden Script of Work-Related Burdens in Home Care - A Cross Over Mixed Analysis of Audio Diaries. Journal of applied gerontology : the official journal of the Southern Gerontological Society, 42(4), 704–716. https://doi.org/10.1177/07334648221130747 | II- Research (Qual) | B-Work related burden (Germany) | Theme 2- Personal Safety and Wellbeing (Work related burden) | - The lived experiences of work-related burdens in the daily working routines of home care workers are insufficiently investigated. Therefore, the aim of this study was to examine the types, frequencies, and distributions of work-related burdens and to explore their co-occurrence. - Qualitative data was collected via audio diaries and analyzed applying a crossover mixed analysis using content as well as network analysis. In all, 23 home care workers (mean age = 46.70 ± 10.40; 91.30% female) produced 242 diary entries. - Participants reported work-related burdens 580 times with 77 different types, predominately in relation to work organization (50.5%). Network analysis reveals a complex picture, which shows the strong relation between time pressure and travel between homes and identifies additional tasks as the central node in the network of burdens. A holistic understanding of setting-specific burdens provides an important starting point for measures of workplace health promotion. |
| Muramatsu, N., Yin, L., & Lin, T. T. (2017). Building Health Promotion into the Job of Home Care Aides: Transformation of the Workplace Health Environment. *International journal of environmental research and public health*, *14*(4), 384. https://doi.org/10.3390/ijerph14040384 | II- Research (Mixed method) | B- Home Care Aide’s physical activity programme (USA) | Theme 2- Personal Safety and Wellbeing (workplace health promotion interventions) | - Home care aides (HCAs), predominantly women, constitute one of the fastest growing occupations in the United States. HCAs work in clients' homes that lack typical workplace resources and benefits. - This mixed-methods study examined how HCAs' work environment was transformed by a pilot workplace health promotion program that targeted clients as well as workers. The intervention started with training HCAs to deliver a gentle physical activity program to their older clients in a Medicaid-funded home care program. - Older HCAs aged 50+ reported increased time doing the types of physical activity that they delivered to their clients (stretching or strengthening exercise). Almost all (98%) HCAs were satisfied with the program. These quantitative results were corroborated by qualitative data from open-ended survey questions and focus groups. HCAs described how they exercised with clients and how the psychosocial work environment changed with the program. Building physical activity into HCAs' job is feasible and can effectively promote HCAs' health, especially among older HCAs. |
| Wilberforce, M., Abendstern, M., Tucker, S., Ahmed, S., Jasper, R., & Challis, D. (2017). Support workers in community mental health teams for older people: roles, boundaries, supervision and training. *Journal of advanced nursing*, *73*(7), 1657–1666. https://doi.org/10.1111/jan.13264 | II- Research (Qual) | B- Support workers in community mental health teams for older adults (England) | Theme 2- Role Clarity, Preventing Role Drift and Having Clear Boundaries (role drift into unagreed, unsafe, or unlawful practices) | - Explores the support worker functions in community mental health teams for older adults in relation to roles, boundaries, supervision and training. Support workers in community mental health teams provide important help to older people with complex mental and physical health needs in their own homes. Their numbers have grown substantially in recent years, but without professional registration there is concern that boundaries with qualified practitioners are insufficiently clear and that they do not receive the support they require. - Qualitative research using interview data and thematic framework analysis investigated support workers' and registered practitioners' perspectives on roles, boundaries, supervision and training. Semi-structured face-to-face interviews were undertaken in 2011, with 42 members of nine teams spread across England, including support workers and community mental health nurses. - Findings show that support workers undertook diverse roles and had considerable autonomy over their duties. Participants agreed about what tasks support workers should not undertake, yet there was evidence of 'negotiated' boundaries and examples of these being breached. Lines of authority were complex, yet support workers were supported through open communication with the wider team. Training was problematic, with few courses tailored for support workers and efforts towards formal qualification hindered by low pay and time pressures. - Argues that local and national attention is needed to prevent 'drift' into activities that both support workers and registered practitioners consider outside their remit. Barriers to training and further qualification need to be addressed. |
| Turner, N., Schneider, J., Pollock, K., Travers, C., Perry-Young, L., & Wilkinson, S. (2020). 'Going the extra mile' for older people with dementia: Exploring the voluntary labour of homecare workers. *Dementia (London, England)*, *19*(7), 2220–2233. https://doi.org/10.1177/1471301218817616 | II- Research (Qual) | B- Working conditions in home care (England) | Theme 2- Role Clarity, Preventing Role Drift and Having Clear Boundaries (voluntary labour, working beyond paid hours) | - Homecare workers provide essential physical, social and emotional support to growing numbers of older people with dementia in the UK. Although it is acknowledged that the work can sometimes be demanding, some homecare workers regularly 'go the extra mile' for service users, working above and beyond the usual remit of the job. This form of voluntarism has been interpreted as an expression of an essentially caring nature, but also as the product of a work environment structured to tacitly endorse the provision of unpaid labour. - This paper draws on a qualitative study of what constitutes 'good' homecare for older people with dementia. Using homecare workers' reflexive diaries (n = 11) and interviews with homecare workers (n = 14) and managers (n = 6), explored manifestations of, and motivations for, homecare workers going the extra mile in their everyday work. - Describes three modes of voluntary labour based on these accounts which are characterised as affective, performative and pragmatic. The study highlights the complex relationships between job satisfaction, social benefit and commercial gain in the homecare work sector. Further research is needed to define the full range of affective and technical skills necessary to deliver good homecare, and to ensure that homecare work is appropriately credited. |
| Torres, J. M., Kietzman, K. G., & Wallace, S. P. (2015). Walking the Line: Navigating Market and Gift Economies of Care in a Consumer-Directed Home-Based Care Program for Older Adults. *The Milbank quarterly*, *93*(4), 732–760. https://doi.org/10.1111/1468-0009.12163 | II- Research (Qual) | B- Pay and unpaid work (USA) | Theme 2- Role Clarity, Preventing Role Drift and Having Clear Boundaries (voluntary labour, ‘gifted’ hours, working beyond paid hours) | - Paid caregivers of low-income older adults navigate their role at what Hochschild calls the "market frontier": the fuzzy line between the "world of the market," in which services are exchanged for monetary compensation, and the "world of the gift," in which caregiving is uncompensated and motivated by emotional attachment. This study examined how political and economic forces, including the reduction of long-term services and supports, shape the practice of "walking the line" among caregivers of older adults. - This study used data from a longitudinal qualitative study with related and nonrelated caregivers (n = 33) paid through California's In-Home Supportive Services (IHSS) program and consumers of IHSS care (n = 49). Semi-structured interviews (n = 330) were completed between 2010 and 2014, were analysed using a constructivist grounded theory approach. - Related and nonrelated caregivers are often expected to "gift" hours of care above and beyond what is compensated by formal services. Cuts in formal services and lapses in pay push caregivers to further "walk the line" between market and gift economies of care. Both related and nonrelated caregivers who choose to stay on and provide more care without pay often face adverse economic and health consequences. Some, including related caregivers, opt out of caregiving altogether. While some consumers expect that caregivers would be willing to "walk the line" in order to meet their needs, most expressed sympathy for them and tried to alter their schedules or go without care in order to limit the caregivers' burden. - Given economic and health constraints, caregivers cannot always compensate for cuts in formal supports by providing uncompensated time and resources. Similarly, low-income older adults are not competitive in the caregiving marketplace and, given the inadequacy of compensated hours, often depend on unpaid care. Policies that restrict formal long-term services and supports thus leave the needs of both caregivers and consumers unmet. |
| Karlsson, N. D., Markkanen, P. K., Kriebel, D., Galligan, C. J., & Quinn, M. M. (2020). "That's not my job": A mixed methods study of challenging client behaviors, boundaries, and home care aide occupational safety and health. *American journal of industrial medicine*, *63*(4), 368–378. https://doi.org/10.1002/ajim.23082 | II- Research (Quant) | B- Home care aides (USA) | Theme 2- Role Clarity, Preventing role drift Boundaries and Delegation (role uncertainty, role boundary-challenging behaviours) | - Home care (HC) aide is among the fastest-growing jobs. Aides often work in long-term care relationships with elders or people with disabilities in clients' homes, assisting with daily activities. The purpose of this mixed-methods paper is to elucidate aides' experiences around the boundary-challenging behaviors of clients asking for services beyond aides' job duties and to identify possible interventions. - A cross-sectional survey of HC aides in Massachusetts (n = 1249) provided quantitative data. Post-survey qualitative data were collected from nine HC aide focus groups (n = 70) and seven in-depth interviews with HC industry and workforce representatives. - Quantitatively, aides who reported often being asked to do tasks outside their job duties were more likely to report abuse (prevalence ratio [PR] = 1.93, for verbal, PR = 1.81, for physical/sexual) and pain/injury with lost work time or medical care (PR = 1.58). They were also less likely to want to remain in their job (PR = 0.94) or recommend it to others (PR = 0.94). Qualitative data showed that clients' requests for tasks beyond job duties were frequent and can lead to injuries, abuse, and psychosocial stress. Yet, requests often reflected genuine need. - Helping clients stay at home, compassion, and feeling appreciated contributed to job satisfaction; therefore, aides can feel conflicted about refusing requests. Client task requests outside HC services are a complex problem. Employer support, training, care plans, and feeling part of a care team can help aides navigate professional boundaries while delivering high quality care. |
| Koru, G., Alhuwail, D., Jademi, O., Uchidiuno, U., & Rosati, R. J. (2018). Technology Innovations for Better Fall Risk Management in Home Care. *Journal of gerontological nursing*, *44*(7), 15–20. https://doi.org/10.3928/00989134-20180412-01 | II- Research (Document analysis) | A- Home care falls prevention (USA) | Theme 2- Safety in the Home Setting (falls prevention) | - Achieving better fall risk management is an integral component of quality home care. - This qualitative study uncovers the challenges and opportunities of home health agencies (HHAs) in achieving better fall risk management. A secondary document analysis was adopted to learn from rich contextual information in fall incident reports recorded in a HHA. - Poor engagement of patients and caregivers was a contributing factor in many fall incidents. Patients often fell as a result of not understanding or accepting their physical limitations. For better fall risk management, many incidents highlighted a need for providing complete and thorough care, better coordination of care, higher levels of sociocultural awareness, patient engagement, and caregiver involvement. The results provide evidence regarding the challenges and opportunities for improving fall risk management in home care along with insight about how information technology solutions can support improvement initiatives. |
| Brouillette, N. M., Markkanen, P. K., Quinn, M. M., Galligan, C. J., Sama, S. R., Lindberg, J. E., & Karlsson, N. D. (2023). Aide and Client Safety "Should Go Hand-In-Hand": Qualitative Findings From Home Care Aides, Clients, and Agency Leaders. Journal of applied gerontology : the official journal of the Southern Gerontological Society, 42(4), 571–580. https://doi.org/10.1177/07334648221146769 | II- Research (Qual) | B- Home care aides (USA) | Theme 2- Safety in the Home Setting (home layout, accessibility, and home safety risks) | - Retention of the home care (HC) aide workforce is essential to meet the needs of our aging population. Some studies suggest that improving HC safety could increase job retention. - This study explores qualitatively the connection between aide and client safety and factors impacting this care relationship. Data consisted of audio-recorded, *verbatim* responses to open-ended questions of two focus groups with aides (*n* = 10), two in-person interviews with HC agency managers, and 37 phone interviews with those working in (aides, *n* = 16; managers, *n* = 12) and receiving (clients, *n* = 9) HC. - Clients reported home layout and accessibility as safety concerns. Aides and managers reported that client family members can make the care job more challenging. The aide-client connection was affected by communication style, family and HC agency support, allotted care time, and job task boundaries. Interventions that address the safety of both clients and aides can influence HC job satisfaction and retention. |
| Schoenfisch, A. L., Lipscomb, H., & Phillips, L. E. (2017). Safety of union home care aides in Washington State. *American journal of industrial medicine*, *60*(9), 798–810. https://doi.org/10.1002/ajim.22747 | II- Research (Mixed method) | B- Occupational injury risks for Home Care Aides (USA) | Theme 2- Safety in the Home Setting (organisational systems and support structures for safety in homes) | - A rate-based understanding of home care aides' adverse occupational outcomes related to their work location and care tasks is lacking. Within a 30-month, dynamic cohort of 43 394 home care aides in Washington State, injury rates were calculated by aides' demographic and work characteristics. Injury narratives and focus groups provided contextual detail. - Injury rates were higher for home care aides categorized as female, white, 50 to <65 years old, less experienced, with a primary language of English, and working through an agency (versus individual providers). In addition to direct occupational hazards, variability in workload, income, and supervisory/social support is of concern. Policies should address the roles and training of home care aides, consumers, and managers/supervisors. Home care aides' improved access to often-existing resources to identify, manage, and eliminate occupational hazards is called for to prevent injuries and address concerns related to the vulnerability of this needed workforce. |
| Ohta, R., Ryu, Y., & Katsube, T. (2020). Home care workers' judgments about users' acute conditions: A qualitative study on interprofessional collaboration. *Home health care services quarterly*, *39*(3), 184–195. https://doi.org/10.1080/01621424.2020.1736228 | II- Research (Qual) | B- Home care worker’s approaches to acute symptoms (Japan) | Theme 2- Safety in the Home Setting (organisational systems and support structures for emergency situations) | - Home care workers' (HCWs) approaches to home care users' acute symptoms are critical for users' safety and quality of life. However, the processes of these approaches are unclear. - This study investigates how HCWs assess users' conditions. Focus group discussions and semi-structured interviews with HCWs were conducted in a rural Japanese city. - HCWs' decisions were affected by interactions and previous relationships with care managers, home care nurses, physicians, and users' families. Rural HCWs act flexibly, changing the professionals and families they consult with. Understanding HCWs' behaviors and improving relationships among medical/care professionals and families can improve management of users' acute conditions. |
| Swedberg, L., Chiriac, E. H., Törnkvist, L., & Hylander, I. (2013). From risky to safer home care: health care assistants striving to overcome a lack of training, supervision, and support. *International journal of qualitative studies on health and well-being*, *8*, 20758. https://doi.org/10.3402/qhw.v8i0.20758 | II- Research (Qual) | B- Home carers providing health care (Sweden) | Theme 2- Safety in the Home Setting (organisational systems and support structures for safety in homes) | - In Sweden, 24-hour home care is often carried out by municipality-employed paraprofessionals such as health care assistants (HC assistants) with limited or no health care training, performing advanced care without formal training or support. The aim of this study was to investigate the work experience of the HC assistants and to explore how they manage when delivering 24-h home care to patients with substantial care needs. - Grounded theory methodology involving multiple data sources comprising interviews with HC assistants (n=19) and field observations in patients' homes was used to collect data and constant comparative analysis was used for analysis. - The initial analysis revealed a number of barriers, competence gap; trapped in the home setting; poor supervision and unconnected to the patient care system, describing the risks associated with the situations of HC assistants working in home care, thus affecting their working conditions as well as the patient care. - The core process identified was the HC assistants' strivings to combine safe home care with good working conditions by using compensatory processes. The four identified compensatory processes were: day-by-day learning; balancing relations with the patient; self-managing; and navigating the patient care system. By actively employing the compensatory processes, the HC assistants could be said to adopt an inclusive approach, by compensating for their own barriers as well as those of their colleagues' and taking overall responsibility for their workplace. - Argues for the importance of supporting HC assistants in relation to their needs for training, supervision, and support from health care professionals must be addressed when organising 24-h home care to patients with substantial care needs in the future. |
| Backhouse, T., Ruston, A., Killett, A., Ward, R., Rose-Hunt, J., & Mioshi, E. (2022). Risks and risk mitigation in homecare for people with dementia-A two-sided matter: A systematic review. *Health & social care in the community*, *30*(6), 2037–2056. https://doi.org/10.1111/hsc.13865 | III- Review of literature | B- Risks in dementia home care (England) | Theme 2- Safety in the Home Setting (organisational systems and support structures for safety in homes) | - Policy guidance promotes supporting people to live in their own homes for as long as possible with support from homecare services. People living with dementia who need such support can experience a range of physical and cognitive difficulties, which can increase the risks associated with homecare for this group. - This study aimed to examine risk and safety issues for people with dementia and their homecare workers and risk mitigation practices adopted by homecare workers to address identified risks. The review included searches of MEDLINE, EMBASE, AMED, CINAHL, PsycINFO, ASSIA and Cochrane Central Register of Controlled Trials databases 5 March 2021. Included studies focused on homecare for people with dementia and had a risk or safety feature reported. Risk of bias was assessed with the Joanna Briggs Institute Critical Appraisal tools. Two authors assessed articles for potential eligibility and quality. A narrative synthesis combines the findings. - The search identified 2259 records; 27 articles, relating to 21 studies, met the eligibility criteria. The review identified first-order risks that homecare workers in the studies sought to address. Two types of risk mitigation actions were reported: harmful interventions and beneficial interventions. Actions adopted to reduce risks produced intended benefits but also unintended consequences, creating second-order risks to both clients with dementia and homecare workers, placing them at greater risk. - The authors conclude that risk mitigation interventions should be person-centred, the responsibility of all relevant professions, and planned to minimise the creation of unintended risks. |
| Small, T. (2020) Occupational Hazards in Home Care. Home Healthcare Now 38(4):p 221. doi: 10.1097/NHH.0000000000000895 | V- Commentary | B- Occupational health risks in home settings (USA) | Theme 2- Safety in the Home Setting (organisational systems and support structures for safety in homes) | - Woking in home care involves unique risks. Unlike healthcare professionals working in institutional settings, those working in home care have unpredictable and uncontrolled work environments along with frequent commutes to people’s homes throughout the day. The ‘worksite’ is the person’s home which is unregulated and beyond the control of the employer or worker. - Risks include exposure to violence in the community or home, unrestrained animals, pests (most commonly bed bugs, rats and roaches), unhygienic conditions, poor indoor air quality, and driving related accidents. - Some strategies for mitigating risks include performing a community and home risk assessment prior to the first home visit, employe training on safe driving and workplace violence prevention, and implementation of nonsmoking policies while working in the home. |
| Kurata, S., & Ojima, T. (2014). Knowledge, perceptions, and experiences of family caregivers and home care providers of physical restraint use with home-dwelling elders: a cross-sectional study in Japan. *BMC geriatrics*, *14*, 1-11. | II- Research (Qual) | B- Family caregiver use of physical restraint in home care (Japan) | Theme 2- Safety in the Home Setting (physical restraints) | - The use of physical restraints by family caregivers with home-dwelling elders has not been extensively studied but it might be widespread. Furthermore, it is also not clear how home care providers who support family caregivers perceive the use of physical restraint in elders' homes. This study assessed family caregivers' and home care providers' knowledge and perceptions of physical restraint used with elders living at home in Japan, a country with the highest proportion of elders in the world and where family caregiving is common. - A cross-sectional study of 494 family caregivers, 201 home helpers, 78 visiting nurses, 131 visiting physicians, and 158 care managers of home-dwelling frail elders needing some care and medical support in Japan, using questionnaires on knowledge of 11 physical restraint procedures prohibited in institutions and 10 harmful effects of physical restraints, perceptions of 17 reasons for requiring physical restraints, and experiences involving physical restraint use. - Family caregivers were aware of significantly fewer recognized prohibited physical restraint procedures and recognized harmful effects of physical restraint than home care providers, and differences among home care providers were significant. The average importance rating from 1 (least) to 5 (most) of the 17 reasons for requiring physical restraints was significantly higher among family caregivers than home care providers, and significantly different among the home care providers. Moreover, these differences depended in part on participation in physical restraint education classes. While 20.1% of family caregivers had wavered over using physical restraints, 40.5% of home care providers had seen physical restraints used in elders' homes and 16.7% had advised physical restraint use or used physical restraints themselves. - Knowledge and perceptions of physical restraints differed between family caregivers and home care providers and were also diverse among home care providers. Because both groups might be involved in physical restraint use with home-dwelling elders, home care providers should acquire standardized and appropriate knowledge and perceptions of physical restraints to help family caregivers minimize abusive physical restraint use. |
| Malhotra, R., Arambepola, C., Tarun, S., de Silva, V., Kishore, J., & Østbye, T. (2013). Health issues of female foreign domestic workers: a systematic review of the scientific and gray literature. International journal of occupational and environmental health, 19(4), 261–277. https://doi.org/10.1179/2049396713Y.0000000041 | III- Review of Literature | B- Health issues of female migrant workers (worldwide) | Theme 3- Careers of Migrant Workers (health issues for migrant workers) | - Although the number of female foreign domestic workers (FDWs) is increasing worldwide, little is known about their health issues. - This study aimed to systematically review the literature on health issues of female FDWs to ascertain the problems studied, identify limitations, and suggest future research and policy implications. - A systematic database (PubMed, EBSCO Host, and Google Scholar) and bibliographic search identified the English-language scientific and gray literature published during 1990-2012 addressing health issues of female FDWs living with the family of the employer, using qualitative and/or quantitative research methods. Studies in which female FDWs constituted less than half of the participants were excluded. - The health issues studied and identified were adverse work conditions and associated health problems (such as physical, verbal, and sexual abuse at the workplace, caregiving tasks associated with musculoskeletal strain, and chemical exposure associated with respiratory difficulty), mental health (psychotic, neurotic, and mood disorders), infectious diseases (most of the studies were on intestinal parasitic infections), and health knowledge/attitudes/practices (most of the studies were in context of sexual and reproductive health). Most of the studies were medical record reviews or questionnaire-based surveys utilizing convenience sampling or qualitative interviews/focus group discussions. - Female FDWs face numerous health problems. Studies on representative, possibly longitudinal, samples of female FDWs focusing on specific health conditions are needed to better understand the epidemiology of such conditions. Concerted efforts through the governments of both labor-sending and host countries are required to improve the health, work conditions, and safety of this vulnerable group of women. |
| Yuan, Q., Zhang, Y., Samari, E., Jeyagurunathan, A., Tan, G. T. H., Devi, F., Wang, P., Magadi, H., Goveas, R., Ng, L. L., & Subramaniam, M. (2022). The impact of having foreign domestic workers on informal caregivers of persons with dementia - findings from a multi-method research in Singapore. *BMC geriatrics*, *22*(1), 305. https://doi.org/10.1186/s12877-022-03002-w | II- Research (Mixed method) | B- Impact of migrant domestic workers on caregivers (Singapore) | Theme 3- Careers of Migrant Workers (impact of migrant workers on caregivers) | - Informal caregivers of persons with dementia (PWDs) sometimes engage foreign domestic workers (FDWs) to support their caregiving journey. However, there has not been much research to establish if this is really beneficial. The current study aims to investigate whether engaging FDWs specifically for caregiving of PWDs truly moderates caregiver stress and to explore caregivers' experiences of engaging FDWs. - A multi-method study design with a quantitative and qualitative sub-study was adopted. For the quantitative sub-study, 282 informal caregivers of PWDs were recruited. Propensity score matching analysis was used. For the qualitative sub-study, 15 informal caregivers with FDWs were interviewed. Inductive thematic analysis was conducted. - The quantitative sub-study confirmed that engaging FDWs did moderate the depressive symptoms of informal dementia caregivers (marginal effect = -3.35, p = 0.0497). However, such support did not affect their caregiving burden, self-efficacy, and perceived positive aspects of caregiving. The qualitative sub-study suggested that engaging FDWs is an ambivalent experience, which entails both support and challenges. - This study confirmed previous research findings, that engaging FDWs moderated depressive symptoms among caregivers of PWDs, and it could be through their physical support such as in daily caregiving activities. Policymakers may consider providing more subsidies to caregivers caring for PWDs with mobility issues to hire FDWs. They may also consider providing training to FDWs on dementia caregiving skills and improving the intake of such training as this might be helpful for both FDWs and caregivers during this journey. |
| Gallotti M,. (2015) Migrant Domestic Workers Across the World. Geneva, Switzerland, International Labour Organization. (Based on the ILO Report on Global Estimates on Migrant Workers. https://associazionedomina.it/wp-content/uploads/2017/05/Migrant-Domestic-Workers-Across-the-World.pdf | II- Research (Quant) | B- Global estimates of migrant domestic workers (worldwide) | Theme 3- Careers of Migrant Workers (reliance on migrant workers) | - The ILO report on Global Estimates on Migrant Workers, 2015 unveils the statistical importance of domestic work as a source of employment for millions of migrant workers worldwide and provides regional and global estimates of the share of domestic workers (DWs) among migrants and the share of migrants among domestic workers. - The report shows that there are 11.5 million migrant domestic workers in the world (2015). This represents 17.2 per cent of a total estimate of 67.1 million domestic workers globally. This number indicates that the growing needs for personal and household services in many parts of the world are filled by migrant workers. - MDWs are concentrated in high income countries. Together, the Arab states, North America and northern, southern and Western Europe account for about 52 per cent of the 11.5 million domestic workers worldwide. - Demand for domestic and care services has increased over the last three decades in northern, southern and Western Europe, as has the presence of migrants in these services. This represents a significant sector of migrant employment especially for women. With more than 2 of the 4 million domestic workers in the region being migrant workers, the region has the highest percentage of migrants in the sector after the Arab States and North America. |
| Ow Yong, B., & Manthorpe, J. (2016). The experiences of Indian migrant care home staff working with people with dementia: a pilot study exploring cultural perspectives. Working with Older People, 20(1), 3-13. | II- Research (Qual) | B- Migrant Indian care workers in long-term care facilities (England) | Theme 3- Careers of Migrant Workers (support and career opportunities for migrant workers) | - Little is known about migrant Indian care workers working in long-term care facilities for people with dementia in England and the purpose of this paper is to remedy this lack of information in the light of political interest in immigration to the UK and continued staff shortages in parts of the social care sector. - This pilot study investigated the experiences of workplace acculturation among 12 migrant Indian care workers who were employed in English care homes. Qualitative face-to-face interviews were conducted in 2013. Analysis of the interviews was conducted using principles of interpretative phenomenological analysis. - Following analysis five themes emerged along an acculturation timeline. First, during the first six months of their employment, the migrant care workers recalled feeling vulnerable, seemingly marked by a sense of insecurity and an overwhelming state of cognitive burden within an unfamiliar cultural context. Second, simultaneously, the migrants felt perturbed about their new role as direct care workers. Third, few had been able to draw on their networks of friends and relatives to build up knowledge of their new work environments before starting care home employment. Fourth, two years into the work, although they reported feeling better adapted, psychological and socio-cultural adjustments were still thought to be needed. Fifth, most participants retained their ambition to be recognised as a qualified nurse in the UK and to pursue a nursing career outside the social care sector. - This is a pilot study in which 12 migrant Indian care home workers were interviewed. Further interviews might provide a greater range of views and experiences. The care homes that participated in this research were in the London region where staff shortages are common in dementia services such as care homes. - The findings suggest a need for employers and human resource managers to respond to the specific needs of Indian and other migrants working with older people who are resident in care homes. Such responses should reflect the timeline of their acculturation and employers need also to acknowledge and address aspirations to move on to NHS work. - This study is unique to the best of the authors’ knowledge in addressing Indian care workers specifically as a substantial part of the migrant care workforce in the UK. It offers information about their perceptions and suggests practical human response and managerial initiatives. |
| Tam, W. J., Koh, G. C., Legido-Quigley, H., Ha, N. H. L., & Yap, P. L. K. (2018). "I Can't Do This Alone": a study on foreign domestic workers providing long-term care for frail seniors at home. *International psychogeriatrics*, *30*(9), 1269–1277. https://doi.org/10.1017/S1041610217002459 | II- Research (Qual) | B- Support for MDWs (Singapore) | Theme 3- Careers of Migrant Workers (support for migrant workers) | - Foreign domestic workers (FDWs) play an important role in long-term caregiving of seniors at home. However, how FDWs cope with the caregiving demands, the dynamic interaction between familial and FDW caregivers and its impact on care recipients remain largely un-explored. Existing caregiver interventions mainly target familial caregivers; little assistance is available for FDW caregivers. This study explores FDWs' challenges, coping strategies, and the support they need in caring for seniors. - FDWs were recruited from a geriatric ward and outpatient clinic of a tertiary hospital in Singapore. Qualitative in-depth interviews were conducted with 25 FDWs caring for frail seniors and five healthcare staff. Interviews were transcribed and analyzed using thematic analysis. - FDWs were from Indonesia, Philippines, and Myanmar. Nineteen cared for seniors with dementia (SWDs). The analysis derived six subthemes, clustered into three salient themes: two described social support to FDWs by the senior's family members, two described their coping strategies, and two described their job satisfaction. Those who cared for SWDs faced more difficulties. It derived two family models of care: FDW-centered family dynamics, where family members rely on FDWs to perform most duties, causing poor impact on seniors' well-being and team-based family dynamics, where family members and FDWs share the caregiving burden, resulting in better impact on seniors' well-being. - FDWs face significant challenges in eldercare. Improving FDWs' access to training courses in eldercare, providing them with more emotional support, engaging employers to create healthy caregiving spaces at home, and improving access to senior care services can be helpful. |
| Ha, N. H. L., Chong, M. S., Choo, R. W. M., Tam, W. J., & Yap, P. L. K. (2018). Caregiving burden in foreign domestic workers caring for frail older adults in Singapore. *International psychogeriatrics*, *30*(8), 1139–1147. https://doi.org/10.1017/S1041610218000200 | II- Research (Quant) | B- MDW carer burden (Singapore) | Theme 3- Careers of Migrant Workers (support for migrant workers) | - Although foreign domestic workers (FDWs) play a significant role in caring for frail seniors in Singapore and are vulnerable to caregiving burden, there has been little research conducted hitherto. This study explored caregiver burden and its determinants. - FDWs (N = 221, Mage = 32.3, SD = 6.23) recruited from a hospital geriatric unit completed the Zarit Burden Interview (ZBI) administered in English, Bahasa Melayu, or Burmese. Univariate and multivariate regression were employed to investigate factors influencing caregiving burden in FDWs. - Majority were Indonesians (60.0%), married (57.5%) with children (62.4%), with secondary-level education (59.7%), and providing care for >1 year (79.9%). Importantly, 25.1% reported physical health problems and 23.1% encountered language difficulties with employers. - Univariate analysis revealed three significant factors associated with caregiving burden: nationality, lack of privacy, and caring for persons with dementia (PWD). On multivariate regression, FDWs who cared for PWD were 5.47 times (p = 0.013) more likely to experience burden, while FDWs who encountered language difficulties were 5.46 times more likely to experience burden. Filipinos FDWs were 9.73 times more likely to express burden compared to their Indonesian and Burmese counterparts. - The study highlights caregiver burden in FDWs and potential ways to alleviate it by empowering FDWs with dementia-specific caregiving skills, providing language training opportunities, and supporting particular FDW ethnic groups with more emotional and practical help. |
| Eltaybani, S., Kitamura, S., Fukui, C., Igarashi, A., Sakka, M., Noguchi-Watanabe, M., Takaoka, M., Inagaki, A., Yasaka, T., Kobayashi, H., & Yamamoto-Mitani, N. (2023). Toward developing care outcome quality indicators for home care for older people: A prospective cohort study in Japan. *Geriatrics & gerontology international*, *23*(5), 383–394. https://doi.org/10.1111/ggi.14578 | II- Research (Mixed method) | B- Quality indicators for home care (Japan) | Theme 3- Families and Caregivers (differences in the impact of paid carers on family caregivers) | - Care quality in Japan's long-term care (LTC) agencies, including home care, is the responsibility primarily of individual agencies, and the evaluation of service processes and outcomes is minimal. This article describes the development of quality indicators for LTC (QIs-LTC) in Japan. - QIs-LTC were developed through literature review and expert panel discussions and then were piloted and used in a 2-year longitudinal survey. The survey (launched in September 2019) targeted older people receiving home care (n = 1450), their family members (n = 880), their professional home care providers (n = 577), and managers of home care agencies (n = 122). - Across eight domains (maintaining dignity, minimizing symptoms and disease deterioration, maintaining nutritional status, maintaining bladder/bowel control, encouraging physical activities, experiencing sound sleep, maintaining serenity and contentedness, and maintaining family's well-being), 24 care quality objectives were set with 24 outcome QIs-LTC and 144 process QIs-LTC. In the survey, 84.8% of clients were using home care nursing, 26.3% were living alone, and 39.5% had dementia. In the month preceding the data collection, 13.9% of clients had a new disease or worsening of an existing disease, 8.8% were hospitalized at least once, and 47.9% did not participate in activities of interest. About 20% of clients' families were unable to spend time peacefully, and 52.8% were exhausted from the client's care. - The QIs-LTC developed in the current study are generic and client- and family-centered. They encompass objective and subjective information and would facilitate standardized monitoring if adopted and comparison between LTC settings, including home care. |
| Kim, E. Y., & Yeom, H. E. (2016). Influence of home care services on caregivers' burden and satisfaction. *Journal of clinical nursing*, *25*(11-12), 1683–1692. https://doi.org/10.1111/jocn.13188 | II- Research (Quant) | B- Impact of home care on caregivers (South Korea) | Theme 3- Families and Caregivers (differences in the impact of paid carers on family caregivers) | - This study aimed to examine the factors affecting the burden and satisfaction of family caregivers, focusing on the beneficial impacts of home care service use. - Long-term care for older patients is a multifaceted process that brings both burden and satisfaction to family caregivers. It is expected that home care services offered by the Korean long-term care insurance may contribute to decreasing the burden of family caregivers and enhancing their satisfaction by assisting with practical caregiving tasks. - A cross-sectional study involving a convenience sample of 157 family caregivers recruited from five home care service agencies in South Korea. Information about the caregivers, caregiving history, older care recipients and use of home care services was assessed. The effects of home care service use on caregiving burden and satisfaction were tested using hierarchical multiple regression analyses after adjusting for the characteristics of the caregivers, caregiving history and older care recipients. - There was no significant influence of home care service use on reducing caregiving burden or on increasing caregivers' satisfaction. Although several factors were associated with caregiving burden and satisfaction, family functioning was the most unique factor to significantly affect both caregiving burden and satisfaction. - Home care services might not automatically have a positive impact on caregivers' burden and satisfaction, but maintaining healthy family functioning is an important issue for family caregivers. The findings highlight the important need to reconsider ways to provide home care services and to develop nursing interventions to reinforce supportive family functioning. - Practical strategies for providing home care services should be developed through a concrete assessment of the family dynamics and the needs of family caregivers. Health professionals should play a pivotal role in performing the assessment and in developing interventions to strengthen supportive family functioning. |
| Ramaboa, K. K. K. M., & Fredericks, I. (2019). Demographic Characteristics Associated with the Likelihood to Use Paid Home Care for People with Dementia among South African Muslims. Dementia and geriatric cognitive disorders, 48(5-6), 337–348. https://doi.org/10.1159/000506511 | II- Research (Quant) | C- Paid home care for dementia support (South Africa) | Theme 3- Families and Caregivers (family preferences towards paid providers/paid family caregivers) | - In societies where the value of filial piety is observed, a preference for caregiving to take place at home exists. In fact, institutional and paid home care for people with dementia (PWD) are still taboo in some Muslim societies. However, economic development and globalization have resulted in intergenerational separation, thus impacting the ability of young adults to provide care for the elderly at home. - The research team established the demographic characteristics most likely to be associated with the use of paid home care - age, gender, education level, marital status, family structure, experience with dementia care in the family, and number of dependents - for PWD among South African Muslims. A survey, administered in the form of an online questionnaire, of Muslim families across each of the 9 provinces of South Africa was conducted. Multiple logistic regression was used to test the effects of the demographic variables on the type of care choice arrangement (family as primary caregiver vs. paid home caregiving). - 422 responses were analysed, 28% of which indicated the respondents' desire to use paid home caregivers. The multiple logistic regression results indicate that South African Muslim families are more likely to use paid home care if they are older (that is, over 40 years), are female, and have high levels of education. - Home-based care is touted as the next dementia care model. Given that intergenerational mobility is likely to increase as future generations continue to participate more in the labour market, minority groups with a disposition to the same familial social values will require appropriate support in order to cope with the demands of caring for PWD. Suitable interventions for Muslim families who are not open to using external assistance, as well as those who are, need to be administered to enable the caregiver and care recipient to thrive at home. |
| Fabius, C. D., & Parker, L. J. (2023). Paid Help and Caregiving Experiences of Black Caregivers of Community-Dwelling Older Adults. *Clinical gerontologist*, *46*(1), 91–100. https://doi.org/10.1080/07317115.2022.2099776 | II- Research (Quant) | C- Caregiver’s experiences of paid help (USA) | Theme 3- Families and Caregivers (support to find paid help and arrange suitable home care) | - This study aimed to examine associations between use of paid help and caregiving-related experiences (emotional, financial, and physical difficulty) of Black family and unpaid caregivers of older adults. The study examined a sample of N = 572 non-Hispanic Black caregivers of community-dwelling older adults receiving help with daily activities from the 2015 National Health and Aging Trends Study (NHATS) and National Study of Caregiving (NSOC). Guided by Pearlin's Stress Process Model, logistic regression models examine associations between assisting with finding paid help and caregiver experiences. - Black caregivers who helped care recipients find paid help more often had a college degree or higher, were helping older adults who received assistance with three or more self-care/mobility activities or who were living in poverty and were not receiving help with caregiving from family and friends. In fully adjusted models, assisting with finding paid help was associated with emotional (AOR 1.92) and physical (AOR 2.16) difficulty. - Greater efforts are needed to support Black family and unpaid caregivers who are caring for older adults using paid help. Future interventions that target Black caregivers of older adults using paid help could be useful for improving caregiving experiences. |
| Colak, M., Gokdemir, O., Yaprak, S., and Kartal, M. (2016).Evaluation of home care services at a training and research hospital in Izmir. Marmara Medical Journal. | II- Research (Quant) | B- Health needs and demographics of patients receiving hospital home care services (Turkey) | Theme 3- Interface with Other Services or Providers (hospital home care) | - The objective of this study was to define the demographic characteristics, health and dependence status of patients receiving home care services (HCSs) and the services needed and received by the patients. - This descriptive study included 120 patients that had HCSs from a training and research hospital home care unit, where the files of the patients were evaluated retrospectively between January and August 2010. - Most of the patients in need of HCSs were female (61.8%), their mean age was 72.6±16.4 (range: 16-105) and 75.5% were 65 years and above, while 79.2% were bedridden. The personal hygiene and nutritional status were deemed inappropriate in 21.7% and 31.7%, respectively. The most common diseases were hypertension (19.3%), cerebrovascular accidents (13.5%), and Alzheimer’s disease (9.5%). - Family members mentioned their training needs’ topics as nutrition (30.3%), sanitation-hygiene (21.2%), and general care (18.2%). - The study shows different aspects of HCSs including medical, nursing, and social welfare problems for all parties, especially family physicians. People applied for HCSs were mostly women, elders with impaired functional status and bedridden. Their caregivers were mostly family members who needed social support not only in economic means but also in training so that they can provide better care for their relatives. |
| Franzosa, E., Tsui, E. K., & Baron, S. (2018). Home Health Aides' Perceptions of Quality Care: Goals, Challenges, and Implications for a Rapidly Changing Industry. *New solutions : a journal of environmental and occupational health policy : NS*, *27*(4), 629–647. https://doi.org/10.1177/1048291117740818 | II- Research (Qual) | B- Home care aides views on quality care (USA) | Theme 3- Service Economic Perspectives (costs and benefits of payment models) | - Home care payment models, quality measures, and care plans are based on physical tasks workers perform, ignoring relational care that supports clients' cognitive, emotional, and social well-being. As states seek to rein in costs and improve the efficiency and quality of care, they will need to consider how to measure and support relational care. - Four focus groups (n = 27) of unionized, agency-based New York City home health aides. - Workers reported aide-client relationships were a cornerstone of high-quality care, and building them required communication, respect, and going the extra mile. Since much of this care was invisible outside the worker-client relationship, aides received little supervisory support and felt excluded from the formal care team. - Aligning payment models with quality requires understanding the full scope of services aides provide and a quality work environment that offers support and supervision, engages aides in patient care, and gives them a voice in policy decisions. |
| McGilton, K. S., Rochon, E., Sidani, S., Shaw, A., Ben-David, B. M., Saragosa, M., Boscart, V. M., Wilson, R., Galimidi-Epstein, K. K., & Pichora-Fuller, M. K. (2017). Can We Help Care Providers Communicate More Effectively With Persons Having Dementia Living in Long-Term Care Homes? *American journal of Alzheimer's disease and other dementias*, *32*(1), 41–50. https://doi.org/10.1177/1533317516680899 | II- Programme evaluation | B- Dementia training in long term care (Canada) | Theme 3- Service Economic Perspectives (costs and benefits of workforce training in long-term care) | - Effective communication between residents with dementia and care providers in long-term care homes (LTCHs) is essential to resident-centered care. - To determine the effects of a communication intervention on residents' quality of life (QOL) and care, as well as care providers' perceived knowledge, mood, and burden. - The intervention included (1) individualized communication plans, (2) a dementia care workshop, and (3) a care provider support system. Pre- and postintervention scores were compared to evaluate the effects of the intervention. A total of 12 residents and 20 care providers in an LTCH participated in the feasibility study. - The rate of care providers' adherence to the communication plans was 91%. Postintervention, residents experienced a significant increase in overall QOL. Care providers had significant improvement in mood and perceived reduced burden. The results suggest that the communication intervention demonstrates preliminary evidence of positive effects on residents' QOL and care providers' mood and burden. |
| Wagner, A., Schaffert, R., Möckli, N., Zúñiga, F., & Dratva, J. (2020). Home care quality indicators based on the Resident Assessment Instrument-Home Care (RAI-HC): a systematic review. *BMC health services research*, *20*(1), 366. https://doi.org/10.1186/s12913-020-05238-x | I- Systematic review | B- Home care quality indicators (worldwide) | Theme 3- Service Quality Indicators and Measures (home care quality indicators) | - One way of measuring the quality of home care are quality indicators (QIs) derived from data collected with the Resident Assessment Instrument-Home Care (RAI-HC). To produce meaningful results for quality improvement and quality comparisons across home care organizations (HCOs) and over time, RAI-HC QIs must be valid and reliable. - The aim of this systematic review was to identify currently existing RAI-HC QIs and to summarize the scientific knowledge on the validity and reliability of these QIs. A systematic review was performed using the electronic databases PubMed, CINAHL, Embase, PsycINFO and Cochrane Library. Studies describing the development process or the psychometric characteristics of RAI-HC QIs were eligible. The data extraction involved a general description of the included studies as well as the identified RAI-HC QIs and information on validity and reliability. The methodological quality of the identified RAI-HC QI sets was assessed using the Appraisal of Indicators through Research and Evaluation (AIRE) instrument. - Four studies out of 659 initial hits met the inclusion criteria. The included studies described the development and validation process of three RAI-HC QI sets comprising 48 unique RAI-HC QIs, which predominantly refer to outcome of care. Overall, the validity and reliability of the identified RAI-HC QIs were not sufficiently tested. The methodological quality of the three identified RAI-HC QI sets varied across the four AIRE instrument domains. None of the QI sets reached high methodological quality, defined as scores of 50% and higher in all four AIRE instrument domains. - This is the first review that systematically summarized and appraised the available scientific evidence on the validity and reliability of RAI-HC QIs. It identified insufficient reporting of RAI-HC QIs validation processes and reliability as well as missing state-of-the-art methodologies. The review provides guidance as to what additional validity and reliability testing are needed to strengthen the scientific soundness of RAI-HC QIs. Considering that RAI-HC QIs are already implemented and used to measure and compare quality of home care, further investigations on RAI-HC QIs reliability and validity is recommended. |
| Tang, X., Chen, X., Pang, Y., & Zhou, L. (2018). The development of quality indicators for home care in China. International journal for quality in health care : journal of the International Society for Quality in Health Care, 30(3), 208–218. https://doi.org/10.1093/intqhc/mzx202 | II- Research (Quant) | C- Home care service quality indicators (China) | Theme 3- Service Quality Indicators and Measures (home care quality indicators) | - The objective of this study was to develop a comprehensive system of quality indicators for home care in China. - The study used a modified Delphi technique and analytic hierarchy process. Twenty experts were invited to participate in the Delphi expert consultation. Experts rated the perceived importance of 92 potential indicators through two rounds of e-mail surveys in November and December 2016. The analytic hierarchy process was used to determine the relative importance of the quality indicators identified through the Delphi expert consultation. - After two rounds of Delphi expert consultation, 77 quality indicators were identified as important in the Chinese home care setting. The mean importance ratings ranged from 4.35 to 4.95 on a 5-point scale. The relative importance of indicators was assessed in the second round. The absolute and relative importance of 77 indicators identified as potentially valid measures of the quality of Chinese home care was determined. This instrument is the first set of home care quality indicators developed specifically for mainland China, and it should be useful in evaluating and improving the quality of Chinese home care. |
| Foong, H. Y., Siette, J., & Jorgensen, M. (2022). Quality indicators for home- and community-based aged care: A critical literature review to inform policy directions. Australasian journal on ageing, 41(3), 383–395. https://doi.org/10.1111/ajag.13103 | III- Review of the literature | B- Home care quality indicators (Australia) | Theme 3- Service Quality Indicators and Measures (home care quality indicators) | - Australia is lagging behind other countries in implementing quality indicators (QIs) in home- and community-based aged care. This research aimed to identify and appraise home care QI sets used internationally for older adults, to inform the future development and utilisation of QIs in the Australian context. - A systematic search of eligible studies outlining the development and validation of home care QI sets for older adults was undertaken. QIs were categorised using the Donabedian model to identify potential gaps in coverage of key areas of care quality. Each QI was classified as potentially "derivable" or not from existing national routinely collected datasets. Methodological quality was determined using the Appraisal of Indicators through Research and Evaluation instrument. Three sets of home care QIs developed and used internationally for older adults were identified. Two of the QI sets focused predominantly on clinical and functional aspects of care. Of 45 unique QIs, the majority were outcome measures (93%), with only three QIs measuring care processes (7%), and zero indicators measuring quality in terms of the structure of care (e.g., waiting time to access services). Nearly half of the individual indicators identified would require Australian home care providers to undertake additional data collection. There were significant methodological limitations in the development of QI sets, particularly in the scientific evidence domain. - This review identified important gaps in existing QI sets, which should be considered by policymakers, researchers, and other stakeholders when developing and applying QIs in the Australian setting. |
| Macdonald, M. T., Lang, A., Storch, J., Stevenson, L., Barber, T., Iaboni, K., & Donaldson, S. (2013). Examining markers of safety in homecare using the international classification for patient safety. *BMC health services research*, *13*, 191. https://doi.org/10.1186/1472-6963-13-191 | III- Review of the literature | B- Home care safety makers (Canada) | Theme 3- Service Quality Indicators and Measures (home care safety indicators) | - Homecare is a growth enterprise. The nature of the care provided in the home is growing in complexity. This growth has necessitated both examination and generation of evidence around patient safety in homecare. - The purpose of this paper is to examine the findings of a recent scoping review of the homecare literature 2004-2011 using the World Health Organization International Classification for Patient Safety (ICPS), which was developed for use across all care settings, and discuss the utility of the ICPS in the home setting. The scoping review focused on Chronic Obstructive Pulmonary Disease (COPD), and Congestive Heart Failure (CHF); two chronic illnesses commonly managed at home and that represent frequent hospital readmissions. - The scoping review identified seven safety markers for homecare: Medication mania; Home alone; A fixed agenda in a foreign language; Strangers in the home; The butcher, the baker, the candlestick maker; Out of pocket: the cost of caring at home; and My health for yours: declining caregiver health. The safety markers from the scoping review were mapped to the 10 ICPS high-level classes that comprise 48 concepts and address the continuum of health care: Incident Type, Patient Outcomes, Patient Characteristics, Incident Characteristics, Contributing Factors/Hazards, Organizational Outcomes, Detection, Mitigating Factors, Ameliorating Actions, and Actions Taken to Reduce Risk. - Safety markers identified in the scoping review of the homecare literature mapped to three of the ten ICPS classes: Incident Characteristics, Contributing Factors, and Patient Outcomes. The ICPS does have applicability to the homecare setting, however there were aspects of safety that were overlooked. A notable example is that the health of the caregiver is inextricably linked to the wellbeing of the patient within the homecare setting. The current concepts within the ICPS classes do not capture this, nor do they capture how care responsibilities are shared among patients, caregivers, and providers. |
| Guardian Professional (2013) Attitudes to homecare in England: research conducted by Guardian Professional in association with Department of Health: top line findings. pp. 38. London, UK. https://static.guim.co.uk/ni/1383067901570/Homecare-survey-full-result.pdf?guni=Article:in%20body%20link | II- Research (Qual) | B- Challenges of delivering quality home care (England) | Theme 3- Structural Organisation of Care and Jobs (a multifaceted workforce) | - Time constraints, low pay and lack of training for frontline staff are the key challenges facing care workers. Guardian Professional, together with Department of Health, have produced this piece of research in order to understand current attitudes towards homecare in England. - Survey participants were members of the Guardian Professional public sector and voluntary sector database ((the Guardian Social Care Network). 2,020 people started this survey (which was released on 15th July 2013 and closed on 7th August 2013), and 1,443 completed it (a completion rate of just over 70%.). - The top three most important factors in offering good homecare were identified as sufficient time for care (59%), friendly, respectful, capable care workers (58%) and choice about services (43%). - The main challenges facing organisations that provide care were too few fully trained care workers, council commissioning, and a shortage of care workers. Respondents also offered their ideas for improving homecare, for example making it more locally based and with smaller catchment areas. - As to users' experiences of homecare, 46% of respondents reported a positive experience with individual care staff, compared with 15% who said their experience was negative. 39% said their experience of local authorities assessing their needs and arranging care was poor or very poor, compared with just 17% who thought it was good or very good. Respect for homecare as a career was not seen as a high priority. |
| Busnel, C., Vallet, F., Ashikali, E. M., & Ludwig, C. (2022). Assessing multidimensional complexity in home care: congruencies and discrepancies between patients and nurses. *BMC nursing*, *21*(1), 166. https://doi.org/10.1186/s12912-022-00942-x | II- Research (Quant) | B- Assessing complexity as part of care plans (Switzerland) | Theme 3- Structural Organisation of Care and Jobs (assessing levels, types and complexity of client care and support needs) | - Person-centered care allows for the inclusion of the totality of a person's needs and preferences, beyond just the clinical or medical aspect. This approach requires the development of tools to allow for the integration of the patient in his/her healthcare. Based on a 30-item tool developed for nurses to evaluate the complexity of home care situations (COMID), this study proposed a version for the patients (i.e., COMID-P). Both instruments were used, independently by nurses and patients, to rate the complexity of individual situations, to compare ratings. - The COMID-P and the COMID were completed during the fraXity study at the patients' homes, independently by patients (aged 65 and over) and nurses. Item-level and scale-level analyses were performed using Kappa and McNemar tests, and intra-class correlation (ICC). - A total of 159 pairs of COMID and COMID-P ratings were retained for analyses. Results demonstrated a high degree of patient/nurse agreement for 12/30 items, a moderate agreement for 10/30 items, and a low degree of agreement for 7/30 items. The intra-class correlation between the COMID-P and the COMID was high (ICC= .826, 95%CI [.761-.873]). - The results demonstrate that patients and nurses can assess complexity using tools that have comparable structural properties. They also reveal congruencies and discrepancies in scoring the components of complexity, highlighting the need to reach consensus in designing care plans. Further work is needed to demonstrate the benefits of joint assessment in developing care plans that truly meet patients' needs. |
| Kelly C. (2017). Exploring experiences of Personal Support Worker education in Ontario, Canada. *Health & social care in the community*, *25*(4), 1430–1438. https://doi.org/10.1111/hsc.12443 | II- Research (Qual) | B- Educational issues in personal support worker home care (Canada) | Theme 3- Structural Organisation of Care and Jobs (casualisation of labour, disconnect between theory and working conditions) | - There is growing attention to the training and education of Personal Support Workers, or PSWs, who work in community, home and long-term care settings supporting older people and people with disabilities. In Ontario, Canada, amid a volatile policy landscape, the provincial government launched an effort to standardise PSW education. - Using qualitative methods, this study considered the question: What are the central educational issues reflected by students, working PSWs and key informants, and are they addressed by the PSW programme and training standards? Phase one was a public domain analysis completed between January and March 2014 and updated for major developments after that period. Phase two, completed between August 2014 and March 2015, included 15 key informant interviews and focus group discussions and mini-phone interviews with 35 working PSWs and current PSW students. - According to the participants, the central educational issues are: casualisation of labour that is not conveyed in educational recruitment efforts, disconnect between theory and working conditions, overemphasis on long-term care as a career path, and variability of PSW education options. While the standards should help to address the final issue, they do not address the other key issues raised, which have to do with the structural organisation of work. - The authors conclude that there is a disconnect between the experiences of students, PSWs and key informants and the policy decisions surrounding this sector. This is particularly significant as education is often touted as a panacea for issues in long-term and community care. In fact, the curriculum of some of the PSW programmes, especially those in public college settings, is robust. Yet, the underlying issues will remain barring a structural overhaul of the organisation of long-term and community care sectors founded on a social revaluing of older people and the gendered work of care. |
| Habibnejad-Ledari, H., Rabbani, M., & Ghorbani-Kutenaie, N. (2019). Solving a multi-objective model toward home care staff planning considering cross-training and staff preferences by NSGA-II and NRGA. *Scientia Iranica*, *26*(5), 2919-2935. doi: 10.24200/sci.2018.20800 | II- Research (Model) | B- Staffing in home care (Iran) | Theme 3- Structural Organisation of Care and Jobs (staffing policies, staff allocation models, rostering) | - Home Care (HC) staff assignment problem is defined as deciding which staff to assign to each patient. In this study, a multi-objective non-linear mathematical programming model is presented to address staff assignment problem considering cross-training of caregivers for HC services. The first objective of the model is to minimize the cost of workload balancing, cross-training, and maintenance. The second objective minimizes the number of employees for each service, while the third objective function maximizes the satisfaction level of caregivers. Several constraints including skill matching, staff preferences, regularity, synchronization, staff absenteeism, and multi-functionality are considered to build a service plan. - Due to NP-hardness of the problem, a Non-dominated Sorting Genetic Algorithm (NSGA-II) with a proposed who-rule heuristic initialization procedure is applied. Due to the absence of benchmark available in the literature, a Non-dominated Ranking Genetic Algorithm (NRGA) is employed to validate the obtained results. The data required to run the model are gathered from a real-world HC provider. The results indicate that the proposed NSGA-II is superior to the NRGA with regard to comparison indexes. Based on the results obtained, it is now possible to determine which staff to cross-train for each service and how to assign staff to services. |
| Shiri, M., Ahmadizar, F., Thiruvady, D., & Farvaresh, H. (2023). A sustainable and efficient home health care network design model under uncertainty. *Expert Systems with Applications*, *211*, 118185. | II- Research (Model) | B- Staffing in home care (Iran) | Theme 3- Structural Organisation of Care and Jobs (staffing policies, staff allocation models, rostering) | - To cater to the increasing demands, particularly during diseases such as Covid-19, the design and planning of home health care systems is of significant importance. - The current study proposes a multi-objective mixed-integer linear model for a home health care network in two stages; the first is the opening of efficient health centres, and the second is the routing and scheduling considering corporate social responsibility and efficiency. There are multiple objectives that the researchers consider, including minimization of total costs and inefficiency considerations, and maximization of social aspects. A novel aspect of this study is the consideration of social responsibility, which includes employment opportunities and regional economic development, and efficiency in terms of time, energy, and mismanagement of budgets. To measure efficiency, an augmented version of the data envelopment analysis approach is incorporated into the proposed optimization model. Additionally, the TH approach is developed as an interactive fuzzy method to deal with the proposed multi-objective model. Within the HHC problem, costs, social factors, and service time are inherently uncertain, and hence, to solve this problem, a robust-fuzzy approach is proposed. - The ensuing model is applied to a real case study of Kermanshah in Iran. Moreover, several problem instances motivated by real cases are generated with different characteristics to measure the performance of the proposed model and approach. The results show that decision-makers' preferences play a key role in human resource planning and regional development. Furthermore, the results confirm the efficiency of the proposed approach in different instances within reasonable time frames. |
| Polacsek, M., Goh, A., Malta, S., Hallam, B., Gahan, L., Cooper, C., Low, L. F., Livingston, G., Panayiotou, A., Loi, S., Omori, M., Savvas, S., Batchelor, F., Ames, D., Doyle, C., Scherer, S., & Dow, B. (2020). 'I know they are not trained in dementia': Addressing the need for specialist dementia training for home care workers. *Health & social care in the community*, *28*(2), 475–484. https://doi.org/10.1111/hsc.12880 | II- Research (Qual) | B- Dementia care training needs (Australia) | Theme 3- Structural Organisation of Care and Jobs (staffing policies, client/worker choices, rostering of carers) | - Argues that global population ageing has meant a rapid increase in the numbers of older people with dementia, most of whom live in their own homes. Staying at home is an important determinant of health and well-being. As care needs increase, the quality of community support which older people receive directly influences their capacity to remain in their own homes. While many are supported informally by family carers, formal support provided by home care workers often enables them to remain at home for longer period. However, providing community-based care for people with dementia can be challenging. Workers often lack training in dementia-specific care for clients with increasingly complex needs, and typically work without direct supervision. As the demand for person-centred home care for people with dementia increases, specialist dementia training for home care workers is urgently needed. - This qualitative study used in-depth interviews of a purposive sample, comprising 15 family carers and four older people with dementia, to understand the experience of receiving community care. Data analysis was guided by Braun and Clarke's approach to thematic analysis. - Overlapping themes, relating to home care workers' understanding of dementia, person-centred care, communication and rapport, mutual collaboration, and the influence of organisational constraints on continuity of care. Although participants acknowledged that service providers operated under challenging circumstances, they were frustrated with home care workers' lack of dementia knowledge and inconsistent staff rostering. Conversely, an understanding of the lived experience of dementia, effective communication and rapport, and continuity of care contributed significantly to a positive experience of receiving care. - The findings of this study will be used to inform the essential elements of a training program aimed at enabling and empowering a skilled, specialist home care workforce to support older people with dementia to live well at home for as long as possible. |
| Andersen, G. R., & Westgaard, R. H. (2015). Discrepancies in assessing home care workers' working conditions in a Norwegian home care service: differing views of stakeholders at three organizational levels. *BMC health services research*, *15*, 286. https://doi.org/10.1186/s12913-015-0945-6 | II- Research (Mixed method) | B- Quality of work environment (Norway) | Theme 3- Structural Organisation of Care and Jobs (staffing to avoid occupational injury, stress, sick leave, disability retirement) | - The present study is a follow-up study of factors contributing to an undesirable quality of work environment and sick leave rate in the home care services in a Norwegian municipality. The underlying assumption is that organizational discrepancies in the perceptions and appraisals of significant factors and processes in an organization have detrimental effects on the management of the organization and on work environment conditions. Thus, the study aim is to explore potential organizational discrepancies in the appraisals of factors relating to home care workers' working conditions. - The study, using a mixed-methods design, comprised six home care units. It included survey responses of home care workers (80 respondents, response rate 54 %) and qualitative descriptions of stakeholders' appraisals of organizational issues gathered through semi-structured interviews (33 interviews with stakeholders at three organizational levels). - Employees at different organizational levels in the home care services expressed divergent appraisals of factors related to the working conditions of home care workers, including impact of organizational measures (i.e. time pressure, work tasks, a new work program, organizational changes, budget model, budget allocation and coping strategies). Survey responses supported interview descriptions by home care workers. Results suggest that organizational discrepancy serve as an important barrier to a sustainable, well-functioning organization in general and to quality-enhancing changes to work procedures in particular. - It is recommended to improve communication channels and facilitate the exchange of information across levels to ensure a common understanding of matters significant to the organization of the home care services and to the work environment of home care workers. The prevalence and impact of organizational discrepancy should be included in organization research, particularly when exploring explanatory factors of an unhealthy organization. |
| Andersen, G. R., & Westgaard, R. H. (2013). Understanding significant processes during work environment interventions to alleviate time pressure and associated sick leave of home care workers--a case study. BMC health services research, 13, 477. https://doi.org/10.1186/1472-6963-13-477 | II- Research (Mixed method) | B- Work environment (Norway) | Theme 3- Structural Organisation of Care and Jobs (staffing to avoid occupational injury, stress, sick leave, disability retirement) | - Ergonomic and work stress interventions rarely show long-term positive effect. The municipality participating in this study received orders from the Norwegian Labour Inspectorate due to an identified unhealthy level of time pressure and responded by effectuating several work environment interventions. The study aim is to identify critical factors in the interaction between work environment interventions and independent rationalization measures in order to understand a potential negative interfering effect from concurrent rationalizations on a comprehensive work environment intervention. - The study, using a historic prospective mixed-method design, comprised 6 home care units in a municipality in Norway (138 respondents, response rate 76.2%; 17 informants). The study included quantitative estimations, register data of sick leave, a time line of significant events and changes, and qualitative descriptions of employee appraisals of their work situation gathered through semi-structured interviews and open survey responses. - The work environment interventions were in general regarded as positive by the home care workers. However, all units were simultaneously subjected to substantial contextual instability, involving new work programs, new technology, restructurings, unit mergers, and management replacements, perceived by the home care workers to be major sources of stress. Findings suggest that concurrent changes induced through rationalization resulted in negative exposure effects that negated positive work environment intervention effects, causing an overall deteriorated work situation for the home care workers. - Establishment and active utilization of communication channels from workers to managers are recommended in order to increase awareness of putative harmful and interruptive effects of rationalization measures. |
| Grasmo, S. G., Liaset, I. F., & Redzovic, S. E. (2021). Home care workers' experiences of work conditions related to their occupational health: a qualitative study. *BMC health services research*, *21*(1), 962. https://doi.org/10.1186/s12913-021-06941-z | II- Research (Qual) | B- Home care work experiences (Norway) | Theme 3- Structural Organisation of Care and Jobs (staffing to avoid occupational injury, stress, sick leave, disability retirement) | - The need for home care workers (HCWs) is rapidly growing in Norway due to the increasingly growing elderly population. HCWs are exposed to a number of occupational hazards and physically demanding work tasks. Musculoskeletal disorders, stress, exhaustion, high sick leave rates and a high probability of being granted a disability pension are common challenges. - This qualitative study explored the views of HCWs on how working conditions affect their safety, health, and wellbeing. A descriptive and explorative design was utilised using semi-structured individual interviews with eight HCWs from three home care units in a middle-sized Norwegian city. Interviews were conducted in the Norwegian language, audio-recorded, and transcribed verbatim. The data was analysed by systematic text condensation. Key data quotes were translated into English by the authors. - HCWs reported that meaningful work-related interactions and relationships contributed to their improved wellbeing. Challenging interactions, such as verbal violence by consumers, were deemed stressful. The unpredictable work conditions HCWs encounter in users' homes contributed to their exposure to environmental hazards and unhealthy physical workloads. This was the case, although the employer promoted ergonomic work practices such as ergonomic body mechanics when mobilising and handling of clients, using safe patient handling equipment. HCWs perceived high level of individual responsibility for complying with company safety policies and practices, representing a health barrier for some. Organisational frameworks created unhealthy work conditions by shift work, time pressure and staffing challenges. Performing tasks in accordance with HCWs professional skills and identity was perceived as health-promoting. - This study suggests that unpredictable working conditions at users' home can adversely affect the safety, health, and wellbeing of HCWs. The interaction between the unpredictable environment at users' homes, HCWs' perceived high level of individual responsibility for complying with company safety policies and practices, and staffing challenges due to sickness-related absences upon the workplace creates tense work conditions with a negative influence on HCWs health. |
| Liaset, I. F., Fimland, M. S., Holtermann, A., Mathiassen, S. E., & Redzovic, S. (2023). Can home care work be organized to promote health among the workers while maintaining productivity? An investigation into stakeholders' perspectives on organizational work redesign concepts based on the Goldilocks Work principles. *BMC health services research*, *23*(1), 667. https://doi.org/10.1186/s12913-023-09691-2 | II- Research (Qual) | B- Work redesign to distribute occupational physical activity between workers (Norway) | Theme 3- Structural Organisation of Care and Jobs (staffing to avoid occupational injury, stress, sick leave, disability retirement) | - Due to the aging population, the need for home care services is increasing in most Western countries, including Norway. However, the highly physical nature of this job could contribute to make recruiting and retaining qualified home care workers (HCWs) challenging. This issue may be overcome by adopting the Goldilocks Work principles, aiming at promoting workers' physical health by determining a "just right" balance between work demands and recovery periods while maintaining productivity. - The aim of this study was to 1) gather suggestions from home care employees on suitable organizational (re)design concepts for promoting HCWs' physical health and 2) have researchers and managers define actionable behavioral aims for the HCWs for each proposed (re)design concept and evaluate them in the context of the Goldilocks Work principles. - HCWs, safety representatives, and operation coordinators (n = 14) from three Norwegian home care units participated in digital workshops led by a researcher. They suggested, ranked, and discussed redesign concepts aimed at promoting HCWs' health. The redesign concepts were subsequently operationalized and evaluated by three researchers and three home care managers. - Workshop participants suggested five redesign concepts, namely "operation coordinators should distribute work lists with different occupational physical activity demands more evenly between HCWs", "operation coordinators should distribute transportation modes more evenly between HCWs", "Managers should facilitate correct use of ergonomic aids and techniques", "HCWs should use the stairs instead of the elevator", and "HCWs should participate in home-based exercise training with clients". Only the first two redesign concepts were considered to be aligned with the Goldilocks Work principles. A corresponding behavioral aim for a "just right" workload was defined: reduce inter-individual differences in occupational physical activity throughout a work week. - Operation coordinators could have a key role in health-promoting organizational work redesign based on the Goldilocks Work principles in home care. By reducing the inter-individual differences in occupational physical activity throughout a work week, HCWs' health may be improved, thus reducing absenteeism and increasing the sustainability of home care services. The two suggested redesign concepts should be considered areas for evaluation and adoption in practice by researchers and home care services in similar settings. |
| Nisbet, E., & Morgan, J. C. (2019). Where Policy Meets Practice: Employer Perspectives on Scheduling and Hours for Home Care Aides. Journal of applied gerontology : the official journal of the Southern Gerontological Society, 38(11), 1615–1634. https://doi.org/10.1177/0733464817739153 | II- Research (Qual) | B- Working hours (USA) | Theme 3- Structural Organisation of Care and Jobs (staffing to support job satisfaction and retention) | - In the context of growing demand for home-based direct care services, the need to retain direct care workers (DCWs) is clear. The Patient Protection and Affordable Care Act, changes to the Fair Labor Standards Act, and state-level changes in Medicaid support for home-based care together have affected agencies that hire DCWs, with implications for an issue that affects worker satisfaction: scheduling. Many home-based aides employed by agencies cannot count on consistent or sufficient hours. Hours shortfall and instability have been recognized as important issues for retail and restaurant workers but focused on less for care aides. - This study uses semi structured interviews with agency representatives to examine these issues from an employer perspective, with a focus on how the competing influences of health care, labor, and employment policy shape scheduling and a review of how recommendations for changes in policy and practice in other sectors might apply to home care. |
| Johannessen, T., Ree, E., Aase, I. et al. (2020) Exploring challenges in quality and safety work in nursing homes and home care – a case study as basis for theory development. BMC Health Serv Res 20, 277. https://doi-org.proxy.library.rcsi.ie/10.1186/s12913-020-05149-x | II- Research (Qual) | C- Service quality and safety (Norway) | Theme 3- Structural Organisation of Care and Jobs (staffing to support safety) | - Management, culture and systems for better quality and patient safety in hospitals have been widely studied in Norway. Nursing homes and home care, however, have received much less attention. An increasing number of people need health services in nursing homes and at home, and the services are struggling with fragmentation of care, discontinuity, and restricted resource availability. - The aim of the study was to explore the current challenges in quality and safety work as perceived by managers and employees in nursing homes and home care services. The study is a multiple explorative case study of two nursing homes and two home care services in Norway. Managers and employees participated in focus groups and individual interviews. The data material was analyzed using directed content analysis guided by the theoretical framework ‘Organizing for Quality’, focusing on the work needed to meet quality and safety challenges. - Challenges in quality and safety work were interrelated and depended on many factors. In addition, they often implied trade-offs for both managers and employees. Managers struggled to maintain continuity of care due to sick leave and continuous external-facilitated change processes. Employees struggled with heavier workloads and fewer resources, resulting in less time with patients and poorer quality of patient care. The increased external pressure affected the possibility to work towards engagement and culture for improvement, and to maintain quality and safety as a collective effort at managerial and employee levels. - Despite contextual differences due to the structure, size, nature and location of the nursing homes and home care services, the challenges were similar across settings. The study indicates a dualistic contextual dimension. Understanding contextual factors is central for targeting improvement interventions to specific settings. Context is, however, not independent from the work that managers do; it can be and is acted upon in negotiations and interactions to better support managers’ and employees’ work on quality and safety in nursing homes and home care. |
| Backhouse, T., & Ruston, A. (2022). Home-care workers' experiences of assisting people with dementia with their personal care: A qualitative interview study. *Health & social care in the community*, *30*(3), e749–e759. https://doi.org/10.1111/hsc.13445 | II- Research (Qual) | B- Home workers dementia care (England) | Theme 3- Structural Organisation of Care and Jobs (sufficient time for client interactions and interpersonal sensitivity) | - Home-care workers are increasingly caring for clients living with dementia. Workers usually have limited dementia training and are low paid and often lone working. Little is known about how home-care workers assist people with dementia with their personal care. - This study aimed to explore the experiences of home-care workers and the knowledge and skills they rely on when providing personal care to people with dementia. In 2020, 17 semi-structured, face-to-face interviews with home-care workers in the East of England. Analysis was inductive and thematic. - Two key themes were present in the data: 'structural conditions' and 'clients' dementia-related characteristics'. For each of these, findings illustrated the challenges faced by home-care workers and the strategies they used to manage these challenges. Challenges included time allocation for visits, completing care plan tasks, lone working, communication and understanding, refusals of care, and client behaviours. To mitigate these challenges, home-care workers utilised system support, time management, training and experience and enacted a caring relationship, thought about their approach, and used distraction and communication skills. Workers relied on skills such as, relationship building, team working, observation, communication, decision making and interpersonal sensitivity. They drew on knowledge about the person, the person's needs, their own abilities, company policies and procedures and their role and responsibilities as a home-care worker. Home-care workers had more scope to mitigate client-based challenges by adapting care within client interactions, than to manage structural challenges where there was a limit to what workers could do. - Despite a commissioning focus on time- and task-based care, when caring for people with dementia, home-care workers used interaction as a way to bring the person along and complete care activities. Home-care services should acknowledge the importance of interactions with people with dementia within home care and support their workers to develop interpersonal sensitivity. |
| Bandini, J. I., Siconolfi, D., Feistel, K., & Etchegaray, J. (2023). Low Tech, High Potential: Using Technology to Improve Communication across Home Care Workers. *Journal of applied gerontology : the official journal of the Southern Gerontological Society*, *42*(4), 776–781. https://doi.org/10.1177/07334648221144027 | II- Research (Mixed method) | B- Use of technologies in home care (USA) | Theme 4- Digital Technology and Digital Skills in Home Care (digital communication technologies in home care, electronic care plans) | - This study sought to examine how technology is currently utilized in home care and how the integration of new technologies in the completion of tasks may change the future of work for home care workers (HCWs), including personal care aides and home health aides. - The study triangulated data from three sources: A scoping review, interviews with HCWs, and monthly stakeholder input from 17 experts in home care and technology. - The findings suggest that while current technology use is limited and rudimentary within home care, technology may be especially beneficial in mitigating challenges around communication handoffs among HCWs. The study points to the potential for the introduction and integration of technology into home care, particularly for communication to improve direct care worker experiences in providing care to vulnerable clients in their homes. |
| Oung, C., et al (2021) Developing the digital skills of the social care workforce: evidence from the Care City test bed. Nuffield Trust. pp. 32. London, UK. https://www.nuffieldtrust.org.uk/sites/default/files/2021-09/workforce-research-summary-final.pdf | II- Research (Mixed method) | B- Digital skills in home care (England) | Theme 4- Digital Technology and Digital Skills in Home Care (digital skills and use of digital technologies for home care skills development) | - This research summary explores how domiciliary care agencies have trained staff to use digital technology in order to monitor the vital signs of services users, and presents findings relating to the experience and skills development of care staff. The findings draw on a mixed-methods evaluation of the Care City test bed, which piloted a number of innovations in three distinct care pathways in East London. - The summary describes the key benefits and challenges of upskilling staff through digital innovations and offers ideas on how to maximise the potential for digital tools to aid skill development in the domiciliary care workforce. It also makes the case for more investment and joint working to ensure that the use of digital health technologies in social care settings is seen as a joint responsibility and priority between health and social care services. - Key findings include: care staff are often undervalued and underpaid, with limited opportunities for career progression; providing opportunities for care workers to develop new skills and use new technologies can improve job satisfaction and help staff progress towards their career goals or inspire new career pathways; digital skills development must sit within a wider career pathway for social care; and harnessing the potential of care staff to provide more joined up care highlights the need for buy-in from stakeholders across health and social care. |
| Sterling, M. R., Dell, N., Tseng, E., Okeke, F., Cho, J., Piantella, B., & Tobin, J. N. (2020). Home care workers caring for adults with heart failure need better access to training and technology: A role for implementation science. Journal of clinical and translational science, 4(3), 224–228. https://doi.org/10.1017/cts.2020.36 | II- Research (Qual) | B- Use of health technology for heart failure (USA) | Theme 4- Digital Technology and Digital Skills in Home Care (digital communication technologies in home care, electronic care plans) | - Although highly involved in heart failure (HF) patients' care, home care workers (HCWs) lack HF training and are poorly integrated into the healthcare team. - This study examined the role of technology among HCWs caring for HF patients. It involved 38 interviews with key stakeholders. - Overall, four themes emerged. Participants reported that technology is critical for HF care, but existing systems are outdated and ineffective. HCWs also have limited access to electronic resources. Technology, training, and principles of implementation science can be leveraged to improve HCWs' experience in caring for HF patients and home healthcare delivery. |
| Welch, V., Mathew, C. M., Babelmorad, P., Li, Y., Ghogomu, E. T., Borg, J., Conde, M., Kristjansson, E., Lyddiatt, A., Marcus, S., Nickerson, J. W., Pottie, K., Rogers, M., Sadana, R., Saran, A., Shea, B., Sheehy, L., Sveistrup, H., Tanuseputro, P., Thompson-Coon, J., … Howe, T. E. (2021). Health, social care and technological interventions to improve functional ability of older adults living at home: An evidence and gap map. *Campbell systematic reviews*, *17*(3), e1175. https://doi.org/10.1002/cl2.1175 | III- Review of the literature | B- Use of technologies to support functional ability (worldwide) | Theme 4- Digital Technology and Digital Skills in Home Care (functional ability technologies) | - By 2030, the global population of people older than 60 years is expected to be higher than the number of children under 10 years, resulting in major health and social care system implications worldwide. Without a supportive environment, whether social or built, diminished functional ability may arise in older people. Functional ability comprises an individual's intrinsic capacity and people's interaction with their environment enabling them to be and do what they value. - This evidence and gap map aims to identify primary studies and systematic reviews of health and social support services as well as assistive devices designed to support functional ability among older adults living at home or in other places of residence. - The authors systematically searched from inception to August 2018 in: MEDLINE, EMBASE, Cochrane Database of Systematic Reviews, CENTRAL, CINAHL, PsycINFO, AgeLine, Campbell Library, ASSIA, Social Science Citation Index and Social Policy & Practice. They conducted a focused search for grey literature and protocols of studies (e.g., ProQuest Theses and Dissertation Global, conference abstract databases, Help Age, PROSPERO, Cochrane and Campbell libraries and ClinicalTrials.gov). Screening and data extraction were performed independently in duplicate according to our intervention and outcome framework. The review included completed and on-going systematic reviews and randomized controlled trials of effectiveness on health and social support services provided at home, assistive products and technology for personal indoor and outdoor mobility and transportation as well as design, construction and building products and technology of buildings for private use such as wheelchairs, and ramps. - Included articles were coded interventions and outcomes, and the number of studies that assessed health inequities across equity factors. The review mapped outcomes based on the International Classification of Function, Disability and Health (ICF) adapted categories: intrinsic capacities (body function and structures) and functional abilities (activities) and assessed methodological quality of systematic reviews using the AMSTAR II checklist. - After de-duplication, 10,783 records were screened. The map includes 548 studies (120 systematic reviews and 428 randomized controlled trials). Interventions and outcomes were classified using domains from the International Classification of Function, Disability and Health (ICF) framework. - Most systematic reviews (n = 71, 59%) were rated low or critically low for methodological quality. The most common interventions were home-based rehabilitation for older adults (n = 276) and home-based health services for disease prevention (n = 233), mostly delivered by visiting healthcare professionals (n = 474). There was a relative paucity of studies on personal mobility, building adaptations, family support, personal support and befriending or friendly visits. The most measured intrinsic capacity domains were mental function (n = 269) and neuromusculoskeletal function (n = 164). The most measured outcomes for functional ability were basic needs (n = 277) and mobility (n = 160). There were few studies which evaluated outcome domains of social participation, financial security, ability to maintain relationships and communication. There was a lack of studies in low- and middle-income countries (LMICs) and a gap in the assessment of health equity issues. There is substantial evidence for interventions to promote functional ability in older adults at home including mostly home-based rehabilitation for older adults and home-based health services for disease prevention. Remotely delivered home-based services are of greater importance to policymakers and practitioners in the context of the COVID-19 pandemic. This map of studies published prior to the pandemic provides an initial resource to identify relevant home-based services which may be of interest for policymakers and practitioners, such as home-based rehabilitation and social support, although these interventions would likely require further adaptation for online delivery during the COVID-19 pandemic. There is a need to strengthen assessment of social support and mobility interventions and outcomes related to making decisions, building relationships, financial security, and communication in future studies. More studies are needed to assess LMIC contexts and health equity issues. |
| Turjamaa, R., Vaismoradi, M., Kajander-Unkuri, S., & Kangasniemi, M. (2023). Home care professionals' experiences of successful implementation, use and competence needs of robot for medication management in Finland. *Nursing open*, *10*(4), 2088–2097. https://doi.org/10.1002/nop2.1456 | II- Research (Qual) | B- Robot for medications (Finland) | Theme 4- Digital Technology and Digital Skills in Home Care (robots for medication administration) | - This article describes home care professionals' individual experiences of the implementation, use and competence needs of a robot for medication management in older people's home care. - A qualitative focus group interview study. Data were collected during spring and autumn 2021 by semi-structured focus group interviews and analysed using inductive content analysis. The participants were 62 home care professionals working in older people's home care. - The successful implementation and use of the robot for medication management consisted of a timely and adequate introduction before the implementation of the robot, the fluent usability of the robot in daily work, and confidence in work competence. - The authors conclude that there is a need for the reorganization of home care professionals' use of digital solutions to make workflow fluent, prevent burnout and turnover among home care professionals. Professionals' competence should also be developed to ensure that it corresponds to digitalized healthcare. |
| Ma, G., Hou, J., Peng, S., Liu, Y., Shi, Z., Fan, Y., & Zhang, J. (2022). Construction of Internet +home Care Quality Supervision Indicators in China Based on the Delphi Method. *Risk management and healthcare policy*, *15*, 1325–1341. https://doi.org/10.2147/RMHP.S368592 | II- Research (Mixed method) | B- Internet and home care based chronic disease management (China) | Theme 4- Digital Technology and Digital Skills in Home Care (use of the internet in home care) | - With the aging of China's population, the incidence rate of chronic diseases is rising. At the same time, residents' health awareness is also increasing. Implementing Internet +home care is an inevitable trend in adapting social development. Currently, Internet +home care is in the pilot stage in China, relevant institutional measures are neither standardized nor perfect, and there is no set of quality supervision indicators (QSIs). - The construction of Internet +home care QSIs in China will provide a theoretical basis for Internet +home care quality supervision. The Service-Quality model was used as the theoretical framework, and a literature review, semi-structured interviews, focus group discussions, and Delphi consultations determined the contents of the QSIs and the weight of each indicator. - Internet +home care QSIs were constructed and included 5 first-level indicators, 17 secondary indicators, and 69 tertiary indicators. The effective recovery rates of the two rounds of Delphi expert consultation were 100% and 85%, the expert authority coefficients were 0.810 and 0.833, and the Kendall harmony coefficients were 0.189 and 0.125. The final set of Internet +home care QSIs was as follows: tangibility (4 secondary and 16 tertiary indicators), reliability (4 secondary and 19 tertiary indicators), guarantee (5 secondary and 20 tertiary indicators), responsiveness (2 secondary and 8 tertiary indicators), and empathy (2 secondary and 6 tertiary indicators). - The construction of Internet +home care QSIs based on the SERVQUAL model is scientifically valid, and the indicators are reliable. They provide guidance and reference values for the continuous improvement and promotion of Internet +home care. This work also provides a theoretical basis for researching and developing an Internet +home care quality supervision platform. |
| Savassi, L. C. M., Dias, M. B., Boing, A. F., Verdi, M., & Lemos, A. F. (2020). Educational strategies for human resources in home health care: 8 years' experience from Brazil. Revista panamericana de salud publica = Pan American journal of public health, 44, e103. https://doi.org/10.26633/RPSP.2020.103 | II- Research (Quant) | B- Self-instruction online courses for home care (Brazil) | Theme 4- Employee Voice, Engagement and Involvement (learning strategies, challenges and needs) | - To analyze characteristics, enrollments and completion rates of healthcare professionals enrolled in Self-Instructional Online Courses of the Home Health Care Multicentre Qualification Program, developed by the Ministry of Health and the Universidade Aberta do SUS (UNASUS), and its relationship with Home Health Care Teams implementation. - Data were extracted from the Self-Instructional Online Courses' UNASUS enrollment platform database (2012-2018), cross-referenced with the Health Facilities' National Database and compared to Home Care General Coordination team's database. Main outcomes were completion rates and number of courses enrollments, analyzed by sex, age, region, location, profession, workplace, health teams and course type. - Men applied to courses slightly more than women and completion rates were higher (37.1 vs 30.5); there was a small decline in completion rates by age groups (from 32.8% in 18-29 yr to 31.1% in 46-50 yr age group) and a rise in course enrollment number, probably related to progressively "digital native" generations. Self-Instructional Online Courses were attended in all Brazilian states and reached all municipality sizes, with completion rates rising from 29.9% in the North to 37.3 in the South; 30-hour courses were completed by almost twice as many professionals as 45-hour and 60-hour courses, suggesting that modularity may improve completion rates. State distribution and national coverage suggest adequate range and coincidence between enrollment and Home Health Care Teams distribution. - Regional aspects influence professional interaction with courses; the feminization of health professions and women's lower completion rates suggest the need for a deeper gender perspective in health facilities and training services. Self- Instructional Online Courses for Home Health Care were an important outreach strategy, with professional's doubts answered more contextually. |
| Pavloff, M., & Labrecque, M. E. (2021). Continuing Education for Home Care Nurses: An Integrative Literature Review. Home healthcare now, 39(6), 310–319. https://doi.org/10.1097/NHH.0000000000001005 | III- Review of the literature | C- Continuing nursing education in home care (Canada) | Theme 4- Employee Voice, Engagement and Involvement (learning strategies, challenges and needs) | - With the wide range of clinical skills and responsibilities that home care nurses (HCNs) are expected to perform, it is important they are supported with access to relevant continuing nursing education (CNE) to perform their job safely and effectively. An integrative literature review was conducted to explore the current evidence on CNE for HCNs. Medline and CINAHL were searched and 13 articles that met the criteria were reviewed. - The analysis identified three themes: (1) learning strategies (simulation, virtual gaming, eLearning, traditional learning); (2) challenges (staffing, time, access, skill) and opportunities (incentive to stay employed, decreased burnout); and (3) learning needs (palliative, patient and family needs, older adults and dementia, acute nursing skills). - Nurses who provide care to patients in their homes have very complex roles and responsibilities. In order to keep patients and nurses safe, standards of education for HCNs, beyond their basic education program, must be developed. These educational standards must be designed to address the complex medical needs of patients while making the educational opportunities accessible and value-added. Improving the CNE experience for HCNs has the potential to increase patient safety, improve care outcomes, increase nurse competence, improve retention, and decrease nurse burnout. |
| Gebhard, D., & Herz, M. (2023). How to address the health of home care workers: a systematic review of the last two decades. Journal of Applied Gerontology, 42(4), 689-703. | I- Systematic review | A- Health interventions for home care workers (worldwide) | Theme 4- Employee Voice, Engagement and Involvement (tailored and participative development of staff health interventions) | - Making home care a healthy workplace is a societal concern but research on specific interventions is still scarce. The aim of this systematic review was to provide an initial overview of interventions addressing home care workers’ health. All (quasi-) experimental studies, presenting any intervention among home care employees, and reporting any outcome related to occupational health, safety, or well-being were included. PsycArticles, Medline, PubMed, and Web of Science were searched from January 2000 to February 2022. - Of the 16,345 identified publications, 18 studies with 2432 participants were included and assessed with the Joanna Briggs Institute Critical Appraisal Tools. - Organizational and training/educational approaches were found in five studies each, a behavioral approach in one, and seven studies presented a combined approach. Due to methodological limitations and the heterogeneity of interventions, the existing studies are insufficient to inform new programs, but emphasize the need for tailored approaches, integrated concepts, and participatory intervention development. |
| Lawani, M. A., Turgeon, Y., Côté, L., Légaré, F., Witteman, H. O., Morin, M., Kroger, E., Voyer, P., Rodriguez, C., & Giguere, A. (2021). User-centered and theory-based design of a professional training program on shared decision-making with older adults living with neurocognitive disorders: a mixed-methods study. *BMC medical informatics and decision making*, *21*(1), 59. https://doi.org/10.1186/s12911-021-01396-y | II- Programme evaluation | B- Shared decision-making training for clinicians (Canada) | Theme 4- Employee Voice, Engagement and Involvement (user-centered and theory-based design) | - Little is known about the best approaches to design training for healthcare professionals. This study explored how user-centered and theory-based design contribute to the development of a distance learning program for professionals, to increase their shared decision-making (SDM) with older adults living with neurocognitive disorders and their caregivers. - In this mixed-methods study, healthcare professionals who worked in family medicine clinics and homecare services evaluated a training program in a user-centered approach with several iterative phases of quantitative and qualitative evaluation, each followed by modifications. The program comprised an e-learning activity and five evidence summaries. A subsample assessed the e-learning activity during semi-structured think-aloud sessions. A second subsample assessed the evidence summaries they received by email. All participants completed a theory-based questionnaire to assess their intention to adopt SDM. Descriptive statistical analyses and qualitative thematic analyses were integrated at each round to prioritize training improvements with regard to the determinants most likely to influence participants' intention. - Of 106 participants, 98 completed their evaluations of either the e-learning activity or evidence summary (93%). The professions most represented were physicians (60%) and nurses (15%). Professionals valued the e-learning component to gain knowledge on the theory and practice of SDM, and the evidence summaries to apply the knowledge gained through the e-learning activity to diverse clinical contexts. The iterative design process allowed addressing most weaknesses reported. - Participants' intentions to adopt SDM and to use the summaries were high at baseline and remained positive as the rounds progressed. Attitude and social influence significantly influenced participants' intention to use the evidence summaries (P < 0.0001). Despite strong intention and the tailoring of tools to users, certain factors external to the training program can still influence the effective use of these tools and the adoption of SDM in practice. - A theory-based and user-centered design approach for continuing professional development interventions on SDM with older adults living with neurocognitive disorders and their caregivers appeared useful to identify the most important determinants of learners' intentions to use SDM in their practice and validate the initial interpretations of learners' assessments during the subsequent evaluation round. |
| Palesy D. (2017) Learning in the Absence of Direct Supervision: Person-Dependent Scaffolding. Vocations and learning.10(3):365–82. | II- Research (Qual) | B- Scaffolding for learning in home care (Australia) | Theme 4- Employee Voice, Engagement and Involvement (user-centered and theory-based design of training programmes) | - Contemporary accounts of learning emphasise the importance of immediate social partners such as teachers and co-workers. Yet, much of our learning for work occurs without such experts. - This paper provides an understanding of how and why new home care workers use scaffolding to learn and enact safe manual handling techniques in their workplaces and suggests how their learning may be supported in the absence of direct supervision. - A qualitative approach was adopted for this inquiry, in which newly recruited workers were directly observed and interviewed in their workplaces following classroom training. When learning without direct supervision, these workers were found to use the scaffolding in person-dependent ways. They constructed, engaged with, and subsequently dismantled their scaffolding as personally required, rather than relying on their teacher to decide how and when these forms of learning support should be used and withdrawn. - Consequently, a range of scaffolds should be provided in the workplaces of these individuals, without rigid stipulations about how and when they are to be accessed. That is, the learners themselves should be encouraged to decide on the type and frequency of their interaction with the scaffolding provided, and to access or withdraw this support as required. |
| Attenborough, J., Abbott, S., Brook, J., & Knight, R. A. (2019). Everywhere and nowhere: Work-based learning in healthcare education. *Nurse education in practice*, *36*, 132–138. https://doi.org/10.1016/j.nepr.2019.03.004 | II- Research (Qual) | C- Work-based learning opportunities in healthcare settings (UK) | Theme 4- Employee Voice, Engagement and Involvement (user-centered and theory-based design of training programmes) | - The shortage of healthcare professions is a global issue, which has highlighted the need to establish effective practice learning. In 2015 the UK government introduced a change to the way that healthcare education is funded. A subsequent fall in applications to healthcare programmes and high levels of vacancies across the sector in the UK have led to widespread concern about workforce shortages, especially nurses. - Subsequently, initiatives that both address the shortage and aim to bridge the gap between registered nurse and healthcare support worker have been introduced, presenting opportunities to further develop the clinical workplace as a learning environment for employees. A sample of nine healthcare professionals was recruited: seven nurses and two allied health professionals. Semi-structured interviews were conducted between March and June 2018. These were recorded verbatim, transcribed, and thematically analysed. - Respondents identified opportunities for work-based learning and factors for success. The importance of an effective learning culture, commitment to work-based learning and time were identified as factors for success. Despite the richness of learning opportunities in healthcare, respondents identified challenges for both learners and supervisors in identifying these opportunities in the workplace. These findings have immediate relevance to healthcare education systems internationally. Areas for future research include the relationship between supervisor and learner and further insight into why the busiest areas might be identified as more effective learning environments. |
| Guerrero, L. R., Richter Lagha, R., Shim, A., Gans, D., Schickedanz, H., Shiner, L., & Tan, Z. (2020). Geriatric Workforce Development for the Underserved: Using RCQI Methodology to Evaluate the Training of IHSS Caregivers. *Journal of applied gerontology : the official journal of the Southern Gerontological Society*, *39*(7), 770–777. https://doi.org/10.1177/0733464818780635 | II- Research (Qual) | Barriers to training in home care  (USA) | Theme 4- Employee Voice, Engagement and Involvement (user-centered and theory-based design of training programmes) | - Caregivers play an important role in the in-home care of community dwelling older adults living with Alzheimer's disease or related dementias (ADRD); however, many of these caregivers lack training in caring for this vulnerable population. In 2015, a research team developed and implemented an interactive, community-based, knowledge and skills-based training program for In-Home Supportive Services (IHSS) caregivers [*TP]. - This report shares the results of a process evaluation of this training program as it evolved over the course of three training sessions in Riverside County, California. - An iterative evaluation process revealed the unique needs of training and assessing a population of demographically diverse adult learners and provides guidance for those planning to implement similar training in underserved communities. Factors such as reliance on self-reported abilities, language readability level, and test anxiety may have confounded attempts to capture learner feedback and actual knowledge gains from our caregiver training program. |
| Stone, R. (2021). Developing a quality home care workforce to complement family caregivers and bridge the emerging care gap. In *Bridging the family care gap* (pp. 321-340). Academic Press. | V- Comment | C- Training standards for health and personal care aides (USA) | Theme 4- Employee Voice, Engagement and Involvement (user-centered and theory-based design of training programmes) | - Home health and personal care aides are one of the largest groups of health care workers in the US, with nearly three million people providing direct care for people with serious illness living in the community. These home care workers face challenges in recruitment, training, retention, and regulation, and there is a lack of data and research to support evidence-based policy change. - Personal care aides receive little formal training, and they experience low pay and a lack of respect for the skill required for their jobs. High turnover and occupational injury rates are widely reported. There is little research on the factors associated with higher-quality home care, the extent to which worker training affects client outcomes, and how regulations affect access to and quality of home care. Health care leaders should seek to fill these gaps in knowledge, support the establishment of training standards and programs, implement Medicaid reimbursement strategies that incentivize improvements in pay and working conditions, reform regulations that now prevent the full utilization of home care workers, and create sustainable career pathways in home care policies. |
| Spetz, J., Stone, R. I., Chapman, S. A., & Bryant, N. (2019). Home and community-based workforce for patients with serious illness requires support to meet growing needs. *Health Affairs*, *38*(6), 902-909. | III- Review | C- Training standards for health and personal care aides (USA) | Theme 4- Employee Voice, Engagement and Involvement (user-centered and theory-based design of training programmes) | - Home health and personal care aides are one of the largest groups of health care workers in the US, with nearly three million people providing direct care for people with serious illness living in the community. These home care workers face challenges in recruitment, training, retention, and regulation, and there is a lack of data and research to support evidence-based policy change. - Personal care aides receive little formal training, and they experience low pay and a lack of respect for the skill required for their jobs. High turnover and occupational injury rates are widely reported. There is little research on the factors associated with higher-quality home care, the extent to which worker training affects client outcomes, and how regulations affect access to and quality of home care. - Health care leaders should seek to fill these gaps in knowledge, support the establishment of training standards and programs, implement Medicaid reimbursement strategies that incentivize improvements in pay and working conditions, reform regulations that now prevent the full utilization of home care workers, and create sustainable career pathways in home care policies. |
| Bluestone J, Ricca J, Traicoff D, Tchoualeu D. (2021) It’s time to move beyond traditional health care worker training approaches. *Glob Health Sci Pract*. 9(3):431-432. https://doi.org/10.9745/GHSP-D-21-00553 | V- Commentary | A- Effectiveness of training approaches | Theme 4- Employee Voice, Engagement and Involvement (user-centered and theory-based design of training programmes) | - Critiques classroom-based train of trainer (TOT) approaches to education, arguing that evidence has shown that there are approaches that deliver training more effectively and efficiently than the classic group-based training, which removes health care workers from the workplace, and that instead develop their skills in the workplace itself. Consequently, emphasis has increased on workplace-based training combined with mentorship and follow-up. Such approaches have been facilitated through expanded access to digital technology and real-time data to support just-in-time mobile learning. - Bluestone et al’s 2013 literature review on effective in-service training techniques, setting, frequency, and media found that interactive, case-based learning, hands-on practice or simulation, delivered in the workplace can improve learning outcomes; and that computer or mobile-delivered instruction, if appropriately designed for user engagement, can be equally as effective as live instruction. - Significant challenges remain in terms of operationalizing these newer approaches to improving health provider capacity, especially at scale and under routine conditions. There are also questions about how best to ground these efforts to support and improve health care provider performance in more comprehensive quality improvement initiatives. There are ample opportunities to leverage technology to support effective learning and more cost-effective and targeted supervision. |
| Morgan, J. C., Edris, N., Luz, C. C., Ochylski, D. P., Stineman, A., Winchester, L., & Chapman, S. A. (2018). Testing US state-based training models to meet health workforce needs in long-term care. Ageing international, 43, 123-140. | V- Commentary | C- Personal and home care aides (USA) | Theme 4- Employee Voice, Engagement and Involvement (user-centered and theory-based design of training programmes) | - The US health care system and its workforce is rapidly changing to meet the triple aim of reducing costs, increasing quality of care, and improving the patient experience. There is a need to align training models with system needs and patient preferences in ways that allow the most cost-effective members of the care team to shoulder increasing shares of this care. - One entry-level and in-demand class of health care workers are personal and home care aides (PHCAs). The US Bureau of Labor Statistics projects a 26% increase in PHCAs to over 2.2 million workers by 2024 (OOH 2014). System needs for rebalancing care from institutional settings into the community and patient preferences for in-home care have aligned to drive the need for PHCAs. The increasing prevalence of chronic disease, medical complexity and dementia mean that these workers will be required to handle increasingly challenging clients and function as a key member of increasingly integrated health care teams. Therefore, the development of new models of education and training are necessary. Standards for PHCA training are quite low (reported in Marquand and Chapman 2014) and states leave most training to employers with little to no oversight (Kelly et al. Journal of Applied Gerontology, 32(7): 804-832, 2013). - The purpose of this study is to present case studies of six state-based training models for PHCAs funded by the Affordable Care Act. The authors discuss state approaches to recruitment of trainees, curriculum design and delivery methods, and key lessons learned to inform model development internationally. |
| Williams, L., Rycroft-Malone, J., Burton, C. R., Edwards, S., Fisher, D., Hall, B., McCormack, B., Nutley, S. M., Seddon, D., & Williams, R. (2016). Improving skills and care standards in the support workforce for older people: a realist synthesis of workforce development interventions. *BMJ open*, *6*(8), e011964. https://doi.org/10.1136/bmjopen-2016-011964 | III- Review of literature | B- Workforce development interventions in older people’s services (UK) | Theme 4- Employee Voice, Engagement and Involvement (workforce development design) | - This evidence review was conducted to understand how and why workforce development interventions can improve the skills and care standards of support workers in older people's services. - Following recognised realist synthesis principles, the review was completed by (1) development of an initial programme theory; (2) retrieval, review and synthesis of evidence relating to interventions designed to develop the support workforce; (3) 'testing out' the synthesis findings to refine the programme theories and establish their practical relevance/potential for implementation through stakeholder interviews; and (4) forming actionable recommendations. Participants were stakeholders who represented services, commissioners and older people were involved in workshops in an advisory capacity, and 10 participants were interviewed during the theory refinement process. - Eight context-mechanism-outcome (CMO) configurations were identified which cumulatively comprise a new programme theory about 'what works' to support workforce development in older people's services. The CMOs indicate that the design and delivery of workforce development includes how to make it real to the work of those delivering support to older people; the individual support worker's personal starting points and expectations of the role; how to tap into support workers' motivations; the use of incentivisation; joining things up around workforce development; getting the right mix of people engaged in the design and delivery of workforce development programmes/interventions; taking a planned approach to workforce development, and the ways in which components of interventions reinforce one another, increasing the potential for impacts to embed and spread across organisations. - It is important to take a tailored approach to the design and delivery of workforce development that is mindful of the needs of older people, support workers, health and social care services and the employing organisations within which workforce development operates. Workforce development interventions need to balance the technical, professional, and emotional aspects of care. |
| Rycroft-Malone, J., Burton, C. R., Williams, L., Edwards, S., Fisher, D., Hall, B., McCormack, B., Nutley, S., Seddon, D., & Williams, R. (2016). *Improving skills and care standards in the support workforce for older people: a realist synthesis of workforce development interventions*. NIHR Journals Library. | III- Review of literature | B- Workforce development interventions in older people’s services (UK) | Theme 4- Employee Voice, Engagement and Involvement (workforce development design) | - Support workers make up the majority of the workforce in health and social care services for older people. There is evidence to suggest that support workers are not deployed as effectively as possible, are often undervalued, and that there are gaps in understanding support worker roles across different care settings. In the context of a population that is growing older, having a skilled and knowledgeable workforce is an imperative. Workforce development includes the support required to equip those providing care to older people with the right skills, knowledge and behaviours to deliver safe and high-quality services. - The review answered the question ‘how can workforce development interventions improve the skills and the care standards of support workers within older people’s health and social care services?’. - A realist synthesis was conducted. In realist synthesis, contingent relationships are expressed as context–mechanism–outcomes (CMOs), to show how particular contexts or conditions trigger mechanisms to generate outcomes. The review was conducted in four iterative stages over 18 months: (1) development of a theoretical framework and initial programme theory; (2) retrieval, review and synthesis of evidence relating to interventions designed to develop the support workforce, guided by the programme theories; (3) ‘testing out’ the synthesis findings to refine the programme theories and establish their practical relevance/potential for implementation; and (4) forming recommendations about how to improve current workforce development interventions to ensure high standards in the care of older people. - Twelve stakeholders were involved in workshops to inform programme theory development, and 10 managers, directors for training/development and experienced support workers were interviewed in phase 4 of the study to evaluate the findings and inform knowledge mobilisation. - Eight CMO configurations emerged from the review process, which provide a programme theory about ‘what works’ in developing the older person’s support workforce. The findings indicate that the design and delivery of workforce development should consider, and include, a number of starting points. These include personal factors about the support worker, the specific requirements of workforce development and the fit with broader organisational strategy and goals. - The review has resulted in an explanatory account of how the design and delivery of workforce development interventions work to improve the skills and care standards of support workers in older people’s health and social care services. Implications for the practice of designing and delivering older person’s support workforce development interventions are directly related to the eight CMO configuration of the programme theory. Our recommendations for future research relate both to aspects of research methods and to a number of research questions to further evaluate and explicate our programme theory. - Limitations were that reports of studies evaluating workforce development interventions tended to lack detail about the interventions that were being evaluated. The review found a lack of specificity in reports about what were the perceived and actual intended impacts from the workforce development initiatives being implemented and/or evaluated. |
| Sanerma, P., Miettinen, S., Paavilainen, E., & Åstedt-Kurki, P. (2020). A client-centered approach in home care for older persons - an integrative review. Scandinavian journal of primary health care, 38(4), 369–380. https://doi.org/10.1080/02813432.2020.1841517 | III- Review of the literature | C- Client centered care in home care for older people (Finland) | Theme 4- Models of Home Care Provision (client centered care, person-centred care) | - The objective of this study was to describe and synthesize client-centered care and service in home care for older people. The study was an integrative review using the guidelines for literature reviews by the Joanna Briggs Institute. The research process followed the Whittemore and Knafl framework and PRISMA toolkit in the selection of eligible articles. The CINAHL, Medline, Scopus, Web of Science and Social Sciences abstracts were searched for articles published between January 2007 and May 2020 according to previously designed search strategies. In total, 24 articles were deemed relevant for an analysis using a thematic analysis. - The analysis resulted in four themes with sub-themes which revealed that client-centered care and service in home care consist of: 1) Clients' involvement in their own care; self-care, decision-making, satisfactory daily life, 2) Family members' and care partners' participation in care; family members' and care partners' commitment to care, family members' and care partners' competence in care, 3) Communication and co-operation; communication models, empowerment, partnership, and 4) Evidence-based service competence; delivery and organization of services, implementation of services, versatile clinical skills, quality outcomes and personnel wellbeing. - According to the results, achieving client-centered care and service in home care requires the realization of all of the above aspects. The practice of nursing must better identify all dimensions of client-centered care and take these into account in the delivery of home care services. Client-centeredness is a fundamental value and the basis of nursing and care in home care provided for older persons. This paper deepens and structures the concept of client-centered care in the context of home care, assists professionals to understand the factors behind client-centered care within the home care environment, provides deeper understanding of the roles of the older person, family members, and the service system in developing client-centered services in home care for older persons. |
| Ordway, A., Johnson, K. L., Kneale, L., Amtmann, D., & Demiris, G. (2019). The Experience of Home Care Providers and Beneficiaries With Enhanced Training Requirements in Washington State. *Journal of aging and health*, *31*(10_suppl), 124S–144S. https://doi.org/10.1177/0898264319860298 | II- Programme evaluation | B- Training for Home Care Aides (USA) | Theme 4- Models of Home Care Provision (client directed care, personalization) | - The objective of this study was to understand the perceived impact of Washington State's upgraded training and certification requirements of long-term care workers providing personal care services from the perspectives of consumers and home care aides. - The study applied conventional qualitative content analysis to semi-structured interviews with 17 consumers and 10 certified home care aides. - The findings show that consumers in this study put a high premium on directing many aspects of their personal care services. The study also found that while home care aides supported what consumers desired for their own care, some were unsure how to reconcile providing individualized services with the State's standardized, competency-based training and certification program. State-based efforts, such as the one in Washington State, serve as an important starting point for building a broader effort toward the identification of competencies and associated training standards for the home care workforce. |
| Leverton, M., Burton, A., Beresford-Dent, J., Rapaport, P., Manthorpe, J., Azocar, I., Giebel, C., Lord, K., & Cooper, C. (2021). Supporting independence at home for people living with dementia: a qualitative ethnographic study of homecare. *Social psychiatry and psychiatric epidemiology*, *56*(12), 2323–2336. https://doi.org/10.1007/s00127-021-02084-y | II- Research (Qual) | A- Home care to support dementia (England) | Theme 4- Models of Home Care Provision (dementia support for independent living) | - The aim of this ethnographic study was to investigate how homecare workers support or inhibit independence in people living with dementia. - The study involved 100 h of participant observations with homecare workers (n = 16) supporting people living with dementia (n = 17); and 82 qualitative interviews with people living with dementia (n = 11), family carers (n = 22), homecare managers and support staff (n = 11), homecare workers (n = 19) and health and social care professionals (n = 19). Data were triangulated and analysed thematically. - Three themes: (1) independence and the home environment, highlighting ongoing negotiations between familiarity, suitability and safety for care; (2) independence and identity, exploring how homecare workers' understanding of their clients' identity can enable active participation in tasks and meaningful choices; and (3) independence and empowerment, considering the important position of homecare workers to advocate for clients living with dementia while navigating authoritative power amongst proxy decision-makers. - The authors argue that person-centred care should also be home-centred, respecting the client's home as an extension of self. Homecare workers can use their understanding of clients' identities, alongside skills in providing choice and developing relationships of interdependence to engage clients in everyday tasks. Homecare workers are well placed to advocate for their client's voice within the care network, although their ability to do so is limited by their position within power structures. |
| Hallberg, I. R., Cabrera, E., Jolley, D., Raamat, K., Renom-Guiteras, A., Verbeek, H., Soto, M., Stolt, M., & Karlsson, S. (2016). Professional care providers in dementia care in eight European countries; their training and involvement in early dementia stage and in home care. *Dementia (London, England)*, *15*(5), 931–957. https://doi.org/10.1177/1471301214548520 | III- Review of literature | B- Roles in caring for people in dementia (worldwide) | Theme 4- Models of Home Care Provision (dementia support) | - Knowledge concerning professionals involved in dementia care throughout its trajectory is sparse; the focus has mainly been on nursing-home care and less on home care, diagnosis and treatment of the disease and its complications even though home care is the most prominent type of care. - The aim of this study was to explore and describe professional care providers involved in dementia care and their educational level applying the International Standard Classification of Education (ISCED) and further to investigate practice in the RightTimePlaceCare-countries with regard to screening, diagnostic procedures and treatment of dementia and home care. - The findings demonstrate more similarities than differences in terms of type of professionals involved among the countries although untrained staff were more common in some countries. Findings also show that many types of professionals are involved, who to turn to may not be clear, for instance in terms of medical specialities and it may be unclear who bears the ultimate responsibility. The professionals involved in diagnosis, treatment and care are educated to bachelor's level or above whilst everyday care is provided by people trained at a lower ISCED level or with no formal training. Registered nurses as well as occupational therapists have bachelor's degrees in most countries, but not in Germany or Estonia. Professionals specifically trained in dementia care are not so common. - Further research is needed to reveal not only who provides the diagnostics and treatment, but also how home care is organised and quality assured. Many different types of professionals serve as providers along the trajectory of the disease which may be difficult for the patient and the informal caregiver to cope with. |
| Ageing well at home: Emergent Models of Home Care Provision and the professionalisation of the home care workforce (2018) https://drive.google.com/file/d/1ikHAedrFkH_J5lNc4b4I53fFcBuph4RZ/view | II- Research (Qual) | B- Calls for new models and innovation in models of home care provision (England) | Theme 4- Models of Home Care Provision (engagement and early intervention to avoid deterioration) | - Everyone would like to age well, and most people would prefer to do so, for as long as possible, in the comfort and security of their own home. The projected increase in the number of older people, together with continuing shortages of care workers, means home care is heading towards (and in some places has already reached) crisis. Home care is a profession negatively affected by poor pay and conditions and low status. - This Sustainable Care Policy Perspective provides insights from expert contributors on these and related issues, and makes recommendations for policymakers, providers and commissioners in home care and for the Sustainable Care programme. - Recommendations include a need for Government to invest in the home care workforce to improve its status as a profession and help reverse endemic recruitment and retention problems; and the need for the sector to develop new ways of working, including appropriate use of emerging technologies to improve wellbeing outcomes and ensure everyone is able to age well. |
| Walters, K., Frost, R., Kharicha, K., Avgerinou, C., Gardner, B., Ricciardi, F., Hunter, R., Liljas, A., Manthorpe, J., Drennan, V., Wood, J., Goodman, C., Jovicic, A., & Iliffe, S. (2017). Home-based health promotion for older people with mild frailty: the HomeHealth intervention development and feasibility RCT. *Health technology assessment (Winchester, England)*, *21*(73), 1–128. https://doi.org/10.3310/hta21730 | II- Research (Mixed method) | C- Frailty intervention support in home setting (England) | Theme 4- Models of Home Care Provision (frailty support, complex vulnerabilities and preventative care) | - Mild frailty or pre-frailty is common and yet is potentially reversible. Preventing progression to worsening frailty may benefit individuals and lower health/social care costs. However, little is known about effective approaches to preventing frailty progression. - Objectives: (1) To develop an evidence- and theory-based home-based health promotion intervention for older people with mild frailty. (2) To assess feasibility, costs, and acceptability of (i) the intervention and (ii) a full-scale clinical effectiveness and cost-effectiveness randomised controlled trial (RCT). - The study involved evidence reviews, qualitative studies, intervention development and a feasibility RCT with process evaluation. Two systematic reviews (including systematic searches of 14 databases and registries, 1990-2016 and 1980-2014), a state-of-the-art review (from inception to 2015) and policy review identified effective components for our intervention. Data on health priorities and potential intervention components were collected using semi structured interviews and focus groups with older people (aged 65-94 years) (n = 44), carers (n = 12) and health/social care professionals (n = 27). These data and evidence reviews fed into development of the 'HomeHealth' intervention in collaboration with older people and multidisciplinary stakeholders. - 'HomeHealth' comprised 3-6 sessions with a support worker trained in behaviour change techniques, communication skills, exercise, nutrition, and mood. Participants addressed self-directed independence and well-being goals, supported through education, skills training, enabling individuals to overcome barriers, providing feedback, maximising motivation, and promoting habit formation. - Feasibility of single-blind RCT, individually randomised to 'HomeHealth' or treatment as usual (TAU). Community settings in London and Hertfordshire, UK. A total of 51 community-dwelling adults aged ≥ 65 years with mild frailty. Main outcome measures: Feasibility - recruitment, retention, acceptability, and intervention costs. Clinical and health economic outcome data at 6 months included functioning, frailty status, well-being, psychological distress, quality of life, capability and NHS and societal service utilisation/costs. - The study successfully recruited to target, with good 6-month retention (94%). Trial procedures were acceptable with minimal missing data. Individual randomisation was feasible. The intervention was acceptable, with good fidelity and modest delivery costs (£307 per patient). A total of 96% of participants identified at least one goal, which were mostly exercise related (73%). - The study found significantly better functioning (Barthel Index +1.68), better grip strength (+6.48 kg), reduced psychological distress (12-item General Health Questionnaire -3.92) and increased capability-adjusted life-years [+0.017] at 6 months in the intervention arm than the TAU arm, with no differences in other outcomes. NHS and carer support costs were variable but, overall, were lower in the intervention arm than the TAU arm. The main limitation was difficulty maintaining outcome assessor blinding. - Evidence is lacking to inform frailty prevention service design, with no large-scale trials of multidomain interventions. From stakeholder/public perspectives, new frailty prevention services should be personalised and encompass multiple domains, particularly socialising and mobility, and can be delivered by trained non-specialists. Our multicomponent health promotion intervention was acceptable and delivered at modest cost. Our small study shows promise for improving clinical outcomes, including functioning and independence. A full-scale individually RCT is feasible. |
| Social Care Wales (2017) Development of a strategic plan for care and support at home: literature review. pp. 50. Cardiff. https://socialcare.wales/cms-assets/documents/Care-and-support-at-home-in-Wales-Literature-review.pdf | III- Review of literature | B- Assessment of evidence on home care and innovative practice (Wales) | Theme 4- Models of Home Care Provision (outcomes-focused approaches) | - This rapid literature review provides a broad overview of the key messages emerging from the evidence on home care and signposts to examples of innovative practice. The review draws on research, policy and practice literature. The report summarises the main themes around domiciliary care workforce; person-centred, relationship based care; outcomes-focused services, specialist services, integrated care, commissioning, and characteristics of the home care market. - Workforce themes: workforce size and characteristics; training and qualifications; pay and job security; worker motivations and occupational status; recruitment, retention and good recruitment practice; and career pathways. The review also suggests that there is limited evidence on the effectiveness of person-centred, relationship based care and on the effectiveness of outcomes-focused approaches to home care. Examples of outcomes-focused innovative practice include: the Helped-to-live-at-Home service in Wiltshire and the local offer of support in Hertfordshire. The evidence related to dementia and end of life home care; reablement is also reported. |
| Darwich, A. S., Boström, A. M., Guidetti, S., Raghothama, J., & Meijer, S. (2023). Investigating the Connections Between Delivery of Care, Reablement, Workload, and Organizational Factors in Home Care Services: Mixed Methods Study. *JMIR human factors*, *10*, e42283. https://doi.org/10.2196/42283 | II- Research (Mixed method) | B- Home care systems model of reablement (Sweden) | Theme 4- Models of Home Care Provision (reablement, recovery or restorative home care) | - Home care is facing increasing demand due to an aging population. Several challenges have been identified in the provision of home care, such as the need for support and tailoring support to individual needs. Goal-oriented interventions, such as reablement, may provide a solution to some of these challenges. The reablement approach targets adaptation to disease and relearning of everyday life skills and has been found to improve health-related quality of life while reducing service use. - The objective of this study is to characterize home care system variables (elements) and their relationships (connections) relevant to home care staff workload, home care user needs and satisfaction, and the reablement approach. This is to examine the effects of improvement and interventions, such as the person-centered reablement approach, on the delivery of home care services, workload, work-related stress, home care user experience, and other organizational factors. The focus was on Swedish home care and tax-funded universal welfare systems. - The study used a mixed methods approach where a causal loop diagram was developed grounded in participatory methods with academic health care science research experts in nursing, occupational therapy, aging, and the reablement approach. The approach was supplemented with theoretical models and scientific literature. The developed model was verified by the same group of experts and empirical evidence. Finally, the model was analyzed qualitatively and through simulation methods. - The final causal loop diagram included elements and connections across the categories: stress, home care staff, home care user, organization, social support network of the home care user, and societal level. The model was able to qualitatively describe observed intervention outcomes from the literature. The analysis suggested elements to target for improvement and the potential impact of relevant studied interventions. For example, the elements "workload" and "distress" were important determinants of home care staff health, provision, and quality of care. - The developed model may be of value for informing hypothesis formulation, study design, and discourse within the context of improvement in home care. Further work will include a broader group of stakeholders to reduce the risk of bias. Translation into a quantitative model will be explored. |
| Rooijackers, T. H., Zijlstra, G. A. R., van Rossum, E., Vogel, R. G. M., Veenstra, M. Y., Kempen, G. I. J. M., & Metzelthin, S. F. (2021). Process evaluation of a reablement training program for homecare staff to encourage independence in community-dwelling older adults. BMC geriatrics, 21(1), 5. https://doi.org/10.1186/s12877-020-01936-7 | II- Research (Mixed methods) | B- Reablement programme (Holland) | Theme 4- Models of Home Care Provision (reablement, recovery or restorative home care) | - Many community-dwelling older adults experience limitations in (instrumental) activities of daily living, resulting in the need for homecare services. Whereas services should ideally aim at maintaining independence, homecare staff often take over activities, thereby undermining older adults' self-care skills and jeopardizing their ability to continue living at home. Reablement is an innovative care approach aimed at optimizing independence. The reablement training program 'Stay Active at Home' for homecare staff was designed to support the implementation of reablement in the delivery of homecare services. This study evaluated the implementation, mechanisms of impact and context of the program. - A process evaluation was conducted alongside a 12-month cluster randomized controlled trial, using an embedded mixed-methods design. One hundred fifty-four homecare staff members (23 nurses, 34 nurse assistants, 8 nurse aides and 89 domestic workers) from five working areas received the program. Data on the implementation (reach, dose, fidelity, adaptations, and acceptability), possible mechanisms of impact (homecare staff's knowledge, attitude, skills, and support) and context were collected using logbooks, registration forms, checklists, log data and focus group interviews with homecare staff (n = 23) and program trainers (n = 4). - The program was largely implemented as intended. Homecare staff's average compliance to the program meetings was 73.4%; staff members accepted the program, and particularly valued its practical elements and team approach. They experienced positive changes in their knowledge, attitude, and skills about reablement, and perceived social and organizational support from colleagues and team managers to implement reablement. However, the extent to which homecare staff implemented reablement in practice, varied. Perceived facilitators included digital care plans, the organization's lump sum funding and newly referred clients. Perceived barriers included resistance to change from clients or their social network, complex care situations, time pressure and staff shortages. - The program was feasible to implement in the Dutch homecare setting and was perceived as useful in daily practice. Nevertheless, integrating reablement into homecare staff's working practices remained challenging due to various personal and contextual factors. Future implementation of the program may benefit from minor program adaptations and a more stimulating work environment. |
| Whitehead, P. J., Worthington, E. J., Parry, R. H., Walker, M. F., & Drummond, A. E. (2015). Interventions to reduce dependency in personal activities of daily living in community dwelling adults who use homecare services: a systematic review. *Clinical rehabilitation*, *29*(11), 1064–1076. https://doi.org/10.1177/0269215514564894 | III- Review of Literature | B- Interventions to reduce dependency on support for activities of daily living (England) | Theme 4- Models of Home Care Provision (reablement, recovery or restorative home care) | - This review identified interventions that aim to reduce dependency in activities of daily living (ADL) in homecare service users. To determine: content; effectiveness in improving ability to perform ADL; and whether delivery by qualified occupational therapists influences effectiveness. - Data sources were The Cochrane Central Register of Controlled Trials, MEDLINE, EMBASE, AMED, CINAHL, PsycINFO, OTseeker, PEDro, Web of Science, CIRRIE, and ASSIA. Studies included: randomised controlled trials, non-randomised controlled trials and controlled before and after studies. Two reviewers independently screened studies for inclusion, assessed risk of bias and extracted data. A narrative synthesis of the findings was conducted. - Thirteen studies were included, with a total of 4975 participants. Ten (77%) were judged to have risk of bias. Interventions were categorised as those termed 're-ablement' or 'restorative homecare' (n=5/13); and those involving separate components which were not described using this terminology (n=8/13). Content of the intervention and level of health professional input varied within and between studies. Effectiveness on ADL: eight studies included an ADL outcome, five favoured the intervention group, only two with statistical significance, both these were controlled before and after studies judged at high risk of bias. ADL outcome was reported using seven different measures. Occupational therapy: there was insufficient evidence to determine whether involvement of qualified occupational therapists influenced effectiveness. - The findings demonstrate there is limited evidence that interventions targeted at personal ADL can reduce homecare service users' dependency with activities, the content of evaluated interventions varies greatly. |
| Global Coalition on Aging (2018) pp.36, London. Relationship-based home care: a sustainable solution for Europe's elder care crisis. https://globalcoalitiononaging.com/wp-content/uploads/2018/06/RHBC_Report_DIGITAL.pdf | II- Research (Mixed method) | B- Review and consultation on relationship-based home care (Europe) | Theme 4- Models of Home Care Provision (relationship-based home care) | - This report examines the growing and increasingly complex care challenges facing Europe due to rapid ageing. It explores how relationship-based home care, also referred to as outcomes-based home care or person-centred home care, can enable the delivery of high quality care that can improve lives. Relationship-based home care is based around the needs of the individual and his or her family, rather than a predefined set of task. - Drawing publicly available data and interviews with health and care experts across Europe, the report highlights how relationship-based home can result in benefits such as reduced care costs, better care coordination, better care for people living with dementia, better outcomes for older people, reduced burden on families, and increased employment opportunities. - The report also considers ways of tackling barriers to providing relationship-based care and proposes a set of policy actions to support its integration into European health and care systems. These include to: build a body of evidence that quantifies the value of relationship-based home care, make a highly skilled caregiving workforce the heart of the solution and promote caregiving as fulfilling career and, increase investment in high-quality, person-centred care for people living with dementia. |
| Abdi, S., Spann, A., Borilovic, J. *et al.* (2019) Understanding the care and support needs of older people: a scoping review and categorisation using the WHO international classification of functioning, disability and health framework (ICF). *BMC Geriatr* 19, 195. https://doi.org/10.1186/s12877-019-1189-9 | III- Review of literature | B- Older people’s care and support needs (UK) | Theme 4- Models of Home Care Provision (Rights, needs and eligibility for home care) | - The number of older people with unmet care and support needs is increasing substantially due to the challenges facing the formal and informal care system in the United Kingdom. Addressing these unmet needs is becoming one of the urgent public health priorities. In order to develop effective solutions to address some of these needs, it is important first to understand the care and support needs of older people. - A scoping review was conducted, using the Arksey and O’Malley original and enhanced framework, to understand the care and support needs of older people, focusing on those living at home with chronic conditions in the UK. The search was conducted using five electronic data bases, grey literature and reference list checks. The WHO International Classification of Functioning, Disability and Health (ICF) framework was used to analyse and categorise the literature findings. - Forty studies were included in the final analysis- 32 from academic literature and 8 from grey literature. The review highlighted that older adults faced a range of physical, social and psychological challenges due to living with chronic conditions and required care and support in three main areas: 1) social activities and relationships; 2) psychological health; and 3) activities related to mobility, self-care and domestic life. The review also highlighted that many older people demonstrated a desire to cope with their illness and maintain independence, however, environmental factors interfered with these efforts including: 1) lack of professional advice on self-care strategies; 2) poor communication and coordination of services; and 3) lack of information on services such as care pathways. A gap in the knowledge was also identified about the care and support needs of two groups within the older population: 1) older workers; and 2) older carers. - The review highlighted that older people living with chronic conditions have unmet care needs related to their physical and psychological health, social life, as well as the environment in which they live and interact. Findings of this review also emphasized the importance of developing care models and support services based around the needs of older people. |
| Scales, K. (2019). Meeting the integration mandate: the implications of Olmstead for the home care workforce. *Geo. J. on Poverty L. & Pol'y*, *27*, 261. | V- Commentary | B- Home care worker job quality (USA) | Theme 4- Models of Home Care Provision (rights, needs and eligibility for home care) | - In Olmstead v. LC ex rel. Zimring, the Supreme Court ruled that the unnecessary institutionalization of individuals with disabilities constitutes unlawful discrimination under the Americans with Disabilities Act (ADA).'By protecting the right of individuals to be free from unjustified segregation on the basis of disability, and to live independently in their own homes and communities, the 1999 ruling also placed an explicit obligation on states and public entities to provide the supports and services that are required to fulfill that right. - In the two decades since Olmstead, an estimated 50,000 individuals with physical disabilities, intellectual and development disabilities, and mental illness have benefitted from statewide settlements" giving them the opportunity to receive health, residential, employment, and day services in their communities and... to leave, or avoid entering, segregated institutions." Countless more have been impacted by the broader" rebalancing" trend in the long-term services and supports (LTSS) sector-ie, the shift in public spending from nursing homes and other congregate settings to home and community-based settings-which originated in the 1970s and gained considerable momentum in the years after Olmstead. Progress toward fulfilling Olmstead's" integration mandate" has been hindered, however, by inadequate attention to building and strengthening the home care workforce-the workforce which provides the daily personal assistance that is essential for many individuals with disabilities to live independently in the community. - Despite the escalating demand for their services and the increasing complexity of their role, home care workers continue to struggle to attain basic elements of job quality, such as livable wages, sustainable schedules, training and career development. |
| Wang, J., Wang, Y., Cai, H. et al. (2020) Analysis of the status quo of the Elderly’s demands of medical and elderly care combination in the underdeveloped regions of Western China and its influencing factors: a case study of Lanzhou. BMC Geriatr 20, 338. https://doi.org/10.1186/s12877-020-01616-6 | IV- Case study | C- Demand for elderly care (China) | Theme 4- Models of Home Care Provision (rights, needs and eligibility for home care) | - This study analysed the current demand by senior citizens in Lanzhou, China for a combination of medical and elderly care services and to identify the factors influencing their needs. 7500 participants aged 60 or above living in Lanzhou, China, were recruited, a unified questionnaire concerning elderly people’s demand for a service combining medical and elderly care has been adopted to survey these subjects. The status quo of the demand of the service combining medical and elderly care and its influencing factors were analysed with the single-factor Chi-square test and multi-factor binomial logistic regression method. - Results showed that 3772 of 7320 older people have the demand for the service combining medical and elderly care, accounting for 53.15% of survey respondents. Many factors are in play, including gender, marital status, degree of education, occupation before retirement, number of children, monthly income, health self-assessment status, endowment insurance type, medical insurance type, current nursing arrangements, old-age demands, self-care ability and the knowledge of combining medical and elderly care and the willingness to pay for the combination of medical and elderly care have statistical significance (P < 0.05) with the elderly’s needs. - Different ages, living styles and the prevalence of chronic diseases, have no statistical significance (P > 0.05) with the elderly’s care needs in Lanzhou. - The number of children, type of medical insurance and willingness to pay for the combination of medical treatment and nursing care are major influencing factors among the complex factors influencing the elderly’s demand for the proposed service. - The low knowledge rate and demand rate, the number of children, the type of medical insurance, and the willingness to pay for the medical-nursing combination service for the elderly in Lanzhou have a great impact on the elderly’s demand rate for combining medical and elderly care. - Argues that relevant government departments should focus more on the promotion of the endowment model of combining medical and elderly care and provide integrated medical care services by integrating multiple resources and improving social security. |
| Koehler, I. (2015) Key to Care Report Card. Local Government Information Unit, London.  https://lgiu.org/publication/key-to-care-one-year-on/ | II- Research (Mixed method) | B- Progress on recommendations for home care sector reform (England) | Theme 4- Policy, Research and Planning (identifying priorities for sector development) | - Updates progress on the 11 recommendations outlined in 'Key to Care: Report of the Burstow Commission on the Future of the Home Care Workforce', which set out what was needed to have a professional, well-paid, well-trained and properly regulated home care workforce to provide the quality of care at home that people need. - The report highlights the improvements that have been made, such as higher wages paid to care workers through the National Living Wage. However it finds that many of the recommendations, such as granting key worker status for care workers and developing a clear training and career pathway are still to be implemented. |
| Institute of Health and Social Care Management. A people plan for social care: a special insight. Social Care Innovators Subcommittee (2021). pp. 54, London, UK. https://ihscm.org.uk/wp-content/uploads/2021/12/A-People-Plan-for-Social-Care-A-special-Insight-November-2021-1.pdf | II- Research (Mixed method) | B- Social care people plan (England) | Theme 4- Policy, Research and Planning (identifying priorities for sector development) | - This People Plan takes a comprehensive view of the major issues facing the social care workforce and offers an appraisal of the current situation, examples of best practice, and recommendations for the future. - The plan covers five thematic areas: public perception of career image; training; recruitment; retention and staff wellbeing; pay and benefits. The Plan is the result of a four-month consultation with care managers, providers and leaders, and frontline staff by the Institute of Health and Social Care Management (IHSCM) and the IHSCM Social Care Innovators People Plan Sub-Committee. - There is a clear need for the improvement of the public perception and awareness of social care – without a positive and well-informed understanding of social care, recruitment and retention will continue to suffer; recruitment in social care does not have one quick fix, neither can it be remedied by one single entity – instead, it is going to require the coproduction and engagement of both central and local governments and care providers. - Retention may increase and improve through large sector wide changes – however, as the sector is made up predominantly of individual providers and large organisations, there are significant barriers to overcome to achieve these outcomes. - Training is an integral part of working in social care – it should promote and enable good and outstanding care and not detrimentally impact the morale of the workforce or put services under further pressures. - Pay scales and progression, public perception, funding and fee related issues, employment benefits and conditions of work were the most common themes when discussing the current situation of pay within the social care sector – in the long term, it is important to explore the creation of a centrally governed social care pension scheme, introduce pay and conditions banding for social care staff that mirrors that of NHS staff, and create a professional register of qualified social care staff. |
| Jarrín, O. F., Pouladi, F. A., & Madigan, E. A. (2019). International priorities for home care education, research, practice, and management: Qualitative content analysis. *Nurse education today*, *73*, 83–87. https://doi.org/10.1016/j.nedt.2018.11.020 | II- Research (Qual) | B- Priorities for home care sector education, research, practice, and management (USA) | Theme 4- Policy, Research and Planning (identifying priorities for sector development) | - Despite growing demand for home care nursing, there is a growing home care workforce shortage, due in part to hospital-centric nursing curricula that lead students to undervalue home care and community practice setting. - The objective of this study was to articulate an international vision for the future of home care education, research, practice, and management shared by experienced home care nurses working in leadership roles. Qualitative content analysis. The sample included 50 home care professionals from 17 countries. Home care nurse leaders (in education, research, practice, and management roles) were recruited through professional international nursing networks to participate in a structured online survey about priorities for the future of home care in 2014. Responses were open coded by two independent researchers. Preliminary categories and sub-themes were developed by the research team and revised after a modified member-checking process that included presentation and discussion of preliminary findings at three international nursing meetings in 2015 and 2016. - Four major themes emerged reflecting international priorities for the future of home care education, research, practice, and management: 1) Build the evidence base for home care; 2) Design better systems of care; 3) Develop leaders at all levels; and 4) Address payment and policy issues. - Collectively, the findings provide a major call to action for nurse educators to re-design existing pre- and post-licensure educational programs to meet the growing demand for home care nurses. Innovations in education that focus on filling gaps in the evidence-base for community nursing practice, and improving access to continuing education and evidence-based resources for practicing home care nurses and nurse managers should be prioritized. |
| Spetz, J., & Dudley, N. (2019). Consensus‐Based Recommendations for an Adequate Workforce to Care for People with Serious Illness. Journal of the American Geriatrics Society, 67(S2), S392-S399. | II- Research (Qual) | C- Priorities to develop community-based care (USA) | Theme 4- Policy, Research and Planning (identifying priorities for sector development) | - The lack of an adequately prepared workforce is a critical barrier to delivering high‐quality community‐based care for individuals living with serious illness. This article presents 16 consensus‐based recommendations to improve the capacity of the workforce in this area within the next 5 years, focusing on older adults. - The recommendations were developed at a summit of 40 national leaders from practice, payment, labor, advocacy, and research arenas. The consensus‐based recommendations include specific steps for geriatrics leaders including curriculum reforms to increase skills in the care of older and seriously ill populations, expanding experiential learning opportunities for students to increase interest in careers in geriatric and palliative care, developing and improving curriculum in interprofessional and cultural competency skills, preparing clinicians to support incorporation of home care aides and family caregivers as healthcare team members, development of skills to support shared decision making with patients, and requiring specific skills related to serious illness care in licensing, accreditation, and continuing education regulations. - Together, these recommendations put forward a charge to healthcare leaders to act to ensure a workforce that will optimize support for those with serious illness living in the community. J Am Geriatr Soc 67:S392–S399, 2019. |
| Koehler, I. (2014) Key to care: report of the Burstow Commission on the future of the home care workforce. Local Government Information Unit, London. pp 44. https://lgiu.org/wp-content/uploads/2014/12/Key-to-Care_FINAL-VERSION.pdf | III- Review of evidence to the commission | B- Assessment of home care provision and recommendations for change (England) | Theme 4- Policy, Research and Planning (identifying priorities for sector development) | - An assessment of home care provision, examining what needs to change to ensure a professional, well-paid, well-trained and properly regulated workforce who can provide the quality of care at home that people need. The report examines the life of home care workers considering what is like working in home care, wages and employment terms and conditions. It then provides a profile of home care, looking at access to care and future demand for services, and assesses the current commissioning system outlining what councils need to do to commission care better. - The report examines how to develop a career of esteem in home care, through the introduction of a licence to practise and a training and career pathway for care workers and discusses the role of design and technology in helping improve home care for the people who receive it and the people who deliver it. A final section sets out a vision for the future of the home care workforce. - Key recommendations: minimum payments for contact hours; moving away from time and task commissioning; a living wage for care workers; a licence to practise; a training and career pathway for care workers; and innovation and use of new technologies. - Recommends a training and career pathway for care workers: care workers in both health and social care need minimum standards of training which can be developed into pathways of specialism or the basis for further training and entry into allied care professions such as nursing or social work. A more formalised career path would include apprenticeships alongside clear career pathways. |
| Great Britain Parliament. (2022) House of Commons. Health and Social Care Committee. Workforce: recruitment, training and retention in health and social care: third report of session 2022-23. pp. 79. London, UK. https://publications.parliament.uk/pa/cm5803/cmselect/cmhealth/115/report.html | III- Review of policy and practice (committee) | B- Social care committee review of social care workforce (England) | Theme 4- Policy, Research and Planning (identifying priorities for sector development) | - This report outlines the scale of the health and social care workforce crisis, focusing on recruitment, training and retention. New research suggests the NHS in England is short of 12,000 hospital doctors and more than 50,000 nurses and midwives; evidence on workforce projections say an extra 475,000 jobs will be needed in health and an extra 490,000 jobs in social care by the early part of the next decade; hospital waiting lists reached a record high of nearly 6.5 million in April. - The report finds the Government to have shown a marked reluctance to act decisively. The refusal to do proper workforce planning risked plans to tackle the Covid backlog - a key target for the NHS. The value of continuity of care in social care settings, particularly amongst those who care for adults with autism or other severe learning disabilities, is a key issue, but it can only be addressed by improving retention in the sector. The report repeats a recommendation that annual funding for social care should be increased by £7 billion by 2023-24. This will account for demographic changes; uplift staff pay in line with the National Minimum Wage and protect people who face catastrophic social care costs. - The report argues that a long-term, sustainable strategy for the social care workforce is needed with the prospect of pay progression, professional development, and career pathways. New regulations should be introduced by 2023 in which care workers initially employed on zero-hours contracts are offered a choice of contract after three-months of employment. The new regulations should state that time allocated for travel and care must be clearly set out in the contracts of domiciliary care workers. - The Government should introduce an externally validated care certificate which is transferable between social care providers and between social care and the NHS. The Government should bring forward clear progression paths for those who want to continue professional development who enter social care and provide standardisation between training received in the sector to allow such progress and recognition. |
| Hamblin, K. and Manthorpe, J. (2018) How can we create better jobs in care? University of Sheffield. CIRCLE pp.4. Sheffield, UK. | V- Commentary | B- Ways to improve and enhance job quality in social care (England) | Theme 4- Policy, Research and Planning (identifying priorities for sector development) | - This Policy Perspective provides insights from expert contributors attending a roundtable discussion to explore how best to improve and enhance job quality in social care. Key issues identified included: low levels of pay, employment contracts which are often part-time or zero-hours; and lack of training and progression opportunities. - Recommendations for policy, employers and research include the need to reconceptualise social care as skilled work and to ensure the structures are in place to support recognised and valued training to facilitate horizontal and vertical career progression. It is one of seven Policy Perspectives produced as part of the Sustainable Care programme, led by Sheffield University, to explore issues relating to the sustainability of care arrangements in the UK. |
| Donovan, T. (2017) Ready, steady, go! Driving stability and innovation in the adult social care market. Association of Directors of Adult Social Services. pp. 8. London, UK. https://www.adass.org.uk/media/5861/new-dialogues-ready-steady-go-may-2017.pdf | V- Commentary | B- Social care people plan (England) | Theme 4- Policy, Research and Planning (identifying priorities for sector development) | - Presents the findings of a roundtable on the sustainability of the social care market in the context of an unprecedented pressure on local authorities’ finances, rising demand for services and the risks of market failure exemplified by the high-profile collapse of several care providers. The roundtable discussion identified many ways in which new thinking can tackle these challenges. - Making care work a more attractive vocational career option and appealing to pools of potential recruits, such as unemployed partners living on military garrisons, offer a way to deal with the workforce challenges. Working with housing and care providers more closely can help find new ways to deliver both the quality and quantity of accommodation and support adults need. The use of technology and care plans that give providers more scope to use the expertise they have are also promising. - Experiments such as Hampshire’s premium payments for higher need individuals are already delivering savings and better care. - Sustainability as more than an economic issue, that involves a new funding relationship between state, a family and the individual. - The paper concludes by arguing that while the challenge may be big the adult social care sector is anything but short of creative ideas on how to deliver change for the better. |
| Scales, K. (2022)  Transforming direct care jobs, reimagining long-term services and supports. Journal of the American Medical Directors Association, 23(2) pp.207-213.  https://www.jamda.com/article/S1525-8610(21)01056-2/fulltext | V- Commentary | B- Transformation of direct care jobs (USA) | Theme 4- Policy, Research and Planning (identifying priorities for sector development) | - The diverse array of individuals who receive long-term services and supports share one common experience, which is the need for assistance with personal care and/or other daily activities. The direct care workers (including nursing assistants, home health aides, and personal care aides) who provide this assistance play a critical role in keeping individuals safe, supporting their health and well-being, and helping prevent adverse outcomes. - Despite decades of research, advocacy, and incremental policy and practice reform, direct care workers remain inadequately compensated, supported, and respected. Long-standing direct care job quality concerns are linked to high turnover and job vacancy rates in this workforce, which in turn compromise the availability and quality of essential care for older adults and people with disabilities - which has never been more evident than during the COVID-19 pandemic. - This special article makes the case for transforming direct care jobs and stabilizing this workforce as a centrepiece of efforts to reimagine long-term services and supports system in the United States, as a public health priority, and as a social justice imperative. - Drawing on research evidence and examples from the field, the article demonstrates that a strong, stable direct care workforce requires: a competitive wage and adequate employment benefits for direct care workers; updated training standards and delivery systems that prepare these workers to meet increasingly complex care needs across settings, while also enhancing career mobility and workforce flexibility; investment in well-trained frontline supervisors and peer mentors to help direct care workers navigate their challenging roles; and an elevated position for direct care workers in relation to the interdisciplinary care team. - The article concludes by highlighting federal and state policy opportunities to achieve direct care job transformation, as well as discussing research and practice implications. |
| Timonen, V., & Lolich, L. (2019). "The Poor Carer": Ambivalent Social Construction of the Home Care Worker in Elder Care Services. Journal of gerontological social work, 62(7), 728–748. https://doi.org/10.1080/01634372.2019.1640334 | II- Research (Qual) | A- Social construction of the home care worker role (Ireland) | Theme 4- Policy, Research and Planning (Lack of occupational status) | - This article examines the social construction of the home care worker from the perspective of various professionals in the elder care sector in Ireland. The research, using the Grounded Theory method, involved focus groups with 31 participants comprising health and social work professionals as well as care agency managers and policy planners. - The social construction of the elder care worker is characterised by ambivalence. We connect the concept of ambivalence at the micro level of human relationships to structural factors that are driving the ambivalence. Ambivalence towards home care workers is shaped by structural factors including the precariousness of care work, the commodification of time, and the stipulated personalisation of services. The irreconcilable contrasts between portrayals of care workers as both 'good' and 'bad' are indicative of deep contradictions in the expectations that contemporary care systems direct at paid caregivers. - Ambivalence arises from the commodified and dispensable status of care workers, and fundamental transformations in their training, working conditions and pay are required to move away from this ambivalence and towards care workers' equal status with professionals in the care sector. |
| Nisbet, E., & Morgan, J. C. (2021). Constructing legality: theorizing work and the challenges for mobilization of home care workers. *International Journal of Care and Caring*, *5*(1), 45-63. | V- Comment | C- Training standards for health and personal care aides (USA) | Theme 4- Policy, Research and Planning (legality of home care work and roles) | - Aides providing long-term care in home- and community-based settings in the US experience low wages and poor conditions. Worker advocates seek to improve care jobs through policy change, a multifaceted endeavour given the complexity of health and labour policies in a federal system. - This theoretical overview draws on labour process theory to delineate domains of work and legal consciousness theory to highlight challenges related to ‘sources of authority’ governing each domain. For the context of publicly funded care provided through agencies, the authors identify aide actions related to these sources that could support rights claiming and suggest research to inform policy improvements. |
| Molyneux, P. (2021) Career opportunities: understanding the opportunity to develop the health and care workforce together  Housing Associations’ Charitable Trust (HACT)  pp. 16. London, UK. https://www.housinglin.org.uk/_assets/Resources/Housing/OtherOrganisation/Report_CareerOpportunitiesHACTRegenda.pdf | III- Review of evidence (white paper) | B- The role of housing associations in supporting recruitment to care jobs locally (England) | Theme 4- Policy, Research and Planning (local community, place-based approaches to home care, working with anchor institutions such as housing associations) | - This paper makes the case for all anchor institutions, including health and care providers and housing associations, working across a system to come together with their partners, suppliers and contractors to ensure that they fully understand the local labour market, have strong place-based leadership, share workforce, work together to improve access and promote the concept of good work. Housing associations act as anchor institutions and are well placed to work with local health and care organisations to co-develop workforce strategies with the explicit intention of supporting local recruitment and developing the current and future health and social care workforce. - The immediate opportunity of directing employment programmes for local people needs to evolve into strategic alliances around all workforce issues. There is considerable opportunity to build longer-term collaboration through shared workforce development, that enhances leadership across systems and taps into all the resources and talents in our organisations and communities. |
| Manchha, A. V., Way, K. A., Thai, M., & Tann, K. (2023). "The Stigma is what you do": Examining the Relationship Between Occupational Stigma and Worker Outcomes in the Aged Care Context. Journal of applied gerontology : the official journal of the Southern Gerontological Society, 42(2), 221–230. https://doi.org/10.1177/07334648221129849 | II- Research (Mixed method) | B- Stigma of aged care work (Australia) | Theme 4- Policy, Research and Planning (occupational stigma) | - Negative social evaluations about aged-care work continue to discredit those who perform this type of work. Drawing on 'dirty work' literature from the field of management and stigma theory from the field of psychology, this study examines occupational stigma in the context of aged-care work and its relationship with negative outcomes for people who work in aged care. - Findings from a path analysis revealed that aged-care workers (n = 185) who ascribe occupational taints and poor occupational conditions with aged-care work perceive aged-care work as being stigmatized, and in turn, are more likely to internalize this stigma. Through this process of perceiving and internalizing occupational stigma, aged-care workers experience greater psychological distress, job dissatisfaction, and turnover intentions. This research fills a void within the gerontology literature regarding the psychological processes underlying how occupational stigma may come to negatively impact this workforce. Findings offer practical implications for policy reform and managerial training. |
| Manchha, A. V., Way, K. A., Tann, K., & Thai, M. (2022). The Social Construction of Stigma in Aged-Care Work: Implications for Health Professionals' Work Intentions. *The Gerontologist*, *62*(7), 994–1005. https://doi.org/10.1093/geront/gnac002 | II- Research (Mixed-method) | B- Stigma of aged care work (Australia) | Theme 4- Policy, Research and Planning (occupational stigma) | - Although society has cultivated a deeper appreciation for essential health services, societal discourses reinforce a stigma of working in aged care. Drawing on dirty work and Stigma Theory, this study aims to investigate stigma in the context of recruiting health professionals. - The study employed a mixed-methods design to examine the nature and implications of the stigma of working in aged care. A path analysis was used to test whether health professionals' (n = 159) negative perceptions of aged-care work would negatively predict their willingness to work in aged care. A linguistic analysis was conducted to understand how health professionals' (n = 168) use of language positions themselves toward or away from engaging in aged-care work. - Quantitative findings revealed that perceptions of physical taint directly predicted lower willingness to perform aged-care work. Perceptions of social taint, moral taint, and poor occupational conditions negatively predicted willingness to work in institutional aged care, indirectly via social devaluation. Findings from the linguistic analysis demonstrated that health professionals (re)produce stigma through aligning themselves with devaluing discourses about aged-care workers, work, and institutions. - This study provides insight about the role that stigma plays in the aged-care recruitment crisis, with implications for aged-care institutions. Societal discourse may obstruct the employment of health professionals in aged care because it can (re)produce the stigma of working in aged care. Recommendations for ways to reduce the impact of this stigma include public messaging and training. |
| Manchha, A. V., Walker, N., Way, K. A., Dawson, D., Tann, K., & Thai, M. (2021). Deeply Discrediting: A Systematic Review Examining the Conceptualizations and Consequences of the Stigma of Working in Aged Care. *The Gerontologist*, *61*(4), e129–e146. https://doi.org/10.1093/geront/gnaa166 | III- Review of literature | B- Stigma of aged care work (Australia) | Theme 4- Policy, Research and Planning (occupational stigma) | - The stigma of working in aged care can discredit and devalue those working in gerontology. This overlooked workforce issue may underpin complex staffing challenges like chronic worker shortages and inadequate care delivery. Our review synthesizes the existing literature and introduces a conceptual framework based on linguistics to reconcile disparate conceptualizations and negative consequences of this stigma. - The team conducted a systematic review and assessed peer-reviewed articles published from 1973 to 2019 across 5 databases. Fifty-nine articles were selected based on criteria grounded in stigma theory. - Only 10 articles explicitly used the term "stigma" when conceptualizing the stigma of working in aged care. An additional 49 articles conceptualized this stigma in terms of stigma processes (e.g., status loss). Findings from a deeper examination using a linguistic analysis revealed societal groups predominantly conceptualized stigma in 3 distinct ways based on (a) unfavorable character judgment of aged care workers, (b) lower value placed on aged care work, and (c) negative emotional reactions towards working in aged care. Last, stigma was associated with adverse psychological and job-related consequences. - Reconceptualizing this workforce issue and recognizing it as a societal challenge will enable policymakers to design evidence-based interventions at industry and societal levels. We propose workforce challenges in the aged care sector such as attraction, retention, and well-being may lessen with interventions aimed at mitigating the stigma of working in aged care. |
| Costa, C., Girace, E., Fanton, E., Stevanin, S., Tasca, T., & Mantoan, D. (2019). [National survey to verify the implementation of State-Regions agreements introducing the professional profile of health social care worker and instituting a complementary training program for nursing assistants, Italy]. *Igiene e sanita pubblica*, *75*(3), 201–210. | V- Commentary | C- Development of the nursing assistant role and competencies (Italy) | Theme 4- Policy, Research and Planning (professional core competencies and training requirements) | - The professional profile of health social care worker (OSS) was introduced in Italy in 2001 and has become a fundamental figure in the Italian healthcare and social-health services system. However, changes have occurred in Italian health, clinical care and social realities since the OSS profile was defined. - This article reports the results of a national-level study to investigate the state of application of the State-Regions Agreement of 22 February 2001, establishing the professional profile of OSS and regulating its characteristics and competencies, as that of the agreement of 16 January 2003, regulating the complementary healthcare training for nursing assistants. |
| Poh, L., Tan, S. Y., & Lim, J. (2021). Governance of Assisted Living in Long-Term Care: A Systematic Literature Review. *International journal of environmental research and public health*, *18*(21), 11352. https://doi.org/10.3390/ijerph182111352 | III- Review of literature | B- Levels of regulation in assisted living model (worldwide) | Theme 4- Policy, Research and Planning (regulation systems, reporting requirements and quality control) | - Assisted living (AL) is an emerging model of care in countries where long-term care needs are escalating, with emphasis given to promoting independence and autonomy among the residents to achieve active and healthy ageing. Unlike established nursing homes, the governance of AL is nebulous due to its novelty and diverse nature of operations in many jurisdictions. A comprehensive understanding of how AL is governed globally is important to inform regulatory policies as the adoption of AL increases. - A systematic literature review was undertaken to understand the different levels of regulations that need to be instituted to govern AL effectively. A total of 65 studies, conducted between 1990 to 2020, identified from three major databases (PubMed, Medline, and Scopus), were included. - Thematic synthesis analytical approach identified macro-level regulations (operational authorization, care quality assessment and infrastructural requirements), meso-level regulations (operational management, staff management and distribution, service provision and care monitoring, and crisis management), and micro-level regulations (clear criteria for resident admission and staff hiring) that are important in the governance of AL. - Argues that large-scale adoption of AL without compromising the quality, equity and affordability would require clear provisions of micro, meso- and macro-level regulations. |
| Luijnenburg, O., Manthorpe, J., Samsi, K. (2022)  Skills at the heart of care: a scoping review of evidence on skills gaps in the social care workforce  NIHR Policy Research Unit in Health and Social Care Workforce, The Policy Institute, King’s College London. pp. 64. London, UK. https://kclpure.kcl.ac.uk/ws/portalfiles/portal/172111450/Luijnenburg_et_al_2022_Scoping_review_skills_gaps.pdf | III- Review of literature | B- Skills gaps in the adult social care workforce (England) | Theme 4- Policy, Research and Planning (research gaps around skills levels and gaps, effective support for client groups) | - This report sets out the findings of a scoping review highlighting areas where there are particular claims as to skills gaps in the adult social care workforce. It has necessarily focused on gaps rather than the presence of skills. - Some of the gaps may be surprising, such as lack of evidence about skills in supporting stroke survivors, people living with diabetes and people with hearing loss or impairment. Others show how change has occurred, such as the greater investment in dementia care and thinking about this as part of the wider skills agenda in ASC. - Other innovations have included mandatory obligations, which may be particularly useful in areas of great concern. There is room for evidence about 'basic skills' in terms of literacy and numeracy among the ASC workforce and any measures that are needed here. This would enhance digital skills but also enable ASC staff to participate in other forms of training and career development. - The review has highlighted the lack of 'voice' in such debates and delivery from people using ASC services and carers. This needs to be rectified. - Overall, much research calls for 'greater skills' and 'more training'. Few studies have considered how such recommendations fit with the current training system and different skills levels. Few provide evidence of cost-effectiveness. - The creation of a Knowledge and Skills Framework and other commitments outlined in People at the Heart of Care (DHSC 2021) will need to consider the balance of general and specialist skills, especially in the context of a population with multiple long-term care needs as well as needs for rehabilitation and short-term support. The research reported here also highlights the need for any skills developments to be considered not solely as a means to avoid NHS treatment or support but to add quality to life overall. |
| Darby Rauch, K., (2014) Finding and Training a Workforce That Can Address Older Adults' Long-Term Care Needs. Science of Caring. University of California. https://nursing.ucsf.edu/scienceofcaring/news/finding-and-training-workforce-can-address-older-adults-long-term-care-needs | V- Commentary | B- Workforce research (USA) | Theme 4- Policy, Research and Planning (research groups and networks to inform policy and practice) | - Article describing UC San Francisco School of Nursing and its partners across the medical center are home to a new national research center aimed at informing policymakers about how to address this potential long-term care crisis. UCSF’s Health Workforce Research Center is one of four centers nationwide scrutinizing different aspects of the workforce needs of an aging population. |
| Tsui, E. K., LaMonica, M., Hyder, M., Landsbergis, P., Zelnick, J., & Baron, S. (2021). Expanding the Conceptualization of Support in Low-Wage Carework: The Case of Home Care Aides and Client Death. International journal of environmental research and public health, 19(1), 367. https://doi.org/10.3390/ijerph19010367 | II- Research (Qual) | B- Occupational risks and stress in home care (USA) | Theme 4- Policy, Research and Planning (research on policy and community-level support for home support workers) | - Home care aides are a rapidly growing, non-standard workforce who face numerous health risks and stressors on the job. While research shows that aides receive limited support from their agency employers, few studies have explored the wider range of support that aides use when navigating work stress and considered the implications of these arrangements. - To investigate this question, this study involved 47 in-depth interviews with 29 home care aides in New York City, focused specifically on aides' use of support after client death. Theories of work stress, the social ecological framework, and feminist theories of care informed our research. - The analysis demonstrates aides' extensive reliance on personal sources of support and explores the challenges this can create in their lives and work, and, potentially, for their communities. - The authors also document aides' efforts to cultivate support stemming from their home-based work environments. Home care aides' work stress thus emerges as both an occupational health and a community health issue. While employers should carry responsibility for preventing and mitigating work stress, moving toward health equity for marginalized care workers requires investing in policy-level and community-level supports to bolster employer efforts, particularly as the home care industry becomes increasingly fragmented and non-standard. |
| Markkanen, P., Quinn, M., Galligan, C., Sama, S., Brouillette, N., & Okyere, D. (2014). Characterizing the nature of home care work and occupational hazards: a developmental intervention study. *American journal of industrial medicine*, *57*(4), 445–457. https://doi.org/10.1002/ajim.22287 | II- Research (Qual) | B- Occupational safety in home care (USA) | Theme 4- Policy, Research and Planning (research to assess staff and client safety together) | - Home care (HC) aide is the fastest growing occupation, yet job hazards are under-studied. - This study documents the context of HC aide work, characterizes occupational safety and health (OSH) hazards, and identifies preventive interventions using qualitative methods. The study involved 12 focus groups among aides and 26 in-depth interviews comprising 15 HC agency, union, and insurance company representatives as well as 11 HC recipients in Massachusetts. All focus groups and interviews were audio-recorded, transcribed, and coded with NVIVO software. - Major OSH concerns were musculoskeletal disorders from client care tasks and verbal abuse. Performing tasks beyond specified job duties may be an OSH risk factor. HC aides' safety and clients' safety are closely linked. Client handling devices, client evaluation, care plan development, and training are key interventions for both aides' and clients' safety. |
| Quinn, M. M., Markkanen, P. K., Galligan, C. J., Sama, S. R., Kriebel, D., Gore, R. J., Brouillette, N. M., Okyere, D., Sun, C., Punnett, L., Laramie, A. K., & Davis, L. (2016). Occupational health of home care aides: results of the safe home care survey. *Occupational and environmental medicine*, *73*(4), 237–245. https://doi.org/10.1136/oemed-2015-103031 | II- Research (Quant) | B- Home care worker occupational safety and health (USA) | Theme 4- Policy, Research and Planning (research to assess staff and client safety together) | - In countries with ageing populations, home care (HC) aides are among the fastest growing jobs. There are few quantitative studies of HC occupational safety and health (OSH) conditions. - The objectives of this study were to: (1) assess quantitatively the OSH hazards and benefits for a wide range of HC working conditions, and (2) compare OSH experiences of HC aides who are employed via different medical and social services systems in Massachusetts, USA. - HC aides were recruited for a survey via agencies that employ aides and schedule their visits with clients, and through a labour union of aides employed directly by clients or their families. The questionnaire included detailed questions about the most recent HC visits, as well as about individual aides' OSH experiences. - The study population included 1249 HC aides (634 agency-employed, 615 client-employed) contributing information on 3484 HC visits. Hazards occurring most frequently related to musculoskeletal strain, exposure to potentially infectious agents and cleaning chemicals for infection prevention and experience of violence. Client-hired and agency-hired aides had similar OSH experiences with a few exceptions, including use of sharps and experience of verbal violence. - The OSH experience of HC aides is like that of aides in institutional healthcare settings. Despite OSH challenges, HC aides enjoy caring for others and the benefits of HC work should be enhanced. Quantification of HC hazards and benefits is useful to prioritize resources for the development of preventive interventions and to provide an evidence base for policy-setting. |
| Grønoset Grasmo, S., Frostad Liaset, I., & Redzovic, S. E. (2021). Home health aides' experiences of their occupational health: a qualitative meta-synthesis. *Home health care services quarterly*, *40*(2), 148–176. https://doi.org/10.1080/01621424.2021.1921650 | III- Review of Literature | B- Occupational safety in home care (Norway) | Theme 4- Policy, Research and Planning (research to assess staff and client safety together) | - Home health aides (HHA) have high sickness absence while the need for home care services is rapidly growing. - The aim of this study was to derive new conceptual understandings by identifying, describing and interpreting key concepts across qualitative studies on how HHA experience their occupational health related to their working conditions. A qualitative ethnographic meta-synthesis was used as a method to analyze 27 articles included from systematic searches in CINAHL, MEDLINE and PsycINFO. - HHA experience physical strenuous work task demands in combination with unfortunate organizational conditions in an uncontrolled and ever-changing psychosocial and physical working environment as the main obstacle to their occupational health, although many positive presence factors with opposite effects were reported. - More research is needed to investigate whether physical demanding work tasks can have positive effects on HHA's occupational health by reorganizing their work while preserving patients' empowerment at their home. |
| Quinn, M. M., Markkanen, P. K., Galligan, C. J., Sama, S. R., Lindberg, J. E., & Edwards, M. F. (2021). Healthy Aging Requires a Healthy Home Care Workforce: the Occupational Safety and Health of Home Care Aides. *Current environmental health reports*, *8*(3), 235–244. https://doi.org/10.1007/s40572-021-00315-7 | III- Review of the literature | B- Home care aide occupational safety (USA) | Theme 4- Policy, Research and Planning (research to assess staff and client safety together) | - The purpose of this review was to identify important home care (HC) aide occupational safety and health (OSH) hazards and examine how addressing these can improve aide health and the delivery of HC services overall. Specifically, the review seeks to answer: Why is HC aide OSH important? What are the most significant OSH challenges? How can improving HC aide OSH also improve the safety and health of their clients? What implications do the findings have for future research? - HC is one of the fastest growing US industries. Aides comprise its largest workforce and are increasingly needed to care for the rapidly aging population. There is an aide shortage due in part to instabilities in HC work organization and to serious job-specific hazards, resulting in aides losing work time. - Recent social, economic, and technological factors are rapidly changing the nature of HC work, creating OSH hazards similar to those found in nursing homes. At the same time, aides are experiencing social and economic inequities that increase their vulnerability to OSH hazards. These hazards are also a burden on employers who are challenged to recruit, retain, and train aides. OSH injuries and illness interrupt the continuity of care delivery to clients. Many OSH hazards also put HC clients and families at risk. - A new framework and methodologies are needed to assess aide and client safety together in order to guide future HC research, policies, and practices. Government, industry, and labor commitment is needed to fund and coordinate a comprehensive, multidisciplinary research program. |
| Cataldo, F., Kielmann, K., Kielmann, T., Mburu, G., & Musheke, M. (2015). 'Deep down in their heart, they wish they could be given some incentives': a qualitative study on the changing roles and relations of care among home-based caregivers in Zambia. BMC health services research, 15, 36. https://doi.org/10.1186/s12913-015-0685-7 | II- Research (Qual) | B- HIV caregiving in home care (Sub-Saharan Africa) | Theme 4- Policy, Research and Planning (shifts in social and professional relations) | - Across Sub-Saharan Africa, the roll-out of antiretroviral treatment (ART) has contributed to shifting HIV care towards the management of a chronic health condition. While the balance of professional and lay tasks in HIV caregiving has been significantly altered due to changing skills requirements and task-shifting initiatives, little attention has been given to the effects of these changes on health workers' motivation and existing care relations. - This paper draws on a cross-sectional, qualitative study that explored changes in home-based care (HBC) in the light of widespread ART rollout in the Lusaka and Kabwe districts of Zambia. Methods included observation of HBC daily activities, key informant interviews with programme staff from three local HBC organisations (n = 17) and ART clinic staff (n = 8), as well as in-depth interviews with home-based caregivers (n = 48) and HBC clients (n = 31). - Results show that since the roll-out of ART, home-based caregivers spend less time on hands-on physical care and support in the household, and are increasingly involved in specialised tasks supporting their clients' access and adherence to ART. Despite their pride in gaining technical care skills, caregivers lament their lack of formal recognition through training, remuneration or mobility within the health system. Care relations within homes have also been altered as caregivers' newly acquired functions of monitoring their clients while on ART are met with some ambivalence. Caregivers are under pressure to meet clients and their families' demands, although they are no longer able to provide material support formerly associated with donor funding for HBC. - As their responsibilities and working environments are rapidly evolving, caregivers' motivations are changing. It is essential to identify and address the growing tensions between an idealized rhetoric of altruistic volunteerism in home-based care, and the realities of lay worker deployment in HIV care interventions that not only shift tasks but transform social and professional relations in ways that may profoundly influence caregivers' motivation and quality of care. |
| Gary, M., & Berlinger, N. (2023). Centering Home Care in Bioethics Scholarship, Education, and Practice. *The Hastings Center report*, *53*(3), 34–36. https://doi.org/10.1002/hast.1488 | V- Commentary | B- Home care (USA) | Theme 4- Policy, Research and Planning (systems thinking, systemic challenges and change) | - This commentary responds to "Home Care in America: The Urgent Challenge of Putting Ethical Care into Practice," by Coleman Solis and colleagues, in the May-June 2023 issue of the Hastings Center Report. More specifically, the commentary responds to the authors' call for "inquiry into the nature, value, and practice" of home care. - The authors argue that the most urgently needed normative reset for thinking about care work is the replacement of dominant individualistic thinking with systemic thinking. Deepening a focus on the social, economic, and historical forces that shape the state of contemporary care work will help bioethicists to argue more effectively for improvements to working conditions. In turn, better working conditions will ease the oppositional stance between caregivers and receivers that has been set up by the current system, enabling all parties involved to better pursue the feminist ethical ideal of care. |
| Chabouh, S., El-Amraoui, A., Hammami, S., & Bouchriha, H. (2023). A systematic review of the home health care planning literature: Emerging trends and future research directions. Decision Analytics Journal, 100215. | II- Research (Model) | B- Staffing in home care (Tunisia) | Theme 4- Policy, Research and Planning (the need for planning levels linked to outcomes) | - Patients are traditionally treated in hospitals and healthcare facilities. However, many care services known as Home Health Care (HHC) are provided at patients’ homes. Despite the growing trend in life expectancy in developing countries, the number of health facilities and resources is not increasing at a similar rate. Therefore, the need for HHC is increasing worldwide. Over the last decade, research on operations management and planning in the HHC industry has received increased attention. - This paper presents a systematic literature review to analyse the research on HHC management practices. The emphasis has been given to research papers considering uncertainty. The studied articles are assigned to research themes defined for each planning level. Each theme is discussed while identifying emerging trends and future research directions. The findings of this review can be used as the basis for HHC operational, tactical, and strategic planning and related topics. |
| Friedman, E. M., Ghosh-Dastidar, M., Ruder, T., Siconolfi, D., & Shih, R. A. (2021). Trends In Home Care Versus Nursing Home Workforce Sizes: Are States Converging Or Diverging Over Time? Study examines trends in home care versus nursing home workforce sizes. *Health Affairs*, *40*(12), 1875-1882. | II- Research (Quant) | B- State-level size variation of the home care workforce (USA) | Theme 4- Policy, Research and Planning (workforce data) | - There is strong preference among people with disabling conditions to receive care at home rather than in an institutional setting. Differences in state policies may make this more feasible in some states than others. Yet no study to date has examined trends in the long-term care workforce across states. - Using state-level data on direct care workers from the period 2009–20, this study examined trends in the sizes of the nursing home and home care workforces. - Since 2009 most states have increased the size of their home care workforces and decreased the size of their nursing home workforces, but there is substantial variation across states in the magnitude of these changes. In addition, the gap between leading and lagging states in home care workforce size has grown over time. This suggests that more targeted efforts may be needed to ensure that people with disabling conditions can have their needs met in their desired setting across the nation. |
| Work Foundation Total Jobs (2021) Social Care: a guide to attracting and retaining a thriving workforce. pp20. London, UK. https://www.lancaster.ac.uk/media/lancaster-university/content-assets/documents/lums/work-foundation/reports/SocialCareGuide.pdf | II- Research (Mixed method) | B- Tackling workforce challenges in adult social care (England) | Theme 4- Policy, Research and Planning (workforce planning) | - A guide to tackling workforce challenges in adult social care, based on output from a mixed-methods approach, including: an evidence review, semi-structured interviews with policy stakeholders and employers; and two surveys – a carers survey and a candidate survey. The sector faces long-standing challenges which have been made more acute by the pandemic and recent changes to immigration policy. - From attracting individuals with the right qualities to retaining skilled staff and developing routes to progression, care providers are struggling to build and sustain their workforce. The guide aims to support care providers navigating these challenges, highlighting key insights from our research and offering recommendations for employers and government to create long-term solutions for a thriving workforce. The research findings show that a third (31%) of jobseekers are considering a career in care; younger candidates are most likely to be planning to pursue careers in care, with one in four (25%) 16–25-year-olds expecting to pursue a career in the sector in the near future; analysis of Totaljobs candidates found that the number of people applying for social care roles has increased by 39% between 2019 and 2021, with 56% of new starters in care joining from other sectors. - The report makes a number of recommendations, including: create opportunities for young people to build an understanding of care work; adopt a values-based approach to recruiting care workers, for example through using scenario-based questions, or group assessment days; consult with their workforce to understand the rewards and benefits they would value most, and use this insight to develop a benefits package that aligns with staff preferences; social care sector bodies and regulators should coordinate with central Government and national governments to create a sector-wide, long-term strategy for workforce development including creating a Continuing Professional Development Framework well supported by funding. |
| Association of Directors of Adult Social Services. (2021) The legacy of Covid: what might the pandemic’s long-term impact be for Adult Social Care? Eastern Region, England. pp24. London, UK. https://www.scie-socialcareonline.org.uk/the-legacy-of-covid-what-might-the-pandemics-long-%C2%ADterm-impact-be-for-adult-social-care/r/a116f00000Um0yUAAR | II- Research (Mixed method) | B- Long-term impact of Covid-19 on the adult social care sector (England) | Theme 4- Public Health, Disaster Preparedness, Crisis Management (frontline essential care, learning from Covid-19) | - In considering the long-term impact of Covid, this report addresses both the legacy of the disease and the policy response in the UK – in particular, the effect of lockdowns. It focuses on the economy and employment; the political landscape; the social and cultural landscape; health and wellbeing; long-term impact of Covid on Adult Social Care, looking specifically at markets, workforce, interface with the NHS, quality and safeguarding, inequalities, carers, the voluntary and community sector (VCS), and digital technology. - Key highlights include: the economy will recover, but this resurgence will not return us to a pre-Covid world and will be uneven; the political, social and cultural landscape has changed – the need to protect people from infection will need to be balanced against the need to safeguard personal freedoms; health outcomes will take time to improve and this will have a huge impact on demand for Adult Social Care; the experience of Covid is accelerating the desire for home-based solutions, supported by technology and away from residential care; the need to improve recruitment, retention and career structures for the workforce has become more urgent, and looks set to be a long-term challenge; the pandemic’s impact has highlighted to the public the need for reform and sustainable funding for Adult Social Care, but the needs of working age adults and of preventing hospital admissions in the first place remain less well recognised; in the longer-term, the legacy of Covid may be to permanently widen and entrench existing inequalities; digital solutions offer opportunities that the pandemic has shown can lead to rapid innovation, but there is also a need to address risks and support person-centred and relationship-based planning; communities can provide vital support to connect and support people, with the voluntary sector a key partner with health and care services, if these can be appropriately supported. |
| Sterling, M. R., Tseng, E., Poon, A., Cho, J., Avgar, A. C., Kern, L. M., Ankuda, C. K., & Dell, N. (2020). Experiences of Home Health Care Workers in New York City During the Coronavirus Disease 2019 Pandemic: A Qualitative Analysis. *JAMA internal medicine*, *180*(11), 1453–1459. https://doi.org/10.1001/jamainternmed.2020.3930 | II- Research (Qual) | B- Care workers during Covid-19 (USA) | Theme 4- Public Health, Disaster Preparedness, Crisis Management (frontline essential care, learning from Covid-19) | - Home health care workers care for community-dwelling adults and play an important role in supporting patients with confirmed and suspected coronavirus disease 2019 (COVID-19) who remain at home. These workers are mostly middle-aged women and racial/ethnic minorities who typically earn low wages. Despite being integral to patient care, these workers are often neglected by the medical community and society at large; thus, developing a health care system capable of addressing the COVID-19 crisis and future pandemics requires a better understanding of the experiences of home health care workers. - This study aimed to understand the experiences of home health care workers caring for patients in New York City during the COVID-19 pandemic. From March to April 2020, a qualitative study with 1-to-1 semi structured interviews of 33 home health care workers in New York City was conducted in partnership with the 1199SEIU Home Care Industry Education Fund, a benefit fund of the 1199 Service Employees International Union United Healthcare Workers East, the largest health care union in the US. Purposeful sampling was used to identify and recruit home health care workers. Audio-recorded interviews were professionally transcribed and analyzed using grounded theory. Major themes and subthemes were identified. - In total, 33 home health care workers employed by 24 unique home care agencies across the 5 boroughs of New York City participated. Participants had a mean (SD) age of 47.6 (14.0) years, 32 (97%) were women, 21 (64%) were Black participants, and 6 (18%) were Hispanic participants. - Five major themes emerged: home health care workers (1) were on the front lines of the COVID-19 pandemic but felt invisible; (2) reported a heightened risk for virus transmission; (3) received varying amounts of information, supplies, and training from their home care agencies; (4) relied on nonagency alternatives for support, including information and supplies; and (5) were forced to make difficult trade-offs in their work and personal lives. - In this qualitative analysis, home health care workers reported providing frontline essential care, often at personal risk, during the COVID-19 pandemic. They experienced challenges that exacerbated the inequities they face as a marginalized workforce. Interventions and policies to better support these frontline health care professionals are urgently needed. |
| Lethin, C., Kenkmann, A., Chiatti, C., Christensen, J., Backhouse, T., Killett, A., & Malmgren Fänge, A. (2021, June). Organizational support experiences of care home and home care staff in Sweden, Italy, Germany and the United Kingdom during the COVID-19 pandemic. In *Healthcare* (Vol. 9, No. 6, p. 767). MDPI. | II- Research (Qual) | B- Healthcare worker Covid-19 related stress and anxiety | Theme 4- Public Health, Disaster Preparedness, Crisis Management (frontline essential care, learning from Covid-19) | - The COVID-19 pandemic has affected care workers all over the globe, as older and more vulnerable people face a high risk of developing severe symptoms and dying from the virus infection. The aim of this study was to compare staff experiences of stress and anxiety as well as internal and external organizational support in Sweden, Italy, Germany, and the United Kingdom (UK) in order to determine how care staff were affected by the pandemic. - A 29-item online questionnaire was used to collect data from care staff respondents: management (n = 136), nurses (n = 132), nursing assistants (n = 195), and other healthcare staff working in these organizations (n = 132). Stress and anxiety levels were highest in the UK and Germany, with Swedish staff showing the least stress. Internal and external support only partially explain the outcomes. Striking discrepancies between different staff groups' assessment of organizational support as well as a lack of staff voice in the UK and Germany could be key factors in understanding staff's stress levels during the pandemic. Structural, political, cultural, and economic factors play a significant role, not only factors within the care organization or in the immediate context. |
| Franzosa, E., Wyte-Lake, T., Tsui, E., Reckrey, J., Sterling, M. (2022) Essential but Excluded: Building Disaster Preparedness Capacity for Home Health Care Workers and Home Care Agencies. Journal of the American Medical Directors Association, 23, 12, 1990-1996. https://doi.org/10.1016/j.jamda.2022.09.012. | V- Commentary | B- Home care sector involvement in disaster preparation (USA) | Theme 4- Public Health, Disaster Preparedness, Crisis Management (public health crises, impact and learning from Covid-19) | - COVID-19 has demonstrated the essential role of home care services in supporting community-dwelling older and disabled individuals through a public health emergency. As the pandemic overwhelmed hospitals and nursing homes, home care helped individuals remain in the community and recover from COVID-19 at home. Yet unlike many institutional providers, home care agencies were often disconnected from broader public health disaster planning efforts and struggled to access basic resources, jeopardizing the workers who provide this care and the medically complex and often marginalized patients they support. - The exclusion of home care from the broader COVID-19 emergency response underscores how the home care industry operates apart from the traditional health care infrastructure, even as its workers provide essential long-term care services. This special article (1) describes the experiences of home health care workers and their agencies during COVID-19 by summarizing existing empiric research; (2) reflects on how these experiences were shaped and exacerbated by longstanding challenges in the home care industry; and (3) identifies implications for future disaster preparedness policies and practice to better serve this workforce, the home care industry, and those for whom they care. |
| Ramos LR, d'Orsi E, Simões EJ. Editorial: Longevity with functionality and quality of life. Front Aging. 2023 Nov 15;4:1281737. doi: 10.3389/fragi.2023.1281737. PMID: 38034418; PMCID: PMC10684901. | III- Review of research topic | B- Longevity and successful ageing (worldwide) | Theme 4- Sector Economic Perspective (socioeconomic and health implications of longevity and public health) | - This editorial explains how the world is facing a longevity revolution, with an ever-growing aged population, with important socioeconomic and health implications. A large proportion of this elderly population has a chronic health condition without a cure, which may lead to disability. The concept of successful aging is associated with being able to live an independent and autonomous life, not necessarily free of any disease. - Public health in this paradigm should look at health promoting interventions that prevent functional loss both in physical and mental terms thus promoting quality of life. The objective of this Research Topic was to gather evidence on risk factors for loss of functional capacity and effective interventions to preserve or improve functional capacity of the elderly on a population basis. |
| Velazquez, F., Fox, S., & Chatha, D. (2022). Adaptive Strategies to Building a Sustainable Workforce: Health Care Support Worker (HCSW) and the Health Career Access Program (HCAP). International Journal of Integrated Care (IJIC), 22. | II- Programme evaluation | B- Large-scale career building strategy for health care careers (Canada) | Theme 4- Sector Economic Perspectives (cost-benefit of large-scale policy-directives or workforce development interventions) | - The COVID-19 pandemic has had a tremendous impact on organizations. Health care in particular has been affected in many ways. Health care professionals have been working around the clock to support patients and families during these tumultuous times. It is well known that these demands have taken a significant toll on staff which has led to burnout. To ensure that the health care workforce remains strong, the British Columbia Government has implemented the Health Career Access Program (HCAP). Fraser Health coordinates the management of HCAP in the region that it serves (1.8 million people, 1 out of 3 British Columbians). - The British Columbia Government opted to introduce a new career building strategy in the midst of a pandemic. Through HCAP, individuals have the opportunity to apply to become a Health Care Support Worker (HCSW). The HCSW role is an entry-level position that provides non-direct care to residents in AL and LTC. While working, HCSWs are afforded the opportunity to go to school to become health care assistants, with all costs covered by the Government, including their salary. The Return of Service agreement ensures that staff remain and there is stability and sustainment in the workforce. - Using key change management principles, the Fraser Health HCAP Project Team has been supporting the HCSW, the care team, as well as residents and families. Focused on building a team-based care environment the project team creates opportunities for open dialogue and engagement. This supports building "Awareness" and "Desire." Preparing resources such as the "HCSW Can Do-Can Not Do" list in addition to the shadow-shifts and buddy-pairing amongst other tactics, allow for the "Knowledge" and "Ability" to show up. By investing in, and supporting the growth of an individual, there is emphasis on sustainment and longevity (Reinforcement). - Although early in the implementation phase, the feedback has been positive from both the provider and resident-experience. A Logic Model is being built to measure the true impact of the program, and how the model can inform the future of the provision of AL and LTC services. - While working in health care this past year has certainly brought its challenges, there have also been opportunities. The broad interest in HCAP has shown that rather than shying away from health care, the opposite appears to be true in that people want to be part of the field. - While having taken a system-wide engagement approach, HCAP focuses on current and immediate future needs. It is highly dependent on the supplementary funding from the government and questions will remain as to how long funding will be available. |
| Community Integrated Care (2021) Unfair to care: understanding the social care pay gap and how to close it: full report. pp. 64. Epsom, UK.https://www.unfairtocare.co.uk/wp-content/uploads/2021/07/Unfair-To-Care-Full-Report-Single-Pages.pdf | II- Research (Mixed method) | B- Costs and benefits of social care workforce (England) | Theme 4- Sector economic perspectives (funding/resourcing rising home care costs, why and how to pay for home care) | - A report on the social care workforce crisis, providing in-depth evidence that frontline carers receive an unjust deal in comparison to other public funded industries and challenging the stereotype that social care is a ‘low-skilled’ sector. This research indicates many social care workers would be paid up to 39% more – an additional £7,000 – if they worked in other public funded sectors. With the role matching or exceeding the level of skill and accountability as professions such as healthcare assistants, police community support officers, and senior teaching assistants, the roots of the workforce crisis are laid bare. - The report also reveals that the sector loses 34% of its workforce every year – the issue of low pay is at the heart of the crisis affecting social care; social care contributes £46bn to the national economy annually; every year, social care creates 1.65m jobs, which each contribute to their local economy. The report calls on the Government to: provide an immediate and fair pay rise to all frontline social care workers, as an initial step towards achieving equity with the public sector and NHS; immediately instruct an expert-led social care workforce review, which has cross-party support – this should deliver a system of fair and objective pay benchmarks and bandings for all roles, to deliver parity with the NHS and other publicly-funded sectors at the earliest opportunity; create a wider strategy to make social care a viable, respected, and sustainable career – this includes a greater focus on learning and development and qualifications, registration, the development of career pathways and improving the image of the sector. |
| Lu, J. (2015) Status Quo, Problems and Countermeasure Research of Home-Based Care Service Market in China—A Case Study of Sichuan Province. Open Journal of Social Sciences, 3, 150-156. doi: 10.4236/jss.2015.311020. | II- Research (Qual) | C- Supply and demand challenges in home care (China) | Theme 4- Sector Economic Perspectives (service market, business models and profit margins) | - This paper makes a case study of Sichuan Province where a large aging population lives. Using social investigation, 350 aged community people were interviewed to discuss the conflict between supply and demand in the current home-based care service market and the problems arising out of the market-oriented operation. In addition, this paper brings about specific counter measures and recommendations, with a view to give full play to the role of market- oriented mechanism in optimized allocation of resources, and to inspire participance of various forces into home-based care service market, thus diversifying the home-based care service providers and provision modes in China, and greatly improving the QOL (quality of life) of the elderly. |
| Rooijackers, T. H., Metzelthin, S. F., van Rossum, E., Kempen, G. I. J. M., Evers, S. M. A. A., Gabrio, A., & Zijlstra, G. A. R. (2021). Economic Evaluation of a Reablement Training Program for Homecare Staff Targeting Sedentary Behavior in Community-Dwelling Older Adults Compared to Usual Care: A Cluster Randomized Controlled Trial. *Clinical interventions in aging*, *16*, 2095–2109. https://doi.org/10.2147/CIA.S341221 | II- Research (Quant) | B- Cost-effectiveness and cost-utility of staff reablement training (Holland) | Theme 4- Sector Economic Perspectives (the need for better economic evaluation methods linked to outcomes) | - Training and supporting homecare staff in reablement aims to change staff behavior from "doing for" to "doing with" older adults and is assumed to benefit the health and quality of life of older adults and reduce healthcare utilization and costs. This study evaluated the cost-effectiveness and cost-utility of the staff reablement training program "Stay Active at Home" (SAaH) from a societal perspective. - An economic evaluation was embedded in a 12-month cluster randomized controlled trial. Ten Dutch homecare nursing teams participated (n = 313 staff members), of which five teams were trained in reablement and the other five provided usual care. Cost and effect data were collected from 264 older adults at baseline, 6 and 12 months. Costs included "intervention," "healthcare," and "patient and family" costs (collectively, societal costs) and were assessed using questionnaires and client records or estimated by bottom-up micro-costing. Effects included sedentary behavior and quality-adjusted life years (QALYs). Multiple imputed bootstrapped data were used to generate cost-effectiveness planes and acceptability curves. - No statistically significant differences were observed between the intervention and control group in terms of sedentary time (adjusted mean difference: 4.8 minutes [95% CI -26.4, 36.0]), QALYs ( 0.01 [95% CI -0.03, 0.04]), and societal costs ( €2216 [95% CI -459, 4895]), except lower costs for domestic help in the intervention group ( €-173 [95% CI -299, -50]). The probability that SAaH was cost-effective compared to usual care ranged from 7.1% to 19.9%, depending on the willingness-to-pay (WTP) (€0‒€50,000)/minute of sedentary time averted and was 5.9% at a WTP of €20,000/QALY gained. - SAaH did not improve outcomes or reduce costs and was not cost-effective from a societal perspective compared to usual care in Dutch older adults receiving homecare. Consequently, there is insufficient evidence to justify widespread implementation of the training program in its current form. |
| Connolly, C. (2014). Advanced Direct Care Worker: A Role to Improve Quality and Efficiency of Care for Older Adults and Strengthen Career Ladders for Home Care Workers. Annals of Long-Term Care. https://www.hmpgloballearningnetwork.com/site/altc/articles/advanced-direct-care-worker-role-improve-quality-and-efficiency-care-older-adults-and | III- Review of evidence and professional opinion | B- Advanced direct care worker role (USA) | Theme 4- Upskilling and Delegation (advanced direct care worker, healthcare support, more complex levels of care) | - This article was prepared by the Eldercare Workforce Alliance, a group of 30 national organizations who represent healthcare professionals in America, including direct-care workers, and family caregivers and consumers. The Alliance works toward practical solutions to strengthen the eldercare workforce and improve the quality of care. - The Alliance believes that with the appropriate training, supervision, and support, some home care workers can play an enhanced role, with more responsibility for improving safety and quality of care for older adults and their family caregivers. - Sets out the evidence for the need for Advanced Direct Care. - For advanced direct care workers working with older adults, training specific to geriatric syndromes and the unique needs and symptoms of older adults is necessary for this role to effectively improve outcomes. Additionally, training for the advanced direct care worker in effectively communicating with the interdisciplinary care team, the consumer and family members—as well as for the team members in communicating with the advanced direct care worker—is essential. |
| Denton, M., Brookman, C., Zeytinoglu, I., Plenderleith, J., & Barken, R. (2015). Task shifting in the provision of home and social care in Ontario, Canada: implications for quality of care. *Health & social care in the community*, *23*(5), 485–492. https://doi.org/10.1111/hsc.12168 | II – Research (Qual) | B- Home care worker role (Canada) | Theme 4- Upskilling and Delegation (client healthcare support, more complex levels of care) | - Growing healthcare costs have caused home-care providers to look for more efficient use of healthcare resources. Task shifting is suggested as a strategy to reduce the costs of delivering home-care services. Task shifting refers to the delegation or transfer of tasks from regulated healthcare professionals to home-care workers (HCWs). - The purpose of this paper is to explore the impacts of task shifting on the quality of care provided to older adults from the perspectives of home healthcare workers. This qualitative study was completed in collaboration with a large home and community care organisation in Ontario, Canada, in 2010-2011. Using a purposive sampling strategy, semi-structured telephone interviews were conducted with 46 home healthcare workers including HCWs, home-care worker supervisors, nurses and therapists. - Study participants reported that the most common skills transferred or delegated to HCWs were transfers, simple wound care, exercises, catheterisation, colostomies, compression stockings, G-tube feeding and continence care. A thematic analysis of the data revealed mixed opinions on the impacts of task shifting on the quality of care. HCWs and their supervisors, more often than nurses and therapists, felt that task shifting improved the quality of care through the provision of more consistent care; the development of trust-based relationships with clients; and because task shifting reduced the number of care providers entering the client's home. - Nurses followed by therapists, as well as some supervisors and HCWs, expressed concerns that task shifting might compromise the quality of care because HCWs lacked the knowledge, training and education necessary for more complex tasks, and that scheduling problems might leave clients with inconsistent care once tasks are delegated or transferred. Policy implications for regulating bodies, employers, unions and educators are discussed. |
| Saari, M., Patterson, E., Kelly, S., & Tourangeau, A. E. (2018). The evolving role of the personal support worker in home care in Ontario, Canada. *Health & social care in the community*, *26*(2), 240–249. https://doi.org/10.1111/hsc.12514 | II- Research (Document analysis) | B- Home support worker roles and task shifting (Canada) | Theme 4- Upskilling and Delegation (client healthcare support, more complex levels of care) | - To meet increasing demand for home care, the role of personal support workers (PSWs) is shifting from providing primarily personal and supportive care to include care activities previously provided by regulated health professionals (RHPs). Much of the research examining this shift focuses on specialty programmes, with few studies investigating the daily care being provided by PSWs, frequency of care activities being provided by PSWs, and characteristics of the population receiving more complex tasks. - In 2015, a review of 517 home-care service user charts was undertaken in Ontario, Canada, to: (1) describe the range of tasks being performed by PSWs in home care, (2) identify tasks transferred by RHPs to PSWs, and (3) examine characteristics of service users receiving transferred care. - Findings indicate that normally, PSWs provide personal and supportive care commensurate with their training. However, in approximately one quarter of care plans reviewed, PSWs also completed more complex care activities transferred to them by RHPs. Service users receiving transferred care were older and had higher levels of cognitive and functional impairment. Although there is potential for the expansion of home-care services through increased utilisation of PSWs, healthcare leadership must ensure that the right provider is being utilised at the right time and in the right place to ensure safe and effective quality care. - Thus, several actions are recommended: PSW core competencies be clearly articulated, processes used to transfer care activities from RHPs to PSWs be standardised and a team-based approach to the delivery of home-care services be considered. Utilisation of a team-based model can help establish positive relationships among home-care providers, provide increased support for PSWs, allow for easier scheduling of initial training, and ensure regular reassessments of PSW competence among PSWs providing added skills. |
| Saari, M., Patterson, E., Killackey, T., Raffaghello, J., Rowe, A., & Tourangeau, A. E. (2017). Home-based care: barriers and facilitators to expanded personal support worker roles in Ontario, Canada. *Home health care services quarterly*, *36*(3-4), 127–144. https://doi.org/10.1080/01621424.2017.1393482 | II- Research (Qual) | B- Home support worker role (Canada) | Theme 4- Upskilling and Delegation (client healthcare support, more complex levels of care) | - To accommodate the increasing demand for home care in Ontario, Canada, some care tasks traditionally performed by regulated health professionals are being transferred to personal support workers (PSW). However, this expansion of PSW roles is not uniform across the province. - Between December 2014 and April 2015, barriers, and facilitators to expansion of PSW roles in home care were explored in a series of 13 focus groups. - Home care staff identified seven categories of factors affecting the expansion of PSW roles in home care including: communication and documentation; organization and structures of care; attitudes and perceptions of the expanding PSW role; adequate staffing; education, training, and support; PSW role clarity and variation in practices, policies, and procedures. Addressing barriers and promoting facilitators at the funder and employer levels will enable the provision of safe, effective, and equitable care by PSWs. |
| Henwood, T., Hetherington, S., Purss, M., Rouse, K., Morrow, J., & Smith, M. (2019). active@home: Investigating the Value of a Home Care Worker-Led Exercise Program for Older Adults With Complex Care Needs. *Journal of aging and physical activity*, *27*(2), 284–289. https://doi.org/10.1123/japa.2017-0443 | II- Research (Quant) | Exercise training module for home care workers (Australia) | Theme 4- Upskilling and Delegation (new enhanced roles) Support for client exercise | - Exercise has proven health benefits for older adults independent of age, disability, and disease. However, barriers to exercise participation exist, including travel to, and access to, appropriate facilities and programs. Evidence shows that in-home exercise delivered by allied health professionals can improve physical health and prolong independence among individuals with government supported aged care packages. - A less costly alternative is program delivery by home care workers. However, effective training for workers and resources to guide the consumer is required. This project evaluated an exercise training module for home care workers and a consumer resource to promote in-home exercise participation among older Australians with government supported aged care packages. - Outcomes included a significant improvement in functional capacity as measured by the short physical performance battery (mean increase of 1.4 points), a 19% reduction in participants classified as frail and a reduction in healthcare service access of 47% across the intervention. |
| Fernández-Barrés, S., García-Barco, M., Basora, J., Martínez, T., Pedret, R., Arija, V., & Project ATDOM-NUT group (2017). The efficacy of a nutrition education intervention to prevent risk of malnutrition for dependent elderly patients receiving Home Care: A randomized controlled trial. *International journal of nursing studies*, *70*, 131–141. https://doi.org/10.1016/j.ijnurstu.2017.02.020 | II – Programme evaluation | C- Nutrition training for caregivers (Spain) | Theme 4- Upskilling and Delegation (nutrition support, assessment and prevention of malnutrition) | - The aim of this study was to assess the effect of a nutrition education intervention included in the Home Care Program for caregivers to prevent the increasing risk of malnutrition of dependent patients at risk of malnutrition. - Randomized controlled multicenter trial of 6 months of duration and 12 months follow-up. 10 Primary Care Centers, Spain. Patients enrolled in the Home Care Program between January 2010 and March 2012, who were dependent and at risk of malnutrition, older than 65, and had caregivers (n=190). The nurses conducted initial educational intervention sessions for caregivers and then monitored them at home every month for 6 months. The nutritional status was assessed using the Mini Nutritional Assessment test (primary outcome), diet, anthropometry, and biochemical parameters (albumin, prealbumin, hemoglobin and cholesterol). Other descriptive and outcome measures were recorded: current medical history, Activities of daily living (Barthel test), cognitive state (Pfeiffer test), and mood status (Yesavage test). All the measures were recorded in a schedule of 0-6-12 months. - 173 individuals participated after exclusions (intervention n=101; control n=72). Mean age was 87.8±8.9years, 68.2% were women. Differences were found between the groups for Mini Nutritional Assessment test score change (repeated measures ANOVA, F=10.1), the intervention improved the Mini Nutritional Assessment test score of the participants in the intervention group. The egg consumption (F=4.1), protein intake (F=3.0), polyunsaturated fatty acid intake (F=5.3), folate (F=3.3) and vitamin E (F=6.4) showed significant group time interactions. - A nutrition education intervention for caregivers halted the tendency of nutritional decline and reduced the risk of malnutrition of older dependent patients. |
| Mole, L., Kent, B., Hickson, M., & Abbott, R. (2019). 'It's what you do that makes a difference' An interpretative phenomenological analysis of health care professionals and home care workers experiences of nutritional care for people living with dementia at home. *BMC geriatrics*, *19*(1), 250. https://doi.org/10.1186/s12877-019-1270-4 | II- Research (Qual) | B- Nutritional support and training (England) | Theme 4- Upskilling and Delegation (nutrition support, assessment and prevention of malnutrition) | - People living with dementia at home are a group who are at increased risk of malnutrition. Health care professionals and home care workers are ideally placed to support nutritional care in this vulnerable group. Yet, few, if any studies, have captured the experiences of these workers in respect of treating and managing nutritional issues. - This interpretative phenomenological study aimed to explore the experiences and perceptions of the nutritional care of people living with dementia at home from the perspectives of health care professionals and home care workers. Semi-structured interviews were conducted between December 2017 and March 2018, and supplemented with the use of a vignette outlining a scenario of a husband caring for his wife with dementia. Health care professionals and home care workers were purposively recruited from local care providers in the south west of England, who had experience of working with people with dementia. An Interpretative Phenomenological Analysis (IPA) approach was used. - Seven participants took part including two home care workers, a general practitioner, dietitian, occupational therapist, nurse and social worker. The time in their professions ranged from 3 to 15 years (mean = 8.9 years). - Following analysis, four superordinate themes were identified: 'responsibility to care', 'practice restrained by policy', 'in it together', and 'improving nutritional care'. This group of health care professionals and home care workers recognised the importance of improving nutritional care for people living with dementia at home and felt a responsibility for it. However they felt that they were restricted by time and/or knowledge. The importance of supporting the family carer and working collaboratively was highlighted. - Health care professionals and home care workers require further training to better equip them to provide nutritional care for people living with dementia at home. Models of care may also need to be adapted to enable a more flexible and tailored approach to incorporate nutritional care. Future work in this area should focus on how health care professionals and home care workers can be better equipped to screen for malnutrition, and support changes to nutritional intake to mitigate malnutrition risk. |
| Watkinson-Powell, A., Barnes, S., Lovatt, M., Wasielewska, A., & Drummond, B. (2014). Food provision for older people receiving home care from the perspectives of home-care workers. *Health & social care in the community*, *22*(5), 553–560. https://doi.org/10.1111/hsc.12117 | II- Research (Qual) | B- Food provision in home care (England) | Theme 4- Upskilling and Delegation (nutrition support, assessment and prevention of malnutrition) | - Malnutrition is a significant cause of morbidity and mortality, particularly among older people. Attention has focused on the inadequacies of food provision in institutions, yet the majority suffering from malnutrition live in the community. - The aim of this study was to explore barriers and facilitators to food provision for older people receiving home care. It was a qualitative exploratory study using semi-structured interviews with nine home-care workers in June 2013 employed by independent agencies in a large city in northern England. Data were analysed thematically, based on the principles of grounded theory. - Findings showed that significant time pressures limited home-care workers in their ability to socially engage with service users at mealtimes or provide them with anything other than ready meals. Enabling choice was considered more important than providing a healthy diet, but choice was limited by food availability and reliance on families for shopping. Despite their knowledge of service users and their central role in providing food, home-care workers received little nutritional training and were not involved by healthcare professionals in the management of malnutrition. - Despite the rhetoric of individual choice and importance of social engagement and nutrition for health and well-being, nutritional care has been significantly compromised by cuts to social care budgets. The potential role for home-care workers in promoting good nutrition in older people is undervalued and undermined by the lack of recognition, training and time dedicated to food-related care. This has led to a situation whereby good quality food and enjoyable mealtimes are denied to many older people on the basis that they are unaffordable luxuries rather than an integral component of fundamental care. |
| Johansson, L., Björklund, A., Sidenvall, B., & Christensson, L. (2017). Staff views on how to improve mealtimes for elderly people with dementia living at home. *Dementia (London, England)*, *16*(7), 835–852. https://doi.org/10.1177/1471301215619083 | II- Research (Qual) | B- Nutritional support in home care (Sweden) | Theme 4- Upskilling and Delegation (nutrition support, assessment and prevention of malnutrition) | - Dementia commonly leads to difficulties in performing daily activities, which can also often affect the ability to prepare and eat meals. As a result, formal support to maintain good nutritional intake might be needed, but there is a lack of knowledge concerning how to support older persons with dementia living at home. - The aim of this study was to explore and describe staff views on how to improve mealtimes for persons with dementia who are still living at home. A qualitative descriptive study was performed and data were collected during 2011-2012 through four focus group interviews with staff working in the homes of persons with dementia. Data were analyzed using inductive content analysis. - The participants described several ways to improve mealtimes for persons with dementia and advocated adjustments facilitating the preservation of the persons' independence. Finding suitable actions calls for knowledge about the person and his/her individual situation. Proposed actions were enabling meals at home, taking over, and moving meals outside of the home. In addition, it was found that the types of meals served to these persons should be as familiar to the individual as possible. - The results of this study indicate the importance of using a person-centered approach and meeting the individual needs when supporting people with dementia regarding their meals when living at home. Individualized care in the home may be expensive, however, it is fair to say that people who become malnourished and admitted to hospitals is even more costly. Furthermore, sharing, and reflecting experiences and knowledge can assist staff to identify ways to manage complex situations. Therefore, the use of refection should be a part of staff members' everyday work. |
| McPherson, C. J., Etele, J., Ta, V. C. Y., & Raghubir, A. (2019). Unregulated care providers’ engagement in palliative care to older clients and their families in the home setting: a mixed methods study. *BMC palliative care*, *18*(1), 1-15. | II- Research (Qual) | C- Home care workers and palliative care (USA) | Theme 4- Upskilling and Delegation (palliative care support) | - Unregulated care providers (UCPs) are at the forefront of direct client care in the community. Their services are required to meet the demand for home-based palliative care from a growing older population, yet understanding of UCPs involvement in care is limited. - This study aimed to identify the types and frequencies of tasks performed by UCPs in home-based palliative care to older clients (> 65 years) and their families and to describe UCPs’ engagement in care, and barriers and facilitators to their work. A mixed method approach was used comprising a quantitative retrospective chart review of UCPs’ tasks (n = 66), qualitative content analysis of progress notes from clients’ charts (n = 85), and thematic analyses of in-depth interviews with UCPs (n = 10). A thematic structure was derived from analyses and integration of data from the chart review and interviews. - The themes reflect the physical, affective, and relational aspects of UCPs involvement in the care of clients and families at the end of life. The findings indicate that although a significant proportion (63%) of the 13, 558 UCP tasks identified were directed toward meeting clients’ physical care needs, their presence in the home, made UCPs an important source of information on the client’s condition; observing and appraising the situation. Further, the nature of their work and frequent interactions with clients and families also presented opportunities for UCPs to provide emotional support; a role UCPs felt was integral to their work. - The study highlights the challenging nature of palliative care to older clients and their families whose needs are often complicated, situated within the unique environment of home care where supervision of UCPs is at a distance. Challenges and facilitators to UCPs’ work in this context are discussed with recommendations to support UCPs in their roles. |
| Herber, O. R., & Johnston, B. M. (2013). The role of healthcare support workers in providing palliative and end‐of‐life care in the community: a systematic literature review. *Health & social care in the community*, *21*(3), 225-235. | III- Review of literature | C- Home care workers and palliative care (Scotland) | Theme 4- Upskilling and Delegation (palliative care support) | - Despite the widespread use of Health Care Support Workers (HCSWs) in providing palliative and end-of-life care, there is little information available about their contributions towards supporting patients who want to be cared for at home or to die at home. Between January and April 2011, a systematic review was conducted to address two questions: (i) What particular tasks/roles do HCSWs perform when caring for people at the end of life and their families to comply with their desire to remain at home?; (ii) What are the challenges and supporting factors that influence HCSWs' ability to provide palliative and end-of-life care in the community? Databases searched for relevant articles published between 1990 until April 2011 included CINAHL, EMBASE, PsychINFO, British Nursing Index, Web of Science, Medline and ASSIA. In total, 1695 papers were identified and their titles and abstracts were read. Ten papers met the eligibility criteria of the study. After the methodological quality of the studies was appraised, nine papers were included in the review. Judgements regarding eligibility and quality were undertaken independently by the authors. - The findings indicate that HCSWs invest a great deal of their time on emotional and social support as well as on assisting in the provision of personal care. They are also involved in providing care for the dying, respite care for family members and offer domestic support. - Although it is important to acknowledge the many positive aspects that HCSWs provide, the findings suggest three challenges in the HCSWs role: emotional attachment, role ambiguity and inadequate training. Support factors such as informal peer grief-support groups, sense of cohesiveness among HCSWs and task orientation enabled HCSWs to overcome these challenges. To conclude, induction and training programmes, a defined period of preceptorship, appropriate support, supervision and clearly defined role boundaries may be helpful in reducing the challenges identified in HCSWs' roles. |
| Leonhardt Caprio, A. M., Burgen, D. M., & Benesch, C. G. (2017). Reducing Stroke Readmissions Utilizing a Home Care Based Transitions Coaching Program. Stroke, 48(suppl_1), A23-A23. | II- Programme evaluation | B- Home care-based programme for stroke patients (USA) | Theme 4- Upskilling and Delegation (support for client self-management of chronic conditions, heath coaching, home care-based programmes for specific patient populations) | - The Center for Medicare and Medicaid Services (CMS) has instituted utilization of all-cause, unplanned 30 day hospital readmissions following discharge for ischemic stroke as part of the Hospital Inpatient Quality Reporting Program. While readmission reduction following stroke is a hospital imperative, there is minimal evidence on stroke-specific readmission reduction programs. - This study sought to reduce 30-day readmissions following ischemic stroke through utilization of an established care transitions program in collaboration with our institution’s affiliated home care agency. - The institution’s home care agency has reduced readmission rates in the general medical population utilizing the Coleman Care Transitions Model©. This model incorporates motivational interviewing in visits prior to hospital discharge, with a home visit 24 to 48 hours after discharge, and by telephone at least weekly by a trained transitions coach. The coach ensures understanding of medications, follow up visits, and recognition of red flag symptoms. A one-year pilot program for stroke patients utilizing this established program was initiated in October 2015. Basic stroke training was provided for all coaching staff. Quarterly readmission rates for 1 year prior to the intervention and 6 months following were analyzed. - During the first 6 months of the pilot program 18.2% of patients discharged with a diagnosis of ischemic stroke qualified for and completed the coaching program. Of those in the program 3.3% were readmitted. Overall, there were 15 readmissions in 13 patients, 85% of whom did not receive transitions coaching services. The readmission rate in the year prior to intervention was 7.8%, in the 6 months following 4.5%. These findings represent a 42.7% overall reduction in readmissions (p = 0.06) and a treatment effect of 1.7. - Utilizing the Coleman Care Transitions Model© led to a meaningful reduction of hospital readmissions for patients with ischemic stroke in this pilot program. In an area in which readmission reduction has proven challenging, expanding the availability of a home care-based program for specific patient populations may be an effective strategy. |
| Skills for Care (2013) There for carers: the workforce development needs of carer support workers. Leeds, pp20. | II- Research (Mixed method) | B- Roles and activities of carer support workers (England) | Theme 4- Upskilling and Delegation (supporting informal caregiver/family education) | - Skills for Care undertook a survey to examine the role of Carer Support Workers (CSWs) and their learning and development needs. The aim was to build a picture of what CSWs do day-to-day, the skills, knowledge and qualifications that they have or think they need to have, also to explore their thoughts on their career path. - An electronic survey sent on 19 July 2013 to 109 CSWs and those employing or managing them received 100 responses (61 from CSWs and 39 from managers/employers) by the time it closed on 23 August 2013. Telephone interviews were also conducted with 10 CSWs and 8 managers or employers. - Three-quarters of respondents were female, and two thirds were aged 45-64. Respondents worked primarily in charities (43%) and voluntary organisations (38%); 17% worked in local authorities and the remaining 2% in the health service. - Survey results reveal a complex, skilled role that involves giving emotional support, both in person and by phone, and enabling carers to support themselves (by helping them to organise support). Three-fifths of CSWs undertake Carer’s Assessments and two-fifths give benefits advice. The range of tasks is wide and demands a high skill level across many areas. About 70% of CSWs deal with all carers and do not specialise in carers with one primary issue (e.g. mental health). Despite their lack of status and their under-used skills, CSWs appear to be a stable workforce, most having been in their job for over a year. - Among recommendations are that those organisations employing or commissioning CSW roles need to develop or promote a clear definition and profile of the role and the skills required. The generic nature of most CSWs role needs to be supported by quality supervision and continual learning: Skills for Care should strengthen and make clear its offer to CSWs and their employers. Employers should pay close attention to improving the skills of CSWs in basic counselling skills and the skills and confidence of asking carers whether they wish to continue caring. Employers should also work to recognise and use the under-utilised skills and knowledge of their CSWs to better promote the service and the CSW role. (Edited publisher abstract) |
| Hendrix CC, Landerman R, Abernethy AP. (2013) Effects of an Individualized Caregiver Training Intervention on Self-Efficacy of Cancer Caregivers. *Western Journal of Nursing Research*. 35(5):590-610. doi:10.1177/0193945911420742 | II- Research (Quant) | C- Caregiver training for older cancer patients (USA) | Theme 4- Upskilling and Delegation (supporting informal caregiver/family education) | - Caring for older cancer patients after hospital discharge is challenging and many of their caregivers lack confidence to do so. This study investigated the effects of an individualized caregiver training program on self-efficacy in home care and symptom management. A total of 120 patient–caregiver dyads were randomly assigned to either the treatment (n = 60) or control group (n = 60). The training focused on prevention of infection, pain control, maintenance of nutrition and adequate elimination, and specific care issues identified by the caregiver. The control group received information about community-based resources. - Results revealed a significant increase in self-efficacy after the training in the treatment group but not in the control group. No intervention effect was seen on caregiver’s psychological well-being (depression, anxiety, and quality of life) and patient’s physical symptoms. |
| Walsh, J. E., Lane, S. J., & Troyer, J. L. (2014). Impact of medication aide use on skilled nursing facility quality. *The Gerontologist*, *54*(6), 976–988. https://doi.org/10.1093/geront/gnt085 | II- Research (Quat) | B- Medication Aides in nursing facilities (USA) | Theme 4- Upskilling and Delegation (task shifting) medications assistance | - A number of states have begun to allow skilled nursing facilities to employ medication aides, who have less formal education than registered nurses (RNs) or licensed practical nurses (LPNs), to administer medications. If this results in fewer RNs or LPNs, quality degradation may occur. - This study evaluated the effect of regulations allowing for medication aides on subsequent medication aide use and the effect of changes in medication aide use on other nurse staffing, deficiencies, and Nursing Home Quality Initiative (NHQI) health outcome measures. Staffing levels and inspection deficiencies from the Online Survey and Certification and Reporting System and NHQI data from 2004 to 2010 for facilities from eight southeastern U.S. states are used in instrumental variables models with facility fixed effects. - Facilities in states allowing for medication aide use increased medication aide use with no statistically significant reduction in RN or LPN use. Medication aide use decreased the probability that a facility received a deficiency citation for unnecessary drug use or having a medication error rate greater than or equal to 5% and had no effect on deficiencies for significant or harmful medication errors. Increased medication aide use was associated with fewer pharmacy and total deficiency citations and decreased use of physical restraints; in contrast, more use of medication aides was associated with an increase in the percentage of residents needing help with activities of daily living and losing continence. - This study provides support for state policies that allow skilled nursing facilities to use medication aides. |
| Lee, C. Y., Beanland, C., Goeman, D., Johnson, A., Thorn, J., Koch, S., & Elliott, R. A. (2015). Evaluation of a support worker role, within a nurse delegation and supervision model, for provision of medicines support for older people living at home: the Workforce Innovation for Safe and Effective (WISE) Medicines Care study. *BMC health services research*, *15*, 460. https://doi.org/10.1186/s12913-015-1120-9 | II- Research (model) | A- HSW delegation of medicines care by nurses for low-risk older people (Australia) | Theme 4- Upskilling and Delegation (task shifting) medications assistance | - Describes development and testing of a new workforce model: 'Workforce Innovation for Safe and Effective (WISE) Medicines Care', which enabled nurses to delegate medicines support home visits for low-risk clients to support workers (known as community care aides [CCAs]). Primary study aims were to assess whether the model increased the number of medicines support home visits conducted by CCAs, explore nurses', CCAs’, and consumers' experiences with the CCAs' expanded role, and identify enablers and barriers to delegation of medicines support. - A prospective before-after mixed-methods study was conducted within a community nursing service that employed a small number of CCAs. The CCAs' main role prior to the WISE Medicines Care model was personal care, with a very limited role in medicines support. CCAs received training in medicines support, and nurses received training in assessment, delegation, and supervision. Home visit data over two three-month periods were compared. Focus groups and interviews were conducted with purposive samples of nurses (n = 27), CCAs (n = 7) and consumers (n = 28). - Medicines support visits by CCAs increased from 43/16,863 (0.25 %) to 714/21,552 (3.3 %). Nurses reported mostly positive experiences, and high levels of trust and confidence in CCAs. They reported that delegating to CCAs sometimes eliminated the need for duplicate nurse and CCA visits (for people requiring personal care plus medicines support) and enabled them to visit people with more complex needs. - CCAs enjoyed their expanded role and were accepted by clients and/or carers. Nurses and CCAs reported effective communication when medicine-related problems occurred. No medication incidents involving CCAs were reported. Barriers to implementation included the limited number of CCAs employed in the organisation and reluctance from some nurses to delegate medicines support to CCAs. Enablers included training and support, existing relationships between CCAs and nurses, and positive staff attitudes. |
| Pratt, J. (2016) Career Progression in Care Project, End of project report. Skills for Care. pp.27. Leeds, UK. | II- Programme evaluation | B- Apprenticeship scheme to attract young people into caring careers (England) | Theme 4- Workforce Shortages, Shortfalls and Untapped strengths (pre-service preparation experiences, apprenticeships) | - Reports on the findings of a three-year project to help small and medium sized employers across adult social care implement an apprenticeship programme to help recruitment and career progression. The project was based on evidence that apprenticeships boost productivity by enabling businesses to develop their skills base. A total of 22 small and medium enterprises (SMEs) across the adult social care sector participated in the project. The SMEs operated across a number of areas in adult social care including adult day care, home care, residential care, extra care housing and supported living. Participating organisations were contracted to achieve a minimum of five apprentices over the lifetime of the project. - By the end of the project, there had been a total of 342 Apprenticeships starts, exceeding the original project target of 105. SMEs reported that the benefits of embedding apprenticeships outweighed the challenges. Benefits included: improving the quality of care for people who need care and support; helping to create a learning culture within the organisation; developing a talent pipe line for the future workforce; and providing opportunities help encourage young people to consider a career in the sector. However, there continue to be challenges in generating sufficient interest in Apprenticeship vacancies and recruiting the right individual with the right values for the organisation, including apprentices. The report makes recommendations to help Skills for Care to capitalise on the success of the project and help SMEs overcome challenges in recruitment and retention. |
| Sharman Z. (2014). Recruitment and retention of home support workers in rural communities. *Home health care services quarterly*, *33*(4), 229–243. https://doi.org/10.1080/01621424.2014.964897 | II- Research (Qual | B- Home support worker recruitment in rural communities (Canada) | Theme 4- Workforce Shortages, Shortfalls and Untapped Strengths (recruitment to home care in rural communities) | - This qualitative study examined recruitment and retention of home support workers (HSWs) providing home support in rural communities. Thirty-two participants were recruited across four island-based communities located in British Columbia, Canada. - Thematic analysis of interview data revealed several key themes: (a) how the rural context shapes HSWs' employment decisions and opportunities; (b) why people become (and stay) HSWs in rural communities; and (c) how rurality influences the nature and scope of HSWs' work. These findings suggest that health human resource policies and programs aimed at HSW recruitment and retention should be tailored to characteristics, strengths, and challenges of rural communities. |
| Gatherum, B., O’Reilly, P., Ross, K. (2018) The experience of the experienced: exploring employment journeys of the social care workforce. Scottish Care. pp.26. Ayr, Scotland. Summary in https://scottishcare.org/wp-content/uploads/2020/03/Qualifying-Care-Final.pdf) | II- Research (Qual) | B- Reports on the experienced staff within the care sector (Scotland) | Theme 4- Workforce Shortages, Shortfalls and Untapped Strengths (valuing experience in the workforce) | - Showcases the life and career journeys of nine experienced care staff working across social care services in Scotland. It shares their experiences, motivations and challenges, and looks at how their experience enables people to receive high quality, care and support. It also asks whether these workers feel that their experience is recognised and appreciated. The report contains direct quotes from interviews with the nine care staff who were working across residential care homes, nursing care homes, care at home and housing support services. - Each participant met one of the following definitions of ‘experience’: someone aged 45 or over who had joined the independent care sector within the last 18 months or someone who has worked in the independent care sector for 15 or more years. - The report shows that ‘experience’ can actually mean many things but is a crucial component of delivering high quality care through a confident and competent workforce. The report forms part of the Workforce Matters 4Rs project, an improvement programme exploring the independent social care sector employment journey in Scotland in relation to recruitment, representation, regulation and retention. |
| Skills for Care (2017) Recruitment and retention in adult social care: Secrets of success. Learning from employers what works well. https://www.skillsforcare.org.uk/resources/documents/Recruitment-support/Retaining-your-staff/Secrets-of-Success/Recruitment-and-retention-secrets-of-success-report.pdf | II- Research (Qual) | B- Widening recruitment and diversity (England) | Theme 4- Workforce Shortages, Shortfalls and Untapped Strengths (recruitment strategies) | - Research study amongst adult social care employers with a turnover of less than 10% to explore what it is that they do that they feel contributes to their success in relation to recruitment and retention, and research amongst individual employers to explore their experiences of recruitment and retention. - The results emphasize: Attracting the right people, with the right values, behaviours and attitudes to work in adult social care is vital. Having attracted the right people to apply for your vacancies it is important to select and employ the best people for the jobs. Once the right people have been recruited the priority is to ensure that they stay and grow with you. Retaining good staff is especially important to organisations delivering adult social care as continuity of staff is crucial in delivering high quality care to people who need care and support. |
| Kelly, C., Craft Morgan, J., Kemp, C. L., & Deichert, J. (2020). A profile of the assisted living direct care workforce in the United States. Journal of Applied Gerontology, 39(1), 16-27. | II- Research (Quant) | B- Role of assisted living direct care worker (USA) | Theme 4- Workforce Shortages, Shortfalls and Untapped Strengths (workforce profiles) | - The purpose of this article was to develop a profile of direct care workers (DCWs) in assisted living (AL). - Data from 2014 American Community Survey (ACS) were used to identify the demographic and employment characteristics of AL DCWs. The researchers collected state training requirements for AL DCWs from the administrative rules of state agencies and interviews with state officials. - AL DCWs were more likely than other DCWs to be younger, male, White, English speaking, U.S.-born, never married, and to have attended college. Two Affordable Care Act (ACA)–designated training topics (self-care and the role of the personal care aide) were not required in any state. - AL has tapped a unique pool of workers (i.e., younger, male as well as female, and with some college education) but needs to address its workforce needs via training, improvements to overall job quality, and the development of career opportunities. |
| Chapman, S. A., Greiman, L., Bates, T., Wagner, L. M., Lissau, A., Toivanen-Atilla, K., & Sage, R. (2022). Personal Care Aides: Assessing Self-Care Needs And Worker Shortages In Rural Areas. Health affairs (Project Hope), 41(10), 1403–1412. https://doi.org/10.1377/hlthaff.2022.00483 | II- Research (Quant) | C- Deficit of personal care aides in rural areas (USA) | Theme 4- Workforce Shortages, Shortfalls and Untapped Strengths (recruitment to home care in rural communities) | - Previous research has documented shortages of personal care aides who provide Medicaid home and community-based services, but there are few detailed geographic data to determine the areas of greatest need and assess the availability of personal care aides nationwide. - Using 2013–17 data from the American Community Survey and the Office of Management and Budget, the researchers analysed potential need for personal care aide services among adults and the supply of aides across the US. - Areas with the highest percentages of adults with self-care disability were mainly in the South, and the gap between the potential need for personal care aide services and the aide supply was greatest in southern states. Within states, there were fewer personal care aides per 1,000 adults with self-care disability in the more rural and most rural areas than in the least rural areas. - The authors suggest that wage and benefit increases, improved training and career opportunities, increased flexibility in state Medicaid policies on paid family caregiving, incentives and compensation for travel, and increased data collection and government tracking of workforce data could help boost the supply of personal care aides in rural America. |
| Ben-Galim, D. and Silim, A. (2013) The sandwich generation: older women balancing work and care. Institute for Public Policy Research. London. pp.43 https://www.ippr.org/publications/the-sandwich-generation-older-women-balancing-work-and-care | III- Review of policy and literature | B- Older women employment and caring (England) | Theme 4- Workforce Shortages, Shortfalls and Untapped Strengths (older women in employment and caring) | - Balancing care responsibilities and work is becoming increasingly difficult, particularly for older women. A ‘sandwich generation’ is emerging, whose members are caught between providing care for both grandchildren and ageing parents, often while continuing to earn and pursue their career. - This report provides an overview of the trends in work and caregiving that are affecting older women's lives. While focused on their position and experiences in the workplace, and the influence that the economic crisis has had on their lives, the report also examines the inequalities that underlie the way that older women are – or rather, are not – represented in politics, business and the media. - The authors consider how more progressive policies on parental leave and flexible working, and innovative reciprocal time-banking schemes, could enable older women (and men) to balance a longer and a more fulfilling working life with better-supported caring responsibilities. - Older women of this 'sandwich generation' are more likely than men to have given up work as a result of their greater caring responsibilities; this disparity is particularly acute for older women on low incomes. - Working grandmothers who provide informal childcare for their grandchildren are likely to be younger, in work, and belong to low-income households: 66 per cent of grandmothers who provide between 10-19 hours of childcare a week earn less than £25,999, compared to 25 per cent who earn £44,000 or more. - The labour market position of older women in general has greatly improved over the past 25 years, with a dramatic rise in their employment rate. The recession had a mixed impact: while their employment rates have improved, unemployment - particularly long-term unemployment - has increased. - Mothers aged over 45 are at the vanguard of the rising number of 'maternal breadwinners' (working single mothers and working mothers who earn as much as or more than their partner). - The increasing number of older women in work, combined with an ageing population, means that serious 'care gaps' are emerging in the UK - particularly in childcare. |
| Bryant, J. (2018) Workforce to care force: who cares? IPC discussion paper. Oxford Brookes University. Institute of Public Care. pp.18, Oxford.https://ipc.brookes.ac.uk/files/publications/Workforce_to_Care_Force-IPC_Discussion_Paper_John_Bryant.pdf | V- Commentary | B- Care workforce issues for integrated home and care (England) | Theme 4- Workforce Shortages, Shortfalls and Untapped Strengths (multifaceted care workforce, paid, unpaid, citizen appreciation) | - A discussion paper, from the Head of Integration and Development at Torbay Council, looks at the things that the combined health and social care system can do to improve staffing levels and staff retention rates. It offers a discussion as to a combined approach to the health and social care workforce challenge and how, in part, it can be funded. The author acknowledges that the right workers are at the heart of delivering seamless services to those in need and supports the notion of breaking down the barriers between NHS employees, local authorities, the private and voluntary sectors and those who care informally. He outlines this model as an opportunity for the future, noting that a talented care workforce needs to be based on quality, reliability and affordability, especially in light of increasing pressures on health and social care now and in the coming years. - The report highlights the need to develop a new model of care in the community, part of which would involve redesigning domiciliary care, and would create jobs that are rewarding, provide career opportunities across the public private and voluntary sectors, and help improve people's lives. |
