# Supplementary material for: Exploring a career pathway for home support workers in Ireland: a systematic scoping review of the international evidence
Source: Front Health Serv. 2024 Mar 13;4:1360920. doi: 10.3389/frhs.2024.1360920 (PMC10967662; doi:10.3389/frhs.2024.1360920)
Supplement: Supplementary file 1 [file Datasheet1.zip › Data Sheet 1_v1/Home support worker role terminology.DOCX]

**Supplementary Material: Home support worker role terminology**

**Home support worker:** roles include diverse job titles such as “support workers”_1_, "home support workers" (HSWs) “home care assistants” or "homecare support workers"_2_. Role titles like "community home care assistant" or "community carer" signify varying levels of care experience (e.g., entry level, +1 year, +5 years), or senior positions like "senior carer" (multiple years of experience) or managerial roles like "care manager" within a care team, or describe time commitments (e.g., part-time, full-time, overnight, live-in, weekend), rather than specific knowledge or skill sets_4_.

**Care worker:** The term "care worker" is a less precise term encompassing paid caregiving in various settings like residential care homes/LTRC, community hospitals, and day centers. In contrast, "carer" and "caregiver" typically refer to unpaid, informal caregiving_5_ by family, friends, or neighbours. Ambiguity arises in privately arranged homecare positions like "care assistant" and "home help," which may be considered "domestic worker" roles involving tasks such as cleaning, cooking, childminding, and gardening, potentially including care for older adults or those with disabilities or illness_6_.

**Support worker:** The title "support worker" is more often used to describe roles aimed at assisting individuals of any age with diverse physical abilities, learning disabilities, and mental health needs in achieving greater independence_7_. This support, often termed "assisted living" or "independent living," involves supervision, encouragement, companionship, and guidance. Roles such as "family support workers" assisting children, young people, and families, and "personal assistants" and "shared lives carers" hired privately, also overlap in providing support to individuals in their homes or shared households_8_.

**Health care assistants:** HSW roles differ from health care assistants (HCA), nursing assistants, and registered nurses (e.g., public health nurse, community nurse, palliative care nurse) with specific qualifications and training requirements (see section 5). HSWs also have distinct responsibilities from regulated health professionals (RHPs) like doctors, midwives, health visitors, or social workers, who may provide specialised care at home (referred to as “professional care at home” or “hospital at home”). These distinctions remain significant despite potential overlap (see excluded terms in table 1). The HSW construct excludes roles such as home support by family or informal carers, supportive social networks, general practitioners, medical students, family support workers, social prescribers, data managers, and staff responsible for remote healthcare delivery (as detailed by de Zulueta, 2021).

**Home support providers:** The term is generally used to refer to organisations or services (Sheehan & O’Sullivan, 2023). In Ireland “Draft Regulations for Providers of Home Support Services” and the Department of Health’s 2022 public consultation report_9_ use the terms “providers of home support services” and “home support workers”, whilst recognising many other terms for HSWs are in use.

**Personal assistant:** The terms “personal assistant” (PA) and “PA hours” refer to services provided to adults who are provided for under the disability remit_10_.

**International terminology**: Other terms exist for HSW roles. In Australia, "home care workers" is common, with alternatives like "disability support workers" and "direct care workers" also used, excluding terms like "informal caregiving," "group facilities," or "nursing homes" (Palesy et al., 2018). In Canada, Sweden, Belgium, the UK, and the USA, terms such as "health care assistant," "personal support worker," "home care," and "unregulated care provider" are prevalent (Saari et al., 2018a, 2018b).

**Historical terms:** In the USA, historical terms like "unlicensed assistive personnel" and "professional home health agency staff" reflect the evolving terminology in the field (Kelly et al., 2013; Barter, 1996).

**References**

Barter M. Unlicensed assistive personnel and lay caregivers in the home. Home Care Provid. 1996 May-Jun;1(3):131-3. doi: 10.1016/s1084-628x(96)90022-2.

de Zulueta, P. (2021). Confidentiality, privacy, and general practice: GPDPR and the brave new world of ‘big data’. Br. J. General Pract. J. R. Coll. General Practit. 71, 420–421. doi: 10.3399/bjgp21X717017

Kelly, C. M., Morgan, J. C., & Jason, K. J. (2013). Home care workers: interstate differences in training requirements and their implications for quality. Journal of applied gerontology : the official journal of the Southern Gerontological Society, 32(7), 804–832. https://doi.org/10.1177/0733464812437371

Saari M, Xiao S, Rowe A, Patterson E, Killackey T, Raffaghello J, Tourangeau AE (2018a). The role of unregulated care providers in home care: A scoping review. J Nurs Manag.26(7):782-794. doi: 10.1111/jonm.12613. Epub 2018 Apr 30. PMID: 29708290.

Saari, M., Patterson, E., Kelly, E., Tourangeau, A.E. (2018b). The evolving role of the personal support worker in home care in Ontario, Canada. Health and Social Care in the Community, Volume 26, Issue 2; 240-249 <https://doi.org/10.1111/hsc.12514>

Sheehan, A., O’Sullivan, R. (2023). Draft Regulations for Providers of Home Support Services: An Overview of the Findings of the Department of Health’s Public Consultation. ISBN: 978-1-913829-25-4. <https://doi.org/10.14655/11971-1084904>.

**Footnotes**

1 https://hcci.ie/about/ (Accessed 02 Dec 23).

2 https://www.gov.ie/pdf/?file=https://assets.gov.ie/237210/448892b3-36b4-4b7a-a41e-90368ff2345c.pdf#page=null (Accessed 02 Dec 23).

3 https://ckjobs.ie/guide-to-becoming-a-home-support-worker/#:~:text=To%20become%20an%20HCA%20in,Care%20Skills%20free%20of%20charge (Accessed 02 Dec 23).

4 https://www.ihrec.ie/app/uploads/download/pdf/caring_working_and_public_policy.pdf (Accessed 02 Dec 23).

5 https://ckjobs.ie/guide-to-becoming-a-home-support-worker/#:~:text=To%20become%20an%20HCA%20in,Care%20Skills%20free%20of%20charge (Accessed 02 Dec 23).

6 https://www.citizensinformation.ie/en/employment/types-of-employment/domestic-workers-employment-rights/ (Accessed 02 Dec 23).

7 For an overview of social care roles, see the information developed by Skills for Care, the strategic workforce development and planning body for adult social care in England. https://www.skillsforcare.org.uk/Careers-in-care/Job-roles/Job-roles.aspx (Accessed 02 Dec 23).

8 See ‘HSE Home Support Service for Older People: Booklet and Application Form 2022’ https://www.hse.ie/eng/home-support-services/home-support-services-information-booklet.pdf. (Accessed 02 Dec 23).

9 See Overview of the findings of the Department of Health’s public consultation: https://www.gov.ie/en/publication/56ab1-draft-regulations-for-providers-of-home-support-services-an-overview-of-the-findings-of-the-department-of-healths-public-consultation/ (Accessed 02 Dec 23).

10 https://www.hiqa.ie/sites/default/files/2021-12/Regulation-of-Homecare-Research-Report-Long-version.pdf (Accessed 02 Dec 23).
