# Supplementary material for: Exploring a career pathway for home support workers in Ireland: a systematic scoping review of the international evidence
Source: Front Health Serv. 2024 Mar 13;4:1360920. doi: 10.3389/frhs.2024.1360920 (PMC10967662; doi:10.3389/frhs.2024.1360920)
Supplement: Supplementary file 1 [file Datasheet1.zip › Data Sheet 1_v1/Thematic Code Framework.DOCX]

**Supplementary Material- Thematic Coding Framework**

| **Theme 1: Data and Decision-Making on the Future Workforce:** To address the growing challenges of providing support and care to populations, home care policy, research and planning need to be integrated and coordinated, which requires better decision-making, informed by better workforce data and reporting strategies. Career pathways need to be informed by economic considerations and delivery models, data on where there are shortfalls in the system, and future facing needs for upskilling, protecting public health and communities, and knowledge and skills to utilise digital technologies in home care.  **(95 Articles)** | |
| --- | --- |
| **Subthemes (n=8)** | **Issues (What works/doesn’t work for who, when and why?)** |
| Policy, Research and Planning  (29 Articles) | Policy goals and priorities for future home care workforce |
|  | Identifying priorities for sector development |
|  | Certification, registration, professional licensure |
|  | Legality of home care work and roles |
|  | Research gaps around skills levels and gaps, effective support for client groups |
|  | Research needed to assess staff and client safety together |
|  | Research on policy and community-level support for home support workers |
|  | Regulation systems, reporting requirements and quality control |
|  | Systems thinking, systemic challenges and change |
|  | Research groups and networks to inform policy and practice |
|  | Professional core competencies and training requirements |
|  | Job design (new enhanced roles) |
|  | Occupational status (lack of key worker status and privileges, professional status) shifts in social and professional relations |
|  | Workforce data, minimum data sets (workforce demographics, working patterns) |
|  | Workforce planning (understanding demand and supply needs) |
|  | Local community, place-based approaches to home care, working with anchor institutions such as housing associations |
|  | The need for planning levels linked to outcomes |
| Sector Economic Perspectives  (5 Articles) | Costs and benefits of home care sector, societal costs |
|  | Funding/resourcing rising home care costs, why and how to pay for home care |
|  | Sustainability, funding relationship between state, a family and the individual |
|  | Meeting the costs of home care nationally, cost contributions, direct payments |
|  | Reduction of costs to other sectors |
|  | Service market, business models and profit margins |
|  | The need for better economic evaluation methods linked to outcomes |
|  | Cost-benefit of large-scale policy-directives or workforce development interventions |
| Models of Home Care Provision  (14 Articles) | Time-and-task based (doing for), activities of daily living, instrumental activities |
|  | Rights, needs and eligibility for home care |
|  | Engagement and early intervention to avoid deterioration |
|  | Outcomes-focused approaches |
|  | Assisted living, independent living (doing with/supervising doing) |
|  | Reablement, recovery or restorative home care (encouraging doing) |
|  | Client centered care, person-centred care |
|  | Client directed care, personalization |
|  | Relationship-based home care, relational care, matching on interests |
|  | Companionship |
|  | Live-in workers |
|  | Dementia support |
|  | Frailty support, complex vulnerabilities and preventative care |
|  | Supporting couples, co-dependency, kinship care |
|  | Gerontology/nurse-led home care |
| Employee Voice, Engagement and Involvement  (13 Articles) | Home care sector voice/representation in policy, research, and planning |
|  | Workforce development design |
|  | Learning strategies, challenges and needs |
|  | User-centered and theory-based design of training programmes |
|  | Tailored and participative development of staff health interventions |
|  | Home care research and learning networks |
| Workforce Shortages, Shortfalls and Untapped Strengths  (8 Articles) | Attracting a new generation of workers, intention to join a caring profession |
|  | Workforce profiles (demographics and characteristics of people in job roles) |
|  | Underrepresented staff groups, widening diversity of the workforce |
|  | Recruitment of men to home support roles |
|  | Older women in employment and caring (time-banking schemes) |
|  | Valuing experience in the workforce |
|  | Recruitment to home care in rural communities |
|  | A multifaceted workforce: employed (private/public/voluntary), self-employed, retired, unpaid, citizen appreciation, unemployed, student |
| Public Health, Disaster Preparedness, Crisis Management  (4 Articles) | Reduction of healthcare pressures, correct utilization/reduced readmissions |
|  | Potential for health promotion and healthy ageing interventions |
|  | Frontline essential care, learning from Covid-19 |
|  | Disaster preparation (service continuity through adversity) |
| Upskilling and Delegation (new enhanced roles)  (16 Articles) | Advanced direct care worker, healthcare support, more complex levels of care |
|  | Support for client self-management of chronic conditions, heath coaching, home care-based programmes for specific patient populations |
|  | Client mental health and emotional support |
|  | Medications assistance (delegated/supervised medicines assistance) |
|  | Palliative care support |
|  | Nutrition support, assessment and prevention of malnutrition |
|  | Supporting informal caregiver/family education (caregiver skills training) |
| Digital Technology and Digital Skills in Home Care  (6 Articles) | Digital communication technologies in home care, electronic care plans |
|  | Technology adoption, ensuring devices are accessible and usable |
|  | Digital skills and use of digital technologies for home care skills development |
|  | Robots for medications administration |
|  | Helping clients and families to use communication technologies |
|  | Functional ability technologies |
|  | Use of the internet in home care (remote/virtual care and patient monitoring) |

| **Theme 2) Attracting and Developing a Competent and Motivated Home Care Workforce:** In the context of international home care supply shortages, providers and purchasers must find solutions to attract and facilitate take-up of jobs by suitable candidates. As well as using pay and financial incentives, structures for training and professional development show that values, competence, individual career progression, and retention of knowledge and skills in the sector, are vital for growth and sustainability.  **(83 Articles)** | |
| --- | --- |
| **Subthemes (n=8)** | **Issues (What works/doesn’t work for who, when and why?)** |
| Preparation, entry, early experiences (Career Starters)  (6 Articles) | Promoting and valuing home care as purposeful, meaningful, rewarding work |
|  | Qualifications and requirements for job roles (references, checks) |
|  | Certification, license to practice |
|  | Job application processes |
|  | Candidate’s essential skills, desirable experience for job roles |
|  | Values for home care, humanistic care abilities |
|  | Specifics of the job role, responsibilities, and rights |
|  | Preparatory/induction programmes, new starter support systems |
|  | Early experiences and drop-out/motivations to stay |
| Core Competencies Training for Home Care Work (Early Career Home Support Workers)  (12 Articles) | Recognising medical emergencies and acute care needs |
|  | Person centred care, empathy training |
|  | Personal care training, ADL, IADL, infection control, oral health care |
|  | Personal assistance (instrumental activities, supervised activities) |
|  | Mental health awareness |
|  | Safety in the home (falls prevention) |
|  | Communication with clients, cross-cultural communication |
|  | Communication with clinicians/healthcare professionals |
|  | Disability/ different ability training (hearing loss, visual impairment, neurodiversity) |
|  | LGBTQI+ awareness training |
|  | Cultural competency training, diversity training |
|  | Health literacy (ability to obtain, read, understand, use health information) |
| Advanced or Client Group Specific Training (Individual Contributors) Higher development awards  (35 Articles) | Care of the elderly, aged care, adult social care training |
|  | Dementia care training (virtual reality, Alzheimer’s disease, young onset) |
|  | Heart failure training |
|  | Hyper-tension training |
|  | Cancer care training |
|  | Post-hospitalization care, recovery |
|  | End-of-Life training, palliative care (paediatric, adult) |
|  | Diabetes care training |
|  | Long-term care |
|  | Complex health needs (cancer and dementia) |
| Leadership, Governance and Management Training (People Management)  (1 Article) | Supervisor training |
|  | Care manager training |
|  | Home care administrator/service management education |
|  | Leadership skills |
| Perceived Career Success  (Career Achievers/Leavers)  (1 Article) | Economic security (financial stability, employment stability) |
|  | Supportive work environment (feeling fulfilled, feeling positive about oneself, feeling supported by/connected to co-workers) |
|  | Suitable career fit (having idiosyncratic needs met, pursuing meaningful work, experiencing personal and professional growth) |
|  | Reasons to leave (characteristics of those who leave home care) |
| In-service Learning Opportunities  (Continuous professional development)  (12 Articles) | Using information, policies, and guidance on best practice, decision aides |
|  | Opportunities to clarify expected standards and consistency of practices between staff |
|  | Opportunities to follow up on training and embedding training in practice |
|  | Mandatory training |
|  | Using appropriate learning approaches to gain skills and knowledge |
|  | Supervisor support and climate for innovation |
|  | Workplace learning led by community colleges, living classroom |
|  | Mentorship programmes (mentee, mentor training) |
|  | Opportunities and time for peer learning using group reflective practices |
|  | Opportunities for practice learning, organisational learning, innovation |
|  | Self-instruction online courses |
|  | Training that enables communities of practice |
|  | Nurse-led interventions to enhance care worker competencies |
|  | Professional supportive networks (within and beyond organisations) |
| Career Development and Progression Systems  (9 Articles) | Personal career aspirations, goals, preferences, objectives, home care intent |
|  | Barriers and challenges to career progression |
|  | Appraisals, progress reviews |
|  | Organisational approach to career progression and development strategies |
|  | Supportive work environment (adequate staffing, study time and flexibility) |
|  | Suitability for roles/essential criteria for role progression or attaining licensure |
|  | Transition to advanced roles (client groups and competencies/skill sets) |
|  | Career planning tools and career journey stories and inspiration |
|  | Information about educational programmes and courses |
|  | Job opportunities and information (equality of career opportunities) |
|  | Mentorship programmes |
|  | Participation in continuing professional development (CPD) |
|  | Career development awards, funding for training or development activities |
|  | Retirement planning, continuation planning |
| Cost-Benefits of Employment and Training  (7 Articles) | Costs of courses for individuals and organisations |
|  | Entry level pay, job availability |
|  | Occupational mobility at entry-level |
|  | Pay progression, pay and conditions banding/pay scales |
|  | Earning potential, minimum hour/wage payments, milage payments |
|  | Employee time/costs associated with meeting new training standards |
|  | Sick pay, employee benefits, pensions |
|  | Cost-Benefits of training or certification |

| **Theme 3) Enhancing Working Lives and Retention At Every Stage of Career Pathways:** Individual job satisfaction and feeling valued are recognised globally as key factors for developing a sustainable workforce. Staff are more likely to stay and feel valued when there is a safety culture that minimizes risks and harms associated with demanding job roles and home settings, when their roles and boundaries are clear, when the workload is manageable, when there is time to complete tasks, when they have good working relationships with colleagues, supervisors and managers, when they are encouraged to voice any concerns or suggestions about their work in confidence without fear of negative repercussions, and when they feel they have a say in decisions that affect their work.  **(53 Articles)** | |
| --- | --- |
| **Subthemes (n=7)** | **Issues (What works/doesn’t work for who, when and why?)** |
| Personal Safety and Wellbeing  (15 Articles) | Risks to personal safety, aggression, violence, abuse, harassment, racism (in homes and communities) |
|  | Travel and driving related risks |
|  | Risks of occupational injury (safe handling, physical work, ergonomic hazards) |
|  | Risks to health (tobacco smoke/pollutants, unrestrained pets, infestations/pests) |
|  | Stress, burnout, emotional labour |
|  | Organised emotional support, clinical supervision |
|  | Personal supportive social networks, family and friends |
|  | Coping with client death and bereavement |
|  | Isolation/supportive networks (peer, social, family) |
|  | Sharing work experiences/debriefing |
|  | Quality work environments, supported autonomy, anti-discriminatory practice |
|  | Organisational systems for monitoring personal safety and wellbeing |
|  | Workplace health promotion interventions (exercise) |
| Safety in the Home Setting  (8 Articles) | Home layout, accessibility, and home safety risks |
|  | Physical restraints (prohibited practices, family understanding) |
|  | Client’s acute symptoms/vital signs and health emergency protocol |
|  | Dementia safety |
|  | Falls prevention |
|  | Organisational systems and support structures for safety in homes |
|  | Organisational systems and support structures for emergency situations |
|  | Safety markers for home care |
| Role Clarity, Preventing Role Drift and Having Clear Boundaries  (4 Articles) | Role uncertainty, role boundary-challenging behaviours |
|  | Role descriptions |
|  | Professional relationships and emotional bonds with clients |
|  | Role drift into unagreed, unsafe, or unlawful practices |
|  | Voluntary labour, ‘gifted’ hours, working beyond paid hours |
|  | Care left undone/insufficient time to care (malnutrition, dental carries) |
| Job Satisfaction and Retention  (13 Articles) | Factors associated with job satisfaction (relationships with management, leadership, paid time for training, recognition, and rewards) |
|  | Psychosocial work factors, work group climate, sense of mastery, job control, overall job strain, frustrated empathy, balancing competing needs, balancing emotional involvement, lack of recognition |
|  | Preferred working hours, shift patterns, contracted hours, guaranteed hours |
|  | Employee satisfaction indicators, feedback systems, measurement |
|  | Factors that promote good work and retention (intrinsic, extrinsic motivation) |
|  | Self-efficacy, control, confidence in practice |
|  | Employee morale, camaraderie |
|  | Resilience and joyful moments of work |
| Organisational Economic Perspectives  (3 Articles) | Pay levels linked to experience/time in service/training achieved |
|  | Efficiency of organisations and care manager’s role and behaviours |
|  | Changing cultures of unpaid work/expectations to go the extra mile |
|  | Recognition for service and commitment to the profession |
| Care Planning, Care Plans and Coordination of Care  (5 Articles) | Standardised care plans, personalised care |
|  | Home care plans (holistic needs and assessment) |
|  | Patient care plans/nursing care plans |
|  | Culturally competent organisational policies and practices |
|  | Communication and coordination of care |
| Inclusion and Voice in Teams, Organisations and Policymaking  (5 Articles) | Marginalisation of the workforce, lack of involvement in levels of decision making, gendered constraining versus enabling workplace learning cultures |
|  | Vulnerability to exploitation |
|  | Inclusion in teams and their decision making |
|  | Being able to express views on quality care |
|  | Lack of policy influence or control over policy impact |
|  | Preventing unintended negative impact of innovations in home care |

| **Theme 4) Career Pathways to Improve Quality and Impact:** Career pathways extend and recognise the capabilities and competencies of home support workers to deliver good quality well organised care. Career development at the individual level will advance a sector through the collective knowledge and skills to interface with other providers or services, implement service quality indicators, regulate standards, support the career needs of migrant workers, work more effectively with families and caregivers, and maximise the benefits of home care for the health system and other sectors.  **(30 Articles)** | |
| --- | --- |
| **Subthemes (n=6)** | **Issues (What works/doesn’t work for who, when and why?)** |
| Structural Organisation of Care and Jobs (‘Organizing for Quality’)  (13 Articles) | Assessing levels, types and complexity of client care and support needs |
|  | Adherence to agreed job roles (part-time, full-time, weekends, overnight, live-in) |
|  | Staffing policies, staff allocation models, rostering |
|  | Staffing to support safety |
|  | Staffing to support job satisfaction and retention |
|  | Staffing to avoid occupational injury, stress, sick leave, disability retirement |
|  | Sufficient time for work, client interactions and interpersonal sensitivity |
|  | Casualisation of labour, disconnect between theory and working conditions |
| Interface with Other Services or Providers  (1 Article) | Integrated/joined-up care as part of health systems |
|  | Working with healthcare and other professionals, hospital-at-home |
|  | Voluntary and community organisations |
| Service Quality Indicators and Measures  (4 Articles) | Home care quality indicators, Motivation and Responsibility for Quality (individual, organisation, sector) |
|  | Invisibility and undervaluing of relational care |
|  | Safety indicators |
|  | Service regulatory requirements |
|  | Governance and reporting |
| Employment of Migrant Workers  (6 Articles) | Reliance on migrant workers |
|  | Impact of migrant workers on caregivers |
|  | Support and career opportunities for migrant workers |
|  | Migrant worker carer burden |
|  | Health issues of migrant workers |
| Families and Caregivers  (4 Articles) | Support to find paid help and arrange suitable home care |
|  | Family preferences towards paid providers/paid family caregivers |
|  | Differences in the impact of paid carers on family caregivers |
|  | Caregiver’s experiences of paid help |
| Service Economic Perspectives  (2 Articles) | Costs and benefits of workforce training in long-term care |
|  | Costs of home care for older people, cost savings compared to institutions |
|  | Economic benefits of staff reablement training |
|  | Cost associated with home care worker role and levels of support and care |
|  | Assessing client complexity, higher support and care costs, premium payments |
